# Integrating network pharmacology and experimental verification to explore the pharmacological mechanisms of Guanxin Shutong capsule in treating heart failure

**DOI:** 10.1097/MD.0000000000040118

**Published:** 2024-10-18

**Authors:** Zheming Yang, Jiayin Li, Haixu Song, Hanlin Wu, Shuli Zhang, Zhu Mei, Yu Xue, Xiaolin Zhang, Chenghui Yan, Yaling Han

**Affiliations:** a College of Medicine and Biological Information Engineering, Northeastern University, Shenyang, Liaoning, China; b Department of Cardiology, State Key Laboratory of Frigid Zone Cardiovascular Diseases (SKLFZCD), Cardiovascular Research Institute, General Hospital of Northern Theater Command, Shenyang, China.

**Keywords:** Guanxin Shutong, heart failure, molecular dynamics simulations, network pharmacology, VEGFR2/AKT/eNOS signaling pathways

## Abstract

The Guanxin Shutong capsule (GXST), a traditional Chinese medicine, is commonly used for treating cardiovascular disease, it has shown efficacy in improving symptoms and enhancing the quality of life for patients with heart failure (HF). However, the specific mechanism of action of GXST in HF remains unclear. In this study, we employed a comprehensive approach combining network pharmacology, molecular dynamics (MD) simulations, and in vitro validations to investigate the potential targets and molecular mechanisms of GXST against HF. We collected active ingredients and target genes of GXST, as well as related genes of HF, from multiple public databases. Using bioinformatics analysis, we constructed networks of ingredients-disease-targets and performed functional annotations of the core targets. MD simulations were conducted to verify the binding between the core protein–ligand complexes. In vitro evaluations, including cell proliferation, apoptosis, and protein expression in human umbilical vein endothelial cells (HUVECs) and H9C2 cells were treated with GXST, were performed for pharmacodynamics evaluation. Network analysis revealed 320 intersection genes and 74 active ingredients in the Herbs-ingredients-target genes-disease network. We identified key active ingredients and target genes that overlapped. The KEGG pathways of the intersection genes were primarily enriched in the PI3K-Akt signaling pathway and apoptosis. The protein–protein interaction network highlighted proteins such as AKT1, VEGFR2, and eNOS. MD simulations confirmed stable docking and lower binding energy between 4 identified ingredients (kaempferol, quercetin, (2R)-5,7-dihydroxy-2-(4-hydroxyphenyl) chroman-4-one, and ellagic acid) and their respective core proteins (VEGFR2, eNOS, and AKT). In vitro experiments demonstrated the protective effects of GXST against H_2_O_2_-induced apoptosis in both HUVECs and H9C2 cells. Notably, consistent with the in silico predictions, GXST effectively activates the VEGFR2/AKT/eNOS signaling pathways in HUVECs. This study provides insights into the underlying mechanism of GXST’s therapeutic effects in heart failure. The involvement of the VEGFR2/AKT/eNOS signaling pathways suggests their importance in further elucidating and applying GXST in the clinical treatment of heart failure.

## 1. Introduction

Heart failure (HF) is a clinical condition that results from the structural remodeling and dysfunction of the heart due to various causes, and it is a major cause of death in cardiovascular disease.^[1]^ Despite the availability of western medicine treatments such as diuretics, cardiotonics, vasodilators, and angiotensin-converting enzyme inhibitors,^[2,3]^ the high incidence rate and mortality, multiple complications, recurrent disease, poor drug tolerance, and other factors associated with heart failure often compromise the efficacy of these treatments.^[4,5]^

Traditional Chinese medicine (TCM), with a history of over 2000 years, has its unique therapeutic approach using medicinal formulas that have gained widespread clinical application.^[6]^ TCM is characterized by multiple targets and fewer adverse reactions and has been used for a long time to treat cardiovascular disease.^[7,8]^ One such medication that has gained attention in the treatment of cardiovascular disease is the Guanxin Shutong capsule (GXST). Composed of a combination of 5 Chinese herbs, including *Choerospondias axillaris* (Guangzao), *Salvia miltiorrhiza* (Danshen), *Syzygium aromaticum* (Dingxiang), borneol (Bingpian), and concretio silicea bambusae (Tianzhuhuang), GXST has been widely utilized for many years. Notably, several studies have reported significant improvements in symptoms and positive therapeutic effects associated with the use of GXST.^[9–11]^ Despite its proven effectiveness, the precise mechanisms through which GXST exerts its therapeutic effects in treating heart failure remain to be fully understood. Further research is needed to delve into the underlying molecular pathways and biological processes (BPs) involved in GXST’s pharmacological actions. By gaining a comprehensive understanding of these mechanisms, we can potentially optimize the use of GXST and further enhance its therapeutic benefits for patients with cardiovascular disease.

Network pharmacology utilizes computer information system biology to analyze network data of biological systems and select powerful protein interaction signal nodes for multi-target drug molecule design. TCM network pharmacology is an interdisciplinary field that integrates TCM, effective components of TCM, possible interaction targets of TCM, disease targets, and signal pathway interaction targets information to provide big data analysis for target consistency.^[12,13]^ Molecular docking technology has played a critical role in exploring affinity binding sites of active pharmaceutical ingredients and disease targets,^[14]^ and the development of high-performance computers and parallel computing technology has facilitated the study of protein motion, improving the efficiency of protein structure studies at a quantitative level.^[15,16]^

**Figure 1.**
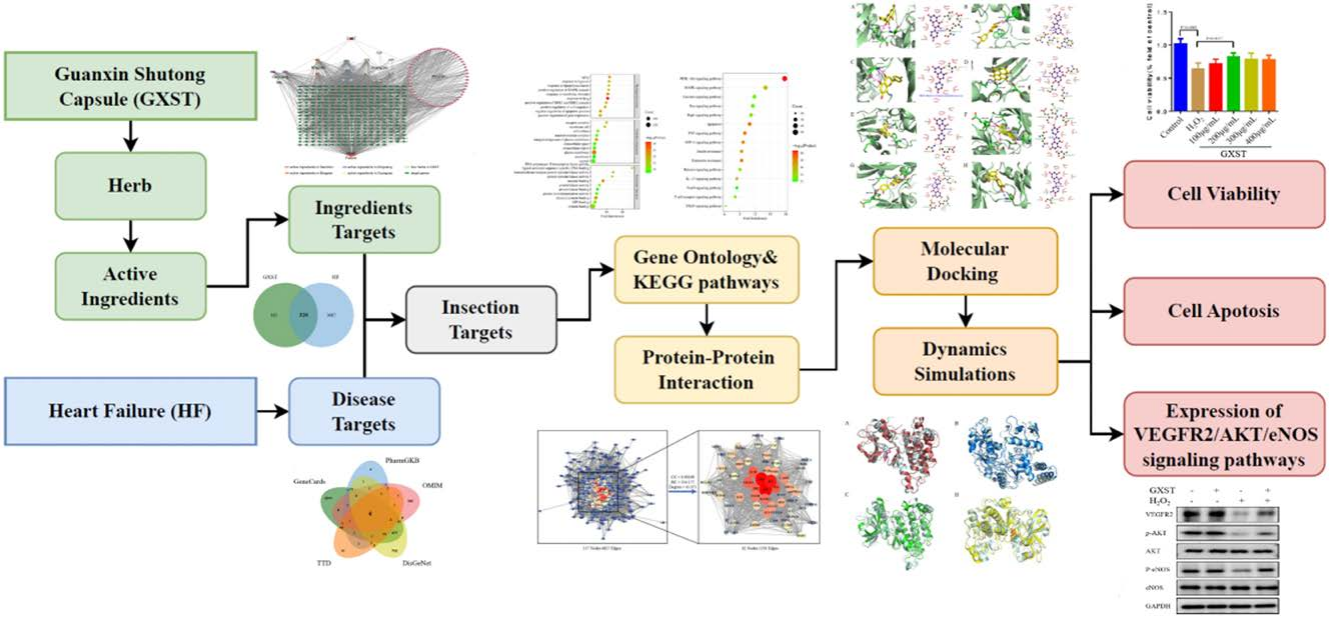
Flowchart of the network pharmacology approach to explore the mechanisms of GXST in the treatment of HF. GXST = Guanxin Shutong capsule, HF = heart failure.

In this study, we utilized the principles, databases, and relevant data processing software of TCM network pharmacology, data statistics, and computer language to verify the low-energy combination mode of effective components and targets through molecular docking and molecular dynamics (MD) simulation. Additionally, we conducted experimental verification to explore the effective active components, core action targets, and potential mechanism of GXST in treating HF. The specific process was shown in Figure 1. This study provides a basis for clinical and experimental research, allowing for a better understanding of the action mechanism of TCM and the development of more effective treatments for HF.

## 2. Results

### 2.1. Identification of active ingredients and targets of GXST

**Figure 2.**
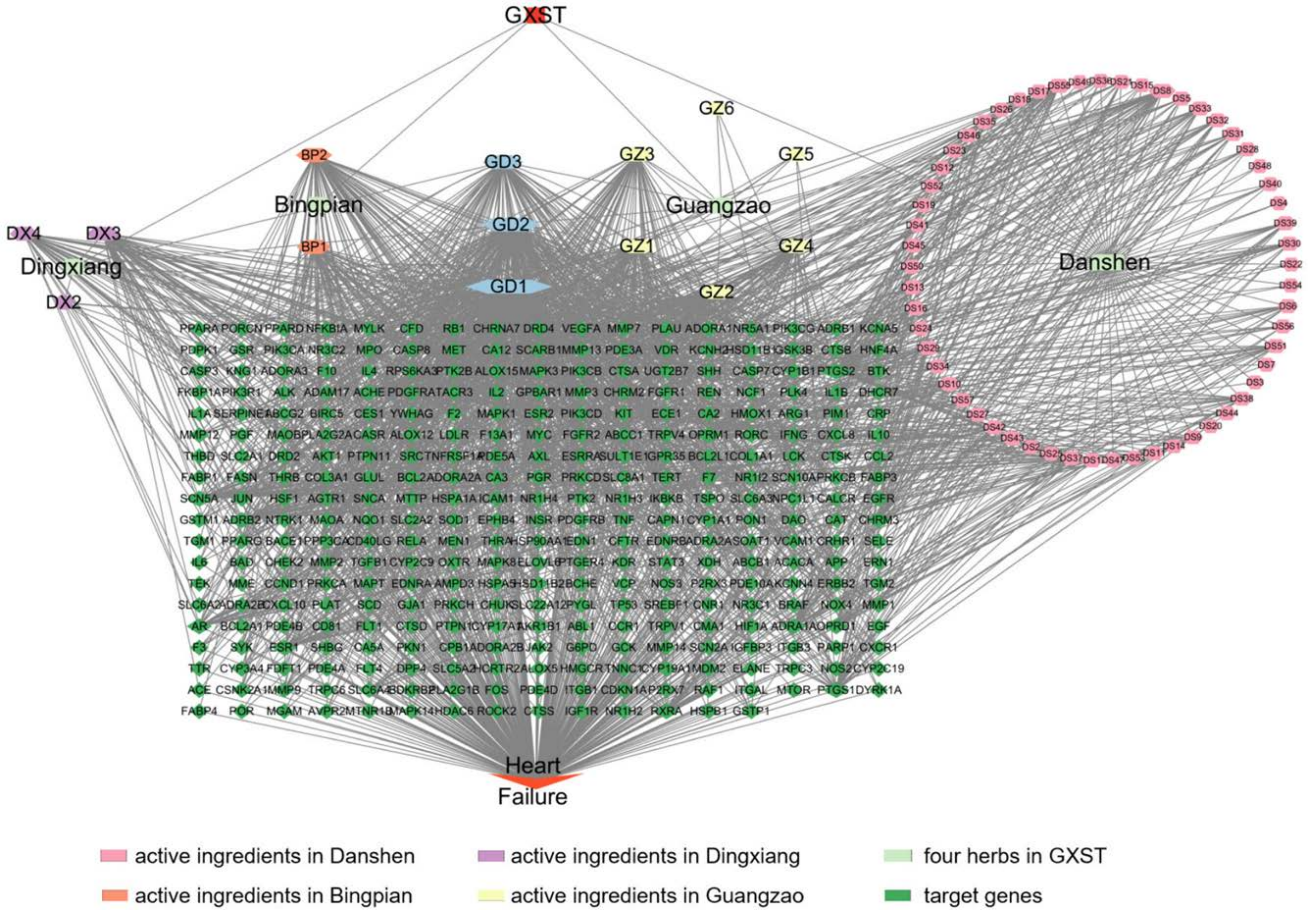
Herbs-ingredients-target genes network. Light green nodes represent 4 herbs in GXST. Pink nodes represent active ingredients in Danshen, orange nodes represent active ingredients in Bingpian, purple nodes represent active ingredients in Dingxiang, yellow nodes represent active ingredients in Guangzao and blue nodes represent active ingredients common to several herbs. Dark green nodes represent target genes for which the active ingredient acts. Edges represent interactions between ingredients and target genes. GXST = Guanxin Shutong capsule.

Eighty-three active ingredients from 5 herbs in GXST were obtained from the TCMSP database based on 2 selection conditions: OB (oral bioavailability) ≥ 30% and DL (drug-likeness) ≥ 0.18. Additionally, the SymMap database was used for obtaining active ingredient data (refer to Table 1 and Table S1, Supplemental Digital Content, http://links.lww.com/MD/N737). Among these ingredients, Guangzao contributed 9 unique active ingredients, Danshen contributed 65, Dingxiang contributed 6, and Bingpian contributed 3. To predict targets associated with these compounds, multiple databases including TCMSP, SWISSTARGET PREDICTION, Pharmaper, and SymMap were utilized. Analysis of the data revealed that out of all the active ingredients, a total of 74 ingredients had identified targets or associations (Table S2, Supplemental Digital Content, http://links.lww.com/MD/N737). This analysis led to the discovery of 2885 GXST targets, duplicate values were merged and deleted, resulting in a final 2115 GXST targets (Table S3, Supplemental Digital Content, http://links.lww.com/MD/N737). To visualize the relationship between drug targets and active ingredients, a herbs-ingredients-target genes network was constructed using Cytoscape software. This network containing 483 target genes. Notably, quercetin (Mol000098), kaempferol (Mol000422), beta-sitosterol (Mol000358), naringenin (Mol004328), and ZINC03860434 (Mol001749) were identified as the top 5 active ingredients based on degree centrality (refer to Fig. 2 and Table S4, Supplemental Digital Content, http://links.lww.com/MD/N737). We will henceforth use GD1, GD2, GD3, GZ1, and DX3 to refer to Mol000098, Mol000422, Mol000358, Mol004328, and Mol001749, respectively (refer to Table S2, Supplemental Digital Content, http://links.lww.com/MD/N737).

**Table 1:** GXST active ingredients list.

No.	Chinese name	Latin name	Class	Active ingredients	OB≥30%	OB≥30% and DL≥0.18	Targets
1	Guangzao	Choerospondiatis Fructus	Blood activating stasis removing drugs	43	19	9	1169
2	Danshen	Salviae Miltiorrhizae Radix Et Rhizoma	Blood activating stasis removing drugs	202	106	65	571
3	Dingxiang	Caryophylli Flos	Warming interior drugs	117	58	6	945
4	Bingpian	Borneolum Syntheticum	Resuscitative stimulant/medicinal	31	19	3	200
5	Tianzhuhuang	Bambusae Concretio Silicea	Phlegresolving medicine	32	18	0	0
Total				425	220	83	2885

DL = drug-likeness, GXST = Guanxin Shutong capsule, OB = oral bioavailability.

### 2.2. Genes of GXST for the treatment of HF

**Figure 3.**
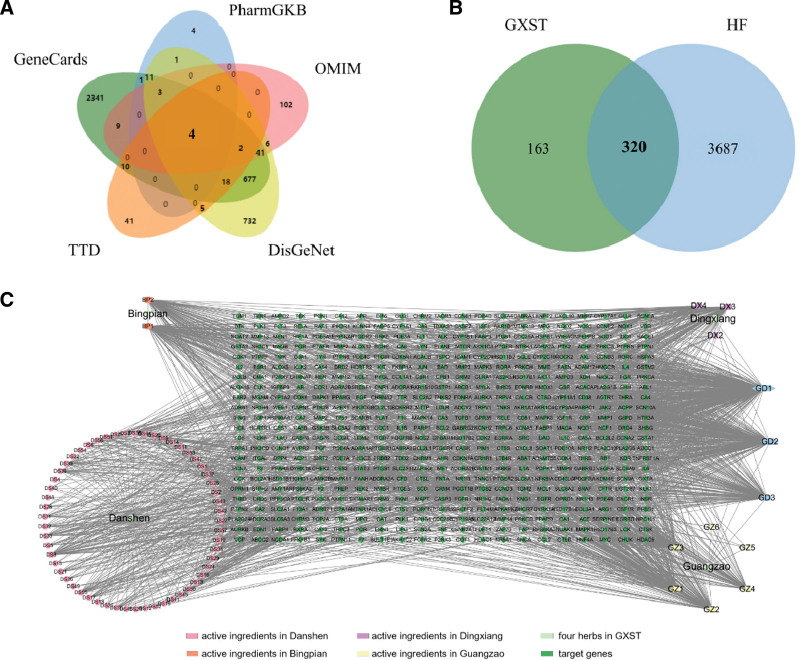
Potential target genes of GXST for the treatment of heart failure. (A) The Venny plot of heart failure-related genes from 5 databases GeneCards, PharmGKB, OMIM, DisGeNet and TTD database. (B) The intersection diagram of GXST predicted targets and heart failure-related targets. (C) Herbs-ingredients-targets-disease network. Light green nodes represent 4 herbs in GXST. Pink nodes represent active ingredients in Danshen, orange nodes represent active ingredients in Bingpian, purple nodes represent active ingredients in Dingxiang, yellow nodes represent active ingredients in Guangzao and blue nodes represent active ingredients common to several herbs. Dark green nodes represent target genes for which the active ingredient acts. Edges represent interactions between ingredients and target genes. GXST = Guanxin Shutong capsule, HF = heart failure.

Based on the keyword of HF, we obtained 4007 nonduplicated HF-related genes from 5 databases GeneCards, PharmGKB, OMIM, DisGeNet, and TTD database over the relevance limits of 5 above (Fig. 3A and Table S5, Supplemental Digital Content, http://links.lww.com/MD/N737). Subsequently, we obtained the overlapping genes between the target genes of GXST and Heart Failure by jvenn software,^[17]^ resulting in 320 collective drug-disease targets for further analysis (Fig. 3B and Table S6, Supplemental Digital Content, http://links.lww.com/MD/N737). Herbs-ingredients–target genes–disease network was performed firstly, which contains 320 intersection genes and 74 active ingredients (Fig. 3C and Table S7, Supplemental Digital Content, http://links.lww.com/MD/N737). Our analysis revealed that PTGS2, SCN5A, PTGS1, ADRB2, and ACHE were the top 5 degrees of potential drug-disease target genes.

### 2.3. Enrichment analysis for GO function and KEGG pathway of intersection genes

**Figure 4.**
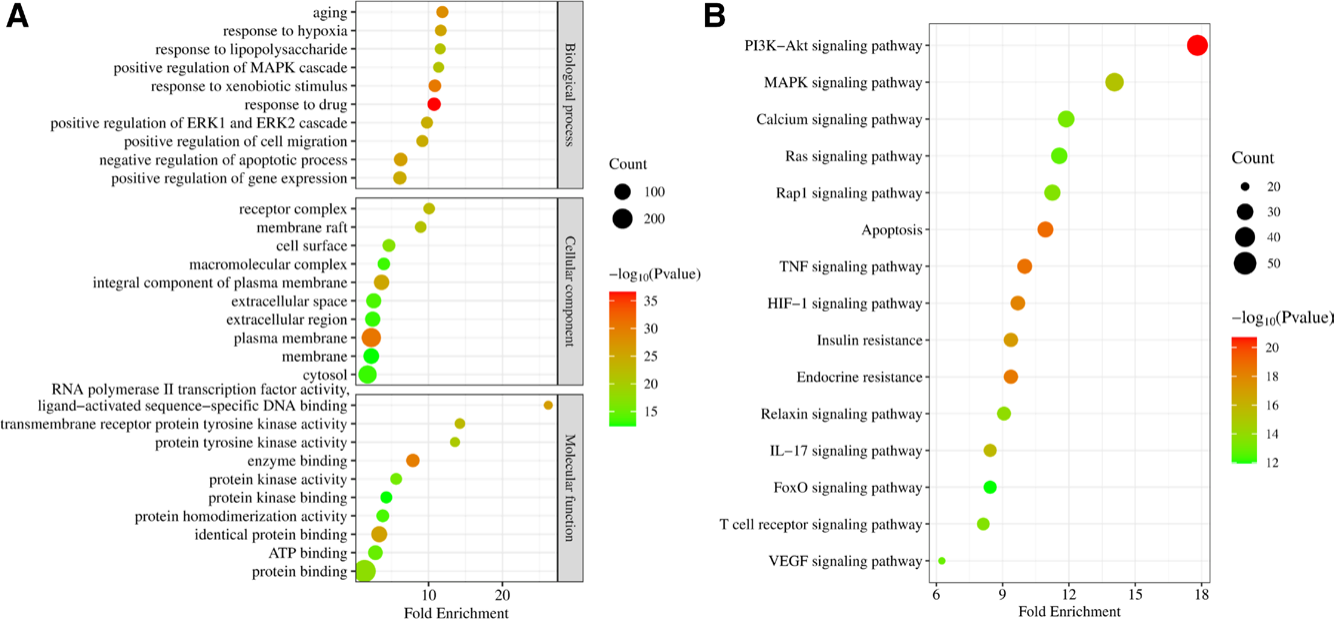
Go and KEGG pathways enrichment analysis. (A) GO-related biological processes, cellular components, and molecular functions enrichment analysis of key targets. (B) KEGG pathways analysis. GO = gene ontology, KEGG = Kyoto encyclopedia of genes and genomes.

To investigate the potential signaling pathways and BPs regulated by GXST on HF, we performed GO-related BPs, cellular component, molecular function (MF), and KEGG pathway enrichment analysis using the DAVID online server. The top 10 for each category in GO function and top 15 disease-related categories for KEGG pathways were represented using RStudio. Among them, BPs are mainly related to response to drug, response to xenobiotic stimulus, aging, negative regulation of apoptotic process, and response to hypoxia. Cellular components result showed that gene expression locations were related to plasma membrane, integral component of plasma membrane, receptor complex, membrane raft, and cell surface. As for MFs, enzyme binding, RNA polymerase II transcription factor activity, ligand-activated sequence-specific DNA binding, identical protein binding, transmembrane receptor protein tyrosine kinase activity, and protein tyrosine kinase activity were mainly involved (Fig. 4A). Moreover, the KEGG pathways were mostly enriched into PI3K-Akt signaling pathway, apoptosis, TNF signaling pathway, endocrine resistance, and HIF-1 signaling pathway (Fig. 4B and Table S8, Supplemental Digital Content, http://links.lww.com/MD/N737).

### 2.4. Identify the core proteins of GXST intervention for HF

**Figure 5.**
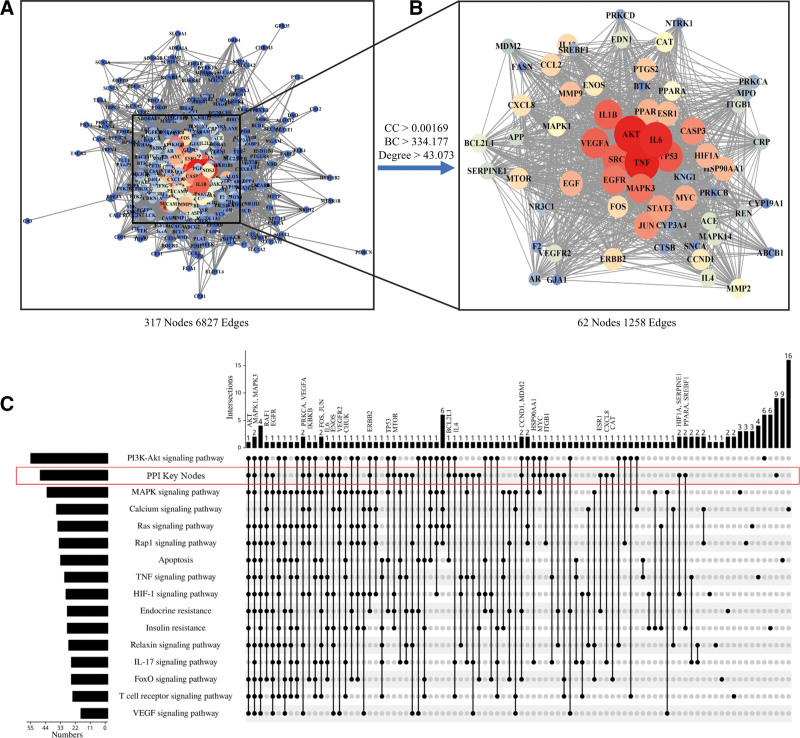
Protein–protein interaction (PPI) network. (A) The interactive PPI network of GXST acting on Heart Failure putative targets. (B) Target proteins screening by the median of DC, BC, CC from A. Each ellipses represent a target protein related to GXST acting on Heart Failure putative targets. The color of nodes is positively correlated with their degrees, respectively. DC denotes connectedness centrality, BC denotes betweenness centrality, and CC denotes compactness centrality. (C) The UpSet graph coupled with target proteins from PPI key nodes and related pathways. BC = betweenness, CC = closeness, GXST = Guanxin Shutong capsule.

To further explore the protein interactions of 320 key target genes that GXST involved in the treatment of HF, the protein–protein interaction (PPI) network was constructed by String database with 317 nodes and 6827 edges after removal of 3 targets without interactions (Fig. 5A). Then, we used the CytoNCA tool of Cytoscape software to calculate network centralities and screen the core proteins based on median values of the betweenness (BC), closeness (CC) and degree as thresholds, respectively. A total of 62 core proteins which closely related to GXST intervention were identified (Fig. 5B). To gain insights into the functional clustering of core proteins, the UpSet plot analysis was performed to compare the intersection between PPI key nodes and related pathways sets (Fig. 5C). As a result, the top 10 genes, namely VEGFR2, eNOS, AKT, MAPK1, PRKCA, IKBKB, IL6, MAPK3, RAF1, and vascular endothelial growth factor A (VEGFA), displayed high degree values and were predicted to be the potential targets for GXST treating HF for further investigation.

### 2.5. Molecular docking and MD simulations of ingredients and core proteins

**Figure 6.**
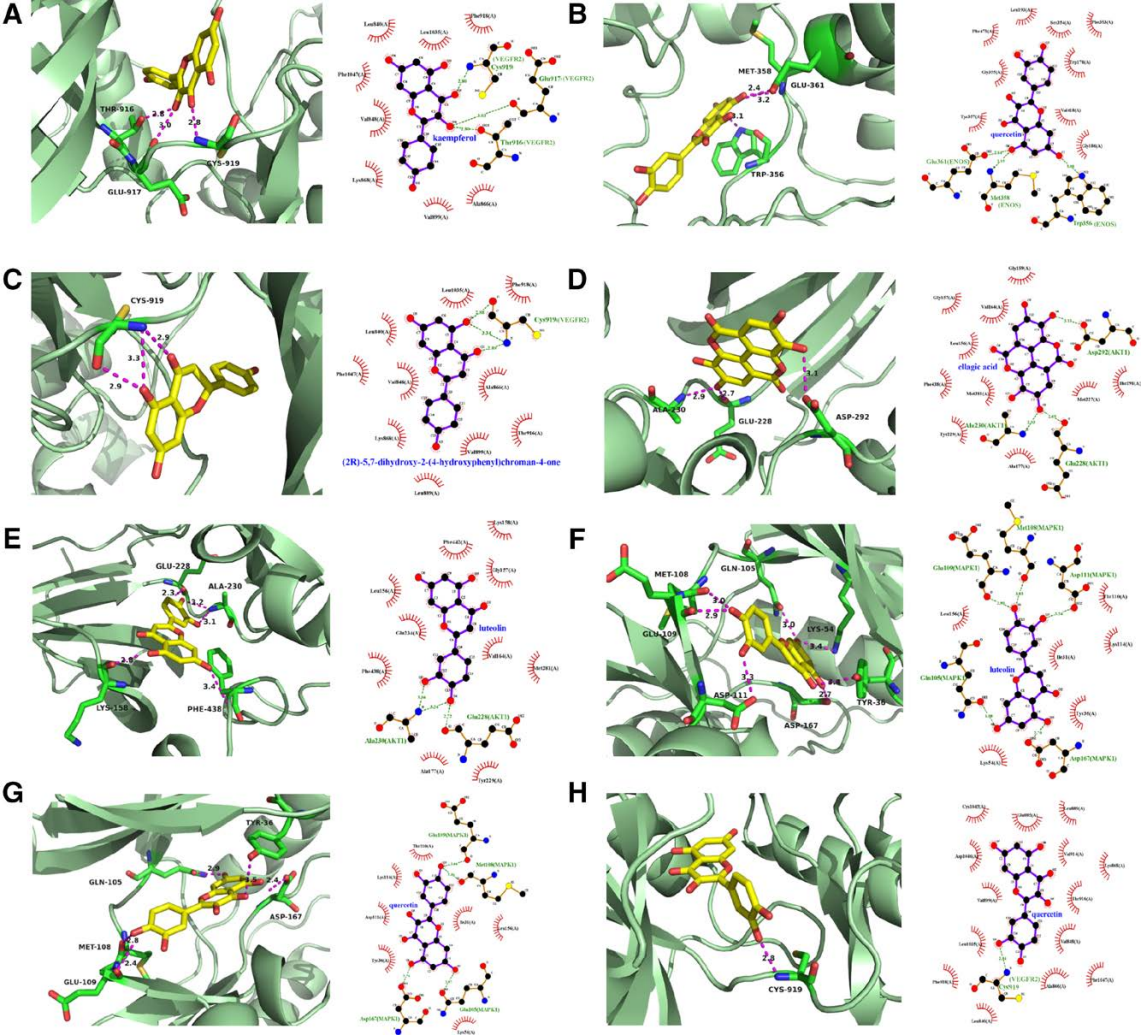
Schematic diagram of docking results and the interaction profile. (A) Results of VEGFR2 with kaempferol. (B) Results of eNOS with Quercetin. (C) Results of VEGFR2 with (2R)-5,7-dihydroxy-2-(4-hydroxyphenyl) chroman-4-one. (D) Results of AKT with ellagic acid. (E) Results of AKT with luteolin. (F) Results of MAPK1 with luteolin. (G) Results of MAPK1 with quercetin. (H) Results of VEGFR2 with quercetin. The green dash lines represent putative hydrogen binding. The arcs represent the putative hydrophobic interactions.

To probe into the potential mechanism of GXST on treating HF, top 10 core proteins were selected to analyze their molecular docking with 13 highly related GXST ingredients using AutoDock Vina. We obtained the crystal structures of VEGFR2 (2RL5), eNOS (6NH7), AKT (6NPZ), MAPK1 (5NHV), PRKCA (4RA4), IKBKB (4KIK), IL6 (1ALU), MAPK3 (6GES), RAF1 (6XGV), and VEGFA (6Z13) from the RCSB PDB database. Binding energy ≤−5.0 kJ/mol was used as the key criterion, with lower energy indicating a more stable interaction. A total of 33 compound-target pairs satisfied the requirements in Table 2. Among them, top 8 compound-target pairs with strong binding efficiency are depicted (Fig. 6). Additionally, we selected the top 4 pairs with low binding energy under ‐7.5 kJ/mol for 50 ns MD simulations.

**Table 2:** Docking parameters and binding energy of potential active ingredients to target proteins.

No	Target	PDB ID	Binding pocket			Compound	MOL ID	Binding Free Energy (kcal/mol)
			center_x	center_y	center_z			
1	VEGFR2	2RL5	64.251	45.695	16.832	Kaempferol	MOL000422	‐9.1
2	ENOS	6NH7	‐17.664	19.762	‐32.504	Quercetin	MOL000098	‐9
3	VEGFR2	2RL5	64.251	45.695	16.832	(2R)-5,7-dihydroxy-2-(4-hydroxyphenyl)chroman-4-one	MOL001040	‐9
4	AKT	6NPZ	‐29.32	‐7.542	19.407	Ellagic acid	MOL001002	‐8.7
5	AKT	6NPZ	‐29.32	‐7.542	19.407	Luteolin	MOL000006	‐8.4
6	MAPK1	5NHV	‐15.847	12.217	41.95	Luteolin	MOL000006	‐8.4
7	MAPK1	5NHV	‐15.847	12.217	41.95	Quercetin	MOL000098	‐8.2
8	VEGFR2	2RL5	64.251	45.695	16.832	Quercetin	MOL000098	‐8.2
9	PRKCA	4RA4	33.558	‐2.764	12.099	Quercetin	MOL000098	‐7.9
10	AKT	6NPZ	‐29.32	‐7.542	19.407	Quercetin	MOL000098	‐7.8
11	AKT	6NPZ	‐29.32	‐7.542	19.407	Kaempferol	MOL000422	‐7.8
12	IKBKB	4KIK	‐10.593	‐8.837	‐83.261	Kaempferol	MOL000422	‐7.8
13	PRKCA	4RA4	33.558	‐2.764	12.099	Beta-sitosterol	MOL000358	‐7.6
14	VEGFR2	2RL5	64.251	45.695	16.832	Ellagic acid	MOL001002	‐7.6
15	AKT	6NPZ	‐29.32	‐7.542	19.407	Naringenin	MOL004328	‐7.2
16	IL6	1ALU	‐0.057	‐29.163	‐12.58	Asiatic acid	MOL006861	‐7.2
17	MAPK3	6GES	61.333	18.333	20.977	Naringenin	MOL004328	‐7.1
18	MAPK1	5NHV	‐15.847	12.217	41.95	Naringenin	MOL004328	‐7
19	MAPK1	5NHV	‐15.847	12.217	41.95	Bronyl acetate	MOL006862	‐6.9
20	VEGFR2	2RL5	64.251	45.695	16.832	Naringenin	MOL004328	‐6.9
21	MAPK3	6GES	61.333	18.333	20.977	Asiatic acid	MOL006861	‐6.7
22	RAF1	6XGV	47.358	46.868	27.845	Quercetin	MOL000098	‐6.3
23	IL6	1ALU	‐0.057	‐29.163	‐12.58	Luteolin	MOL000006	‐6.3
24	IL6	1ALU	‐0.057	‐29.163	‐12.58	Quercetin	MOL000098	‐6.3
25	VEGFR2	2RL5	64.251	45.695	16.832	Bronyl acetate	MOL006862	‐6.2
26	PRKCA	4RA4	33.558	‐2.764	12.099	Bis[(2S)-2-ethylhexyl] benzene-1,2-dicarboxylate	MOL001490	‐6.1
27	PRKCA	4RA4	33.558	‐2.764	12.099	ZINC03860434	MOL001749	‐6
28	VEGFA	6Z13	31.85	18.649	‐10.039	Luteolin	MOL000006	‐5.7
29	VEGFA	6Z13	31.85	18.649	‐10.039	(2R)-5,7-dihydroxy-2-(4-hydroxyphenyl)chroman-4-one	MOL001040	‐5.7
30	MAPK1	5NHV	‐15.847	12.217	41.95	Asiatic acid	MOL006861	‐5.6
31	VEGFA	6Z13	31.85	18.649	‐10.039	Quercetin	MOL000098	‐5.6
32	VEGFA	6Z13	31.85	18.649	‐10.039	Ellagic acid	MOL001002	‐5.4
33	VEGFA	6Z13	31.85	18.649	‐10.039	Naringenin	MOL004328	‐5.1

Based on the binding patterns observed, it was found that kaempferol formed 3 hydrogen bonds with VEGFR2 through THR-916, GLU-917, and CYS-919. Additionally, 8 hydrophobic interactions were observed, including 2 π-sigma interactions (LEU840 and LEU1035) and 4 π-alkyl interactions (VAL848, ALA866, LYS868, and VAL899), which constituted the hydrophobic pocket contributing to the low binding energy of ‐9.1 kcal/mol. In the dissection of the binding pattern of ENOS-quercetin, 3 hydrogen bonds were observed interacting with TRP356, MET358, and GLU361, 2 π-stacking (TRP178 and PHE353), and 1 π-sulfur bond (GLY186) were formed with quercetin, which resulted in a binding energy of ‐9 kcal/mol. These amino acid residues of ENOS constituted a hydrophilic pocket that strongly interacted with the hydroxyl group of quercetin. In the complex of VEGFR2-(2R)-5,7-dihydroxy-2-(4-hydroxyphenyl) chroman-4-one, 3 hydrogen bonds were observed with CYS919 in VEGFR2 with the binding energy of ‐9 kcal/mol. And 3 hydrogen bonds were found between GLU228, ALA230, and ASP292 in AKT and ellagic acid with the binding energy of ‐8.7 kcal/mol. Based on the results described above, kaempferol, quercetin, (2R)-5,7-dihydroxy-2-(4-hydroxyphenyl) chroman-4-one and ellagic acid could constitute stable complexes with VEGFR2, ENOS, and AKT with low binding free energy and good binding pattern, which unveiled the potential compound-target pharmacological interaction (Table 3).

**Table 3:** The MM/PBSA binding free energy for complex during MD simulations.

Macromolecule	Hit ligand	Hydrogen bonds	Potential hydrophobic interactions	Binding free energy (mmPBSA model) (kcal·mol^-1^)
VEGFR2	Kaempferol	THR916, GLU917, CYS919	LEU840, VAL848, ALA866, LYS868, VAL899, PHE918, LEU1035, PHE1047	‐25.098
ENOS	Quercetin	TRP356, MET358, GLU361	TRP178, GLY186, LEU193, PHE353, SER354, GLY355, TYR357, VAL418, PHE473	‐20.867
VEGFR2	(2R)-5,7-dihydroxy-2-(4-hydroxyphenyl)chroman-4-one	CYS919	LEU840, VAL848, ALA866, LYS868, LEU889, VAL899, THR916, PHE918, LEU1035, PHE1047	‐25.7
AKT	Ellagic acid	GLU228, ALA230, ASP292	LEU156, GLY157, GLY159, VAL164, ALA177, MET227, TYR229, MET281, THR291, PHE438	‐5.436

MD = molecular dynamics.

**Figure 7.**
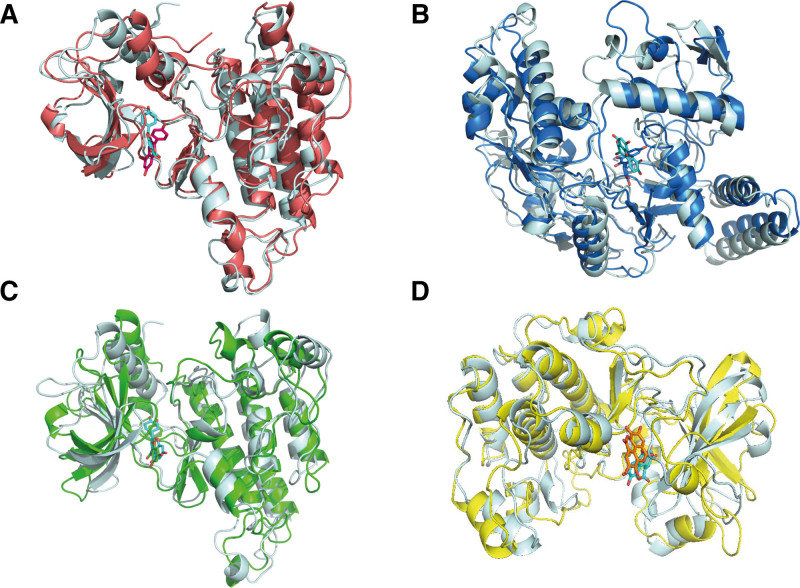
The comparisons of initial conformations and last frames in 50 ns MD simulations. (A) VEGFR2–kaempferol complex. The cyan sticks and cartoons represent kaempferol and VEGFR2 in initial conformation and the red represents last frame. (B) eNOS–quercetin complex. The cyan sticks and cartoons represent quercetin and eNOS in initial conformation and the blue represents last frame. (C) VEGFR2-(2R)-5,7-dihydroxy-2-(4-hydroxyphenyl) chroman-4-one complex. The cyan sticks and cartoons represent (2R)-5,7-dihydroxy-2-(4-hydroxyphenyl) chroman-4-one and VEGFR2 in initial conformation and the green represents last frame. (D) AKT–ellagic acid complex. The cyan sticks and cartoons represent ellagic acid and AKT in initial conformation and the yellow represents last frame.

**Figure 8.**
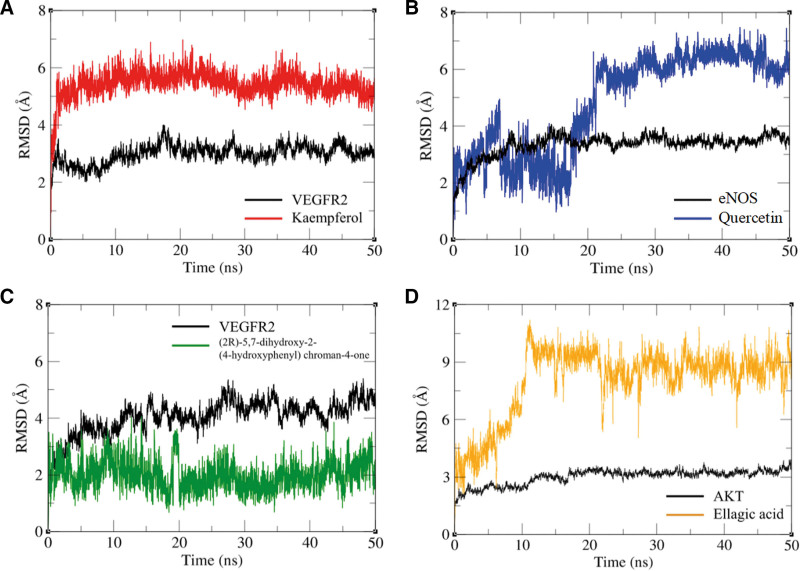
The RMSD plot of complex during 50 ns MD simulations trajectory. (A) VEGFR2-Kaempferol complex. The red line represents kaempferol and the black line represents VEGFR2. (B) eNOS–quercetin complex. The blue line represents quercetin and the black line represents eNOS. (C) VEGFR2-(2R)-5,7-dihydroxy-2-(4-hydroxyphenyl) chroman-4-one complex. The green line represents (2R)-5,7-dihydroxy-2-(4-hydroxyphenyl) chroman-4-one and the black line represents VEGFR2. (D) AKT-ellagic acid complex. The yellow line represents ellagic acid and the black line represents AKT. MD = molecular dynamics, RMSD = root mean square deviation.

**Figure 9.**
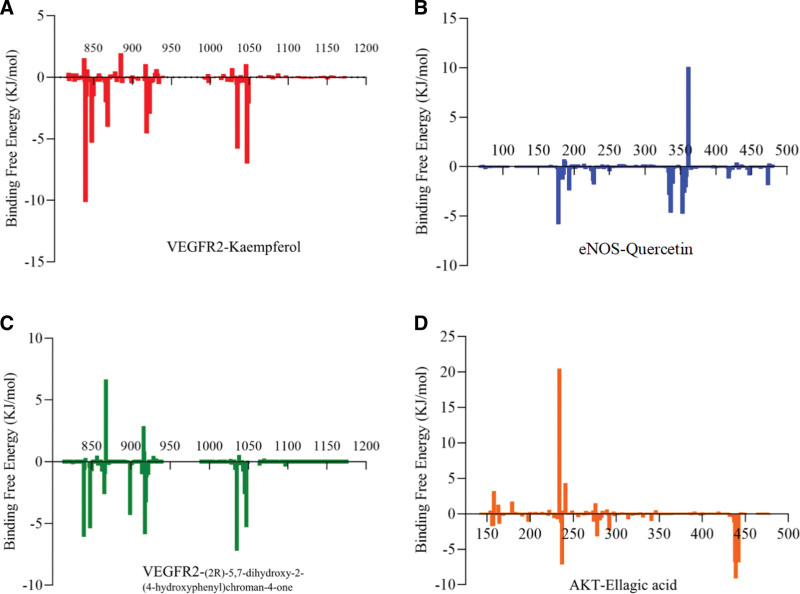
The graph of free energy contribution (MM/PBSA model) of amino acid residues to ligand–protein binding. (A) VEGFR2–kaempferol complex. (B) eNOS–quercetin complex. (C) VEGFR2-(2R)-5,7-dihydroxy-2-(4-hydroxyphenyl) chroman-4-one complex. (D) AKT–ellagic acid complex.

We further conducted the 50 ns MD simulations to investigate the possible binding modes and mechanism of actions between top 4 compound-target pairs. The comparisons between the initial conformations and the last frames of 50 ns MD simulations demonstrated that all compound ligands were stably complexed with their target receptors (Fig. 7). As shown in Figure 8 and Figure S1, Supplemental Digital Content, http://links.lww.com/MD/N736, the root mean square deviation (RMSD) and root mean square fluctuation (RMSF) of VEGFR2, ENOS, and AKT relative to the initial structure were calculated. The conformations of top 4 compound-target pairs both reached equilibrium after 20 ns, as evidenced by their low magnitude of RMSD, which were within the fluctuation range of 1.5 Å, strongly proved that the protein had achieved conformational stability with the ligand molecules. Elevated RMSF were observed in specific regions with high flexibility, such as residues with hydrogen and hydrophobic interactions. We also examined the possible hydrogen binding in the complex, and found that the number of hydrogen bonds changed similarly after 20 ns, which formed hydrogen bonds as indicated by the docking pattern in Figure S2, Supplemental Digital Content, http://links.lww.com/MD/N736 and Table S1, Supplemental Digital Content, http://links.lww.com/MD/N737. Our results revealed that CYS919 in VEGFR2 significantly contributed to binding with kaempferol and (2R)-5,7-dihydroxy-2-(4-hydroxyphenyl) chroman-4-one (Fig. 6A). GLU361 in ENOS played a significant role in hydrogen binding with quercetin. These are consistent with the analysis of hydrogen bond occupancy and could be the potential binding site for HF drug therapy (Fig. 6B). Furthermore, we calculated the total binding free energy of the top 4 compound-target pairs using MM/PBSA model, which were ‐25.098, ‐20.867, ‐25.7, and ‐5.436 kcal/mol, respectively, suggesting the strong interactions between protein ligands and high stability of the complexes formed (Fig. 9, Table S1, Supplemental Digital Content, http://links.lww.com/MD/N737, and Table 3).

### 2.6. Protective effects of GXST on H_2_O_2_-induced apoptosis in vitro

**Figure 10.**
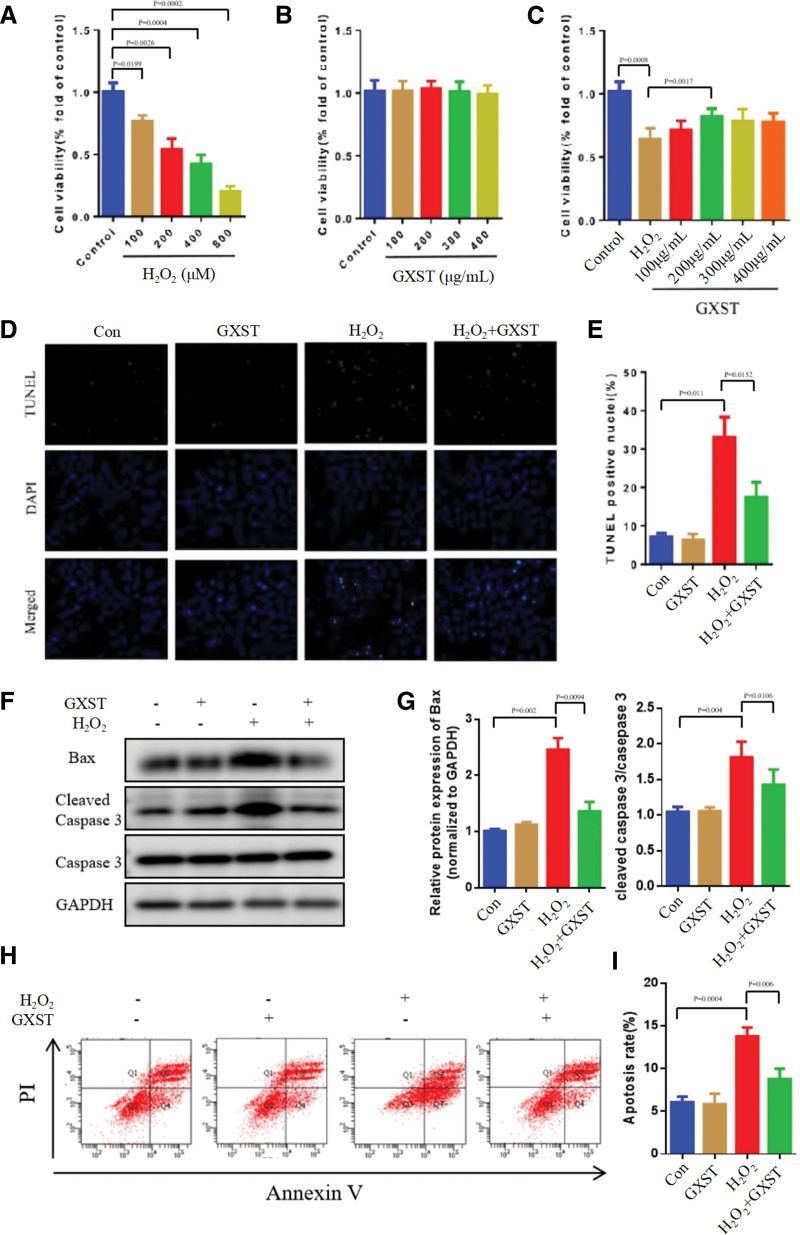
Protective effects of GXST on H_2_O_2_-induced apoptosis in vitro. (A) Cell viability of HUVECs stimulated with different concentrations of H_2_O_2_ (100, 200, 400, and 800 μM) for 24h, n = 5. (B) Cell viability of HUVECs induced with various concentrations of GXST (100, 200, 300, and 400 μg/mL) for 24 hours, n = 5. (C) Cell viability of H_2_O_2_-induced injury following treatment with GXST extract at different concentrations (100, 200, 300, 400 μg/mL), n = 5. (D) Representative images of TUNEL staining in different groups, scale bar: 20 μm. (E) Quantitative analysis of TUNEL-positive, n = 3. (F–G) Representative western blotting pictures and quantitative analysis of BAX, cleaved caspase 3 and caspase 3, n = 3. (H–I) HUVECs were treated with H_2_O_2_ (200 µM) and GXST(200 μg/mL) followed by annexin V/PI double staining and analyzed using flow cytometry. n = 3. GXST = Guanxin Shutong capsule, HUVECs = human umbilical vein endothelial cells, PI = propidium iodide, TUNEL = terminal deoxynucleotidyl transferase dUTP nick-end labeling.

To further investigate the key pharmacological mechanism of GXST in treating HF, as predicted by network pharmacology analysis, we conducted in vitro experiments to elucidate its effects. Endothelial damage caused by H_2_O_2_ is a significant contributor to HF.^[18]^ To study the impact of H_2_O_2_-induced endothelial cell damage, we utilized the human umbilical vein endothelial cells (HUVECs). The cells were exposed to varying concentrations of H_2_O_2_ to induce damage. Cell viability was assessed using the CCK8 method. The results demonstrated that after 24 hours of treatment with 400 μM H_2_O_2_, cell viability decreased by 50% (*P* = .0004) (Fig. 10A). To explore the potential protective effect of GXST on endothelial cell injury, HUVECs were incubated with different concentrations of GXST. The findings indicated that GXST at concentrations ranging from 100 to 400 μg/mL did not negatively affect HUVECs viability (Fig. 10B). However, at a concentration of 200 μg/mL, GXST exhibited a significant protective effect against H_2_O_2_-induced damage in HUVECs (*P* = .0017) (Fig. 10C). Hence, we selected a concentration of 200 μg/mL for further analysis. To examine the potential impact of GXST on endothelial cell apoptosis, we employed the terminal deoxynucleotidyl transferase dUTP nick-end labeling (TUNEL) method. Our results revealed a significantly higher number of apoptotic HUVECs in the H_2_O_2_ group compared to the control group (*P* = .0011). Interestingly, treatment with GXST resulted in a significant reduction in the number of apoptotic HUVECs (*P* = .0152) compared to the H_2_O_2_ group (Fig. 10D and E). Furthermore, western blotting was conducted to assess the expression of apoptosis-related proteins in HUVECs. We observed a substantial increase in the expression of the pro-apoptotic protein Bax and cleaved caspase-3/caspase-3 in the H_2_O_2_ group compared to the control group. However, treatment with GXST under H_2_O_2_ conditions led to a notable reduction in Bax expression levels (*P* = .0094) and a significant inhibition of caspase-3 activation (*P* = .0106) (Fig. 10F and G). Moreover, flow cytometry was employed to evaluate the impact of GXST on apoptosis. Our analysis demonstrated that GXST effectively reduced H_2_O_2_-induced apoptosis in HUVECs (*P* = .0004) (Fig. 10H and I). Our in vitro experiments provide further evidence supporting the pharmacological mechanism of GXST in the treatment of HF. GXST demonstrated protective effects against H_2_O_2_-induced endothelial cell damage, leading to a reduction in cell apoptosis. These findings suggest that GXST may play a crucial role in preserving endothelial cell function and combating HF pathogenesis.

To investigate the protective effect of GXST on HF more comprehensively, we also conducted experiments by incubating H9C2 cells with various concentrations of H_2_O_2_ to induce cellular injury. Similarly, the results demonstrated that after 24 hours of treatment with 200 μM H_2_O_2_, cell viability decreased by 50% (*P* < .0001) (Fig. S3A, Supplemental Digital Content, http://links.lww.com/MD/N736). Furthermore, we observed that different concentrations of GXST had no detrimental impact on the viability of H9C2 cells (Fig. S3B, Supplemental Digital Content, http://links.lww.com/MD/N736). Moreover, our findings revealed that when H9C2 cells were exposed to 200 μg/mL GXST, it exhibited a significant protective effect against H_2_O_2_-induced damage (*P* = .0004) (Fig. S3C, Supplemental Digital Content, http://links.lww.com/MD/N736). These results indicate that GXST has the potential to mitigate the injurious effects of H_2_O_2_ on H9C2 cells. Moreover, western blotting was conducted to further assess the expression of apoptosis-related proteins in H9C2 cells. We observed a substantial increase in the expression of the pro-apoptotic protein Bax and cleaved caspase-3/caspase-3 in the H_2_O_2_ group compared to the control group. However, following treatment with GXST under H_2_O_2_ conditions, there was a notable reduction in Bax expression levels (*P* = .0005) and a significant inhibition of caspase-3 activation (*P* = .0024) (Figure S3D and E, Supplemental Digital Content, http://links.lww.com/MD/N736). These results highlight the ability of GXST to attenuate apoptotic pathways triggered by H_2_O_2_ in myocardial cells. Overall, these findings suggest that GXST enhances cell viability and mitigates H_2_O_2_-induced damage to H9C2 cells, while also reducing apoptosis.

### 2.7. GXST activated the VEGFR2/AKT/eNOS signaling pathways

**Figure 11.**
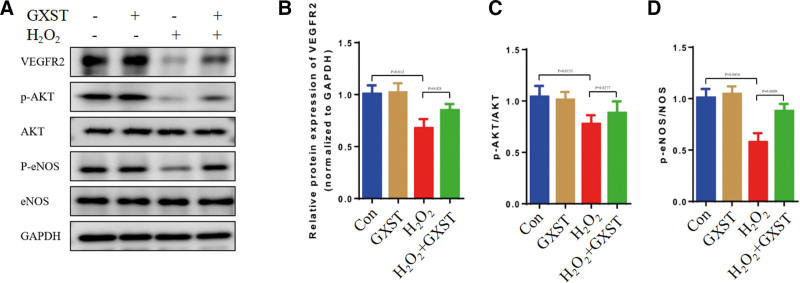
GXST activated the VEGFR2/AKT/eNOS signaling pathwaysin in the HUVECs. (A–D) Representative western blotting pictures and quantitative analysis of VEGFR2, p-Akt/AKT, and p-eNOS/eNOS, n = 3. HUVECs = human umbilical vein endothelial cells.

Through network pharmacology analysis, we identified VEGFR2, AKT, and eNOS as crucial proteins in the PPI network, potentially playing a pivotal role in GXST’s treatment of HF. The VEGFR2/AKT/eNOS pathway is extensively studied and closely associated with angiogenesis, as well as the occurrence and progression of cardiovascular diseases.^[19]^ Our findings demonstrated that the administration of GXST resulted in increased expression of VEGFR2 (*P* = .028). Additionally, there was a significant increase in the ratio of p-AKT/AKT (*P* = .027) and p-eNOS/eNOS (*P* = .0088) in HUVECs (Fig. 11). These results suggest that GXST may modulate the VEGFR2/AKT/eNOS pathway, which is known to be involved in angiogenesis and the pathophysiology of cardiovascular diseases. The upregulation of VEGFR2 expression and the activation of AKT and eNOS indicate potential mechanisms through which GXST exerts its therapeutic effects in HF. These findings provide valuable insights into the pharmacological actions of GXST and support its potential as a treatment for HF by targeting the VEGFR2/AKT/eNOS pathway.

## 3. Discussion

HF is a common cardiovascular disease with a high mortality rate globally.^[20]^ GXST is a widely used Chinese herbal medicine for treating cardiovascular diseases.^[21]^ Despite its widespread use, the underlying mechanism of how GXST treats HF remains unclear. Therefore, this study aims to investigate the potential protective effect of GXST on HF and its molecular mechanisms by using an integrated approach that includes network pharmacology, molecular docking, MD simulations, and experimental validation.

Network pharmacology analysis revealed that GXST mainly regulates HF through its active components, including quercetin, kaempferol, beta-sitosterol, naringenin, and ZINC03860434, as well as other ingredients. These components have been reported to possess various pharmacological activities, including anti-inflammatory, antioxidant, antiapoptotic, and cardioprotective effects.^[22–25]^ Notably, quercetin and kaempferol have been shown to improve cardiac function in animal models of HF and to have antiapoptotic effects in cardiac fibroblasts and cardiomyocytes, respectively.^[26,27]^ Beta-sitosterol has also been reported to reduce cardiac injury and improve functional recovery in a rat model of HF.^[28]^ Naringenin, on the other hand, exerts antioxidant and anti-inflammatory effects and has been shown to improve cardiac function in animal models of HF.^[29]^ Additionally, it has demonstrated antiapoptotic effects in ischemia–reperfusion injury in the heart.^[30]^ These active ingredients target several pathways, including the PI3K-Akt, MAPK, and NF-κB signaling pathways, which are known to be involved in the development and progression of HF.^[31–33]^ Based on the selected GXST active ingredients, we also predicted the possible targets of these active ingredients. Among them, PTGS2, SCN5A, PTGS1, ADRB2, and ACHE were the top 5 degrees of potential drug-disease target genes. Notably, previous studies have shown that these genes contribute to the development and progression of HF.^[34–38]^

The enrichment analysis for GO function and KEGG pathway of intersection genes provides deeper insights into the potential signaling pathways or BPs regulated by GXST in HF. The results showed that BPs related to response to drug, response to xenobiotic stimulus, aging, negative regulation of apoptotic process, and response to hypoxia were the most significant enriched categories. These findings suggest that GXST might exert its therapeutic effects in HF through its ability to modulate these BPs. In addition, the KEGG pathway analysis revealed that PI3K-Akt signaling pathway, apoptosis, TNF signaling pathway, endocrine resistance, and HIF-1 signaling pathway were mostly enriched. These pathways are all known to be involved in the regulation of cardiac function, and their modulation by GXST may contribute to its therapeutic effects in HF.^[39]^ Liu et al found that GXST treated pathologic heart hypertrophy through the PI3K-Akt signaling pathway.^[40]^ Our study further expands on these findings by pinpointing specific molecular interactions and pathways regulated by GXST, thereby elucidating the role of GXST in HF therapy.

Insufficient generation of myocardial capillaries during the occurrence and development of HF can promote the transition from compensatory hypertrophy to decompensated hypertrophy, ultimately leading to HF. This process is closely regulated by endothelial cells and angiogenesis.^[18]^ To investigate the effects of GXST on HF in vitro, a H_2_O_2_-induced HUVECs model was established in this study. Upregulation of Bax protein expression and caspase3 activation are widely recognized as key indicators of apoptosis.^[41]^ We found that GXST effectively enhanced the cell viability of HUVECs stimulated by H_2_O_2_ and reduced apoptosis. To comprehensively investigate the protective effect of GXST on HF, we also conducted similar experiments using H9C2 cells, a well-established myocardial cell line. Consistent with our observations in HUVECs, treatment with GXST significantly attenuated H_2_O_2_-induced cellular injury in H9C2 cells, as evidenced by enhanced cell viability. Our western blot analysis in H9C2 cells further supported the antiapoptotic effects of GXST. This indicates that GXST may inhibit apoptotic pathways triggered by H_2_O_2_ in myocardial cells. Prior studies have demonstrated that GXST exerts a protective effect against myocardial infarction through its antiapoptotic properties.^[42]^ Our study extends these findings by suggesting that GXST may safeguard against cardiac injury by inhibiting apoptosis in both endothelial cells and cardiomyocytes, thereby preserving their functionality within the heart.

Importantly, our molecular docking outcomes revealed that the identified ingredients, namely kaempferol, quercetin, (2R)-5,7-dihydroxy-2-(4-hydroxyphenyl) chroman-4-one, and ellagic acid, formed stable complexes with their respective core proteins: VEGFR2, eNOS, and AKT. Notably, previous studies have suggested that ellagic acid also may be one of the significant antioxidants in GXST.^[43]^ The MD simulations allowed us to investigate the binding modes and mechanisms of action of these top 4 compound-target pairs, which were identified earlier. These pairs demonstrated conformational stability and strong protein–ligand interactions. These findings present intriguing targets for further research for HF.

VEGFR2, AKT, and eNOS, are widely present in endothelial cells and play a crucial role in vascular growth and tissue repair.^[44]^ Prior studies have implicated VEGFR2/AKT/eNOS signaling pathways in the pathophysiology of HF and plays a crucial role in angiogenesis, vascular homeostasis, and cardiovascular health.^[19,45]^ Activation of VEGFR2 leads to downstream activation of AKT, which subsequently promotes the phosphorylation of eNOS and the generation of nitric oxide, ultimately resulting in vasodilation and improved cardiac function.^[46]^ Hence, we further explored the effect of GXST on the VEGFR2/AKT/eNOS signaling pathway under HF conditions in vitro. Our results has found that GXST increased the expression of VEGFR2 and the phosphorylation levels of AKT and eNOS. VEGFR2 is a receptor protein that can bind to VEGF and promote angiogenesis and repair,^[47]^ the expression of VEGFR2 in vascular endothelial cells and ventricular tissue is affected by HF.^[48]^ Interestingly, previous studies have shown that GXST significantly upregulates VEGFA.^[49]^ Hence, these findings suggest that GXST may enhance angiogenesis and vascular repair through the upregulation of both VEGFR2 and VEGFA. This dual effect on the VEGF signaling pathway could potentially have important implications for therapeutic interventions aimed at promoting tissue revascularization and healing. AKT is involved in signal transduction and the regulation of many cellular processes, and in endothelial cells, it can promote cell survival and proliferation while reducing endothelial cell apoptosis.^[50]^ eNOS can catalyze the synthesis of NO, which directly affects the maintenance of normal cardiovascular function, promotes vascular dilation, and reduces endothelial cell apoptosis.^[51]^ Our finding is consistent with previous research where GXST was found to increase the expression of p-AKT and p-eNOS in the hearts of mice with pathological cardiac hypertrophy.^[40]^ Our study expands the potential and scope of application of GXST in regulating AKT and eNOS signaling. These findings provide a foundation for further investigation into the therapeutic potential of GXST in cardiovascular diseases, particularly those involving impaired AKT and eNOS signaling. Overall, our study suggest that GXST may be a potential therapeutic candidate for the treatment of HF by regulating the VEGFR2/AKT/eNOS signaling pathway and promoting angiogenesis.

This study upon verification of the results of network pharmacology, it was discovered that GXST contains a complex polypeptide structure, and exhibits a strong binding ability to VEGFR2, AKT, and eNOS at multiple sites. In vitro experiments showed that GXST was effective in rescuing H_2_O_2_-induced cell death. The mechanism behind the cell-protective effect of GXST may be linked to the protein expressions of VEGFR2, AKT, and eNOS. However, this study has several limitations that need to be acknowledged. Firstly, it is important to note that the findings of this study rely solely on in silico and in vitro investigations. While these methods provide valuable insights into the potential effects of GXST, they do not directly replicate the complex interactions that occur within a living organism. Consequently, the outcomes of these experiments may not accurately represent the safety and effectiveness of GXST. Future research should incorporate in vivo studies involving animal models. Secondly, our study did not explore any possible interactions or synergistic effects of GXST with other herbal remedies or conventional heart failure treatments. Many patients with heart failure often receive multiple treatments, understanding the potential interactions between GXST and these treatments is essential for ensuring the safety and efficacy of GXST when used in combination with other interventions. Thirdly, the evaluation of GXST’s clinical application is limited by the lack of investigation into its long-term effects and potential side events. Gaining an understanding of the long-term effects of GXST is paramount in assessing its overall effectiveness and durability as a treatment option for heart failure. Additionally, monitoring for potential side events is necessary to ensure patient safety. Future research should prioritize conducting comprehensive clinical trials that involve long-term follow-up of patients receiving GXST. Fourthly, an important stage in the drug development process involves translating the findings from in vitro and in silico research into in vivo and clinical settings. However, our study did not address any potential obstacles or constraints that might arise during this transition.

## 4. Conclusions

By integrating network pharmacology and experimental verification, our study provides valuable insights into the pharmacological mechanisms of GXST in treating HF. These findings contribute to a better understanding of TCM’s role in cardiovascular diseases and may guide the development of novel therapeutic approaches for HF.

## 5. Materials and methods

### 5.1. Screening of active ingredients and gene targets

The TCMSP platform (https://test.tcmsp-e.com/) and SymMap database (http://www.symmap.org/) were used to search for the drug ingredients of GXST. According to the absorption of exogenous chemicals by the pharmacokinetic body, ingredients with oral availability ≥ 30% and drug-likeness ≥ 0.18 were screened out. PubChem databases (https://pubchem.ncbi.nlm.nih.gov) were used to obtain the SMILES (Simplified Molecular Input Line Entry System) of the ingredients and then imported into the SwissTargetPrediction database (http://www.swisstargetprediction.ch), Pharmmapper (https://www.lilab-ecust.cn/pharmmapper/), TCMSP platform and SymMap databases to predict the targets. The Excel data of the ingredients and target genes were matched and merged with the jvenn tool (http://www.bioinformatics.com.cn/static/others/jvenn).

Effective Targets for HF-related genes from 5 databases GeneCards (https://www.genecards.org/), PharmGKB (https://www.pharmgkb.org/), OMIM (https://mirror.omim.org/), DisGeNet (https://www.disgenet.org/), and TTD (https://db.idrblab.net/ttd/) database were collected based on the keyword of HF.

### 5.2. Network construction of active ingredients-disease-targets

GXST predicted targets were compared with the HF-related targets, and the common targets were screened using the jvenn tool and common targets were collected for network construction. Cytoscape 3.10.2 software was used to build and analyze the Herbs-Ingredients-Target genes network and the Herbs-Ingredients-Targets-Disease network. The nodes and edges represented the ingredients or targets and the relationship between them, respectively.

### 5.3. Functional annotation of core targets

Functional annotation analyses (Gene Ontology and KEGG pathways) were performed using DAVID (https://david.ncifcrf.gov/) using Homo sapiens genes as background. GO functional enrichment classified genes into 3 categories, including CC, MF, and BP. Terms with Benjamin–Hochberg corrected *P*-values < .05 were determined to be enriched. The STRING database (https://cn.string-db.org/) was used to analyze the PPI networks of the intersection genes. The results were visualized using Cytoscape 3.10.2 software as well. CytoNCA tool of Cytoscape software were used to calculate network centralities based on the BC, CC, and degree, with median values of these parameters as threshold criteria. Subsequently, the core targets network was constructed and narrowed down by UpSet plot analysis.

### 5.4. Molecular docking and MD simulations

The crystal structures of VEGFR2 (2RL5), eNOS (6NH7), AKT (6NPZ), MAPK1 (5NHV), PRKCA (4RA4), IKBKB (4KIK), IL6 (1ALU), MAPK3 (6GES), RAF1 (6XGV), and VEGFA (6Z13) were retrieved from the RCSB PDB database (https://www.rcsb.org/). The 2D structured sdf files of the active ingredients were collected from the PubChem database (https://pubchem.ncbi.nlm.nih.gov/) and converted to pdbqt files by Raccoon2 of AutoDockTools. AutoDock Vina 1.2.5 software were utilized in all the docking experiments with the optimized model of 100 binding modes as the docking target. The 2D protein–ligand interaction profiles was generated using LigPlot+ and VMD educational version. MD simulations were performed with Gromacs 2024.3 package, using the AMBERFF99SB force field. The box dimensions ensured that any protein atom was at least 1 nm away from the wall of the box with periodic boundary conditions and solvated by simple point charge water molecules. NaCl counter ions were added to satisfy the electro-neutrality condition. Energy minimization was carried out using the steepest descent method. Finally, 50 ns MD simulations were carried out for all complexes. Structural analysis was evaluated by RMSD and RMSF. The MM–PBSA calculation was performed using the gmx_mmpbsa of GROMACS. All molecular graphics were displayed and prepared using the PyMOL educational version.

### 5.5. Cell culture

The HUVECs and H9C2 cells (Cell Bank, Chinese Academy of Sciences, Shanghai, China) were cultured in Endothelial Cell Medium (ECM, ScienCell, Carlsbad, CA) supplemented with 10% fetal bovine serum, 1% endothelial cell growth supplement and 1% penicillin/streptomycin solution (P/S). Cells were maintained in a humidified atmosphere with 5 % CO_2_ at 37 °C. The cells were maintained in a specific medium, with the frequency of medium changes being performed every 24 hours, and the cells were used at passage 3 to 8. The passage number of the cells used for analysis was carefully recorded. When the cells were stimulated with H_2_O_2_ (Sigma, Cat#: 88597-100ML-F) or GXST (Buchang Pharmaceutical Co., Ltd, Xi’an, China), they were replaced with RPMI1640 medium with 10% fetal bovine serum and 1% P/S.

### 5.6. Cell proliferation assays

Cells were seeded in 96-well plates at a density of 1 × 10^4^ cells/well and incubated for 24 hours. Following this, the cells were incubated for an additional 24 hours with varying concentrations of H_2_O_2_ or GXST. The cell proliferation rate was determined using a standard CCK-8 assay. After the incubation period, the absorbance of the samples was measured at a wavelength of 450 nm using a microplate reader (BioTek). This experiment was independently performed 3 times to ensure the reliability of the results. To ensure precision and reliability, each experimental group was evaluated in 5 duplicate wells, and the experiments were repeated 3 times.

### 5.7. TUNEL assays

For TUNEL assays, DNA damage analysis was performed using a One Step TUNEL Apoptosis Assay Kit (Cat#: C1088; Beyotime). HUVECs, both normal and those treated with H_2_O_2_ or GXST, had their medium discarded, were washed with phosphate-buffered saline (PBS), and fixed with 4% paraformaldehyde for 30 minutes. After fixation, the cells were washed, and the TUNEL assay solution was prepared and dropped onto the cells. The cells were then incubated in the dark at 37 °C for 1 hour. Following staining, the cells were washed 3 times with PBS. The slides were sealed with anti-fluorescence quenching solution and observed under a fluorescence microscope. The excitation wavelength range was 450 to 500 nm, and the emission wavelength range was 515 to 565 nm, resulting in green fluorescence. To ensure accuracy and consistency, each experimental group was assessed in triplicate wells, and the experiments were replicated 3 times, thereby ensuring precision and reliability of the results.

### 5.8. Flow cytometry

To detect apoptosis in HUVECs, the Annexin V-fluorescein isothiocyanate/propidium iodide apoptosis detection kit (BD Pharmingen, San Diego, CA, Cat#: 556547) was employed. HUVECs were initially seeded in a 6-well plate at a density of 5 × 10^5^ cells/well and subsequently treated with 200 μM H_2_O_2_ and 200 μg/mL GXST for a period of 24 hours. After treatment, the cells were collected, washed twice with cold PBS, and then resuspended in binding buffer at a concentration of 1 × 10^6^ cells/mL. Subsequently, the cells were incubated with 5 μL annexin V-fluorescein isothiocyanate and 5 μL propidium iodide at room temperature in the dark for 15 minutes. Following the incubation, the cells were washed with binding buffer to remove any excess staining reagents. Finally, the apoptosis rates of HUVECs were determined using a flow cytometer (BD Biosciences, San Jose, CA), which allowed for accurate measurement and analysis. In order to ensure the accuracy and reliability of the results, the experiments were replicated 3 times.

### 5.9. Western blot analysis

We extracted total protein from HUVECs and H9C2 cells using RIPA lysis buffer (Thermo Fisher Scientific, China, Cat#: 89901) and determined the protein concentrations using a BCA assay kit (Thermo Fisher Scientific, China, Cat# 23225). To analyze the protein expression levels, we performed Western blotting following a previously described method.^[52]^ To begin, equal amounts (30 µg) of protein samples were separated by SDS-PAGE using 10% and 15% sodium dodecyl sulfate-polyacrylamide gels. The separated proteins were then transferred onto polyvinylidene difluoride membranes (Millipore, Bedford, MA). To ensure proper binding and minimize nonspecific interactions, the membranes were blocked with 5% skimmed milk for 1 hour at room temperature. Next, the membranes were incubated overnight at 4 °C with the respective primary antibodies. We used the following primary antibodies: anti-BAX antibody (#2772, 1:1000, Cell Signaling Technology, Danvers, MA), anti-cleaved caspase3 antibody (#9661, 1:1000, Cell Signaling Technology, Danvers, MA), anti-caspase3 antibody (#9662, 1:1000, Cell Signaling Technology, Danvers, MA), anti-VEGFR2 antibody (#AF6281, 1:1000, Affinity, Cincinnati, OH), anti-p-AKT (T308) antibody (#ab38449, 1:1000, Abcam, Cambridge, UK), anti-AKT antibody (#ab179463, 1:1000, Abcam, Cambridge, UK), anti-GAPDH antibody (#2118, 1:1000, Cell Signaling Technology, Danvers, MA), anti-p-eNOS (S1177) antibody (#ab215717, 1:1000, Abcam, Cambridge, UK), and anti-eNOS antibody (#ab252439, 1:1000, Abcam, Cambridge, UK). Following the primary antibody incubation, the membranes were incubated with HRP-coupled goat anti-rabbit secondary antibody (#ab6721, 1:5000, Abcam, Cambridge, UK) for 2 hours. Finally, the protein signals on the membranes were detected using the ECL Plus assay kit (Cat#: RPN2232, Amersham Bioscience, Buckinghamshire, UK).

### 5.10. Statistical analyses

All experimental data were analyzed by GraphPad Prism 8. Continuous variables are expressed as mean ± SD (standard deviation) of independent experiments. Statistical differences between 2 groups were calculated by the unpaired Student *t* test, and the differences between more than 2 groups were tested by ANOVA which followed the Bonferroni test for sub-two groups comparison. All statistical analyses and visualization plots were executed using R. All the differences result in a *P*-value < .05 were considered significant.

## Acknowledgments

All authors read and approved the final manuscript. We are grateful to all the researchers involved in this study.

## Author contributions

**Conceptualization:** Zheming Yang, Xiaolin Zhang.

**Data curation:** Zheming Yang, Zhu Mei.

**Formal analysis:** Zheming Yang, Haixu Song.

**Investigation:** Yaling Han, Yu Xue.

**Methodology:** Yaling Han, Jiayin Li, Chenghui Yan, Xiaolin Zhang.

**Software:** Jiayin Li, Shuli Zhang.

**Supervision:** Jiayin Li.

**Validation:** Zheming Yang, Haixu Song, Chenghui Yan.

**Visualization:** Hanlin Wu.

**Writing – original draft:** Zheming Yang, Jiayin Li.

**Writing – review & editing:** Yaling Han, Chenghui Yan, Xiaolin Zhang.

## Supplementary Material


