# Supplementary material for: Integrating network pharmacology and experimental verification to explore the pharmacological mechanisms of Guanxin Shutong capsule in treating heart failure
Source: Medicine (Baltimore). 2024 Oct 18;103(42):e40118. doi: 10.1097/MD.0000000000040118 (PMC11495747; doi:10.1097/MD.0000000000040118)
Supplement: Supplementary file 1 [file medi-103-e40118-s001.docx]

| Table S1. Detailed list of active ingredients from GXST herbs. | | | | | | | | | | | | | | | |
| --- | --- | --- | --- | --- | --- | --- | --- | --- | --- | --- | --- | --- | --- | --- | --- |
| DL≥0.18 OB≥30% |  |  |  |  |  |  |  |  |  |  |  |  |  |  |  |
| Guangzao |  |  |  |  |  |  |  |  |  |  |  |  |  |  |  |
| molecule_ID | MOL_ID | file_ID | MOL_ID | molecule_name | ob% | mw | alogp | caco2 | bbb | halflife | hdon | hacc | dl | FASA |  |
| 358 | MOL000358 | fyp_hjs_Molecule461_qt.mol2 | MOL000358 | beta-sitosterol | 36.9139058327 | 414.790 | 8.084 | 1.32463 | 0.98588 | 5.355491 | 1 | 1 | 0.75123 | 0.22550575 |  |
| 1002 | MOL001002 | hc_my_Molecule142.mol2 | MOL001002 | ellagic acid | 43.0645585827 | 302.200 | 1.483 | -0.44261 | -1.41189 | -1.040645 | 4 | 8 | 0.43417 | 0.43301627 |  |
| 1490 | MOL001490 | hcx_ndl_Molecule46.mol2 | MOL001490 | bis[(2S)-2-ethylhexyl] benzene-1,2-dicarboxylate | 43.5933254731 | 390.620 | 7.574 | 0.97947 | 0.67555 | 3.02355 | 0 | 4 | 0.34531 | 0.27978593 |  |
| 98 | MOL000098 | fyp_cg_Molecule36_qt.mol2 | MOL000098 | quercetin | 46.4333481195 | 302.250 | 1.504 | 0.04842 | -0.76890 | 14.400548 | 5 | 7 | 0.27525 | 0.38453352 |  |
| 1736 | MOL001736 | lbh_blg_Molecule113.mol2 | MOL001736 | (-)-taxifolin | 60.5062169158 | 304.270 | 1.485 | -0.24278 | -1.01861 | 14.37314 | 5 | 7 | 0.27342 | 0.40842575 |  |
| 96 | MOL000096 | fyp_cg_Molecule35.mol2 | MOL000096 | (-)-catechin | 49.6763868 | 290.290 | 1.920 | -0.02751 | -0.78354 | 0.383809 | 5 | 6 | 0.24162 | 0.34617227 |  |
| 422 | MOL000422 | fyp_hq_Molecule260.mol2 | MOL000422 | kaempferol | 41.8822495352 | 286.250 | 1.771 | 0.26096 | -0.55335 | 14.743371 | 4 | 6 | 0.24066 | 0 |  |
| 1040 | MOL001040 | hc_my_Molecule190.mol2 | MOL001040 | (2R)-5,7-dihydroxy-2-(4-hydroxyphenyl)chroman-4-one | 42.3633211422 | 272.270 | 2.298 | 0.37818 | -0.47578 | 16.830309 | 3 | 5 | 0.21141 | 0.40827236 |  |
| 4328 | MOL004328 | lbh_yj_Molecule42.mol2 | MOL004328 | naringenin | 59.2938977347 | 272.270 | 2.298 | 0.28421 | -0.37053 | 16.976509 | 3 | 5 | 0.21128 | 0.39587346 |  |
| Danshen |  |  |  |  |  |  |  |  |  |  |  |  |  |  |  |
| molecule_ID | MOL_ID | file_ID | MOL_ID | molecule_name | ob | mw | alogp | caco2 | bbb | halflife | hdon | hacc | dl | FASA |  |
| 7115 | MOL007115 | lxx_ds_Molecule1465.mol2 | MOL007115 | manool | 45.0443163606 | 304.570 | 5.497 | 1.28258 | 1.15512 | 5.812634 | 1 | 1 | 0.20208 | 0.27704036 |  |
| 7077 | MOL007077 | lxx_ds_Molecule1400.mol2 | MOL007077 | sclareol | 43.6706845842 | 308.560 | 4.270 | 0.83900 | 0.50611 | 4.707122 | 2 | 2 | 0.2058 | 0.27364558 |  |
| 1942 | MOL001942 | lbh_bss_Molecule14.mol2 | MOL001942 | isoimperatorin | 45.4642467387 | 270.300 | 3.653 | 0.97234 | 0.66379 | -1.441377 | 0 | 4 | 0.22524 | 0.27245414 |  |
| 7049 | MOL007049 | lxx_ds_Molecule1349.mol2 | MOL007049 | 4-methylenemiltirone | 34.348675885 | 266.360 | 4.332 | 1.25003 | 0.87334 | 14.595298 | 0 | 2 | 0.22726 | 0.38099211 |  |
| 7143 | MOL007143 | lxx_ds_Molecule1504.mol2 | MOL007143 | salvilenone Ⅰ | 32.4347085631 | 270.400 | 2.882 | 1.13263 | 0.76762 | 1.003896 | 1 | 2 | 0.22895 | 0.29523462 |  |
| 7041 | MOL007041 | lxx_ds_Molecule1339.mol2 | MOL007041 | 2-isopropyl-8-methylphenanthrene-3,4-dione | 40.8601540814 | 264.340 | 4.157 | 1.23498 | 0.80713 | 14.889806 | 0 | 2 | 0.22897 | 0.42923275 |  |
| 7124 | MOL007124 | lxx_ds_Molecule1475.mol2 | MOL007124 | neocryptotanshinone ii | 39.4629911416 | 270.350 | 3.612 | 0.75868 | 0.16289 | 26.984845 | 1 | 3 | 0.23157 | 0.32181671 |  |
| 7123 | MOL007123 | lxx_ds_Molecule1474.mol2 | MOL007123 | miltirone Ⅱ | 44.9510664818 | 272.320 | 0.773 | 0.04323 | -0.24992 | 2.24427 | 1 | 4 | 0.23537 | 0.34842026 |  |
| 7145 | MOL007145 | lxx_ds_Molecule1506.mol2 | MOL007145 | salviolone | 31.7241503895 | 268.380 | 4.045 | 1.04459 | 0.71906 | 0.332873 | 1 | 2 | 0.23568 | 0.36115721 |  |
| 6 | MOL000006 | fyp_bdh_Molecule4.mol2 | MOL000006 | luteolin | 36.1626293429 | 286.250 | 2.067 | 0.18500 | -0.84349 | 15.944492 | 4 | 6 | 0.24552 | 0.39326519 |  |
| 7107 | MOL007107 | lxx_ds_Molecule1448.mol2 | MOL007107 | C09092 | 36.069489858 | 286.500 | 5.979 | 1.62786 | 1.53758 | -0.156429 | 1 | 1 | 0.2474 | 0.25135443 |  |
| 7122 | MOL007122 | lxx_ds_Molecule1473.mol2 | MOL007122 | Miltirone | 38.7569863502 | 282.410 | 4.734 | 1.22696 | 0.86706 | 14.824597 | 0 | 2 | 0.25418 | 0.32491559 |  |
| 569 | MOL000569 | fyp_lxc_Molecule49_qt.mol2 | MOL000569 | digallate | 61.8486180263 | 322.240 | 1.529 | -0.76218 | -1.51806 | 5.293312 | 6 | 9 | 0.25635 | 0.42796314 |  |
| 7140 | MOL007140 | lxx_ds_Molecule1501.mol2 | MOL007140 | (Z)-3-[2-[(E)-2-(3,4-dihydroxyphenyl)vinyl]-3,4-dihydroxy-phenyl]acrylic acid | 88.5360210103 | 314.310 | 2.819 | -0.08596 | -0.76746 | 4.311427 | 5 | 6 | 0.25869 | 0.42778867 |  |
| 7149 | MOL007149 | lxx_ds_Molecule1513.mol2 | MOL007149 | NSC 122421 | 34.4929230859 | 300.480 | 4.987 | 1.07567 | 0.63293 | 14.563527 | 1 | 2 | 0.27645 | 0.29196444 |  |
| 2222 | MOL002222 | lbh_dgp_Molecule23.mol2 | MOL002222 | sugiol | 36.113534855 | 300.480 | 4.987 | 1.14054 | 0.69922 | 14.619943 | 1 | 2 | 0.27648 | 0.2726382 |  |
| 7118 | MOL007118 | lxx_ds_Molecule1468.mol2 | MOL007118 | microstegiol | 39.6122945749 | 298.460 | 4.745 | 1.05371 | 0.99257 | 4.515531 | 1 | 2 | 0.27734 | 0.32944259 |  |
| 7098 | MOL007098 | lxx_ds_Molecule1437.mol2 | MOL007098 | deoxyneocryptotanshinone | 49.4003470541 | 298.410 | 4.320 | 0.84911 | 0.23900 | 27.165448 | 1 | 3 | 0.28555 | 0.29978877 |  |
| 7036 | MOL007036 | lxx_ds_Molecule1333.mol2 | MOL007036 | 5,6-dihydroxy-7-isopropyl-1,1-dimethyl-2,3-dihydrophenanthren-4-one | 33.7652523626 | 298.410 | 4.377 | 1.18653 | 0.80305 | 14.905333 | 2 | 3 | 0.28585 | 0.29250512 |  |
| 7156 | MOL007156 | lxx_ds_Molecule1520.mol2 | MOL007156 | tanshinone Ⅵ | 45.6373060194 | 296.340 | 2.436 | 0.47507 | -0.28329 | 15.205185 | 2 | 4 | 0.29549 | 0.38057557 |  |
| 7105 | MOL007105 | lxx_ds_Molecule1446.mol2 | MOL007105 | epidanshenspiroketallactone | 68.2731592943 | 284.380 | 2.371 | 0.89598 | 0.61237 | 1.774292 | 0 | 3 | 0.30549 | 0.32837766 |  |
| 7094 | MOL007094 | lxx_ds_Molecule1432.mol2 | MOL007094 | danshenspiroketallactone | 50.4312810266 | 282.360 | 3.237 | 0.87607 | 0.50639 | 15.189896 | 0 | 3 | 0.3067 | 0.33885625 |  |
| 7130 | MOL007130 | lxx_ds_Molecule1487.mol2 | MOL007130 | prolithospermic acid | 64.3709620672 | 314.310 | 2.771 | 0.09914 | -0.74745 | 8.815048 | 4 | 6 | 0.31017 | 0.42357153 |  |
| 7048 | MOL007048 | lxx_ds_Molecule1348.mol2 | MOL007048 | (E)-3-[2-(3,4-dihydroxyphenyl)-7-hydroxy-benzofuran-4-yl]acrylic acid | 48.2436324414 | 312.290 | 3.213 | 0.18274 | -0.88825 | 8.87262 | 4 | 6 | 0.31229 | 0.40258014 |  |
| 7119 | MOL007119 | lxx_ds_Molecule1470.mol2 | MOL007119 | miltionone Ⅰ | 49.6843943314 | 312.390 | 3.328 | 0.34540 | -0.11007 | 41.487782 | 1 | 4 | 0.32125 | 0.34537977 |  |
| 7100 | MOL007100 | lxx_ds_Molecule1439.mol2 | MOL007100 | dihydrotanshinlactone | 38.6847682981 | 266.310 | 2.765 | 1.25897 | 0.80714 | 5.423846 | 0 | 3 | 0.32227 | 0.37922388 |  |
| 7125 | MOL007125 | lxx_ds_Molecule1476.mol2 | MOL007125 | neocryptotanshinone | 52.4879970089 | 314.410 | 3.012 | 0.34714 | -0.13107 | 14.460218 | 2 | 4 | 0.32306 | 0.28012532 |  |
| 7132 | MOL007132 | lxx_ds_Molecule1493.mol2 | MOL007132 | (2R)-3-(3,4-dihydroxyphenyl)-2-[(Z)-3-(3,4-dihydroxyphenyl)acryloyl]oxy-propionic acid | 109.380524105 | 360.340 | 2.687 | -0.33177 | -1.02339 | 2.007406 | 5 | 8 | 0.35119 | 0.40727496 |  |
| 1601 | MOL001601 | lbh_bb_Molecule6.mol2 | MOL001601 | 1,2,5,6-tetrahydrotanshinone | 38.7453867227 | 280.340 | 2.979 | 0.95625 | 0.39024 | 18.053464 | 0 | 3 | 0.35791 | 0.33164033 |  |
| 7101 | MOL007101 | lxx_ds_Molecule1440.mol2 | MOL007101 | dihydrotanshinoneⅠ | 45.0432791888 | 278.320 | 2.858 | 0.95471 | 0.42535 | 18.324948 | 0 | 3 | 0.36015 | 0.39671803 |  |
| 7061 | MOL007061 | lxx_ds_Molecule1380.mol2 | MOL007061 | Methylenetanshinquinone | 37.07319368 | 278.320 | 4.259 | 1.03341 | 0.45834 | 24.32546 | 0 | 3 | 0.36017 | 0.35667476 |  |
| 7127 | MOL007127 | lxx_ds_Molecule1479.mol2 | MOL007127 | 1-methyl-8,9-dihydro-7H-naphtho[5,6-g]benzofuran-6,10,11-trione | 34.7208221315 | 280.290 | 3.211 | 0.49746 | -0.26789 | 37.88986 | 0 | 4 | 0.36634 | 0.33296603 |  |
| 7121 | MOL007121 | lxx_ds_Molecule1472.mol2 | MOL007121 | miltipolone | 36.5561120639 | 300.430 | 2.739 | 0.49945 | 0.16887 | 1.701162 | 1 | 3 | 0.36803 | 0.30114451 |  |
| 7085 | MOL007085 | lxx_ds_Molecule1417.mol2 | MOL007085 | Salvilenone | 30.3836538737 | 292.400 | 4.258 | 1.46267 | 1.07468 | 20.80859 | 0 | 2 | 0.37639 | 0.34710264 |  |
| 7108 | MOL007108 | lxx_ds_Molecule1454.mol2 | MOL007108 | isocryptotanshi-none | 54.9819324596 | 296.390 | 3.585 | 0.92719 | 0.34017 | 31.924564 | 0 | 3 | 0.39449 | 0.29829186 |  |
| 7088 | MOL007088 | lxx_ds_Molecule1424.mol2 | MOL007088 | cryptotanshinone | 52.3419622629 | 296.390 | 3.435 | 0.95130 | 0.50835 | 17.302488 | 0 | 3 | 0.39555 | 0.28997943 |  |
| 7050 | MOL007050 | lxx_ds_Molecule1350.mol2 | MOL007050 | 2-(4-hydroxy-3-methoxyphenyl)-5-(3-hydroxypropyl)-7-methoxy-3-benzofurancarboxaldehyde | 62.7841472598 | 356.400 | 3.575 | 0.34892 | -0.73075 | 7.891163 | 2 | 6 | 0.39628 | 0.23692864 |  |
| 7111 | MOL007111 | lxx_ds_Molecule1458.mol2 | MOL007111 | Isotanshinone II | 49.9160257407 | 294.370 | 4.661 | 1.02666 | 0.44620 | 24.732716 | 0 | 3 | 0.39674 | 0.29877639 |  |
| 7154 | MOL007154 | lxx_ds_Molecule1518.mol2 | MOL007154 | tanshinone iia | 49.8873000352 | 294.370 | 4.661 | 1.04680 | 0.69577 | 23.563268 | 0 | 3 | 0.39781 | 0.30615467 |  |
| 2651 | MOL002651 | lbh_hb_Molecule26.mol2 | MOL002651 | Dehydrotanshinone II A | 43.7622859945 | 292.350 | 4.216 | 1.02366 | 0.51572 | 23.70972 | 0 | 3 | 0.40019 | 0.33311874 |  |
| 7069 | MOL007069 | lxx_ds_Molecule1392.mol2 | MOL007069 | przewaquinone c | 55.7416730964 | 296.340 | 3.306 | 0.41987 | -0.29600 | 23.69784 | 1 | 4 | 0.40408 | 0.31744847 |  |
| 7059 | MOL007059 | lxx_ds_Molecule1372.mol2 | MOL007059 | 3-beta-Hydroxymethyllenetanshiquinone | 32.1610337648 | 294.320 | 3.157 | 0.38377 | -0.47533 | 22.509092 | 1 | 4 | 0.40894 | 0.35916087 |  |
| 7068 | MOL007068 | lxx_ds_Molecule1391.mol2 | MOL007068 | Przewaquinone B | 62.2400596208 | 292.300 | 2.994 | 0.38523 | -0.44654 | 24.940115 | 1 | 4 | 0.41374 | 0.38426891 |  |
| 7058 | MOL007058 | lxx_ds_Molecule1367.mol2 | MOL007058 | formyltanshinone | 73.4446220045 | 290.280 | 3.358 | 0.53948 | -0.28261 | 24.12138 | 0 | 4 | 0.41736 | 0.41212416 |  |
| 7120 | MOL007120 | lxx_ds_Molecule1471.mol2 | MOL007120 | miltionone Ⅱ | 71.0297032063 | 312.390 | 2.136 | 0.62070 | 0.03316 | 2.908396 | 1 | 4 | 0.43711 | 0.28070304 |  |
| 7064 | MOL007064 | lxx_ds_Molecule1387.mol2 | MOL007064 | przewalskin b | 110.3240001 | 330.460 | 3.179 | 0.34407 | 0.22084 | 2.173508 | 1 | 4 | 0.43809 | 0.3150813 |  |
| 7045 | MOL007045 | lxx_ds_Molecule1345.mol2 | MOL007045 | 3α-hydroxytanshinoneⅡa | 44.92933597 | 310.370 | 3.559 | 0.52953 | 0.21794 | 23.784956 | 1 | 4 | 0.44272 | 0.30376598 |  |
| 7155 | MOL007155 | lxx_ds_Molecule1519.mol2 | MOL007155 | (6S)-6-(hydroxymethyl)-1,6-dimethyl-8,9-dihydro-7H-naphtho[8,7-g]benzofuran-10,11-dione | 65.25893771 | 310.370 | 3.570 | 0.4385 | -0.30502 | 23.482207 | 1 | 4 | 0.44871 | 0.28854188 |  |
| 7079 | MOL007079 | lxx_ds_Molecule1406.mol2 | MOL007079 | tanshinaldehyde | 52.4747043036 | 308.350 | 3.827 | 0.56737 | -0.07021 | 23.492099 | 0 | 4 | 0.45196 | 0.32087722 |  |
| 7070 | MOL007070 | lxx_ds_Molecule1393.mol2 | MOL007070 | (6S,7R)-6,7-dihydroxy-1,6-dimethyl-8,9-dihydro-7H-naphtho[8,7-g]benzofuran-10,11-dione | 41.3104570553 | 312.340 | 2.339 | -0.05953 | -0.67759 | 22.540771 | 2 | 5 | 0.453 | 0.32285529 |  |
| 7152 | MOL007152 | lxx_ds_Molecule1516.mol2 | MOL007152 | Przewaquinone E | 42.8548520397 | 312.340 | 2.339 | -0.03889 | -0.64999 | 22.439703 | 2 | 5 | 0.45301 | 0.32332131 |  |
| 7151 | MOL007151 | lxx_ds_Molecule1515.mol2 | MOL007151 | Tanshindiol B | 42.6658104891 | 312.340 | 2.339 | 0.05436 | -0.63468 | 22.251379 | 2 | 5 | 0.45303 | 0.32894623 |  |
| 7150 | MOL007150 | lxx_ds_Molecule1514.mol2 | MOL007150 | (6S)-6-hydroxy-1-methyl-6-methylol-8,9-dihydro-7H-naphtho[8,7-g]benzofuran-10,11-quinone | 75.3858784662 | 312.340 | 2.418 | 0.03025 | -0.73707 | 23.451995 | 2 | 5 | 0.4551 | 0.2917442 |  |
| 7071 | MOL007071 | lxx_ds_Molecule1394.mol2 | MOL007071 | przewaquinone f | 40.3078839947 | 312.340 | 2.065 | -0.08941 | -0.90018 | 22.448824 | 2 | 5 | 0.45925 | 0.29433328 |  |
| 7082 | MOL007082 | lxx_ds_Molecule1409.mol2 | MOL007082 | Danshenol A | 56.9652489923 | 336.410 | 2.008 | 0.33496 | -0.01425 | 5.146793 | 1 | 4 | 0.52172 | 0.34462488 |  |
| 7093 | MOL007093 | lxx_ds_Molecule1431.mol2 | MOL007093 | dan-shexinkum d | 38.8830210096 | 336.410 | 2.832 | 0.67117 | -0.14900 | 30.00133 | 1 | 4 | 0.55453 | 0.35005915 |  |
| 7081 | MOL007081 | lxx_ds_Molecule1408.mol2 | MOL007081 | Danshenol B | 57.950875299 | 354.480 | 2.585 | 0.53345 | 0.10663 | 4.279868 | 1 | 4 | 0.55764 | 0.2957541 |  |
| 7141 | MOL007141 | lxx_ds_Molecule1502.mol2 | MOL007141 | salvianolic acid g | 45.5648557799 | 340.300 | 2.201 | -0.14033 | -0.97080 | 2.402636 | 4 | 7 | 0.60602 | 0.4549855 |  |
| 7063 | MOL007063 | lxx_ds_Molecule1386.mol2 | MOL007063 | przewalskin a | 37.1065006596 | 398.490 | 2.253 | -0.26273 | -0.69384 | 1.633763 | 1 | 6 | 0.64901 | 0.37890354 |  |
| 7051 | MOL007051 | lxx_ds_Molecule1352.mol2 | MOL007051 | 6-o-syringyl-8-o-acetyl shanzhiside methyl ester | 46.6906586042 | 628.640 | -1.134 | -1.72822 | -2.08499 | 9.938881 | 5 | 16 | 0.71145 | 0.21537822 |  |
| 7142 | MOL007142 | lxx_ds_Molecule1503.mol2 | MOL007142 | salvianolic acid j | 43.3760499149 | 538.490 | 3.783 | -0.81858 | -2.14397 | 5.771558 | 6 | 12 | 0.72497 | 0.43521604 |  |
| 1771 | MOL001771 | lbh_blg_Molecule154_qt.mol2 | MOL001771 | poriferast-5-en-3beta-ol | 36.9139058327 | 414.790 | 8.084 | 1.45001 | 1.14299 | 5.066671 | 1 | 1 | 0.75034 | 0 |  |
| 2776 | MOL002776 | lbh_hh_Molecule79.mol2 | MOL002776 | Baicalin | 40.1236099599 | 446.390 | 0.639 | -0.84777 | -1.74426 | 17.358993 | 6 | 11 | 0.75264 | 0.36380428 |  |
| 1659 | MOL001659 | lbh_bhssc_Molecule27.mol2 | MOL001659 | Poriferasterol | 43.8298515785 | 412.770 | 7.640 | 1.43659 | 1.03472 | 5.341727 | 1 | 1 | 0.75596 | 0.21613795 |  |
| 6824 | MOL006824 | lp_ygz_Molecule382.mol2 | MOL006824 | α-amyrin | 39.5120897831 | 426.800 | 7.349 | 1.37278 | 1.20132 | 3.064368 | 1 | 1 | 0.76221 | 0.23126018 |  |
| Dingxiang |  |  |  |  |  |  |  |  |  |  |  |  |  |  |  |
| molecule_ID | MOL_ID | file_ID | MOL_ID | molecule_name | ob | mw | alogp | caco2 | bbb | halflife | hdon | hacc | dl | FASA |  |
| 358 | MOL000358 | fyp_hjs_Molecule461_qt.mol2 | MOL000358 | beta-sitosterol | 36.9139058327 | 414.790 | 8.084 | 1.32463 | 0.98588 | 5.355491 | 1 | 1 | 0.75123 | 0.22550575 |  |
| 422 | MOL000422 | fyp_hq_Molecule260.mol2 | MOL000422 | kaempferol | 41.8822495352 | 286.250 | 1.771 | 0.26096 | -0.55335 | 14.743371 | 4 | 6 | 0.24066 | 0 |  |
| 1749 | MOL001749 | lbh_blg_Molecule128.mol2 | MOL001749 | ZINC03860434 | 43.5933254731 | 390.620 | 7.574 | 1.04115 | 0.59802 | 3.971174 | 0 | 4 | 0.34527 | 0.29546189 |  |
| 449 | MOL000449 | fyp_htp_Molecule484.mol2 | MOL000449 | Stigmasterol | 43.8298515785 | 412.770 | 7.640 | 1.44458 | 1.00045 | 5.574595 | 1 | 1 | 0.75665 | 0.21679397 |  |
| 98 | MOL000098 | fyp_cg_Molecule36_qt.mol2 | MOL000098 | quercetin | 46.4333481195 | 302.250 | 1.504 | 0.04842 | -0.76890 | 14.400548 | 5 | 7 | 0.27525 | 0.38453352 |  |
| 13219 | MOL013219 | zwj_dx_Molecule161_qt.mol2 | MOL013219 | Strictosamide_qt | 76.3027165324 | 336.420 | 1.984 | 0.58938 | -0.16263 | 8.836935 | 2 | 4 | 0.76283 | 0.2712914 |  |
| Bingpian |  |  |  |  |  |  |  |  |  |  |  |  |  |  |  |
| molecule_ID | MOL_ID | file_ID | MOL_ID | molecule_name | ob | mw | alogp | caco2 | bbb | halflife | hdon | hacc | dl | FASA |  |
| 6862 | MOL006862 | lxx_bp_Molecule1301.mol2 | MOL006862 | bronyl acetate | 59.29526304 | 447.550 | 4.022 | 0.22994 | -0.14753 | 8.811231 | 1 | 8 | 0.51159 | 0.23550934 |  |
| 6861 | MOL006861 | lxx_bp_Molecule1300.mol2 | MOL006861 | asiatic acid | 41.3828121867 | 488.780 | 4.296 | -0.28812 | -0.93104 | 4.276504 | 4 | 5 | 0.71097 | 0.24092095 |  |
| 6865 | MOL006865 | lxx_bp_Molecule1308.mol2 | MOL006865 | dipterocarpol | 41.7080609956 | 442.800 | 6.920 | 1.01106 | 0.41688 | 7.059028 | 1 | 2 | 0.76437 | 0.27177948 |  |

| Table S2. Components of GXST with targets. | | | | | | | |
| --- | --- | --- | --- | --- | --- | --- | --- |
| Guangzao | Code | Danshen | Code | Bingpian | Code | Dingxiang | Code |
| MOL004328 | GZ1 | MOL001601 | DS1 | MOL006861 | BP1 | MOL013219 | DX2 |
| MOL000098 | GD1 | MOL001659 | DS2 | MOL006862 | BP2 | MOL001749 | DX3 |
| MOL000422 | GD2 | MOL001771 | DS3 |  |  | MOL000358 | GD3 |
| MOL001040 | GZ2 | MOL001942 | DS4 |  |  | MOL000422 | GD2 |
| MOL001490 | GZ3 | MOL002222 | DS5 |  |  | MOL000449 | DX4 |
| MOL000358 | GD3 | MOL002651 | DS6 |  |  | MOL000098 | GD1 |
| MOL001002 | GZ4 | MOL000569 | DS7 |  |  |  |  |
| MOL000096 | GZ5 | MOL000006 | DS8 |  |  |  |  |
| MOL001736 | GZ6 | MOL007036 | DS9 |  |  |  |  |
|  |  | MOL007041 | DS10 |  |  |  |  |
|  |  | MOL007045 | DS11 |  |  |  |  |
|  |  | MOL007048 | DS12 |  |  |  |  |
|  |  | MOL007049 | DS13 |  |  |  |  |
|  |  | MOL007050 | DS14 |  |  |  |  |
|  |  | MOL007058 | DS15 |  |  |  |  |
|  |  | MOL007059 | DS16 |  |  |  |  |
|  |  | MOL007061 | DS17 |  |  |  |  |
|  |  | MOL007063 | DS18 |  |  |  |  |
|  |  | MOL007064 | DS19 |  |  |  |  |
|  |  | MOL007068 | DS20 |  |  |  |  |
|  |  | MOL007069 | DS21 |  |  |  |  |
|  |  | MOL007070 | DS22 |  |  |  |  |
|  |  | MOL007071 | DS23 |  |  |  |  |
|  |  | MOL007077 | DS24 |  |  |  |  |
|  |  | MOL007079 | DS25 |  |  |  |  |
|  |  | MOL007081 | DS26 |  |  |  |  |
|  |  | MOL007082 | DS27 |  |  |  |  |
|  |  | MOL007085 | DS28 |  |  |  |  |
|  |  | MOL007088 | DS29 |  |  |  |  |
|  |  | MOL007093 | DS30 |  |  |  |  |
|  |  | MOL007094 | DS31 |  |  |  |  |
|  |  | MOL007098 | DS32 |  |  |  |  |
|  |  | MOL007100 | DS33 |  |  |  |  |
|  |  | MOL007101 | DS34 |  |  |  |  |
|  |  | MOL007105 | DS35 |  |  |  |  |
|  |  | MOL007107 | DS36 |  |  |  |  |
|  |  | MOL007108 | DS37 |  |  |  |  |
|  |  | MOL007111 | DS38 |  |  |  |  |
|  |  | MOL007119 | DS39 |  |  |  |  |
|  |  | MOL007120 | DS40 |  |  |  |  |
|  |  | MOL007121 | DS41 |  |  |  |  |
|  |  | MOL007122 | DS42 |  |  |  |  |
|  |  | MOL007124 | DS43 |  |  |  |  |
|  |  | MOL007125 | DS44 |  |  |  |  |
|  |  | MOL007127 | DS45 |  |  |  |  |
|  |  | MOL007130 | DS46 |  |  |  |  |
|  |  | MOL007132 | DS47 |  |  |  |  |
|  |  | MOL007141 | DS48 |  |  |  |  |
|  |  | MOL007142 | DS49 |  |  |  |  |
|  |  | MOL007143 | DS50 |  |  |  |  |
|  |  | MOL007145 | DS51 |  |  |  |  |
|  |  | MOL007150 | DS52 |  |  |  |  |
|  |  | MOL007151 | DS53 |  |  |  |  |
|  |  | MOL007152 | DS54 |  |  |  |  |
|  |  | MOL007154 | DS55 |  |  |  |  |
|  |  | MOL007155 | DS56 |  |  |  |  |
|  |  | MOL007156 | DS57 |  |  |  |  |

| Table S3. Targets corresponding to each component in GXST. | |
| --- | --- |
| GXST components | Target gene symbol |
| BP1 | CD81 |
| BP1 | PTPN1 |
| BP1 | HSD11B1 |
| BP1 | AKR1B10 |
| BP1 | POLB |
| BP1 | PTPN2 |
| BP1 | CDC25B |
| BP1 | RORC |
| BP1 | PTPRF |
| BP1 | ACP1 |
| BP1 | PDE4D |
| BP1 | PLA2G1B |
| BP1 | PTGES |
| BP1 | CES2 |
| BP1 | NOS2 |
| BP1 | PREP |
| BP1 | PPARG |
| BP1 | LTB4R |
| BP1 | HMGCR |
| BP1 | FNTA |
| BP1 | HSD11B2 |
| BP1 | FABP1 |
| BP1 | PTPN6 |
| BP1 | PTGS2 |
| BP1 | NR3C1 |
| BP1 | CDC25A |
| BP1 | ESR2 |
| BP1 | BACE1 |
| BP1 | AR |
| BP1 | SERPINA6 |
| BP1 | SHBG |
| BP1 | G6PD |
| BP1 | CYP51A1 |
| BP1 | FABP4 |
| BP1 | PTGER2 |
| BP1 | PPARA |
| BP1 | FABP3 |
| BP1 | FABP5 |
| BP1 | FAAH |
| BP1 | TERT |
| BP1 | PPARD |
| BP1 | PGR |
| BP1 | ALOX5 |
| BP1 | PTGER4 |
| BP1 | PTPN11 |
| BP1 | PTGER1 |
| BP1 | NR1H3 |
| BP1 | PTGS1 |
| BP1 | PTGIR |
| BP1 | PTGDR2 |
| BP1 | AMPD2 |
| BP1 | NPC1L1 |
| BP1 | SIGMAR1 |
| BP1 | CYP17A1 |
| BP1 | MME |
| BP1 | AGTR1 |
| BP1 | GPBAR1 |
| BP1 | MAPK3 |
| BP1 | MMP3 |
| BP1 | MMP1 |
| BP1 | MMP2 |
| BP1 | PTGDR |
| BP1 | EDNRB |
| BP1 | EDNRA |
| BP1 | PTGER3 |
| BP1 | MDM2 |
| BP1 | AURKB |
| BP1 | AURKA |
| BP1 | CASP3 |
| BP1 | CASP8 |
| BP1 | PTGFR |
| BP1 | S1PR2 |
| BP1 | ADAMTS5 |
| BP1 | MMP14 |
| BP1 | PRKCH |
| BP1 | CTSA |
| BP1 | SCD |
| BP1 | SLC6A4 |
| BP1 | CNR1 |
| BP1 | ITGB1 |
| BP1 | IL6 |
| BP1 | GLUL |
| BP1 | MAPK1 |
| BP1 | RORA |
| BP1 | FDFT1 |
| BP1 | OXTR |
| BP1 | CCR1 |
| BP1 | REN |
| BP1 | ITGB7 |
| BP1 | P2RX3 |
| BP1 | TNF |
| BP1 | PIK3CA |
| BP1 | CASR |
| BP1 | NR1H4 |
| BP1 | PGGT1B |
| BP1 | ENPP2 |
| BP1 | THRA |
| BP1 | THRB |
| BP1 | PIM1 |
| BP1 | AMPD3 |
| BP2 | ADORA1 |
| BP2 | ADORA2B |
| BP2 | MMP13 |
| BP2 | PDE10A |
| BP2 | MET |
| BP2 | PORCN |
| BP2 | CSF1R |
| BP2 | PDGFRB |
| BP2 | FLT4 |
| BP2 | SYK |
| BP2 | HCRTR2 |
| BP2 | HCRTR1 |
| BP2 | SRC |
| BP2 | CCNA2 |
| BP2 | ERBB2 |
| BP2 | EGFR |
| BP2 | NTRK1 |
| BP2 | CRHR1 |
| BP2 | NR5A1 |
| BP2 | PIK3CA |
| BP2 | GCK |
| BP2 | TAOK1 |
| BP2 | PTK2B |
| BP2 | MAPKAPK2 |
| BP2 | RPS6KA3 |
| BP2 | CASP3 |
| BP2 | CASP6 |
| BP2 | CASP7 |
| BP2 | AURKA |
| BP2 | PDE5A |
| BP2 | KDR |
| BP2 | PIK3CD |
| BP2 | PFKFB3 |
| BP2 | CCND1 |
| BP2 | CCNE2 |
| BP2 | RPS6KA2 |
| BP2 | PARP1 |
| BP2 | THRB |
| BP2 | PIK3CA |
| BP2 | PIK3CD |
| BP2 | MAPK14 |
| BP2 | PIK3R1 |
| BP2 | PIK3CG |
| BP2 | MAPK11 |
| BP2 | ACACB |
| BP2 | AURKB |
| BP2 | PTK2 |
| BP2 | MAPK1 |
| BP2 | MMP2 |
| BP2 | KNG1 |
| BP2 | CFD |
| BP2 | PDPK1 |
| BP2 | TGM2 |
| BP2 | TGM1 |
| BP2 | F13A1 |
| BP2 | F10 |
| BP2 | GSK3B |
| BP2 | CTSK |
| BP2 | MTOR |
| BP2 | HSP90AA1 |
| BP2 | CSNK2A1 |
| BP2 | LIPG |
| BP2 | TNIK |
| BP2 | TRPV1 |
| BP2 | FKBP1A |
| BP2 | ZAP70 |
| BP2 | JAK2 |
| BP2 | CCND3 |
| BP2 | BTK |
| BP2 | HDAC6 |
| BP2 | HDAC1 |
| BP2 | FGFR2 |
| BP2 | FAP |
| BP2 | ROCK2 |
| BP2 | CAPN1 |
| BP2 | SLC8A1 |
| BP2 | FLT1 |
| BP2 | KIT |
| BP2 | ELANE |
| BP2 | PDE7A |
| BP2 | CFTR |
| BP2 | ITGAL |
| BP2 | ITGAL |
| BP2 | SCD |
| BP2 | PDE4A |
| BP2 | DPP4 |
| BP2 | TSPO |
| BP2 | SCN5A |
| BP2 | INSR |
| BP2 | PDGFRA |
| BP2 | LCK |
| BP2 | PIK3CB |
| BP2 | BDKRB2 |
| BP2 | HCK |
| BP2 | FGFR1 |
| BP2 | LYN |
| BP2 | SCN9A |
| BP2 | ABCB1 |
| BP2 | CDK2 |
| BP2 | CDK1 |
| DS1 | PTGS1 |
| DS1 | CHRM3 |
| DS1 | CHRM1 |
| DS1 | SCN5A |
| DS1 | PTGS2 |
| DS1 | HTR3A |
| DS1 | RXRA |
| DS1 | OPRD1 |
| DS1 | PDE3A |
| DS1 | ADRA1A |
| DS1 | SLC6A3 |
| DS1 | ADRB2 |
| DS1 | OPRM1 |
| DS1 | GABRA1 |
| DS1 | NCOA1 |
| DS1 | SLC6A4 |
| DS1 | IGHG1 |
| DS2 | PGR |
| DS2 | NR3C2 |
| DS3 | PGR |
| DS4 | PTGS2 |
| DS5 | CHRM3 |
| DS5 | CHRM1 |
| DS5 | SCN5A |
| DS5 | PTGS2 |
| DS5 | OPRD1 |
| DS5 | ACHE |
| DS5 | ADRA1A |
| DS5 | ADRB2 |
| DS5 | DRD2 |
| DS5 | OPRM1 |
| DS6 | CHRM3 |
| DS6 | CHRM1 |
| DS6 | ESR1 |
| DS6 | AR |
| DS6 | SCN5A |
| DS6 | PTGS2 |
| DS6 | OPRD1 |
| DS6 | ACHE |
| DS6 | ADRA1A |
| DS6 | ADRB2 |
| DS6 | OPRM1 |
| DS6 | GABRA1 |
| DS6 | NCOA1 |
| DS7 | PTGS2 |
| DS7 | AKR1B1 |
| DS8 | PTGS1 |
| DS8 | AR |
| DS8 | PTGS2 |
| DS8 | PRSS1 |
| DS8 | RELA |
| DS8 | EGFR |
| DS8 | AKT1 |
| DS8 | VEGFA |
| DS8 | CCND1 |
| DS8 | BCL2L1 |
| DS8 | CDKN1A |
| DS8 | MMP2 |
| DS8 | MMP9 |
| DS8 | MAPK1 |
| DS8 | IL10 |
| DS8 | RB1 |
| DS8 | TNF |
| DS8 | JUN |
| DS8 | IL6 |
| DS8 | CASP3 |
| DS8 | TP53 |
| DS8 | NFKBIA |
| DS8 | XDH |
| DS8 | TOP1 |
| DS8 | MDM2 |
| DS8 | APP |
| DS8 | MMP1 |
| DS8 | PCNA |
| DS8 | ERBB2 |
| DS8 | PPARG |
| DS8 | HMOX1 |
| DS8 | CASP7 |
| DS8 | ICAM1 |
| DS8 | MCL1 |
| DS8 | BIRC5 |
| DS8 | IL2 |
| DS8 | TYR |
| DS8 | IFNG |
| DS8 | IL4 |
| DS8 | TOP2A |
| DS8 | GSTP1 |
| DS8 | INSR |
| DS8 | CD40LG |
| DS8 | ADCY2 |
| DS8 | MET |
| DS9 | PTGS1 |
| DS9 | CHRM3 |
| DS9 | CHRM1 |
| DS9 | SCN5A |
| DS9 | PTGS2 |
| DS9 | RXRA |
| DS9 | ACHE |
| DS9 | ADRA1A |
| DS9 | ADRB2 |
| DS9 | OPRM1 |
| DS9 | IGHG1 |
| DS9 | NCOA1 |
| DS10 | PTGS1 |
| DS10 | CHRM3 |
| DS10 | CHRM1 |
| DS10 | ESR1 |
| DS10 | AR |
| DS10 | SCN5A |
| DS10 | PTGS2 |
| DS10 | HTR3A |
| DS10 | RXRA |
| DS10 | ADRA1A |
| DS10 | SLC6A3 |
| DS10 | ADRB2 |
| DS10 | SLC6A4 |
| DS10 | OPRM1 |
| DS10 | GABRA1 |
| DS10 | IGHG1 |
| DS10 | CCNA2 |
| DS11 | CHRM1 |
| DS11 | SCN5A |
| DS11 | PTGS2 |
| DS11 | OPRD1 |
| DS11 | ACHE |
| DS11 | ADRB2 |
| DS11 | OPRM1 |
| DS11 | PRSS1 |
| DS11 | NCOA1 |
| DS12 | PTGS2 |
| DS13 | PTGS1 |
| DS13 | CHRM3 |
| DS13 | CHRM1 |
| DS13 | ESR1 |
| DS13 | AR |
| DS13 | SCN5A |
| DS13 | PTGS2 |
| DS13 | ADRA2A |
| DS13 | RXRA |
| DS13 | OPRD1 |
| DS13 | ADRA1A |
| DS13 | SLC6A3 |
| DS13 | ADRB2 |
| DS13 | SLC6A4 |
| DS13 | DRD2 |
| DS13 | OPRM1 |
| DS13 | GABRA1 |
| DS13 | NCOA1 |
| DS14 | NOS2 |
| DS14 | ESR1 |
| DS14 | AR |
| DS14 | ESR2 |
| DS14 | GSK3B |
| DS14 | CCNA2 |
| DS15 | AR |
| DS15 | PTGS2 |
| DS15 | RXRA |
| DS15 | NCOA1 |
| DS16 | CHRM1 |
| DS16 | PTGS2 |
| DS16 | RXRA |
| DS16 | OPRD1 |
| DS16 | ACHE |
| DS16 | ADRA1A |
| DS16 | ADRB2 |
| DS16 | OPRM1 |
| DS16 | IGHG1 |
| DS16 | PRSS1 |
| DS16 | NCOA1 |
| DS17 | CHRM3 |
| DS17 | CHRM1 |
| DS17 | SCN5A |
| DS17 | PTGS2 |
| DS17 | RXRA |
| DS17 | OPRD1 |
| DS17 | ACHE |
| DS17 | ADRA1A |
| DS17 | ADRB2 |
| DS17 | SLC6A4 |
| DS17 | OPRM1 |
| DS17 | GABRA1 |
| DS17 | IGHG1 |
| DS17 | PRSS1 |
| DS17 | NCOA1 |
| DS18 | NR3C2 |
| DS18 | NR3C1 |
| DS19 | PTGS2 |
| DS19 | PGR |
| DS19 | NR3C2 |
| DS19 | NR3C1 |
| DS19 | NCOA1 |
| DS20 | PTGS2 |
| DS20 | RXRA |
| DS20 | IGHG1 |
| DS20 | PRSS1 |
| DS20 | NCOA1 |
| DS21 | PTGS1 |
| DS21 | CHRM3 |
| DS21 | CHRM1 |
| DS21 | SCN5A |
| DS21 | PTGS2 |
| DS21 | OPRD1 |
| DS21 | ACHE |
| DS21 | ADRA1A |
| DS21 | ADRB2 |
| DS21 | OPRM1 |
| DS21 | GABRA1 |
| DS21 | NCOA1 |
| DS22 | PTGS2 |
| DS22 | ACHE |
| DS22 | PRSS1 |
| DS22 | NCOA1 |
| DS23 | PTGS2 |
| DS23 | PRSS1 |
| DS23 | NCOA1 |
| DS24 | PTGS2 |
| DS25 | CHRM1 |
| DS25 | PTGS2 |
| DS25 | OPRD1 |
| DS25 | ACHE |
| DS25 | ADRB2 |
| DS25 | OPRM1 |
| DS25 | PRSS1 |
| DS25 | NCOA1 |
| DS26 | PTGS2 |
| DS26 | PGR |
| DS26 | OPRM1 |
| DS26 | NR3C1 |
| DS26 | NCOA1 |
| DS27 | PTGS1 |
| DS27 | KCNH2 |
| DS27 | SCN5A |
| DS27 | PTGS2 |
| DS27 | RXRA |
| DS27 | NCOA1 |
| DS28 | PTGS1 |
| DS28 | ESR1 |
| DS28 | AR |
| DS28 | PTGS2 |
| DS28 | HTR3A |
| DS28 | ESR2 |
| DS29 | PTGS1 |
| DS29 | CHRM3 |
| DS29 | CHRM1 |
| DS29 | SCN5A |
| DS29 | PTGS2 |
| DS29 | OPRD1 |
| DS29 | ADRA1A |
| DS29 | ADRB2 |
| DS29 | OPRM1 |
| DS29 | NCOA1 |
| DS29 | PGR |
| DS29 | GABRA1 |
| DS29 | RELA |
| DS29 | STAT3 |
| DS29 | CCND1 |
| DS29 | BCL2L1 |
| DS29 | TNF |
| DS29 | APP |
| DS29 | EDN1 |
| DS29 | BIRC5 |
| DS30 | NOS2 |
| DS30 | PTGS1 |
| DS30 | KCNH2 |
| DS30 | CHRM1 |
| DS30 | ESR1 |
| DS30 | AR |
| DS30 | SCN5A |
| DS30 | PTGS2 |
| DS30 | RXRA |
| DS30 | ACHE |
| DS30 | ADRB2 |
| DS30 | ESR2 |
| DS30 | GSK3B |
| DS30 | IGHG1 |
| DS30 | PRSS1 |
| DS30 | CCNA2 |
| DS30 | NCOA1 |
| DS31 | PTGS1 |
| DS31 | CHRM3 |
| DS31 | CHRM1 |
| DS31 | ESR1 |
| DS31 | SCN5A |
| DS31 | PTGS2 |
| DS31 | RXRA |
| DS31 | ACHE |
| DS31 | ADRA1A |
| DS31 | ADRB2 |
| DS31 | CHRNA2 |
| DS31 | SLC6A4 |
| DS31 | OPRM1 |
| DS31 | GABRA1 |
| DS32 | PTGS1 |
| DS32 | CHRM3 |
| DS32 | CHRM1 |
| DS32 | ESR1 |
| DS32 | AR |
| DS32 | SCN5A |
| DS32 | PTGS2 |
| DS32 | RXRA |
| DS32 | OPRD1 |
| DS32 | ADRA1A |
| DS32 | ADRB2 |
| DS32 | OPRM1 |
| DS32 | GSK3B |
| DS32 | IGHG1 |
| DS32 | NCOA1 |
| DS33 | NOS2 |
| DS33 | PTGS1 |
| DS33 | CHRM3 |
| DS33 | CHRM1 |
| DS33 | ESR1 |
| DS33 | AR |
| DS33 | SCN5A |
| DS33 | PTGS2 |
| DS33 | HTR3A |
| DS33 | RXRA |
| DS33 | ACHE |
| DS33 | PDE3A |
| DS33 | ADRA1A |
| DS33 | SLC6A3 |
| DS33 | ADRB2 |
| DS33 | SLC6A4 |
| DS33 | OPRM1 |
| DS33 | GABRA1 |
| DS33 | GSK3B |
| DS33 | IGHG1 |
| DS33 | PRSS1 |
| DS33 | CCNA2 |
| DS34 | PTGS1 |
| DS34 | SCN5A |
| DS34 | PTGS2 |
| DS34 | HTR3A |
| DS34 | RXRA |
| DS34 | ADRA1A |
| DS34 | ADRB2 |
| DS34 | GABRA1 |
| DS34 | IGHG1 |
| DS34 | NCOA1 |
| DS35 | PTGS1 |
| DS35 | CHRM3 |
| DS35 | CHRM1 |
| DS35 | ESR1 |
| DS35 | SCN5A |
| DS35 | PTGS2 |
| DS35 | RXRA |
| DS35 | OPRD1 |
| DS35 | PDE3A |
| DS35 | ADRA1A |
| DS35 | ADRB2 |
| DS35 | SLC6A4 |
| DS35 | OPRM1 |
| DS35 | GABRA1 |
| DS36 | CHRM3 |
| DS36 | CHRM1 |
| DS36 | SCN5A |
| DS36 | ACHE |
| DS36 | ADRA1A |
| DS36 | ADRB2 |
| DS36 | OPRM1 |
| DS37 | NOS2 |
| DS37 | PTGS1 |
| DS37 | CHRM3 |
| DS37 | CHRM1 |
| DS37 | ESR1 |
| DS37 | AR |
| DS37 | SCN5A |
| DS37 | PTGS2 |
| DS37 | RXRA |
| DS37 | OPRD1 |
| DS37 | ACHE |
| DS37 | ADRA1A |
| DS37 | ADRB2 |
| DS37 | DRD2 |
| DS37 | OPRM1 |
| DS37 | GABRA1 |
| DS37 | PRSS1 |
| DS37 | NCOA1 |
| DS38 | NOS2 |
| DS38 | CHRM3 |
| DS38 | CHRM1 |
| DS38 | ESR1 |
| DS38 | AR |
| DS38 | SCN5A |
| DS38 | PTGS2 |
| DS38 | RXRA |
| DS38 | OPRD1 |
| DS38 | ACHE |
| DS38 | ADRA1A |
| DS38 | ADRB2 |
| DS38 | OPRM1 |
| DS38 | ESR2 |
| DS38 | GABRA1 |
| DS38 | GSK3B |
| DS38 | CCNA2 |
| DS39 | PTGS1 |
| DS39 | CHRM3 |
| DS39 | CHRM1 |
| DS39 | ESR1 |
| DS39 | AR |
| DS39 | SCN5A |
| DS39 | PTGS2 |
| DS39 | RXRA |
| DS39 | OPRD1 |
| DS39 | ADRA1A |
| DS39 | ADRB2 |
| DS39 | OPRM1 |
| DS39 | NR3C1 |
| DS39 | GSK3B |
| DS39 | IGHG1 |
| DS39 | CCNA2 |
| DS39 | NCOA1 |
| DS40 | PTGS2 |
| DS40 | ACHE |
| DS40 | PGR |
| DS40 | NR3C1 |
| DS40 | NCOA1 |
| DS41 | ESR1 |
| DS41 | ACHE |
| DS42 | PTGS1 |
| DS42 | CHRM3 |
| DS42 | CHRM1 |
| DS42 | ESR1 |
| DS42 | AR |
| DS42 | DRD5 |
| DS42 | SCN5A |
| DS42 | PTGS2 |
| DS42 | RXRA |
| DS42 | OPRD1 |
| DS42 | ADRA1A |
| DS42 | SLC6A3 |
| DS42 | ADRB2 |
| DS42 | OPRM1 |
| DS43 | PTGS1 |
| DS43 | CHRM3 |
| DS43 | CHRM1 |
| DS43 | ESR1 |
| DS43 | AR |
| DS43 | SCN5A |
| DS43 | PTGS2 |
| DS43 | RXRA |
| DS43 | OPRD1 |
| DS43 | PDE3A |
| DS43 | ADRA1A |
| DS43 | SLC6A3 |
| DS43 | ADRB2 |
| DS43 | SLC6A4 |
| DS43 | OPRM1 |
| DS43 | GABRA1 |
| DS43 | GSK3B |
| DS43 | CCNA2 |
| DS44 | PTGS1 |
| DS44 | CHRM3 |
| DS44 | CHRM1 |
| DS44 | SCN5A |
| DS44 | PTGS2 |
| DS44 | ADRB2 |
| DS44 | OPRM1 |
| DS44 | IGHG1 |
| DS44 | NCOA1 |
| DS45 | PTGS1 |
| DS45 | CHRM3 |
| DS45 | SCN5A |
| DS45 | PTGS2 |
| DS45 | RXRA |
| DS45 | ACHE |
| DS45 | ADRA1A |
| DS45 | ADRB2 |
| DS45 | OPRM1 |
| DS45 | GABRA1 |
| DS45 | IGHG1 |
| DS45 | NCOA1 |
| DS46 | NOS2 |
| DS46 | PTGS1 |
| DS46 | ESR1 |
| DS46 | AR |
| DS46 | PTGS2 |
| DS46 | PRSS1 |
| DS47 | ESR1 |
| DS47 | AR |
| DS47 | PTGS2 |
| DS47 | PRSS1 |
| DS47 | CCNA2 |
| DS48 | PTGS2 |
| DS49 | F7 |
| DS49 | PRSS1 |
| DS50 | PTGS2 |
| DS50 | RXRA |
| DS50 | ACHE |
| DS50 | PGR |
| DS50 | NR3C1 |
| DS50 | NCOA1 |
| DS51 | PTGS1 |
| DS51 | CHRM3 |
| DS51 | CHRM1 |
| DS51 | DRD5 |
| DS51 | SCN5A |
| DS51 | PTGS2 |
| DS51 | ADRA2A |
| DS51 | HTR3A |
| DS51 | OPRD1 |
| DS51 | ACHE |
| DS51 | PDE3A |
| DS51 | SLC6A2 |
| DS51 | ADRA1A |
| DS51 | ADRA2B |
| DS51 | SLC6A3 |
| DS51 | ADRB2 |
| DS51 | CHRNA2 |
| DS51 | SLC6A4 |
| DS51 | DRD2 |
| DS51 | OPRM1 |
| DS51 | GABRA1 |
| DS52 | PTGS2 |
| DS52 | ACHE |
| DS52 | PRSS1 |
| DS52 | NCOA1 |
| DS53 | PTGS2 |
| DS53 | ACHE |
| DS53 | NCOA1 |
| DS54 | PTGS2 |
| DS54 | ACHE |
| DS54 | NCOA1 |
| DS55 | CHRM3 |
| DS55 | CHRM1 |
| DS55 | SCN5A |
| DS55 | PTGS2 |
| DS55 | OPRD1 |
| DS55 | ACHE |
| DS55 | ADRA1A |
| DS55 | ADRB2 |
| DS55 | OPRM1 |
| DS55 | NCOA1 |
| DS55 | RXRA |
| DS55 | RELA |
| DS55 | BCL2 |
| DS55 | FOS |
| DS55 | CDKN1A |
| DS55 | MMP9 |
| DS55 | JUN |
| DS55 | CASP3 |
| DS55 | TP53 |
| DS55 | NFKBIA |
| DS55 | FASN |
| DS55 | EDNRA |
| DS55 | EDN1 |
| DS55 | CYP3A4 |
| DS55 | CYP1A2 |
| DS55 | MYC |
| DS55 | CYP1A1 |
| DS55 | NR1I2 |
| DS55 | ECE1 |
| DS55 | CALCR |
| DS55 | ITGB3 |
| DS56 | CHRM1 |
| DS56 | SCN5A |
| DS56 | PTGS2 |
| DS56 | OPRD1 |
| DS56 | ACHE |
| DS56 | ADRA1A |
| DS56 | ADRB2 |
| DS56 | OPRM1 |
| DS56 | PRSS1 |
| DS56 | NCOA1 |
| DS57 | PTGS1 |
| DS57 | ESR1 |
| DS57 | AR |
| DS57 | SCN5A |
| DS57 | PTGS2 |
| DS57 | IGHG1 |
| DS57 | NCOA1 |
| DX2 | PTGS1 |
| DX2 | KCNH2 |
| DX2 | CHRM1 |
| DX2 | AR |
| DX2 | SCN5A |
| DX2 | PTGS2 |
| DX2 | RXRA |
| DX2 | ACHE |
| DX2 | PRSS1 |
| DX2 | NCOA1 |
| DX3 | SCN5A |
| DX3 | CHRM3 |
| DX3 | ADRB2 |
| DX3 | CHRM1 |
| GD3 | PGR |
| GD3 | PTGS1 |
| GD3 | PTGS2 |
| GD3 | KCNH2 |
| GD3 | CHRM3 |
| GD3 | CHRM1 |
| GD3 | SCN5A |
| GD3 | ADRA1A |
| GD3 | ADRB2 |
| GD3 | CHRNA2 |
| GD3 | SLC6A4 |
| GD3 | OPRM1 |
| GD3 | GABRA1 |
| GD3 | BCL2 |
| GD3 | JUN |
| GD3 | CASP3 |
| GD3 | CASP8 |
| GD3 | PRKCA |
| GD3 | TGFB1 |
| GD3 | PON1 |
| GD3 | MAP2 |
| GD2 | PTGS1 |
| GD2 | AR |
| GD2 | PTGS2 |
| GD2 | PRSS1 |
| GD2 | PGR |
| GD2 | CHRM1 |
| GD2 | ACHE |
| GD2 | SLC6A2 |
| GD2 | GABRA1 |
| GD2 | RELA |
| GD2 | IKBKB |
| GD2 | AKT1 |
| GD2 | BCL2 |
| GD2 | TNF |
| GD2 | JUN |
| GD2 | CASP3 |
| GD2 | MAPK8 |
| GD2 | XDH |
| GD2 | MMP1 |
| GD2 | PPARG |
| GD2 | HMOX1 |
| GD2 | CYP3A4 |
| GD2 | CYP1A2 |
| GD2 | CYP1A1 |
| GD2 | ICAM1 |
| GD2 | SELE |
| GD2 | VCAM1 |
| GD2 | NR1I2 |
| GD2 | CYP1B1 |
| GD2 | ALOX5 |
| GD2 | GSTP1 |
| GD2 | AHR |
| GD2 | NR1I3 |
| GD2 | INSR |
| GD2 | PPP3CA |
| GD2 | GSTM1 |
| GD2 | GSTM2 |
| GD2 | AKR1C3 |
| DX4 | PGR |
| DX4 | NR3C2 |
| DX4 | IGHG1 |
| DX4 | RXRA |
| DX4 | NCOA1 |
| DX4 | PTGS1 |
| DX4 | PTGS2 |
| DX4 | ADRA2A |
| DX4 | SLC6A2 |
| DX4 | SLC6A3 |
| DX4 | ADRB2 |
| DX4 | AKR1B1 |
| DX4 | PLAU |
| DX4 | MAOB |
| DX4 | MAOA |
| DX4 | CHRM3 |
| DX4 | CHRM1 |
| DX4 | ADRB1 |
| DX4 | SCN5A |
| DX4 | ADRA1A |
| DX4 | GABRA1 |
| GD3 | HMGCR |
| GD3 | CYP51A1 |
| GD3 | AR |
| GD3 | NPC1L1 |
| GD3 | NR1H3 |
| GD3 | CYP17A1 |
| GD3 | RORC |
| GD3 | CYP19A1 |
| GD3 | ESR2 |
| GD3 | ESR1 |
| GD3 | SHBG |
| GD3 | SREBF2 |
| GD3 | CYP2C19 |
| GD3 | SLC6A2 |
| GD3 | BCHE |
| GD3 | RORA |
| GD3 | PTPN1 |
| GD3 | SERPINA6 |
| GD3 | G6PD |
| GD3 | NR1I3 |
| GD3 | CHRM2 |
| GD3 | ACHE |
| GD3 | VDR |
| GD3 | CES2 |
| GD3 | NR1H2 |
| GD3 | CDC25A |
| GD3 | PTGER1 |
| GD3 | PTGER2 |
| GD3 | HSD11B1 |
| GD3 | DHCR7 |
| GD3 | GLRA1 |
| GD3 | PPARD |
| GD3 | SQLE |
| GD3 | PTPN6 |
| GD3 | FDFT1 |
| GD3 | NOS2 |
| GD3 | NR3C1 |
| GD3 | CDC25B |
| GD3 | SHH |
| GD3 | UGT2B7 |
| GD3 | HSD11B2 |
| GD3 | DRD2 |
| GD3 | POLB |
| GD2 | NOX4 |
| GD2 | AKR1B1 |
| GD2 | TYR |
| GD2 | FLT3 |
| GD2 | CA2 |
| GD2 | CA7 |
| GD2 | HSD17B2 |
| GD2 | ABCC1 |
| GD2 | HSD17B1 |
| GD2 | CA12 |
| GD2 | ESRRA |
| GD2 | ABCB1 |
| GD2 | ABCG2 |
| GD2 | ADORA1 |
| GD2 | CA4 |
| GD2 | MAOA |
| GD2 | GLO1 |
| GD2 | SYK |
| GD2 | GSK3B |
| GD2 | MMP9 |
| GD2 | MMP2 |
| GD2 | ALOX15 |
| GD2 | ALOX12 |
| GD2 | PTPRS |
| GD2 | ADORA2A |
| GD2 | CDK5R1 |
| GD2 | CCNB3 |
| GD2 | ARG1 |
| GD2 | GPR35 |
| GD2 | ESR2 |
| GD2 | DAPK1 |
| GD2 | MPG |
| GD2 | SLC22A12 |
| GD2 | TTR |
| GD2 | AKR1B10 |
| GD2 | TNKS2 |
| GD2 | TNKS |
| GD2 | CDK6 |
| GD2 | CDK2 |
| GD2 | CYP19A1 |
| GD2 | CSNK2A1 |
| GD2 | EGFR |
| GD2 | AVPR2 |
| GD2 | IGF1R |
| GD2 | F2 |
| GD2 | PIM1 |
| GD2 | AURKB |
| GD2 | DRD4 |
| GD2 | MPO |
| GD2 | PIK3R1 |
| GD2 | PYGL |
| GD2 | CA1 |
| GD2 | SRC |
| GD2 | PTK2 |
| GD2 | KDR |
| GD2 | MMP13 |
| GD2 | MMP3 |
| GD2 | CA3 |
| GD2 | PLK1 |
| GD2 | CA6 |
| GD2 | CDK1 |
| GD2 | PKN1 |
| GD2 | CA14 |
| GD2 | CA9 |
| GD2 | MET |
| GD2 | NEK2 |
| GD2 | CXCR1 |
| GD2 | CAMK2B |
| GD2 | ALK |
| GD2 | NEK6 |
| GD2 | PLA2G1B |
| GD2 | CA5A |
| GD2 | BACE1 |
| GD2 | AXL |
| GD2 | NUAK1 |
| GD2 | AKR1C2 |
| GD2 | AKR1C1 |
| GD2 | AKR1C4 |
| GD2 | CA13 |
| GD2 | AKR1A1 |
| GD2 | APP |
| GD2 | PARP1 |
| GD2 | MMP12 |
| GD2 | CD38 |
| GD2 | TOP1 |
| GD2 | ESR1 |
| GD2 | CFTR |
| GD2 | PFKFB3 |
| GD2 | AMY1A |
| GD2 | GRK6 |
| GD2 | TERT |
| GD2 | MAPT |
| DX3 | PRKCD |
| DX3 | PTPN1 |
| DX3 | PRKCA |
| DX3 | PTPN2 |
| DX3 | AR |
| DX3 | CTSK |
| DX3 | CTSS |
| DX3 | CTSL |
| DX3 | CTSB |
| DX3 | FKBP1A |
| DX3 | PDE10A |
| DX3 | GRM2 |
| DX3 | CCND1 |
| DX3 | CDK1 |
| DX3 | CCNE1 |
| DX3 | MAPK14 |
| DX3 | TSPO |
| DX3 | PTGER1 |
| DX3 | GABRB3 |
| DX3 | GABRB3 |
| DX3 | GABRB3 |
| DX3 | GABRA2 |
| DX3 | PDE5A |
| DX3 | BCL2 |
| DX3 | GPBAR1 |
| DX3 | FNTA |
| DX3 | PREP |
| DX3 | MAPK11 |
| DX3 | ACE |
| DX3 | P2RX7 |
| DX3 | GABRG2 |
| DX3 | ELOVL6 |
| DX3 | KCNA5 |
| DX3 | PGGT1B |
| DX3 | TNFRSF1A |
| DX3 | SCN10A |
| DX3 | CPB1 |
| DX3 | MEN1 |
| DX3 | TRPC6 |
| DX3 | TRPC3 |
| DX3 | ELANE |
| DX3 | TRPV4 |
| DX3 | HCRTR2 |
| DX3 | TRPV1 |
| DX3 | MAP3K8 |
| DX3 | HCRTR1 |
| DX3 | TRPA1 |
| DX3 | SLC2A1 |
| DX3 | GRM5 |
| DX3 | ADAM17 |
| DX3 | CMA1 |
| DX3 | PTGDR2 |
| DX3 | SLC2A3 |
| DX3 | SLC2A2 |
| DX3 | ADORA1 |
| DX3 | ADORA2A |
| DX3 | ADORA2B |
| DX3 | MMP9 |
| DX3 | MMP2 |
| DX3 | MMP7 |
| DX3 | RORC |
| DX3 | PABPC1 |
| DX3 | ABL1 |
| DX3 | CCND3 |
| DX3 | CAPN1 |
| DX3 | SCN2A |
| DX3 | PTGES |
| DX3 | PTGFR |
| DX3 | AGTR1 |
| DX3 | TERT |
| DX3 | CTSV |
| DX3 | KCNN4 |
| DX3 | VCP |
| DX3 | MTNR1A |
| DX3 | MTNR1B |
| DX3 | CASP3 |
| DX3 | SLC6A9 |
| DX3 | PDE4A |
| DX3 | PDE4B |
| DX3 | ALOX15 |
| DX3 | PDE4C |
| DX3 | BAD |
| DX3 | PIK3CA |
| DX3 | MCL1 |
| DX3 | BCL2L1 |
| DX3 | BCL2L2 |
| DX3 | BCL2L10 |
| DX3 | BCL2A1 |
| DX3 | CRHR1 |
| DX3 | CDK5R1 |
| DX3 | MAPK8 |
| DX3 | CCR1 |
| DX3 | APP |
| DX3 | PTAFR |
| DX3 | SCARB1 |
| DX3 | TDO2 |
| DX3 | GRM4 |
| DX3 | NMBR |
| DX3 | TACR3 |
| DX3 | IDO1 |
| DX4 | CYP51A1 |
| DX4 | HMGCR |
| DX4 | NR1H3 |
| DX4 | NPC1L1 |
| DX4 | CYP17A1 |
| DX4 | AR |
| DX4 | SREBF2 |
| DX4 | CYP19A1 |
| DX4 | RORC |
| DX4 | ESR1 |
| DX4 | ESR2 |
| DX4 | SHBG |
| DX4 | SERPINA6 |
| DX4 | PTPN1 |
| DX4 | CHRM2 |
| DX4 | RORA |
| DX4 | BCHE |
| DX4 | SLC6A4 |
| DX4 | ACHE |
| DX4 | CYP2C19 |
| DX4 | G6PD |
| DX4 | CDC25A |
| DX4 | CES2 |
| DX4 | NR1H2 |
| DX4 | VDR |
| DX4 | PTGER1 |
| DX4 | PTGER2 |
| DX4 | HSD11B1 |
| DX4 | PPARD |
| DX4 | GLRA1 |
| DX4 | SQLE |
| DX4 | NR1I3 |
| DX4 | DHCR7 |
| DX4 | PTPN6 |
| DX4 | NR3C1 |
| DX4 | TBXAS1 |
| DX4 | GCGR |
| DX4 | NOS2 |
| DX4 | HSD11B2 |
| DX4 | POLB |
| GD1 | ABCB1 |
| GD1 | ABCC1 |
| GD1 | ABCG2 |
| GD1 | ACACA |
| GD1 | ACHE |
| GD1 | ACPP |
| GD1 | ADORA1 |
| GD1 | ADORA2A |
| GD1 | ADRB2 |
| GD1 | AHR |
| GD1 | AKR1A1 |
| GD1 | AKR1B1 |
| GD1 | AKR1B10 |
| GD1 | AKR1C1 |
| GD1 | AKR1C2 |
| GD1 | AKR1C3 |
| GD1 | AKR1C4 |
| GD1 | AKT1 |
| GD1 | ALK |
| GD1 | ALOX12 |
| GD1 | ALOX15 |
| GD1 | ALOX5 |
| GD1 | APEX1 |
| GD1 | APP |
| GD1 | AR |
| GD1 | ARG1 |
| GD1 | AURKB |
| GD1 | AVPR2 |
| GD1 | AXL |
| GD1 | BACE1 |
| GD1 | BCL2 |
| GD1 | BCL2L1 |
| GD1 | BIRC5 |
| GD1 | CA1 |
| GD1 | CA12 |
| GD1 | CA13 |
| GD1 | CA14 |
| GD1 | CA2 |
| GD1 | CA3 |
| GD1 | CA4 |
| GD1 | CA5A |
| GD1 | CA6 |
| GD1 | CA7 |
| GD1 | CA9 |
| GD1 | CAMK2B |
| GD1 | CASP3 |
| GD1 | CASP8 |
| GD1 | CCL2 |
| GD1 | CCNB3 |
| GD1 | CCND1 |
| GD1 | CD38 |
| GD1 | CD40LG |
| GD1 | CDK1 |
| GD1 | CDK2 |
| GD1 | CDK5R1 |
| GD1 | CDK6 |
| GD1 | CDKN1A |
| GD1 | CHEK2 |
| GD1 | CHUK |
| GD1 | COL1A1 |
| GD1 | COL3A1 |
| GD1 | CRP |
| GD1 | CSNK2A1 |
| GD1 | CTSD |
| GD1 | CXCL10 |
| GD1 | CXCL8 |
| GD1 | CXCR1 |
| GD1 | CYP19A1 |
| GD1 | CYP1A1 |
| GD1 | CYP1A2 |
| GD1 | CYP1B1 |
| GD1 | CYP3A4 |
| GD1 | DAPK1 |
| GD1 | DRD4 |
| GD1 | EGF |
| GD1 | EGFR |
| GD1 | EIF6 |
| GD1 | ERBB2 |
| GD1 | ESR2 |
| GD1 | ESRRA |
| GD1 | F2 |
| GD1 | F3 |
| GD1 | F7 |
| GD1 | FLT3 |
| GD1 | FOS |
| GD1 | GABRA1 |
| GD1 | GJA1 |
| GD1 | GLO1 |
| GD1 | GPR35 |
| GD1 | GSK3B |
| GD1 | GSTM1 |
| GD1 | GSTM2 |
| GD1 | GSTP1 |
| GD1 | HIF1A |
| GD1 | HMOX1 |
| GD1 | HSD17B1 |
| GD1 | HSD17B2 |
| GD1 | HSF1 |
| GD1 | HSPA5 |
| GD1 | HSPB1 |
| GD1 | ICAM1 |
| GD1 | IFNG |
| GD1 | IGF1R |
| GD1 | IGFBP3 |
| GD1 | IL10 |
| GD1 | IL1A |
| GD1 | IL1B |
| GD1 | IL2 |
| GD1 | IL6 |
| GD1 | INSR |
| GD1 | JUN |
| GD1 | KCNH2 |
| GD1 | KDM4E |
| GD1 | KDR |
| GD1 | MAOA |
| GD1 | MAOB |
| GD1 | MAPK1 |
| GD1 | MAPT |
| GD1 | MET |
| GD1 | MGAM |
| GD1 | MMP1 |
| GD1 | MMP12 |
| GD1 | MMP13 |
| GD1 | MMP2 |
| GD1 | MMP3 |
| GD1 | MMP9 |
| GD1 | MPG |
| GD1 | MPO |
| GD1 | MYC |
| GD1 | MYLK |
| GD1 | NCF1 |
| GD1 | NEK2 |
| GD1 | NEK6 |
| GD1 | NFKBIA |
| GD1 | NOS3 |
| GD1 | NOX4 |
| GD1 | NPEPPS |
| GD1 | NQO1 |
| GD1 | NR1I2 |
| GD1 | NR1I3 |
| GD1 | NUAK1 |
| GD1 | ODC1 |
| GD1 | PARP1 |
| GD1 | PIK3CG |
| GD1 | PIK3R1 |
| GD1 | PIM1 |
| GD1 | PKN1 |
| GD1 | PLA2G1B |
| GD1 | PLAT |
| GD1 | PLAU |
| GD1 | PLK1 |
| GD1 | PON1 |
| GD1 | POR |
| GD1 | PPARA |
| GD1 | PPARD |
| GD1 | PPARG |
| GD1 | PRKCA |
| GD1 | PRKCB |
| GD1 | PRSS1 |
| GD1 | PTGER3 |
| GD1 | PTGS1 |
| GD1 | PTGS2 |
| GD1 | PTK2 |
| GD1 | PTPRS |
| GD1 | PYGL |
| GD1 | RAF1 |
| GD1 | RB1 |
| GD1 | RELA |
| GD1 | RXRA |
| GD1 | SCN5A |
| GD1 | SELE |
| GD1 | SERPINE1 |
| GD1 | SLC22A12 |
| GD1 | SOD1 |
| GD1 | SRC |
| GD1 | SULT1E1 |
| GD1 | SYK |
| GD1 | TERT |
| GD1 | TGFB1 |
| GD1 | THBD |
| GD1 | TNF |
| GD1 | TNKS |
| GD1 | TNKS2 |
| GD1 | TOP1 |
| GD1 | TOP2A |
| GD1 | TP53 |
| GD1 | TTR |
| GD1 | TYR |
| GD1 | VCAM1 |
| GD1 | VEGFA |
| GD1 | XDH |
| GZ1 | ABAT |
| GD1 | ABCB1 |
| GD2 | ABCB1 |
| GZ1 | ABCC1 |
| GD1 | ABCC1 |
| GD2 | ABCC1 |
| GZ2 | ABCC1 |
| GD1 | ABCG2 |
| GD2 | ABCG2 |
| GZ2 | ABCG2 |
| GZ1 | ABCG2 |
| GZ3 | ABL1 |
| GD1 | ACACA |
| GZ3 | ACE |
| GD2 | ACHE |
| GD1 | ACHE |
| GD3 | ACHE |
| GD1 | ACPP |
| GZ3 | ADAM17 |
| GZ3 | ADORA1 |
| GD1 | ADORA1 |
| GD2 | ADORA1 |
| GZ2 | ADORA1 |
| GZ1 | ADORA1 |
| GZ3 | ADORA2A |
| GD1 | ADORA2A |
| GD2 | ADORA2A |
| GZ3 | ADORA2B |
| GZ2 | ADORA3 |
| GZ1 | ADORA3 |
| GD3 | ADRA1A |
| GD3 | ADRB2 |
| GD1 | ADRB2 |
| GZ3 | AGTR1 |
| GD2 | AHR |
| GD1 | AHR |
| GD1 | AKR1A1 |
| GD2 | AKR1A1 |
| GD1 | AKR1B1 |
| GZ4 | AKR1B1 |
| GD2 | AKR1B1 |
| GZ2 | AKR1B1 |
| GZ1 | AKR1B1 |
| GD1 | AKR1B10 |
| GD2 | AKR1B10 |
| GZ1 | AKR1C1 |
| GD1 | AKR1C1 |
| GD2 | AKR1C1 |
| GD1 | AKR1C2 |
| GD2 | AKR1C2 |
| GD2 | AKR1C3 |
| GD1 | AKR1C3 |
| GZ2 | AKR1C3 |
| GZ1 | AKR1C3 |
| GD1 | AKR1C4 |
| GD2 | AKR1C4 |
| GD2 | AKT1 |
| GZ1 | AKT1 |
| GD1 | AKT1 |
| GZ4 | AKT1 |
| GD1 | ALK |
| GD2 | ALK |
| GD1 | ALOX12 |
| GD2 | ALOX12 |
| GZ2 | ALOX12 |
| GZ1 | ALOX12 |
| GZ3 | ALOX15 |
| GD1 | ALOX15 |
| GD2 | ALOX15 |
| GD2 | ALOX5 |
| GD1 | ALOX5 |
| GZ4 | ALOX5 |
| GD2 | AMY1A |
| GD1 | APEX1 |
| GZ3 | APP |
| GD1 | APP |
| GD2 | APP |
| GZ2 | APP |
| GZ1 | APP |
| GZ4 | AR |
| GD2 | AR |
| GD1 | AR |
| GD3 | AR |
| GZ3 | AR |
| GD1 | ARG1 |
| GD2 | ARG1 |
| GZ4 | AURKA |
| GZ2 | AURKA |
| GZ1 | AURKA |
| GZ4 | AURKB |
| GD1 | AURKB |
| GD2 | AURKB |
| GD1 | AVPR2 |
| GD2 | AVPR2 |
| GD1 | AXL |
| GD2 | AXL |
| GZ4 | BACE1 |
| GD1 | BACE1 |
| GD2 | BACE1 |
| GZ2 | BACE1 |
| GZ1 | BACE1 |
| GZ3 | BAD |
| GD3 | BCHE |
| GZ2 | BCHE |
| GZ1 | BCHE |
| GD3 | BCL2 |
| GD2 | BCL2 |
| GZ1 | BCL2 |
| GD1 | BCL2 |
| GZ3 | BCL2 |
| GZ2 | BCL2 |
| GZ3 | BCL2A1 |
| GD1 | BCL2L1 |
| GZ3 | BCL2L1 |
| GZ2 | BCL2L1 |
| GZ1 | BCL2L1 |
| GZ3 | BCL2L10 |
| GZ3 | BCL2L2 |
| GD1 | BIRC5 |
| GZ4 | BRAF |
| GZ4 | CA1 |
| GD1 | CA1 |
| GD2 | CA1 |
| GZ2 | CA1 |
| GZ1 | CA1 |
| GZ4 | CA12 |
| GD1 | CA12 |
| GD2 | CA12 |
| GZ2 | CA12 |
| GZ1 | CA12 |
| GZ4 | CA13 |
| GD1 | CA13 |
| GD2 | CA13 |
| GZ2 | CA13 |
| GZ1 | CA13 |
| GZ4 | CA14 |
| GD1 | CA14 |
| GD2 | CA14 |
| GZ4 | CA2 |
| GD1 | CA2 |
| GD2 | CA2 |
| GZ2 | CA2 |
| GZ1 | CA2 |
| GD1 | CA3 |
| GD2 | CA3 |
| GZ2 | CA3 |
| GZ1 | CA3 |
| GZ4 | CA4 |
| GD1 | CA4 |
| GD2 | CA4 |
| GZ2 | CA4 |
| GZ1 | CA4 |
| GZ4 | CA5A |
| GD1 | CA5A |
| GD2 | CA5A |
| GZ2 | CA5A |
| GZ1 | CA5A |
| GZ4 | CA5B |
| GZ2 | CA5B |
| GZ1 | CA5B |
| GZ4 | CA6 |
| GD1 | CA6 |
| GD2 | CA6 |
| GZ2 | CA6 |
| GZ1 | CA6 |
| GZ4 | CA7 |
| GD1 | CA7 |
| GD2 | CA7 |
| GZ2 | CA7 |
| GZ1 | CA7 |
| GZ4 | CA9 |
| GD1 | CA9 |
| GD2 | CA9 |
| GZ2 | CA9 |
| GZ1 | CA9 |
| GD1 | CAMK2B |
| GD2 | CAMK2B |
| GZ3 | CAPN1 |
| GD3 | CASP3 |
| GD2 | CASP3 |
| GZ1 | CASP3 |
| GD1 | CASP3 |
| GZ3 | CASP3 |
| GD3 | CASP8 |
| GD1 | CASP8 |
| GZ1 | CAT |
| GZ4 | CBR1 |
| GZ2 | CBR1 |
| GZ1 | CBR1 |
| GD1 | CCL2 |
| GD1 | CCNB3 |
| GD2 | CCNB3 |
| GD1 | CCND1 |
| GZ4 | CCND1 |
| GZ3 | CCND1 |
| GZ3 | CCND3 |
| GZ3 | CCNE1 |
| GZ2 | CCNE1 |
| GZ1 | CCNE1 |
| GZ2 | CCNE1 |
| GZ1 | CCNE1 |
| GZ3 | CCR1 |
| GD1 | CD38 |
| GD2 | CD38 |
| GD1 | CD40LG |
| GD3 | CDC25A |
| GD3 | CDC25B |
| GD1 | CDK1 |
| GD2 | CDK1 |
| GZ3 | CDK1 |
| GD1 | CDK2 |
| GD2 | CDK2 |
| GZ4 | CDK2 |
| GZ2 | CDK4 |
| GZ1 | CDK4 |
| GZ4 | CDK5R1 |
| GZ3 | CDK5R1 |
| GD1 | CDK5R1 |
| GD2 | CDK5R1 |
| GZ2 | CDK5R1 |
| GZ1 | CDK5R1 |
| GD1 | CDK6 |
| GD2 | CDK6 |
| GZ4 | CDKN1A |
| GD1 | CDKN1A |
| GZ1 | CES1 |
| GZ2 | CES1 |
| GD3 | CES2 |
| GZ4 | CES2 |
| GZ2 | CES2 |
| GZ1 | CES2 |
| GD2 | CFTR |
| GD1 | CHEK2 |
| GD3 | CHRM1 |
| GD2 | CHRM1 |
| GD3 | CHRM2 |
| GZ2 | CHRNA7 |
| GZ1 | CHRNA7 |
| GD1 | CHUK |
| GZ2 | CLK1 |
| GZ1 | CLK1 |
| GZ3 | CMA1 |
| GD1 | COL1A1 |
| GD1 | COL3A1 |
| GZ3 | CPB1 |
| GZ3 | CRHR1 |
| GD1 | CRP |
| GZ4 | CSNK2A1 |
| GD1 | CSNK2A1 |
| GD2 | CSNK2A1 |
| GZ3 | CTSB |
| GZ2 | CTSB |
| GZ1 | CTSB |
| GD1 | CTSD |
| GZ3 | CTSK |
| GZ3 | CTSL |
| GZ3 | CTSS |
| GZ3 | CTSV |
| GD1 | CXCL10 |
| GZ4 | CXCL8 |
| GD1 | CXCL8 |
| GD1 | CXCR1 |
| GD2 | CXCR1 |
| GD3 | CYP17A1 |
| GD3 | CYP19A1 |
| GD1 | CYP19A1 |
| GD2 | CYP19A1 |
| GZ2 | CYP19A1 |
| GZ1 | CYP19A1 |
| GD2 | CYP1A1 |
| GD1 | CYP1A1 |
| GD2 | CYP1A2 |
| GD1 | CYP1A2 |
| GD2 | CYP1B1 |
| GD1 | CYP1B1 |
| GZ2 | CYP1B1 |
| GZ1 | CYP1B1 |
| GD3 | CYP2C19 |
| GZ2 | CYP2C9 |
| GZ1 | CYP2C9 |
| GD2 | CYP3A4 |
| GD1 | CYP3A4 |
| GZ2 | CYP3A4 |
| GZ1 | CYP3A4 |
| GD3 | CYP51A1 |
| GZ4 | DAO |
| GD1 | DAPK1 |
| GD2 | DAPK1 |
| GD3 | DHCR7 |
| GD3 | DRD2 |
| GD1 | DRD4 |
| GD2 | DRD4 |
| GZ2 | DYRK1A |
| GZ1 | DYRK1A |
| GZ2 | DYRK1B |
| GZ1 | DYRK1B |
| GZ2 | EDNRA |
| GZ1 | EDNRA |
| GD1 | EGF |
| GD1 | EGFR |
| GZ4 | EGFR |
| GD2 | EGFR |
| GD1 | EIF6 |
| GZ3 | ELANE |
| GZ3 | ELOVL6 |
| GZ4 | EPHB4 |
| GD1 | ERBB2 |
| GZ4 | ERBB2 |
| GZ2 | ERN1 |
| GZ1 | ERN1 |
| GZ4 | ESR1 |
| GZ2 | ESR1 |
| GZ1 | ESR1 |
| GZ5 | ESR1 |
| GD3 | ESR1 |
| GD2 | ESR1 |
| GD3 | ESR2 |
| GZ4 | ESR2 |
| GD1 | ESR2 |
| GD2 | ESR2 |
| GZ2 | ESR2 |
| GZ1 | ESR2 |
| GD1 | ESRRA |
| GD2 | ESRRA |
| GZ2 | ESRRA |
| GZ1 | ESRRA |
| GZ2 | ESRRB |
| GZ1 | ESRRB |
| GD1 | F2 |
| GD2 | F2 |
| GD1 | F3 |
| GZ2 | F3 |
| GZ1 | F3 |
| GD2 | F7 |
| GD1 | F7 |
| GZ1 | FASN |
| GZ5 | FASN |
| GD3 | FDFT1 |
| GZ2 | FGFR1 |
| GZ1 | FGFR1 |
| GZ4 | FGR |
| GZ3 | FKBP1A |
| GD1 | FLT3 |
| GD2 | FLT3 |
| GZ4 | FLT4 |
| GZ3 | FNTA |
| GD1 | FOS |
| GD3 | G6PD |
| GD3 | GABRA1 |
| GD2 | GABRA1 |
| GD1 | GABRA1 |
| GZ3 | GABRA2 |
| GZ3 | GABRB3 |
| GZ3 | GABRB3 |
| GZ3 | GABRB3 |
| GZ3 | GABRG2 |
| GD1 | GJA1 |
| GD1 | GLO1 |
| GD2 | GLO1 |
| GD3 | GLRA1 |
| GZ1 | GOT1 |
| GZ3 | GPBAR1 |
| GZ4 | GPR35 |
| GD1 | GPR35 |
| GD2 | GPR35 |
| GD2 | GRK6 |
| GZ3 | GRM2 |
| GZ3 | GRM4 |
| GZ3 | GRM5 |
| GZ2 | GRM5 |
| GZ1 | GRM5 |
| GZ4 | GSK3B |
| GD1 | GSK3B |
| GD2 | GSK3B |
| GZ2 | GSK3B |
| GZ1 | GSK3B |
| GZ1 | GSR |
| GZ4 | GSR |
| GZ4 | GSTA1 |
| GZ4 | GSTA2 |
| GZ4 | GSTM1 |
| GD2 | GSTM1 |
| GD1 | GSTM1 |
| GZ4 | GSTM2 |
| GD2 | GSTM2 |
| GD1 | GSTM2 |
| GZ4 | GSTP1 |
| GD2 | GSTP1 |
| GZ1 | GSTP1 |
| GD1 | GSTP1 |
| GZ3 | HCRTR1 |
| GZ3 | HCRTR2 |
| GD1 | HIF1A |
| GZ1 | HMGCR |
| GD3 | HMGCR |
| GD2 | HMOX1 |
| GD1 | HMOX1 |
| GZ2 | HNF4A |
| GZ1 | HNF4A |
| GD3 | HSD11B1 |
| GD3 | HSD11B2 |
| GD1 | HSD17B1 |
| GD2 | HSD17B1 |
| GZ2 | HSD17B1 |
| GZ1 | HSD17B1 |
| GZ2 | HSD17B14 |
| GZ1 | HSD17B14 |
| GD1 | HSD17B2 |
| GD2 | HSD17B2 |
| GZ2 | HSD17B2 |
| GZ1 | HSD17B2 |
| GZ4 | HSD17B3 |
| GD1 | HSF1 |
| GZ4 | HSPA1A |
| GD1 | HSPA5 |
| GD1 | HSPB1 |
| GD2 | ICAM1 |
| GD1 | ICAM1 |
| GZ3 | IDO1 |
| GD1 | IFNG |
| GZ4 | IGF1R |
| GD1 | IGF1R |
| GD2 | IGF1R |
| GZ2 | IGF1R |
| GZ1 | IGF1R |
| GD1 | IGFBP3 |
| GZ2 | IGFBP3 |
| GZ1 | IGFBP3 |
| GD2 | IKBKB |
| GD1 | IL10 |
| GD1 | IL1A |
| GD1 | IL1B |
| GD1 | IL2 |
| GD1 | IL6 |
| GD2 | INSR |
| GD1 | INSR |
| GZ4 | INSR |
| GZ2 | INSR |
| GZ1 | INSR |
| GD3 | JUN |
| GD2 | JUN |
| GD1 | JUN |
| GZ3 | KCNA5 |
| GD3 | KCNH2 |
| GD1 | KCNH2 |
| GZ3 | KCNN4 |
| GD1 | KDM4E |
| GZ4 | KDR |
| GD1 | KDR |
| GD2 | KDR |
| GZ2 | KDR |
| GZ1 | KDR |
| GZ2 | KIT |
| GZ1 | KIT |
| GZ2 | KLK1 |
| GZ1 | KLK1 |
| GZ2 | KLK2 |
| GZ1 | KLK2 |
| GZ2 | LCK |
| GZ1 | LCK |
| GZ1 | LDLR |
| GZ4 | LYN |
| GZ4 | MAOA |
| GD1 | MAOA |
| GD2 | MAOA |
| GD1 | MAOB |
| GZ2 | MAOB |
| GZ1 | MAOB |
| GD3 | MAP2 |
| GZ4 | MAP3K8 |
| GZ3 | MAP3K8 |
| GZ1 | MAPK1 |
| GD1 | MAPK1 |
| GZ3 | MAPK11 |
| GZ3 | MAPK14 |
| GZ1 | MAPK3 |
| GD2 | MAPK8 |
| GZ3 | MAPK8 |
| GD1 | MAPT |
| GD2 | MAPT |
| GZ3 | MCL1 |
| GZ3 | MEN1 |
| GZ4 | MET |
| GD1 | MET |
| GD2 | MET |
| GZ2 | MET |
| GZ1 | MET |
| GD1 | MGAM |
| GD2 | MMP1 |
| GD1 | MMP1 |
| GD1 | MMP12 |
| GD2 | MMP12 |
| GZ2 | MMP12 |
| GZ1 | MMP12 |
| GD1 | MMP13 |
| GD2 | MMP13 |
| GZ2 | MMP13 |
| GZ1 | MMP13 |
| GZ4 | MMP2 |
| GD1 | MMP2 |
| GZ3 | MMP2 |
| GD2 | MMP2 |
| GZ2 | MMP2 |
| GZ1 | MMP2 |
| GD1 | MMP3 |
| GD2 | MMP3 |
| GZ2 | MMP3 |
| GZ1 | MMP3 |
| GZ3 | MMP7 |
| GZ4 | MMP9 |
| GD1 | MMP9 |
| GZ3 | MMP9 |
| GD2 | MMP9 |
| GZ2 | MMP9 |
| GZ1 | MMP9 |
| GD1 | MPG |
| GD2 | MPG |
| GD1 | MPO |
| GD2 | MPO |
| GZ3 | MTNR1A |
| GZ3 | MTNR1B |
| GZ1 | MTTP |
| GD1 | MYC |
| GD1 | MYLK |
| GD1 | NCF1 |
| GD1 | NEK2 |
| GD2 | NEK2 |
| GD1 | NEK6 |
| GD2 | NEK6 |
| GZ4 | NFKBIA |
| GD1 | NFKBIA |
| GZ3 | NMBR |
| GD2 | NOS2 |
| GD3 | NOS2 |
| GD1 | NOS3 |
| GD1 | NOX4 |
| GD2 | NOX4 |
| GZ2 | NOX4 |
| GZ1 | NOX4 |
| GD3 | NPC1L1 |
| GD1 | NPEPPS |
| GD1 | NQO1 |
| GZ2 | NQO2 |
| GZ1 | NQO2 |
| GD3 | NR1H2 |
| GD3 | NR1H3 |
| GD2 | NR1I2 |
| GD1 | NR1I2 |
| GD2 | NR1I3 |
| GD1 | NR1I3 |
| GD3 | NR1I3 |
| GZ2 | NR3C1 |
| GD3 | NR3C1 |
| GZ2 | NR3C2 |
| GZ4 | NUAK1 |
| GD1 | NUAK1 |
| GD2 | NUAK1 |
| GD1 | ODC1 |
| GD3 | OPRM1 |
| GZ3 | P2RX7 |
| GZ3 | PABPC1 |
| GD1 | PARP1 |
| GD2 | PARP1 |
| GZ3 | PDE10A |
| GZ3 | PDE4A |
| GZ3 | PDE4B |
| GZ3 | PDE4C |
| GZ3 | PDE5A |
| GZ4 | PDGFRB |
| GD2 | PFKFB3 |
| GZ2 | PGF |
| GZ1 | PGF |
| GZ3 | PGGT1B |
| GZ4 | PGR |
| GZ2 | PGR |
| GD3 | PGR |
| GD2 | PGR |
| GZ3 | PIK3CA |
| GZ2 | PIK3CA |
| GZ1 | PIK3CA |
| GZ2 | PIK3CB |
| GZ1 | PIK3CB |
| GD1 | PIK3CG |
| GD1 | PIK3R1 |
| GD2 | PIK3R1 |
| GD1 | PIM1 |
| GD2 | PIM1 |
| GD1 | PKN1 |
| GD2 | PKN1 |
| GZ2 | PLA2G10 |
| GZ1 | PLA2G10 |
| GD1 | PLA2G1B |
| GD2 | PLA2G1B |
| GZ2 | PLA2G1B |
| GZ1 | PLA2G1B |
| GZ2 | PLA2G2A |
| GZ1 | PLA2G2A |
| GZ2 | PLA2G5 |
| GZ1 | PLA2G5 |
| GD1 | PLAT |
| GD1 | PLAU |
| GZ4 | PLK1 |
| GD1 | PLK1 |
| GD2 | PLK1 |
| GZ4 | PLK4 |
| GD3 | POLB |
| GZ2 | POLB |
| GZ1 | POLB |
| GD3 | PON1 |
| GD1 | PON1 |
| GD1 | POR |
| GZ1 | PPARA |
| GD1 | PPARA |
| GD1 | PPARD |
| GD3 | PPARD |
| GD2 | PPARG |
| GZ1 | PPARG |
| GZ5 | PPARG |
| GD1 | PPARG |
| GZ2 | PPARG |
| GD2 | PPP3CA |
| GZ3 | PREP |
| GD3 | PRKCA |
| GD1 | PRKCA |
| GZ3 | PRKCA |
| GZ4 | PRKCB |
| GD1 | PRKCB |
| GZ3 | PRKCD |
| GD2 | PRSS1 |
| GD1 | PRSS1 |
| GZ3 | PTAFR |
| GZ3 | PTGDR2 |
| GD3 | PTGER1 |
| GZ3 | PTGER1 |
| GZ2 | PTGER1 |
| GZ1 | PTGER1 |
| GD3 | PTGER2 |
| GZ2 | PTGER2 |
| GZ1 | PTGER2 |
| GD1 | PTGER3 |
| GZ2 | PTGER3 |
| GZ1 | PTGER3 |
| GZ3 | PTGES |
| GZ3 | PTGFR |
| GZ2 | PTGS1 |
| GZ6 | PTGS1 |
| GD3 | PTGS1 |
| GD2 | PTGS1 |
| GZ1 | PTGS1 |
| GZ5 | PTGS1 |
| GD1 | PTGS1 |
| GZ2 | PTGS2 |
| GZ6 | PTGS2 |
| GD3 | PTGS2 |
| GD2 | PTGS2 |
| GZ1 | PTGS2 |
| GZ5 | PTGS2 |
| GD1 | PTGS2 |
| GZ4 | PTGS2 |
| GZ4 | PTK2 |
| GD1 | PTK2 |
| GD2 | PTK2 |
| GD3 | PTPN1 |
| GZ4 | PTPN1 |
| GZ3 | PTPN1 |
| GZ3 | PTPN2 |
| GD3 | PTPN6 |
| GD1 | PTPRS |
| GD2 | PTPRS |
| GD1 | PYGL |
| GD2 | PYGL |
| GD1 | RAF1 |
| GD1 | RB1 |
| GZ4 | RELA |
| GD2 | RELA |
| GZ1 | RELA |
| GD1 | RELA |
| GD3 | RORA |
| GD3 | RORC |
| GZ3 | RORC |
| GD1 | RXRA |
| GZ2 | RXRA |
| GZ1 | RXRA |
| GZ3 | SCARB1 |
| GZ3 | SCN10A |
| GZ3 | SCN2A |
| GZ3 | SCN5A |
| GD3 | SCN5A |
| GD1 | SCN5A |
| GD2 | SELE |
| GD1 | SELE |
| GD3 | SERPINA6 |
| GD1 | SERPINE1 |
| GZ2 | SERPINE1 |
| GZ1 | SERPINE1 |
| GD3 | SHBG |
| GZ2 | SHBG |
| GZ1 | SHBG |
| GD3 | SHH |
| GZ2 | SIRT2 |
| GZ1 | SIRT2 |
| GD1 | SLC22A12 |
| GD2 | SLC22A12 |
| GZ3 | SLC2A1 |
| GZ3 | SLC2A2 |
| GZ3 | SLC2A3 |
| GZ2 | SLC5A2 |
| GZ1 | SLC5A2 |
| GD2 | SLC6A2 |
| GD3 | SLC6A2 |
| GD3 | SLC6A4 |
| GZ3 | SLC6A9 |
| GZ4 | SNCA |
| GZ2 | SNCA |
| GZ1 | SNCA |
| GZ1 | SOAT1 |
| GZ1 | SOAT2 |
| GZ1 | SOD1 |
| GD1 | SOD1 |
| GD3 | SQLE |
| GZ4 | SQLE |
| GZ4 | SRC |
| GD1 | SRC |
| GD2 | SRC |
| GZ2 | SRC |
| GZ1 | SRC |
| GZ1 | SREBF1 |
| GD3 | SREBF2 |
| GD1 | SULT1E1 |
| GD1 | SYK |
| GD2 | SYK |
| GZ2 | SYK |
| GZ1 | SYK |
| GZ3 | TACR3 |
| GZ2 | TAS2R31 |
| GZ1 | TAS2R31 |
| GZ3 | TDO2 |
| GZ4 | TEK |
| GZ3 | TERT |
| GD1 | TERT |
| GD2 | TERT |
| GD3 | TGFB1 |
| GD1 | TGFB1 |
| GD1 | THBD |
| GD2 | TNF |
| GD1 | TNF |
| GZ3 | TNFRSF1A |
| GD1 | TNKS |
| GD2 | TNKS |
| GD1 | TNKS2 |
| GD2 | TNKS2 |
| GZ4 | TNNC1 |
| GD1 | TOP1 |
| GD2 | TOP1 |
| GD1 | TOP2A |
| GD1 | TP53 |
| GZ3 | TRPA1 |
| GZ3 | TRPC3 |
| GZ3 | TRPC6 |
| GZ3 | TRPV1 |
| GZ3 | TRPV4 |
| GZ3 | TSPO |
| GD1 | TTR |
| GD2 | TTR |
| GD1 | TYR |
| GD2 | TYR |
| GD3 | UGT2B7 |
| GD2 | VCAM1 |
| GD1 | VCAM1 |
| GZ3 | VCP |
| GZ2 | VCP |
| GZ1 | VCP |
| GD3 | VDR |
| GZ4 | VEGFA |
| GD1 | VEGFA |
| GZ2 | VEGFA |
| GZ1 | VEGFA |
| GZ2 | WEE1 |
| GZ1 | WEE1 |
| GD2 | XDH |
| GD1 | XDH |
| GZ4 | XDH |
| GZ2 | YWHAG |
| GZ1 | YWHAG |

|  | Table S4. Herbs-ingredients–target genes network statistics. | | | | | | | | | | | | | | | | | | |
| --- | --- | --- | --- | --- | --- | --- | --- | --- | --- | --- | --- | --- | --- | --- | --- | --- | --- | --- | --- |
| name | AverageShortestPathLength | BetweennessCentrality | ClosenessCentrality | ClusteringCoefficient | Degree | degree.layout | Eccentricity | IsSingleNode | NeighborhoodConnectivity | NumberOfDirectedEdges | NumberOfUndirectedEdges | PartnerOfMultiEdgedNodePairs | Radiality | selected | SelfLoops | shared name | Stress | TopologicalCoefficient | Type |
| GD1 | 2.191756272 | 0.289631152 | 0.45625511 | 0 | 384 | 384 | 4 | FALSE | 4.740932642 | 0 | 384 | 191 | 0.993825097 | FALSE | 0 | GD1 | 5872320 | 0.055013715 | GuangDing |
| GD2 | 2.396057348 | 0.124280521 | 0.417352281 | 0 | 264 | 264 | 4 | FALSE | 5.753731343 | 0 | 264 | 130 | 0.992766542 | FALSE | 0 | GD2 | 3411672 | 0.067910448 | GuangDing |
| GD3 | 2.639784946 | 0.05609351 | 0.378818737 | 0 | 128 | 128 | 4 | FALSE | 9.484848485 | 0 | 128 | 62 | 0.991503705 | FALSE | 0 | GD3 | 1375308 | 0.121212121 | GuangDing |
| GZ1 | 2.467741935 | 0.120731796 | 0.405228758 | 0 | 119 | 119 | 4 | FALSE | 5.288135593 | 0 | 119 | 1 | 0.99239512 | FALSE | 0 | GZ1 | 3342604 | 0.064971751 | Guangzao |
| DX3 | 2.582437276 | 0.120383679 | 0.38723109 | 0 | 105 | 105 | 4 | FALSE | 4.631067961 | 0 | 105 | 1 | 0.991800843 | FALSE | 0 | DX3 | 3571610 | 0.074103428 | Dingxiang |
| GZ3 | 2.596774194 | 0.101619148 | 0.385093168 | 0 | 102 | 102 | 4 | FALSE | 3.86 | 0 | 102 | 1 | 0.991726559 | FALSE | 0 | GZ3 | 2733134 | 0.059583333 | Guangzao |
| GZ2 | 2.517921147 | 0.079393515 | 0.397153025 | 0 | 102 | 102 | 4 | FALSE | 5.871287129 | 0 | 102 | 1 | 0.992135124 | FALSE | 0 | GZ2 | 2637464 | 0.070598364 | Guangzao |
| BP2 | 2.621863799 | 0.179272668 | 0.381408066 | 0 | 101 | 101 | 4 | FALSE | 3.12244898 | 0 | 101 | 3 | 0.991596561 | FALSE | 0 | BP2 | 3328894 | 0.049359279 | Bingpian |
| BP1 | 2.525089606 | 0.173624023 | 0.39602555 | 0 | 101 | 101 | 4 | FALSE | 4.386138614 | 0 | 101 | 0 | 0.992097981 | FALSE | 0 | BP1 | 2919392 | 0.050539382 | Bingpian |
| GZ4 | 2.625448029 | 0.06979111 | 0.380887372 | 0 | 73 | 73 | 4 | FALSE | 5.808219178 | 0 | 73 | 0 | 0.991577989 | FALSE | 0 | GZ4 | 1549992 | 0.071764465 | Guangzao |
| PTGS2 | 2.283154122 | 0.073825937 | 0.437990581 | 0 | 64 | 64 | 4 | FALSE | 24.04918033 | 0 | 64 | 3 | 0.993351533 | FALSE | 0 | PTGS2 | 2069810 | 0.060338168 | gene |
| DX4 | 2.654121864 | 0.04784291 | 0.376772451 | 0 | 62 | 62 | 4 | FALSE | 10.43548387 | 0 | 62 | 0 | 0.99142942 | FALSE | 0 | DX4 | 1203970 | 0.134792627 | Dingxiang |
| Danshen | 3.336917563 | 0.015922584 | 0.299677766 | 0 | 57 | 57 | 4 | FALSE | 11.01754386 | 0 | 57 | 0 | 0.987891619 | FALSE | 0 | Danshen | 1041904 | 0.108886346 | drug |
| DS8 | 2.711469534 | 0.030627468 | 0.368803701 | 0 | 46 | 46 | 4 | FALSE | 8.152173913 | 0 | 46 | 0 | 0.991132282 | FALSE | 0 | DS8 | 721272 | 0.102173913 | Danshen |
| SCN5A | 2.326164875 | 0.044045209 | 0.429892142 | 0 | 38 | 38 | 4 | FALSE | 30.11111111 | 0 | 38 | 2 | 0.993128679 | FALSE | 0 | SCN5A | 1252990 | 0.073699015 | gene |
| PTGS1 | 2.444444444 | 0.025017133 | 0.409090909 | 0 | 37 | 37 | 4 | FALSE | 34.88235294 | 0 | 37 | 3 | 0.992515832 | FALSE | 0 | PTGS1 | 605350 | 0.093083387 | gene |
| ADRB2 | 2.537634409 | 0.014554428 | 0.394067797 | 0 | 35 | 35 | 4 | FALSE | 26.6969697 | 0 | 35 | 2 | 0.992032982 | FALSE | 0 | ADRB2 | 529958 | 0.07580227 | gene |
| CHRM1 | 2.702508961 | 0.012247731 | 0.370026525 | 0 | 35 | 35 | 4 | FALSE | 24.63636364 | 0 | 35 | 2 | 0.99117871 | FALSE | 0 | CHRM1 | 498088 | 0.080670183 | gene |
| NCOA1 | 3.401433692 | 0.003378669 | 0.293993678 | 0 | 34 | 34 | 4 | FALSE | 12.91176471 | 0 | 34 | 0 | 0.987557338 | FALSE | 0 | NCOA1 | 241414 | 0.122801698 | gene |
| ACHE | 2.813620072 | 0.012613498 | 0.355414013 | 0 | 33 | 33 | 4 | FALSE | 25.9 | 0 | 33 | 3 | 0.990603005 | FALSE | 0 | ACHE | 496620 | 0.093962264 | gene |
| DS55 | 2.758064516 | 0.021015576 | 0.362573099 | 0 | 32 | 32 | 3 | FALSE | 15.46875 | 0 | 32 | 0 | 0.990890857 | FALSE | 0 | DS55 | 439910 | 0.203785211 | Danshen |
| OPRM1 | 3.401433692 | 0.001747061 | 0.293993678 | 0 | 30 | 30 | 4 | FALSE | 17.24137931 | 0 | 30 | 1 | 0.987557338 | FALSE | 0 | OPRM1 | 87664 | 0.159229209 | gene |
| AR | 2.336917563 | 0.029585501 | 0.42791411 | 0 | 30 | 30 | 4 | FALSE | 41.7037037 | 0 | 30 | 3 | 0.993072966 | FALSE | 0 | AR | 881038 | 0.101505496 | gene |
| RXRA | 2.698924731 | 0.010450103 | 0.370517928 | 0 | 29 | 29 | 4 | FALSE | 29.92857143 | 0 | 29 | 1 | 0.991197281 | FALSE | 0 | RXRA | 350446 | 0.096751075 | gene |
| ESR1 | 2.784946237 | 0.010699129 | 0.359073359 | 0 | 28 | 28 | 4 | FALSE | 31.73076923 | 0 | 28 | 2 | 0.990751574 | FALSE | 0 | ESR1 | 340954 | 0.110941405 | gene |
| ADRA1A | 3.379928315 | 0.001854831 | 0.295864263 | 0 | 28 | 28 | 4 | FALSE | 19.92592593 | 0 | 28 | 1 | 0.987668765 | FALSE | 0 | ADRA1A | 70342 | 0.172053872 | gene |
| CHRM3 | 3.068100358 | 0.006985485 | 0.325934579 | 0 | 27 | 27 | 4 | FALSE | 23 | 0 | 27 | 0 | 0.989284454 | FALSE | 0 | CHRM3 | 315108 | 0.111675127 | gene |
| DS33 | 2.790322581 | 0.005006497 | 0.358381503 | 0 | 23 | 23 | 3 | FALSE | 24.56521739 | 0 | 23 | 0 | 0.990723717 | FALSE | 0 | DS33 | 269200 | 0.33190447 | Danshen |
| GABRA1 | 2.863799283 | 0.005747732 | 0.349186483 | 0 | 23 | 23 | 4 | FALSE | 36.45 | 0 | 23 | 3 | 0.990343009 | FALSE | 0 | GABRA1 | 154192 | 0.135823755 | gene |
| DS51 | 2.79390681 | 0.00948772 | 0.357921745 | 0 | 22 | 22 | 3 | FALSE | 20.72727273 | 0 | 22 | 0 | 0.990705146 | FALSE | 0 | DS51 | 179528 | 0.277848912 | Danshen |
| DS29 | 2.797491039 | 0.00850974 | 0.357463165 | 0 | 21 | 21 | 3 | FALSE | 22.0952381 | 0 | 21 | 0 | 0.990686575 | FALSE | 0 | DS29 | 264110 | 0.29711603 | Danshen |
| PRSS1 | 3.003584229 | 0.006392283 | 0.332935561 | 0 | 21 | 21 | 4 | FALSE | 28.31578947 | 0 | 21 | 2 | 0.989618735 | FALSE | 0 | PRSS1 | 277630 | 0.122492329 | gene |
| OPRD1 | 3.58781362 | 5.09E-04 | 0.278721279 | 0 | 20 | 20 | 4 | FALSE | 16.4 | 0 | 20 | 0 | 0.986591639 | FALSE | 0 | OPRD1 | 12754 | 0.261016949 | gene |
| DS43 | 2.804659498 | 0.003189135 | 0.356549521 | 0 | 19 | 19 | 3 | FALSE | 26.57894737 | 0 | 19 | 0 | 0.990649433 | FALSE | 0 | DS43 | 194102 | 0.360266864 | Danshen |
| DS37 | 2.804659498 | 0.002258076 | 0.356549521 | 0 | 19 | 19 | 3 | FALSE | 29.26315789 | 0 | 19 | 0 | 0.990649433 | FALSE | 0 | DS37 | 189382 | 0.398072646 | Danshen |
| DS13 | 2.804659498 | 0.003030653 | 0.356549521 | 0 | 19 | 19 | 3 | FALSE | 27.36842105 | 0 | 19 | 0 | 0.990649433 | FALSE | 0 | DS13 | 174982 | 0.371386212 | Danshen |
| DS39 | 2.808243728 | 0.002428068 | 0.356094448 | 0 | 18 | 18 | 3 | FALSE | 28.83333333 | 0 | 18 | 0 | 0.990630862 | FALSE | 0 | DS39 | 203856 | 0.392018779 | Danshen |
| DS38 | 2.808243728 | 0.002132812 | 0.356094448 | 0 | 18 | 18 | 3 | FALSE | 27.66666667 | 0 | 18 | 0 | 0.990630862 | FALSE | 0 | DS38 | 185166 | 0.375586854 | Danshen |
| DS30 | 2.808243728 | 0.002504651 | 0.356094448 | 0 | 18 | 18 | 3 | FALSE | 26.77777778 | 0 | 18 | 0 | 0.990630862 | FALSE | 0 | DS30 | 196710 | 0.363067293 | Danshen |
| DS10 | 2.808243728 | 0.002705765 | 0.356094448 | 0 | 18 | 18 | 3 | FALSE | 27.11111111 | 0 | 18 | 0 | 0.990630862 | FALSE | 0 | DS10 | 168094 | 0.367762128 | Danshen |
| DS1 | 2.808243728 | 0.002548141 | 0.356094448 | 0 | 18 | 18 | 3 | FALSE | 26.94444444 | 0 | 18 | 0 | 0.990630862 | FALSE | 0 | DS1 | 141748 | 0.36541471 | Danshen |
| DS32 | 2.815412186 | 0.00162081 | 0.355187778 | 0 | 16 | 16 | 3 | FALSE | 31.25 | 0 | 16 | 0 | 0.990593719 | FALSE | 0 | DS32 | 160256 | 0.426056338 | Danshen |
| DS17 | 2.815412186 | 0.00131533 | 0.355187778 | 0 | 16 | 16 | 3 | FALSE | 30.0625 | 0 | 16 | 0 | 0.990593719 | FALSE | 0 | DS17 | 128170 | 0.409330986 | Danshen |
| DS42 | 2.818996416 | 0.003101099 | 0.354736173 | 0 | 15 | 15 | 3 | FALSE | 29.86666667 | 0 | 15 | 0 | 0.990575148 | FALSE | 0 | DS42 | 128672 | 0.40657277 | Danshen |
| DS35 | 2.818996416 | 0.001568491 | 0.354736173 | 0 | 15 | 15 | 3 | FALSE | 29.86666667 | 0 | 15 | 0 | 0.990575148 | FALSE | 0 | DS35 | 111234 | 0.40657277 | Danshen |
| DS31 | 2.818996416 | 0.001610096 | 0.354736173 | 0 | 15 | 15 | 3 | FALSE | 30.4 | 0 | 15 | 0 | 0.990575148 | FALSE | 0 | DS31 | 114174 | 0.414084507 | Danshen |
| IGHG1 | 3.566308244 | 4.30E-04 | 0.28040201 | 0 | 15 | 15 | 4 | FALSE | 17.46666667 | 0 | 15 | 0 | 0.986703066 | FALSE | 0 | IGHG1 | 22410 | 0.235238095 | gene |
| GSK3B | 2.559139785 | 0.010108605 | 0.390756303 | 0 | 15 | 15 | 4 | FALSE | 64.30769231 | 0 | 15 | 2 | 0.991921556 | FALSE | 0 | GSK3B | 322730 | 0.179341905 | gene |
| PGR | 2.691756272 | 0.011166527 | 0.37150466 | 0 | 15 | 15 | 4 | FALSE | 45.23076923 | 0 | 15 | 2 | 0.991234423 | FALSE | 0 | PGR | 357846 | 0.139970789 | gene |
| ESR2 | 2.505376344 | 0.009859501 | 0.399141631 | 0 | 15 | 15 | 4 | FALSE | 74.83333333 | 0 | 15 | 3 | 0.992200123 | FALSE | 0 | ESR2 | 288320 | 0.200090334 | gene |
| DS6 | 2.822580645 | 0.001040379 | 0.354285714 | 0 | 14 | 14 | 3 | FALSE | 32.85714286 | 0 | 14 | 0 | 0.990556577 | FALSE | 0 | DS6 | 122560 | 0.448692153 | Danshen |
| DS45 | 2.826164875 | 8.70E-04 | 0.353836398 | 0 | 13 | 13 | 3 | FALSE | 33.15384615 | 0 | 13 | 0 | 0.990538006 | FALSE | 0 | DS45 | 94210 | 0.452871073 | Danshen |
| DS21 | 2.826164875 | 8.43E-04 | 0.353836398 | 0 | 13 | 13 | 3 | FALSE | 33.92307692 | 0 | 13 | 0 | 0.990538006 | FALSE | 0 | DS21 | 97554 | 0.463705309 | Danshen |
| DS9 | 2.826164875 | 8.94E-04 | 0.353836398 | 0 | 13 | 13 | 3 | FALSE | 34.15384615 | 0 | 13 | 0 | 0.990538006 | FALSE | 0 | DS9 | 101822 | 0.46695558 | Danshen |
| SLC6A4 | 3.268817204 | 0.00228922 | 0.305921053 | 0 | 13 | 13 | 4 | FALSE | 32.83333333 | 0 | 13 | 1 | 0.98824447 | FALSE | 0 | SLC6A4 | 50288 | 0.204059829 | gene |
| CASP3 | 2.17562724 | 0.023283492 | 0.459637562 | 0 | 13 | 13 | 4 | FALSE | 99.1 | 0 | 13 | 3 | 0.993908667 | FALSE | 0 | CASP3 | 562818 | 0.21187905 | gene |
| DS16 | 2.987455197 | 5.87E-04 | 0.334733053 | 0 | 12 | 12 | 5 | FALSE | 32.16666667 | 0 | 12 | 0 | 0.989702305 | FALSE | 0 | DS16 | 59718 | 0.451690821 | Danshen |
| MMP2 | 2.204301075 | 0.021516178 | 0.453658537 | 0 | 12 | 12 | 4 | FALSE | 106.7 | 0 | 12 | 2 | 0.993760098 | FALSE | 0 | MMP2 | 583690 | 0.232307692 | gene |
| DX2 | 2.844086022 | 7.69E-04 | 0.351606805 | 0 | 11 | 11 | 4 | FALSE | 28.54545455 | 0 | 11 | 0 | 0.99044515 | FALSE | 0 | DX2 | 71056 | 0.405080214 | Dingxiang |
| DS56 | 2.833333333 | 6.73E-04 | 0.352941176 | 0 | 11 | 11 | 3 | FALSE | 34.45454545 | 0 | 11 | 0 | 0.990500864 | FALSE | 0 | DS56 | 77988 | 0.471190781 | Danshen |
| BCL2 | 2.498207885 | 0.009403266 | 0.400286944 | 0 | 11 | 11 | 4 | FALSE | 105.875 | 0 | 11 | 3 | 0.992237265 | FALSE | 0 | BCL2 | 331322 | 0.279666667 | gene |
| DS34 | 2.836917563 | 0.00101656 | 0.352495262 | 0 | 11 | 11 | 4 | FALSE | 31.90909091 | 0 | 11 | 0 | 0.990482292 | FALSE | 0 | DS34 | 70372 | 0.441558442 | Danshen |
| MMP9 | 2.519713262 | 0.010336085 | 0.396870555 | 0 | 11 | 11 | 4 | FALSE | 100 | 0 | 11 | 2 | 0.992125838 | FALSE | 0 | MMP9 | 354576 | 0.269021739 | gene |
| DS5 | 2.833333333 | 8.47E-04 | 0.352941176 | 0 | 11 | 11 | 3 | FALSE | 32.54545455 | 0 | 11 | 0 | 0.990500864 | FALSE | 0 | DS5 | 71788 | 0.444302177 | Danshen |
| NR3C1 | 3.035842294 | 0.004111407 | 0.329397875 | 0 | 11 | 11 | 4 | FALSE | 37.6 | 0 | 11 | 1 | 0.989451594 | FALSE | 0 | NR3C1 | 182120 | 0.164125561 | gene |
| NOS2 | 2.924731183 | 0.002827372 | 0.341911765 | 0 | 11 | 11 | 4 | FALSE | 45.5 | 0 | 11 | 1 | 0.9900273 | FALSE | 0 | NOS2 | 79536 | 0.17519685 | gene |
| CYP19A1 | 2.76702509 | 0.004024904 | 0.361398964 | 0 | 10 | 10 | 4 | FALSE | 96.85714286 | 0 | 10 | 3 | 0.99084443 | FALSE | 0 | CYP19A1 | 149332 | 0.318462269 | gene |
| DS44 | 2.836917563 | 6.37E-04 | 0.352495262 | 0 | 10 | 10 | 3 | FALSE | 35.9 | 0 | 10 | 0 | 0.990482292 | FALSE | 0 | DS44 | 73822 | 0.491549296 | Danshen |
| DS11 | 2.836917563 | 6.07E-04 | 0.352495262 | 0 | 10 | 10 | 3 | FALSE | 35.2 | 0 | 10 | 0 | 0.990482292 | FALSE | 0 | DS11 | 72456 | 0.481690141 | Danshen |
| APP | 2.584229391 | 0.007998087 | 0.386962552 | 0 | 10 | 10 | 4 | FALSE | 102 | 0 | 10 | 2 | 0.991791558 | FALSE | 0 | APP | 294302 | 0.287749288 | gene |
| Guangzao | 2.46953405 | 0.008327721 | 0.404934688 | 0 | 9 | 9 | 4 | FALSE | 88.22222222 | 0 | 9 | 0 | 0.992385834 | FALSE | 0 | Guangzao | 255460 | 0.228330425 | drug |
| CDK5R1 | 2.577060932 | 0.007811062 | 0.388038943 | 0 | 9 | 9 | 4 | FALSE | 117.4285714 | 0 | 9 | 2 | 0.9918287 | FALSE | 0 | CDK5R1 | 275304 | 0.32889427 | gene |
| DS25 | 2.998207885 | 3.49E-04 | 0.333532576 | 0 | 9 | 9 | 5 | FALSE | 35.11111111 | 0 | 9 | 0 | 0.989646591 | FALSE | 0 | DS25 | 40994 | 0.494363929 | Danshen |
| RELA | 2.792114695 | 0.002459811 | 0.358151476 | 0 | 9 | 9 | 4 | FALSE | 88.14285714 | 0 | 9 | 2 | 0.990714432 | FALSE | 0 | RELA | 80452 | 0.296404276 | gene |
| AKR1B1 | 2.738351254 | 0.003580914 | 0.365183246 | 0 | 9 | 9 | 4 | FALSE | 97.71428571 | 0 | 9 | 2 | 0.990992999 | FALSE | 0 | AKR1B1 | 141642 | 0.312991216 | gene |
| INSR | 2.598566308 | 0.006238314 | 0.384827586 | 0 | 9 | 9 | 4 | FALSE | 109 | 0 | 9 | 2 | 0.991717273 | FALSE | 0 | INSR | 195482 | 0.310344828 | gene |
| CCNA2 | 3.401433692 | 0.002635437 | 0.293993678 | 0 | 9 | 9 | 4 | FALSE | 25 | 0 | 9 | 0 | 0.987557338 | FALSE | 0 | CCNA2 | 82964 | 0.196721311 | gene |
| MET | 2.598566308 | 0.006238314 | 0.384827586 | 0 | 9 | 9 | 4 | FALSE | 109 | 0 | 9 | 2 | 0.991717273 | FALSE | 0 | MET | 195482 | 0.310344828 | gene |
| ADORA1 | 2.451612903 | 0.010769145 | 0.407894737 | 0 | 9 | 9 | 4 | FALSE | 121 | 0 | 9 | 2 | 0.99247869 | FALSE | 0 | ADORA1 | 341186 | 0.30848329 | gene |
| PPARG | 2.652329749 | 0.004835164 | 0.377027027 | 0 | 9 | 9 | 4 | FALSE | 99.85714286 | 0 | 9 | 2 | 0.991438706 | FALSE | 0 | PPARG | 143324 | 0.296868297 | gene |
| DS57 | 2.847670251 | 5.38E-04 | 0.351164254 | 0 | 8 | 8 | 4 | FALSE | 36.25 | 0 | 8 | 0 | 0.990426579 | FALSE | 0 | DS57 | 60912 | 0.503571429 | Danshen |
| DS36 | 3.163082437 | 2.97E-04 | 0.316147309 | 0 | 8 | 8 | 5 | FALSE | 34 | 0 | 8 | 0 | 0.988792319 | FALSE | 0 | DS36 | 37532 | 0.507692308 | Danshen |
| JUN | 2.924731183 | 0.00144524 | 0.341911765 | 0 | 8 | 8 | 4 | FALSE | 94.2 | 0 | 8 | 3 | 0.9900273 | FALSE | 0 | JUN | 41700 | 0.35984556 | gene |
| BCL2L1 | 2.616487455 | 0.006387865 | 0.382191781 | 0 | 8 | 8 | 4 | FALSE | 97.42857143 | 0 | 8 | 1 | 0.991624417 | FALSE | 0 | BCL2L1 | 225088 | 0.281132861 | gene |
| SLC6A3 | 3.580645161 | 1.53E-04 | 0.279279279 | 0 | 8 | 8 | 4 | FALSE | 24.5 | 0 | 8 | 0 | 0.986628782 | FALSE | 0 | SLC6A3 | 4364 | 0.321917808 | gene |
| CCND1 | 2.541218638 | 0.008882833 | 0.393511989 | 0 | 8 | 8 | 4 | FALSE | 90.57142857 | 0 | 8 | 1 | 0.992014411 | FALSE | 0 | CCND1 | 236834 | 0.246075353 | gene |
| KDR | 2.623655914 | 0.005340481 | 0.381147541 | 0 | 8 | 8 | 4 | FALSE | 119.5 | 0 | 8 | 2 | 0.991587275 | FALSE | 0 | KDR | 168234 | 0.346491228 | gene |
| SRC | 2.623655914 | 0.005340481 | 0.381147541 | 0 | 8 | 8 | 4 | FALSE | 119.5 | 0 | 8 | 2 | 0.991587275 | FALSE | 0 | SRC | 168234 | 0.346491228 | gene |
| PTGER1 | 2.759856631 | 0.007201835 | 0.362337662 | 0 | 8 | 8 | 5 | FALSE | 93 | 0 | 8 | 1 | 0.990881572 | FALSE | 0 | PTGER1 | 227754 | 0.302631579 | gene |
| BACE1 | 2.620071685 | 0.004751218 | 0.381668947 | 0 | 8 | 8 | 4 | FALSE | 120 | 0 | 8 | 2 | 0.991605846 | FALSE | 0 | BACE1 | 143350 | 0.346938776 | gene |
| Dingxiang | 2.659498208 | 0.005073205 | 0.376010782 | 0 | 7 | 7 | 4 | FALSE | 81.85714286 | 0 | 7 | 0 | 0.991401564 | FALSE | 0 | Dingxiang | 161002 | 0.244281398 | drug |
| CA13 | 2.853046595 | 0.001292916 | 0.350502513 | 0 | 7 | 7 | 4 | FALSE | 123.8 | 0 | 7 | 2 | 0.990398722 | FALSE | 0 | CA13 | 67228 | 0.440143369 | gene |
| CA5A | 2.853046595 | 0.001292916 | 0.350502513 | 0 | 7 | 7 | 4 | FALSE | 123.8 | 0 | 7 | 2 | 0.990398722 | FALSE | 0 | CA5A | 67228 | 0.440143369 | gene |
| CA9 | 2.853046595 | 0.001292916 | 0.350502513 | 0 | 7 | 7 | 4 | FALSE | 123.8 | 0 | 7 | 2 | 0.990398722 | FALSE | 0 | CA9 | 67228 | 0.440143369 | gene |
| CA6 | 2.853046595 | 0.001292916 | 0.350502513 | 0 | 7 | 7 | 4 | FALSE | 123.8 | 0 | 7 | 2 | 0.990398722 | FALSE | 0 | CA6 | 67228 | 0.440143369 | gene |
| CA1 | 2.853046595 | 0.001292916 | 0.350502513 | 0 | 7 | 7 | 4 | FALSE | 123.8 | 0 | 7 | 2 | 0.990398722 | FALSE | 0 | CA1 | 67228 | 0.440143369 | gene |
| IGF1R | 2.853046595 | 0.001292916 | 0.350502513 | 0 | 7 | 7 | 4 | FALSE | 123.8 | 0 | 7 | 2 | 0.990398722 | FALSE | 0 | IGF1R | 67228 | 0.440143369 | gene |
| CA4 | 2.853046595 | 0.001292916 | 0.350502513 | 0 | 7 | 7 | 4 | FALSE | 123.8 | 0 | 7 | 2 | 0.990398722 | FALSE | 0 | CA4 | 67228 | 0.440143369 | gene |
| CA12 | 2.853046595 | 0.001292916 | 0.350502513 | 0 | 7 | 7 | 4 | FALSE | 123.8 | 0 | 7 | 2 | 0.990398722 | FALSE | 0 | CA12 | 67228 | 0.440143369 | gene |
| CA7 | 2.853046595 | 0.001292916 | 0.350502513 | 0 | 7 | 7 | 4 | FALSE | 123.8 | 0 | 7 | 2 | 0.990398722 | FALSE | 0 | CA7 | 67228 | 0.440143369 | gene |
| CA2 | 2.853046595 | 0.001292916 | 0.350502513 | 0 | 7 | 7 | 4 | FALSE | 123.8 | 0 | 7 | 2 | 0.990398722 | FALSE | 0 | CA2 | 67228 | 0.440143369 | gene |
| NR1I3 | 2.953405018 | 0.001360871 | 0.338592233 | 0 | 7 | 7 | 4 | FALSE | 113.75 | 0 | 7 | 3 | 0.98987873 | FALSE | 0 | NR1I3 | 45422 | 0.447420635 | gene |
| CYP3A4 | 2.878136201 | 0.001366164 | 0.347447073 | 0 | 7 | 7 | 4 | FALSE | 115.6 | 0 | 7 | 2 | 0.990268724 | FALSE | 0 | CYP3A4 | 62154 | 0.421323529 | gene |
| DS50 | 3.234767025 | 2.46E-04 | 0.309141274 | 0 | 7 | 7 | 5 | FALSE | 33.28571429 | 0 | 7 | 0 | 0.988420896 | FALSE | 0 | DS50 | 19408 | 0.481876333 | Danshen |
| DS46 | 3.005376344 | 2.80E-04 | 0.33273703 | 0 | 7 | 7 | 5 | FALSE | 33.42857143 | 0 | 7 | 0 | 0.989609449 | FALSE | 0 | DS46 | 31908 | 0.469979296 | Danshen |
| DS28 | 3.005376344 | 5.99E-04 | 0.33273703 | 0 | 7 | 7 | 5 | FALSE | 31.85714286 | 0 | 7 | 0 | 0.989609449 | FALSE | 0 | DS28 | 33082 | 0.447204969 | Danshen |
| KCNH2 | 2.989247312 | 7.97E-04 | 0.334532374 | 0 | 7 | 7 | 4 | FALSE | 59 | 0 | 7 | 2 | 0.989693019 | FALSE | 0 | KCNH2 | 18696 | 0.2406639 | gene |
| DS27 | 2.85125448 | 4.05E-04 | 0.350722816 | 0 | 7 | 7 | 4 | FALSE | 36.42857143 | 0 | 7 | 0 | 0.990408008 | FALSE | 0 | DS27 | 38608 | 0.506122449 | Danshen |
| DS14 | 2.85483871 | 4.45E-04 | 0.350282486 | 0 | 7 | 7 | 4 | FALSE | 22 | 0 | 7 | 0 | 0.990389437 | FALSE | 0 | DS14 | 34454 | 0.304347826 | Danshen |
| GSTP1 | 2.835125448 | 0.001256085 | 0.352718078 | 0 | 7 | 7 | 4 | FALSE | 112.8 | 0 | 7 | 2 | 0.990491578 | FALSE | 0 | GSTP1 | 53576 | 0.393661972 | gene |
| AKT1 | 2.835125448 | 0.001256085 | 0.352718078 | 0 | 7 | 7 | 4 | FALSE | 112.8 | 0 | 7 | 2 | 0.990491578 | FALSE | 0 | AKT1 | 53576 | 0.393661972 | gene |
| CDK1 | 2.630824373 | 0.00659327 | 0.380108992 | 0 | 7 | 7 | 5 | FALSE | 125.6 | 0 | 7 | 2 | 0.991550133 | FALSE | 0 | CDK1 | 186366 | 0.364327485 | gene |
| EGFR | 2.774193548 | 0.003604766 | 0.360465116 | 0 | 7 | 7 | 4 | FALSE | 108.8 | 0 | 7 | 2 | 0.990807287 | FALSE | 0 | EGFR | 96348 | 0.358139535 | gene |
| SYK | 2.677419355 | 0.004301612 | 0.373493976 | 0 | 7 | 7 | 4 | FALSE | 128.8 | 0 | 7 | 2 | 0.991308708 | FALSE | 0 | SYK | 135942 | 0.389634146 | gene |
| MMP13 | 2.677419355 | 0.004301612 | 0.373493976 | 0 | 7 | 7 | 4 | FALSE | 128.8 | 0 | 7 | 2 | 0.991308708 | FALSE | 0 | MMP13 | 135942 | 0.389634146 | gene |
| PIK3CA | 2.670250896 | 0.008820018 | 0.374496644 | 0 | 7 | 7 | 5 | FALSE | 103.5 | 0 | 7 | 1 | 0.99134585 | FALSE | 0 | PIK3CA | 210830 | 0.310606061 | gene |
| TNF | 2.802867384 | 0.002877986 | 0.356777494 | 0 | 7 | 7 | 4 | FALSE | 99 | 0 | 7 | 2 | 0.990658718 | FALSE | 0 | TNF | 64078 | 0.33447099 | gene |
| AURKB | 2.548387097 | 0.007409774 | 0.392405063 | 0 | 7 | 7 | 4 | FALSE | 119.8 | 0 | 7 | 2 | 0.991977269 | FALSE | 0 | AURKB | 147920 | 0.326373626 | gene |
| MMP3 | 2.681003584 | 0.003609293 | 0.372994652 | 0 | 7 | 7 | 4 | FALSE | 129.4 | 0 | 7 | 2 | 0.991290137 | FALSE | 0 | MMP3 | 113762 | 0.39266055 | gene |
| TERT | 2.602150538 | 0.006602406 | 0.384297521 | 0 | 7 | 7 | 4 | FALSE | 126.2 | 0 | 7 | 2 | 0.991698702 | FALSE | 0 | TERT | 185268 | 0.358739255 | gene |
| CES2 | 2.896057348 | 0.003441102 | 0.34529703 | 0 | 7 | 7 | 5 | FALSE | 86.83333333 | 0 | 7 | 1 | 0.990175869 | FALSE | 0 | CES2 | 94082 | 0.321473159 | gene |
| PLA2G1B | 2.681003584 | 0.003609293 | 0.372994652 | 0 | 7 | 7 | 4 | FALSE | 129.4 | 0 | 7 | 2 | 0.991290137 | FALSE | 0 | PLA2G1B | 113762 | 0.39266055 | gene |
| PTPN1 | 2.856630824 | 0.005608085 | 0.350062735 | 0 | 7 | 7 | 5 | FALSE | 84.16666667 | 0 | 7 | 1 | 0.990380151 | FALSE | 0 | PTPN1 | 146604 | 0.299160671 | gene |
| GZ5 | 3.03046595 | 3.28E-04 | 0.329982259 | 0 | 6 | 6 | 5 | FALSE | 23.33333333 | 0 | 6 | 0 | 0.989479451 | FALSE | 0 | GZ5 | 10686 | 0.354497354 | Guangzao |
| GABRB3 | 3.566308244 | 3.45E-07 | 0.28040201 | 0 | 6 | 6 | 5 | FALSE | 101.5 | 0 | 6 | 2 | 0.986703066 | FALSE | 0 | GABRB3 | 30 | 0.975728155 | gene |
| CCNE1 | 3.111111111 | 0.002008531 | 0.321428571 | 0 | 6 | 6 | 5 | FALSE | 105.5 | 0 | 6 | 2 | 0.9890616 | FALSE | 0 | CCNE1 | 73308 | 0.5 | gene |
| MMP12 | 2.917562724 | 7.64E-04 | 0.342751843 | 0 | 6 | 6 | 4 | FALSE | 136.5 | 0 | 6 | 2 | 0.990064442 | FALSE | 0 | MMP12 | 46848 | 0.517175573 | gene |
| CA3 | 2.917562724 | 7.64E-04 | 0.342751843 | 0 | 6 | 6 | 4 | FALSE | 136.5 | 0 | 6 | 2 | 0.990064442 | FALSE | 0 | CA3 | 46848 | 0.517175573 | gene |
| ADORA2A | 2.831541219 | 0.003423872 | 0.353164557 | 0 | 6 | 6 | 5 | FALSE | 132.5 | 0 | 6 | 2 | 0.990510149 | FALSE | 0 | ADORA2A | 110414 | 0.458188153 | gene |
| ALOX12 | 2.917562724 | 7.64E-04 | 0.342751843 | 0 | 6 | 6 | 4 | FALSE | 136.5 | 0 | 6 | 2 | 0.990064442 | FALSE | 0 | ALOX12 | 46848 | 0.517175573 | gene |
| ALOX15 | 2.831541219 | 0.003423872 | 0.353164557 | 0 | 6 | 6 | 5 | FALSE | 132.5 | 0 | 6 | 2 | 0.990510149 | FALSE | 0 | ALOX15 | 110414 | 0.458188153 | gene |
| ABCG2 | 2.917562724 | 7.64E-04 | 0.342751843 | 0 | 6 | 6 | 4 | FALSE | 136.5 | 0 | 6 | 2 | 0.990064442 | FALSE | 0 | ABCG2 | 46848 | 0.517175573 | gene |
| ESRRA | 2.917562724 | 7.64E-04 | 0.342751843 | 0 | 6 | 6 | 4 | FALSE | 136.5 | 0 | 6 | 2 | 0.990064442 | FALSE | 0 | ESRRA | 46848 | 0.517175573 | gene |
| HSD17B1 | 2.917562724 | 7.64E-04 | 0.342751843 | 0 | 6 | 6 | 4 | FALSE | 136.5 | 0 | 6 | 2 | 0.990064442 | FALSE | 0 | HSD17B1 | 46848 | 0.517175573 | gene |
| ABCC1 | 2.917562724 | 7.64E-04 | 0.342751843 | 0 | 6 | 6 | 4 | FALSE | 136.5 | 0 | 6 | 2 | 0.990064442 | FALSE | 0 | ABCC1 | 46848 | 0.517175573 | gene |
| HSD17B2 | 2.917562724 | 7.64E-04 | 0.342751843 | 0 | 6 | 6 | 4 | FALSE | 136.5 | 0 | 6 | 2 | 0.990064442 | FALSE | 0 | HSD17B2 | 46848 | 0.517175573 | gene |
| NOX4 | 2.917562724 | 7.64E-04 | 0.342751843 | 0 | 6 | 6 | 4 | FALSE | 136.5 | 0 | 6 | 2 | 0.990064442 | FALSE | 0 | NOX4 | 46848 | 0.517175573 | gene |
| MAOA | 2.910394265 | 0.001219331 | 0.343596059 | 0 | 6 | 6 | 4 | FALSE | 115.5 | 0 | 6 | 2 | 0.990101584 | FALSE | 0 | MAOA | 39984 | 0.433712121 | gene |
| AKR1C3 | 2.917562724 | 7.64E-04 | 0.342751843 | 0 | 6 | 6 | 4 | FALSE | 136.5 | 0 | 6 | 2 | 0.990064442 | FALSE | 0 | AKR1C3 | 46848 | 0.517175573 | gene |
| CYP1B1 | 2.917562724 | 7.64E-04 | 0.342751843 | 0 | 6 | 6 | 4 | FALSE | 136.5 | 0 | 6 | 2 | 0.990064442 | FALSE | 0 | CYP1B1 | 46848 | 0.517175573 | gene |
| PRKCA | 2.720430108 | 0.003467106 | 0.367588933 | 0 | 6 | 6 | 4 | FALSE | 115.5 | 0 | 6 | 2 | 0.991085854 | FALSE | 0 | PRKCA | 102152 | 0.361198738 | gene |
| SLC6A2 | 3.146953405 | 7.34E-04 | 0.317767654 | 0 | 6 | 6 | 4 | FALSE | 71 | 0 | 6 | 2 | 0.988875889 | FALSE | 0 | SLC6A2 | 22408 | 0.353535354 | gene |
| DS47 | 2.85483871 | 3.69E-04 | 0.350282486 | 0 | 6 | 6 | 4 | FALSE | 33.16666667 | 0 | 6 | 0 | 0.990389437 | FALSE | 0 | DS47 | 35574 | 0.45952381 | Danshen |
| DS40 | 3.238351254 | 1.95E-04 | 0.308799115 | 0 | 6 | 6 | 5 | FALSE | 34.16666667 | 0 | 6 | 0 | 0.988402325 | FALSE | 0 | DS40 | 15238 | 0.495024876 | Danshen |
| DS26 | 3.238351254 | 1.93E-04 | 0.308799115 | 0 | 6 | 6 | 5 | FALSE | 34 | 0 | 6 | 0 | 0.988402325 | FALSE | 0 | DS26 | 14020 | 0.492537313 | Danshen |
| DS20 | 3.238351254 | 1.69E-04 | 0.308799115 | 0 | 6 | 6 | 5 | FALSE | 35.66666667 | 0 | 6 | 0 | 0.988402325 | FALSE | 0 | DS20 | 14120 | 0.517412935 | Danshen |
| DS19 | 3.238351254 | 2.98E-04 | 0.308799115 | 0 | 6 | 6 | 5 | FALSE | 30 | 0 | 6 | 0 | 0.988402325 | FALSE | 0 | DS19 | 14212 | 0.432835821 | Danshen |
| XDH | 3.017921147 | 5.64E-04 | 0.331353919 | 0 | 6 | 6 | 4 | FALSE | 111.5 | 0 | 6 | 2 | 0.98954445 | FALSE | 0 | XDH | 19950 | 0.472222222 | gene |
| VEGFA | 2.863799283 | 0.001321243 | 0.349186483 | 0 | 6 | 6 | 4 | FALSE | 106.2 | 0 | 6 | 1 | 0.990343009 | FALSE | 0 | VEGFA | 56796 | 0.38115942 | gene |
| DRD2 | 3.580645161 | 9.46E-05 | 0.279279279 | 0 | 6 | 6 | 4 | FALSE | 27.4 | 0 | 6 | 1 | 0.986628782 | FALSE | 0 | DRD2 | 2314 | 0.347368421 | gene |
| HTR3A | 3.738351254 | 3.77E-05 | 0.267497603 | 0 | 6 | 6 | 4 | FALSE | 16.5 | 0 | 6 | 0 | 0.985811652 | FALSE | 0 | HTR3A | 1240 | 0.5 | gene |
| CDK2 | 2.802867384 | 0.00294078 | 0.356777494 | 0 | 6 | 6 | 5 | FALSE | 124.5 | 0 | 6 | 2 | 0.990658718 | FALSE | 0 | CDK2 | 77278 | 0.418644068 | gene |
| CSNK2A1 | 2.802867384 | 0.00294078 | 0.356777494 | 0 | 6 | 6 | 5 | FALSE | 124.5 | 0 | 6 | 2 | 0.990658718 | FALSE | 0 | CSNK2A1 | 77278 | 0.418644068 | gene |
| PTK2 | 2.802867384 | 0.00294078 | 0.356777494 | 0 | 6 | 6 | 5 | FALSE | 124.5 | 0 | 6 | 2 | 0.990658718 | FALSE | 0 | PTK2 | 77278 | 0.418644068 | gene |
| MAPK1 | 2.465949821 | 0.007214118 | 0.405523256 | 0 | 6 | 6 | 4 | FALSE | 111.2 | 0 | 6 | 1 | 0.992404405 | FALSE | 0 | MAPK1 | 146312 | 0.284754522 | gene |
| MMP1 | 2.831541219 | 0.002360976 | 0.353164557 | 0 | 6 | 6 | 4 | FALSE | 118.5 | 0 | 6 | 2 | 0.990510149 | FALSE | 0 | MMP1 | 54382 | 0.410839161 | gene |
| ALOX5 | 2.781362007 | 0.002800454 | 0.359536082 | 0 | 6 | 6 | 4 | FALSE | 125.25 | 0 | 6 | 2 | 0.990770145 | FALSE | 0 | ALOX5 | 64206 | 0.414166667 | gene |
| PPARD | 2.788530466 | 0.002414934 | 0.358611825 | 0 | 6 | 6 | 4 | FALSE | 105.5 | 0 | 6 | 2 | 0.990733003 | FALSE | 0 | PPARD | 62176 | 0.350671141 | gene |
| PTGER2 | 3.014336918 | 0.002132547 | 0.331747919 | 0 | 6 | 6 | 5 | FALSE | 89.6 | 0 | 6 | 1 | 0.989563021 | FALSE | 0 | PTGER2 | 65104 | 0.377021277 | gene |
| SHBG | 3.014336918 | 0.002132547 | 0.331747919 | 0 | 6 | 6 | 5 | FALSE | 89.6 | 0 | 6 | 1 | 0.989563021 | FALSE | 0 | SHBG | 65104 | 0.377021277 | gene |
| RORC | 3.050179211 | 0.003409455 | 0.327849589 | 0 | 6 | 6 | 5 | FALSE | 86.4 | 0 | 6 | 1 | 0.98937731 | FALSE | 0 | RORC | 100378 | 0.379555556 | gene |
| POLB | 3.014336918 | 0.002132547 | 0.331747919 | 0 | 6 | 6 | 5 | FALSE | 89.6 | 0 | 6 | 1 | 0.989563021 | FALSE | 0 | POLB | 65104 | 0.377021277 | gene |
| AKR1C1 | 2.931899642 | 4.55E-04 | 0.341075795 | 0 | 5 | 5 | 5 | FALSE | 148.3333333 | 0 | 5 | 2 | 0.989990157 | FALSE | 0 | AKR1C1 | 25984 | 0.566666667 | gene |
| NUAK1 | 3.050179211 | 3.30E-04 | 0.327849589 | 0 | 5 | 5 | 5 | FALSE | 133.3333333 | 0 | 5 | 2 | 0.98937731 | FALSE | 0 | NUAK1 | 13000 | 0.582966226 | gene |
| CA14 | 3.050179211 | 3.30E-04 | 0.327849589 | 0 | 5 | 5 | 5 | FALSE | 133.3333333 | 0 | 5 | 2 | 0.98937731 | FALSE | 0 | CA14 | 13000 | 0.582966226 | gene |
| PLK1 | 3.050179211 | 3.30E-04 | 0.327849589 | 0 | 5 | 5 | 5 | FALSE | 133.3333333 | 0 | 5 | 2 | 0.98937731 | FALSE | 0 | PLK1 | 13000 | 0.582966226 | gene |
| GPR35 | 3.050179211 | 3.30E-04 | 0.327849589 | 0 | 5 | 5 | 5 | FALSE | 133.3333333 | 0 | 5 | 2 | 0.98937731 | FALSE | 0 | GPR35 | 13000 | 0.582966226 | gene |
| BCHE | 3.222222222 | 8.10E-04 | 0.310344828 | 0 | 5 | 5 | 5 | FALSE | 86.75 | 0 | 5 | 1 | 0.988485895 | FALSE | 0 | BCHE | 29802 | 0.481741573 | gene |
| MAOB | 2.831541219 | 0.001304339 | 0.353164557 | 0 | 5 | 5 | 4 | FALSE | 118.5 | 0 | 5 | 1 | 0.990510149 | FALSE | 0 | MAOB | 55144 | 0.410839161 | gene |
| GSTM2 | 3.050179211 | 3.30E-04 | 0.327849589 | 0 | 5 | 5 | 5 | FALSE | 133.3333333 | 0 | 5 | 2 | 0.98937731 | FALSE | 0 | GSTM2 | 13000 | 0.582966226 | gene |
| GSTM1 | 3.050179211 | 3.30E-04 | 0.327849589 | 0 | 5 | 5 | 5 | FALSE | 133.3333333 | 0 | 5 | 2 | 0.98937731 | FALSE | 0 | GSTM1 | 13000 | 0.582966226 | gene |
| NR1I2 | 3.086021505 | 3.31E-04 | 0.324041812 | 0 | 5 | 5 | 4 | FALSE | 119.6666667 | 0 | 5 | 2 | 0.989191598 | FALSE | 0 | NR1I2 | 9238 | 0.549382716 | gene |
| CYP1A1 | 3.086021505 | 3.31E-04 | 0.324041812 | 0 | 5 | 5 | 4 | FALSE | 119.6666667 | 0 | 5 | 2 | 0.989191598 | FALSE | 0 | CYP1A1 | 9238 | 0.549382716 | gene |
| CYP1A2 | 3.086021505 | 3.31E-04 | 0.324041812 | 0 | 5 | 5 | 4 | FALSE | 119.6666667 | 0 | 5 | 2 | 0.989191598 | FALSE | 0 | CYP1A2 | 9238 | 0.549382716 | gene |
| DS52 | 3.241935484 | 9.54E-05 | 0.308457711 | 0 | 5 | 5 | 5 | FALSE | 40.2 | 0 | 5 | 0 | 0.988383754 | FALSE | 0 | DS52 | 10278 | 0.585074627 | Danshen |
| DS22 | 3.241935484 | 9.54E-05 | 0.308457711 | 0 | 5 | 5 | 5 | FALSE | 40.2 | 0 | 5 | 0 | 0.988383754 | FALSE | 0 | DS22 | 10278 | 0.585074627 | Danshen |
| DS15 | 3.012544803 | 1.59E-04 | 0.331945271 | 0 | 5 | 5 | 5 | FALSE | 41.4 | 0 | 5 | 0 | 0.989572307 | FALSE | 0 | DS15 | 23628 | 0.585507246 | Danshen |
| TYR | 3.103942652 | 1.85E-04 | 0.322170901 | 0 | 5 | 5 | 4 | FALSE | 124.3333333 | 0 | 5 | 2 | 0.989098743 | FALSE | 0 | TYR | 6678 | 0.584518167 | gene |
| ICAM1 | 3.103942652 | 1.85E-04 | 0.322170901 | 0 | 5 | 5 | 4 | FALSE | 124.3333333 | 0 | 5 | 2 | 0.989098743 | FALSE | 0 | ICAM1 | 6678 | 0.584518167 | gene |
| HMOX1 | 3.103942652 | 1.85E-04 | 0.322170901 | 0 | 5 | 5 | 4 | FALSE | 124.3333333 | 0 | 5 | 2 | 0.989098743 | FALSE | 0 | HMOX1 | 6678 | 0.584518167 | gene |
| TOP1 | 3.103942652 | 1.85E-04 | 0.322170901 | 0 | 5 | 5 | 4 | FALSE | 124.3333333 | 0 | 5 | 2 | 0.989098743 | FALSE | 0 | TOP1 | 6678 | 0.584518167 | gene |
| NFKBIA | 3.010752688 | 7.63E-04 | 0.332142857 | 0 | 5 | 5 | 4 | FALSE | 86 | 0 | 5 | 1 | 0.989581592 | FALSE | 0 | NFKBIA | 19622 | 0.360169492 | gene |
| CDKN1A | 3.010752688 | 7.63E-04 | 0.332142857 | 0 | 5 | 5 | 4 | FALSE | 86 | 0 | 5 | 1 | 0.989581592 | FALSE | 0 | CDKN1A | 19622 | 0.360169492 | gene |
| NR3C2 | 3.329749104 | 0.001284864 | 0.300322928 | 0 | 5 | 5 | 4 | FALSE | 35 | 0 | 5 | 0 | 0.987928761 | FALSE | 0 | NR3C2 | 63036 | 0.232876712 | gene |
| PDE3A | 3.745519713 | 1.41E-05 | 0.266985646 | 0 | 5 | 5 | 4 | FALSE | 19.4 | 0 | 5 | 0 | 0.985774509 | FALSE | 0 | PDE3A | 296 | 0.613333333 | gene |
| ABCB1 | 2.874551971 | 0.002130598 | 0.347880299 | 0 | 5 | 5 | 5 | FALSE | 141.6666667 | 0 | 5 | 2 | 0.990287295 | FALSE | 0 | ABCB1 | 54156 | 0.509661836 | gene |
| PIK3R1 | 2.874551971 | 0.002130598 | 0.347880299 | 0 | 5 | 5 | 5 | FALSE | 141.6666667 | 0 | 5 | 2 | 0.990287295 | FALSE | 0 | PIK3R1 | 54156 | 0.509661836 | gene |
| PARP1 | 2.874551971 | 0.002130598 | 0.347880299 | 0 | 5 | 5 | 5 | FALSE | 141.6666667 | 0 | 5 | 2 | 0.990287295 | FALSE | 0 | PARP1 | 54156 | 0.509661836 | gene |
| ERBB2 | 2.806451613 | 0.002679699 | 0.356321839 | 0 | 5 | 5 | 4 | FALSE | 102.5 | 0 | 5 | 1 | 0.990640147 | FALSE | 0 | ERBB2 | 65526 | 0.346416382 | gene |
| PIM1 | 2.856630824 | 0.001887217 | 0.350062735 | 0 | 5 | 5 | 4 | FALSE | 142.6666667 | 0 | 5 | 2 | 0.990380151 | FALSE | 0 | PIM1 | 43788 | 0.505952381 | gene |
| CASP8 | 2.82078853 | 0.00179598 | 0.354510801 | 0 | 5 | 5 | 4 | FALSE | 120 | 0 | 5 | 2 | 0.990565863 | FALSE | 0 | CASP8 | 43232 | 0.410344828 | gene |
| AURKA | 2.784946237 | 0.005777138 | 0.359073359 | 0 | 5 | 5 | 5 | FALSE | 98.2 | 0 | 5 | 0 | 0.990751574 | FALSE | 0 | AURKA | 111614 | 0.325083612 | gene |
| PTGER3 | 2.716845878 | 0.002683045 | 0.368073879 | 0 | 5 | 5 | 4 | FALSE | 128.25 | 0 | 5 | 1 | 0.991104426 | FALSE | 0 | PTGER3 | 77748 | 0.400157233 | gene |
| HMGCR | 3.017921147 | 0.001387329 | 0.331353919 | 0 | 5 | 5 | 5 | FALSE | 86.75 | 0 | 5 | 1 | 0.98954445 | FALSE | 0 | HMGCR | 40556 | 0.364893617 | gene |
| AKR1B10 | 2.856630824 | 0.001887217 | 0.350062735 | 0 | 5 | 5 | 4 | FALSE | 142.6666667 | 0 | 5 | 2 | 0.990380151 | FALSE | 0 | AKR1B10 | 43788 | 0.505952381 | gene |
| SERPINE1 | 2.956989247 | 4.88E-04 | 0.338181818 | 0 | 4 | 4 | 4 | FALSE | 137.3333333 | 0 | 4 | 1 | 0.989860159 | FALSE | 0 | SERPINE1 | 27780 | 0.541005291 | gene |
| IGFBP3 | 2.956989247 | 4.88E-04 | 0.338181818 | 0 | 4 | 4 | 4 | FALSE | 137.3333333 | 0 | 4 | 1 | 0.989860159 | FALSE | 0 | IGFBP3 | 27780 | 0.541005291 | gene |
| F3 | 2.956989247 | 4.88E-04 | 0.338181818 | 0 | 4 | 4 | 4 | FALSE | 137.3333333 | 0 | 4 | 1 | 0.989860159 | FALSE | 0 | F3 | 27780 | 0.541005291 | gene |
| VCP | 3.111111111 | 0.002008531 | 0.321428571 | 0 | 4 | 4 | 5 | FALSE | 105.5 | 0 | 4 | 0 | 0.9890616 | FALSE | 0 | VCP | 73308 | 0.5 | gene |
| GRM5 | 3.111111111 | 0.002008531 | 0.321428571 | 0 | 4 | 4 | 5 | FALSE | 105.5 | 0 | 4 | 0 | 0.9890616 | FALSE | 0 | GRM5 | 73308 | 0.5 | gene |
| CTSB | 3.111111111 | 0.002008531 | 0.321428571 | 0 | 4 | 4 | 5 | FALSE | 105.5 | 0 | 4 | 0 | 0.9890616 | FALSE | 0 | CTSB | 73308 | 0.5 | gene |
| MAPT | 3.136200717 | 2.94E-05 | 0.318857143 | 0 | 4 | 4 | 5 | FALSE | 163.5 | 0 | 4 | 2 | 0.988931603 | FALSE | 0 | MAPT | 1790 | 0.796568627 | gene |
| CD38 | 3.136200717 | 2.94E-05 | 0.318857143 | 0 | 4 | 4 | 5 | FALSE | 163.5 | 0 | 4 | 2 | 0.988931603 | FALSE | 0 | CD38 | 1790 | 0.796568627 | gene |
| AKR1A1 | 3.136200717 | 2.94E-05 | 0.318857143 | 0 | 4 | 4 | 5 | FALSE | 163.5 | 0 | 4 | 2 | 0.988931603 | FALSE | 0 | AKR1A1 | 1790 | 0.796568627 | gene |
| AKR1C4 | 3.136200717 | 2.94E-05 | 0.318857143 | 0 | 4 | 4 | 5 | FALSE | 163.5 | 0 | 4 | 2 | 0.988931603 | FALSE | 0 | AKR1C4 | 1790 | 0.796568627 | gene |
| AKR1C2 | 3.136200717 | 2.94E-05 | 0.318857143 | 0 | 4 | 4 | 5 | FALSE | 163.5 | 0 | 4 | 2 | 0.988931603 | FALSE | 0 | AKR1C2 | 1790 | 0.796568627 | gene |
| AXL | 3.136200717 | 2.94E-05 | 0.318857143 | 0 | 4 | 4 | 5 | FALSE | 163.5 | 0 | 4 | 2 | 0.988931603 | FALSE | 0 | AXL | 1790 | 0.796568627 | gene |
| NEK6 | 3.136200717 | 2.94E-05 | 0.318857143 | 0 | 4 | 4 | 5 | FALSE | 163.5 | 0 | 4 | 2 | 0.988931603 | FALSE | 0 | NEK6 | 1790 | 0.796568627 | gene |
| ALK | 3.136200717 | 2.94E-05 | 0.318857143 | 0 | 4 | 4 | 5 | FALSE | 163.5 | 0 | 4 | 2 | 0.988931603 | FALSE | 0 | ALK | 1790 | 0.796568627 | gene |
| CAMK2B | 3.136200717 | 2.94E-05 | 0.318857143 | 0 | 4 | 4 | 5 | FALSE | 163.5 | 0 | 4 | 2 | 0.988931603 | FALSE | 0 | CAMK2B | 1790 | 0.796568627 | gene |
| CXCR1 | 3.136200717 | 2.94E-05 | 0.318857143 | 0 | 4 | 4 | 5 | FALSE | 163.5 | 0 | 4 | 2 | 0.988931603 | FALSE | 0 | CXCR1 | 1790 | 0.796568627 | gene |
| NEK2 | 3.136200717 | 2.94E-05 | 0.318857143 | 0 | 4 | 4 | 5 | FALSE | 163.5 | 0 | 4 | 2 | 0.988931603 | FALSE | 0 | NEK2 | 1790 | 0.796568627 | gene |
| PKN1 | 3.136200717 | 2.94E-05 | 0.318857143 | 0 | 4 | 4 | 5 | FALSE | 163.5 | 0 | 4 | 2 | 0.988931603 | FALSE | 0 | PKN1 | 1790 | 0.796568627 | gene |
| PYGL | 3.136200717 | 2.94E-05 | 0.318857143 | 0 | 4 | 4 | 5 | FALSE | 163.5 | 0 | 4 | 2 | 0.988931603 | FALSE | 0 | PYGL | 1790 | 0.796568627 | gene |
| MPO | 3.136200717 | 2.94E-05 | 0.318857143 | 0 | 4 | 4 | 5 | FALSE | 163.5 | 0 | 4 | 2 | 0.988931603 | FALSE | 0 | MPO | 1790 | 0.796568627 | gene |
| DRD4 | 3.136200717 | 2.94E-05 | 0.318857143 | 0 | 4 | 4 | 5 | FALSE | 163.5 | 0 | 4 | 2 | 0.988931603 | FALSE | 0 | DRD4 | 1790 | 0.796568627 | gene |
| F2 | 3.136200717 | 2.94E-05 | 0.318857143 | 0 | 4 | 4 | 5 | FALSE | 163.5 | 0 | 4 | 2 | 0.988931603 | FALSE | 0 | F2 | 1790 | 0.796568627 | gene |
| AVPR2 | 3.136200717 | 2.94E-05 | 0.318857143 | 0 | 4 | 4 | 5 | FALSE | 163.5 | 0 | 4 | 2 | 0.988931603 | FALSE | 0 | AVPR2 | 1790 | 0.796568627 | gene |
| CDK6 | 3.136200717 | 2.94E-05 | 0.318857143 | 0 | 4 | 4 | 5 | FALSE | 163.5 | 0 | 4 | 2 | 0.988931603 | FALSE | 0 | CDK6 | 1790 | 0.796568627 | gene |
| TNKS | 3.136200717 | 2.94E-05 | 0.318857143 | 0 | 4 | 4 | 5 | FALSE | 163.5 | 0 | 4 | 2 | 0.988931603 | FALSE | 0 | TNKS | 1790 | 0.796568627 | gene |
| TNKS2 | 3.136200717 | 2.94E-05 | 0.318857143 | 0 | 4 | 4 | 5 | FALSE | 163.5 | 0 | 4 | 2 | 0.988931603 | FALSE | 0 | TNKS2 | 1790 | 0.796568627 | gene |
| TTR | 3.136200717 | 2.94E-05 | 0.318857143 | 0 | 4 | 4 | 5 | FALSE | 163.5 | 0 | 4 | 2 | 0.988931603 | FALSE | 0 | TTR | 1790 | 0.796568627 | gene |
| SLC22A12 | 3.136200717 | 2.94E-05 | 0.318857143 | 0 | 4 | 4 | 5 | FALSE | 163.5 | 0 | 4 | 2 | 0.988931603 | FALSE | 0 | SLC22A12 | 1790 | 0.796568627 | gene |
| MPG | 3.136200717 | 2.94E-05 | 0.318857143 | 0 | 4 | 4 | 5 | FALSE | 163.5 | 0 | 4 | 2 | 0.988931603 | FALSE | 0 | MPG | 1790 | 0.796568627 | gene |
| DAPK1 | 3.136200717 | 2.94E-05 | 0.318857143 | 0 | 4 | 4 | 5 | FALSE | 163.5 | 0 | 4 | 2 | 0.988931603 | FALSE | 0 | DAPK1 | 1790 | 0.796568627 | gene |
| ARG1 | 3.136200717 | 2.94E-05 | 0.318857143 | 0 | 4 | 4 | 5 | FALSE | 163.5 | 0 | 4 | 2 | 0.988931603 | FALSE | 0 | ARG1 | 1790 | 0.796568627 | gene |
| CCNB3 | 3.136200717 | 2.94E-05 | 0.318857143 | 0 | 4 | 4 | 5 | FALSE | 163.5 | 0 | 4 | 2 | 0.988931603 | FALSE | 0 | CCNB3 | 1790 | 0.796568627 | gene |
| PTPRS | 3.136200717 | 2.94E-05 | 0.318857143 | 0 | 4 | 4 | 5 | FALSE | 163.5 | 0 | 4 | 2 | 0.988931603 | FALSE | 0 | PTPRS | 1790 | 0.796568627 | gene |
| GLO1 | 3.136200717 | 2.94E-05 | 0.318857143 | 0 | 4 | 4 | 5 | FALSE | 163.5 | 0 | 4 | 2 | 0.988931603 | FALSE | 0 | GLO1 | 1790 | 0.796568627 | gene |
| FLT3 | 3.136200717 | 2.94E-05 | 0.318857143 | 0 | 4 | 4 | 5 | FALSE | 163.5 | 0 | 4 | 2 | 0.988931603 | FALSE | 0 | FLT3 | 1790 | 0.796568627 | gene |
| SQLE | 3.362007168 | 4.78E-04 | 0.297441365 | 0 | 4 | 4 | 5 | FALSE | 67 | 0 | 4 | 1 | 0.987761621 | FALSE | 0 | SQLE | 10976 | 0.471428571 | gene |
| AHR | 3.136200717 | 2.94E-05 | 0.318857143 | 0 | 4 | 4 | 5 | FALSE | 163.5 | 0 | 4 | 2 | 0.988931603 | FALSE | 0 | AHR | 1790 | 0.796568627 | gene |
| VCAM1 | 3.136200717 | 2.94E-05 | 0.318857143 | 0 | 4 | 4 | 5 | FALSE | 163.5 | 0 | 4 | 2 | 0.988931603 | FALSE | 0 | VCAM1 | 1790 | 0.796568627 | gene |
| SELE | 3.136200717 | 2.94E-05 | 0.318857143 | 0 | 4 | 4 | 5 | FALSE | 163.5 | 0 | 4 | 2 | 0.988931603 | FALSE | 0 | SELE | 1790 | 0.796568627 | gene |
| MAPK8 | 3.071684588 | 0.001181349 | 0.325554259 | 0 | 4 | 4 | 5 | FALSE | 112.3333333 | 0 | 4 | 1 | 0.989265883 | FALSE | 0 | MAPK8 | 45814 | 0.503770739 | gene |
| PON1 | 3.014336918 | 4.26E-04 | 0.331747919 | 0 | 4 | 4 | 4 | FALSE | 129.5 | 0 | 4 | 2 | 0.989563021 | FALSE | 0 | PON1 | 13046 | 0.542194093 | gene |
| TGFB1 | 3.014336918 | 4.26E-04 | 0.331747919 | 0 | 4 | 4 | 4 | FALSE | 129.5 | 0 | 4 | 2 | 0.989563021 | FALSE | 0 | TGFB1 | 13046 | 0.542194093 | gene |
| DS54 | 3.245519713 | 5.72E-05 | 0.308117062 | 0 | 4 | 4 | 5 | FALSE | 45.5 | 0 | 4 | 0 | 0.988365183 | FALSE | 0 | DS54 | 6886 | 0.664179104 | Danshen |
| DS53 | 3.245519713 | 5.72E-05 | 0.308117062 | 0 | 4 | 4 | 5 | FALSE | 45.5 | 0 | 4 | 0 | 0.988365183 | FALSE | 0 | DS53 | 6886 | 0.664179104 | Danshen |
| F7 | 3.125448029 | 9.22E-04 | 0.319954128 | 0 | 4 | 4 | 4 | FALSE | 110 | 0 | 4 | 1 | 0.988987316 | FALSE | 0 | F7 | 56646 | 0.531707317 | gene |
| DS23 | 3.245519713 | 6.90E-05 | 0.308117062 | 0 | 4 | 4 | 5 | FALSE | 42.75 | 0 | 4 | 0 | 0.988365183 | FALSE | 0 | DS23 | 7300 | 0.623134328 | Danshen |
| BIRC5 | 3.114695341 | 2.77E-04 | 0.321058688 | 0 | 4 | 4 | 4 | FALSE | 86.66666667 | 0 | 4 | 1 | 0.989043029 | FALSE | 0 | BIRC5 | 6866 | 0.411858974 | gene |
| TP53 | 3.103942652 | 3.30E-04 | 0.322170901 | 0 | 4 | 4 | 4 | FALSE | 90.33333333 | 0 | 4 | 1 | 0.989098743 | FALSE | 0 | TP53 | 8096 | 0.423380727 | gene |
| RORA | 3.336917563 | 3.45E-04 | 0.299677766 | 0 | 4 | 4 | 5 | FALSE | 76.33333333 | 0 | 4 | 1 | 0.987891619 | FALSE | 0 | RORA | 10762 | 0.512471655 | gene |
| IL6 | 2.870967742 | 0.001617758 | 0.348314607 | 0 | 4 | 4 | 4 | FALSE | 113.3333333 | 0 | 4 | 1 | 0.990305867 | FALSE | 0 | IL6 | 33462 | 0.407004831 | gene |
| EDNRA | 3.068100358 | 0.00183758 | 0.325934579 | 0 | 4 | 4 | 4 | FALSE | 88 | 0 | 4 | 0 | 0.989284454 | FALSE | 0 | EDNRA | 38360 | 0.395454545 | gene |
| CYP17A1 | 3.336917563 | 3.45E-04 | 0.299677766 | 0 | 4 | 4 | 5 | FALSE | 76.33333333 | 0 | 4 | 1 | 0.987891619 | FALSE | 0 | CYP17A1 | 10762 | 0.512471655 | gene |
| NPC1L1 | 3.336917563 | 3.45E-04 | 0.299677766 | 0 | 4 | 4 | 5 | FALSE | 76.33333333 | 0 | 4 | 1 | 0.987891619 | FALSE | 0 | NPC1L1 | 10762 | 0.512471655 | gene |
| NR1H3 | 3.336917563 | 3.45E-04 | 0.299677766 | 0 | 4 | 4 | 5 | FALSE | 76.33333333 | 0 | 4 | 1 | 0.987891619 | FALSE | 0 | NR1H3 | 10762 | 0.512471655 | gene |
| PPARA | 2.724014337 | 0.002060098 | 0.367105263 | 0 | 4 | 4 | 4 | FALSE | 137.3333333 | 0 | 4 | 1 | 0.991067283 | FALSE | 0 | PPARA | 53370 | 0.430073607 | gene |
| CYP51A1 | 3.336917563 | 3.45E-04 | 0.299677766 | 0 | 4 | 4 | 5 | FALSE | 76.33333333 | 0 | 4 | 1 | 0.987891619 | FALSE | 0 | CYP51A1 | 10762 | 0.512471655 | gene |
| G6PD | 3.336917563 | 3.45E-04 | 0.299677766 | 0 | 4 | 4 | 5 | FALSE | 76.33333333 | 0 | 4 | 1 | 0.987891619 | FALSE | 0 | G6PD | 10762 | 0.512471655 | gene |
| SERPINA6 | 3.336917563 | 3.45E-04 | 0.299677766 | 0 | 4 | 4 | 5 | FALSE | 76.33333333 | 0 | 4 | 1 | 0.987891619 | FALSE | 0 | SERPINA6 | 10762 | 0.512471655 | gene |
| CDC25A | 3.336917563 | 3.45E-04 | 0.299677766 | 0 | 4 | 4 | 5 | FALSE | 76.33333333 | 0 | 4 | 1 | 0.987891619 | FALSE | 0 | CDC25A | 10762 | 0.512471655 | gene |
| PTPN6 | 3.336917563 | 3.45E-04 | 0.299677766 | 0 | 4 | 4 | 5 | FALSE | 76.33333333 | 0 | 4 | 1 | 0.987891619 | FALSE | 0 | PTPN6 | 10762 | 0.512471655 | gene |
| HSD11B2 | 3.336917563 | 3.45E-04 | 0.299677766 | 0 | 4 | 4 | 5 | FALSE | 76.33333333 | 0 | 4 | 1 | 0.987891619 | FALSE | 0 | HSD11B2 | 10762 | 0.512471655 | gene |
| HSD11B1 | 3.336917563 | 3.45E-04 | 0.299677766 | 0 | 4 | 4 | 5 | FALSE | 76.33333333 | 0 | 4 | 1 | 0.987891619 | FALSE | 0 | HSD11B1 | 10762 | 0.512471655 | gene |
| SNCA | 3.301075269 | 2.32E-04 | 0.302931596 | 0 | 3 | 3 | 5 | FALSE | 97.33333333 | 0 | 3 | 0 | 0.98807733 | FALSE | 0 | SNCA | 9414 | 0.609704641 | gene |
| GZ6 | 3.048387097 | 4.11E-05 | 0.328042328 | 0 | 3 | 3 | 5 | FALSE | 34.66666667 | 0 | 3 | 0 | 0.989386595 | FALSE | 0 | GZ6 | 3128 | 0.551912568 | Guangzao |
| CBR1 | 3.301075269 | 2.32E-04 | 0.302931596 | 0 | 3 | 3 | 5 | FALSE | 97.33333333 | 0 | 3 | 0 | 0.98807733 | FALSE | 0 | CBR1 | 9414 | 0.609704641 | gene |
| CA5B | 3.301075269 | 2.32E-04 | 0.302931596 | 0 | 3 | 3 | 5 | FALSE | 97.33333333 | 0 | 3 | 0 | 0.98807733 | FALSE | 0 | CA5B | 9414 | 0.609704641 | gene |
| SOD1 | 2.982078853 | 2.81E-04 | 0.335336538 | 0 | 3 | 3 | 5 | FALSE | 155.5 | 0 | 3 | 1 | 0.989730161 | FALSE | 0 | SOD1 | 14770 | 0.620481928 | gene |
| PRKCB | 3.089605735 | 2.00E-04 | 0.323665893 | 0 | 3 | 3 | 5 | FALSE | 133 | 0 | 3 | 1 | 0.989173027 | FALSE | 0 | PRKCB | 6902 | 0.608294931 | gene |
| CXCL8 | 3.089605735 | 2.00E-04 | 0.323665893 | 0 | 3 | 3 | 5 | FALSE | 133 | 0 | 3 | 1 | 0.989173027 | FALSE | 0 | CXCL8 | 6902 | 0.608294931 | gene |
| MAP3K8 | 3.265232975 | 0.001119108 | 0.306256861 | 0 | 3 | 3 | 5 | FALSE | 92 | 0 | 3 | 0 | 0.988263042 | FALSE | 0 | MAP3K8 | 26448 | 0.541666667 | gene |
| GLRA1 | 3.58781362 | 1.04E-05 | 0.278721279 | 0 | 3 | 3 | 5 | FALSE | 64 | 0 | 3 | 1 | 0.986591639 | FALSE | 0 | GLRA1 | 376 | 0.807692308 | gene |
| DHCR7 | 3.58781362 | 1.04E-05 | 0.278721279 | 0 | 3 | 3 | 5 | FALSE | 64 | 0 | 3 | 1 | 0.986591639 | FALSE | 0 | DHCR7 | 376 | 0.807692308 | gene |
| NR1H2 | 3.58781362 | 1.04E-05 | 0.278721279 | 0 | 3 | 3 | 5 | FALSE | 64 | 0 | 3 | 1 | 0.986591639 | FALSE | 0 | NR1H2 | 376 | 0.807692308 | gene |
| VDR | 3.58781362 | 1.04E-05 | 0.278721279 | 0 | 3 | 3 | 5 | FALSE | 64 | 0 | 3 | 1 | 0.986591639 | FALSE | 0 | VDR | 376 | 0.807692308 | gene |
| CHRM2 | 3.58781362 | 1.04E-05 | 0.278721279 | 0 | 3 | 3 | 5 | FALSE | 64 | 0 | 3 | 1 | 0.986591639 | FALSE | 0 | CHRM2 | 376 | 0.807692308 | gene |
| CYP2C19 | 3.58781362 | 1.04E-05 | 0.278721279 | 0 | 3 | 3 | 5 | FALSE | 64 | 0 | 3 | 1 | 0.986591639 | FALSE | 0 | CYP2C19 | 376 | 0.807692308 | gene |
| SREBF2 | 3.58781362 | 1.04E-05 | 0.278721279 | 0 | 3 | 3 | 5 | FALSE | 64 | 0 | 3 | 1 | 0.986591639 | FALSE | 0 | SREBF2 | 376 | 0.807692308 | gene |
| PLAU | 3.014336918 | 4.36E-04 | 0.331747919 | 0 | 3 | 3 | 4 | FALSE | 127.5 | 0 | 3 | 1 | 0.989563021 | FALSE | 0 | PLAU | 13316 | 0.533755274 | gene |
| MYC | 3.129032258 | 1.87E-04 | 0.319587629 | 0 | 3 | 3 | 4 | FALSE | 112.5 | 0 | 3 | 1 | 0.988968745 | FALSE | 0 | MYC | 4428 | 0.543902439 | gene |
| FASN | 3.358422939 | 2.57E-04 | 0.297758805 | 0 | 3 | 3 | 4 | FALSE | 52 | 0 | 3 | 0 | 0.987780192 | FALSE | 0 | FASN | 6380 | 0.364285714 | gene |
| FOS | 3.129032258 | 1.87E-04 | 0.319587629 | 0 | 3 | 3 | 4 | FALSE | 112.5 | 0 | 3 | 1 | 0.988968745 | FALSE | 0 | FOS | 4428 | 0.543902439 | gene |
| DS49 | 3.801075269 | 9.81E-05 | 0.263083451 | 0 | 3 | 3 | 5 | FALSE | 26.33333333 | 0 | 3 | 0 | 0.985486657 | FALSE | 0 | DS49 | 2610 | 0.429378531 | Danshen |
| DS41 | 3.428315412 | 2.69E-05 | 0.291688447 | 0 | 3 | 3 | 5 | FALSE | 37.66666667 | 0 | 3 | 0 | 0.987418055 | FALSE | 0 | DS41 | 3568 | 0.564102564 | Danshen |
| CHRNA2 | 3.594982079 | 5.15E-05 | 0.278165503 | 0 | 3 | 3 | 4 | FALSE | 34.33333333 | 0 | 3 | 0 | 0.986554497 | FALSE | 0 | CHRNA2 | 1116 | 0.45045045 | gene |
| DS18 | 3.689964158 | 7.31E-05 | 0.271005342 | 0 | 3 | 3 | 5 | FALSE | 24 | 0 | 3 | 0 | 0.986062362 | FALSE | 0 | DS18 | 2386 | 0.383333333 | Danshen |
| ADRA2A | 3.609318996 | 4.56E-05 | 0.277060576 | 0 | 3 | 3 | 4 | FALSE | 34.33333333 | 0 | 3 | 0 | 0.986480212 | FALSE | 0 | ADRA2A | 1146 | 0.476190476 | gene |
| CD40LG | 3.150537634 | 9.24E-05 | 0.317406143 | 0 | 3 | 3 | 4 | FALSE | 119.5 | 0 | 3 | 1 | 0.988857318 | FALSE | 0 | CD40LG | 2704 | 0.595477387 | gene |
| TOP2A | 3.150537634 | 9.24E-05 | 0.317406143 | 0 | 3 | 3 | 4 | FALSE | 119.5 | 0 | 3 | 1 | 0.988857318 | FALSE | 0 | TOP2A | 2704 | 0.595477387 | gene |
| IFNG | 3.150537634 | 9.24E-05 | 0.317406143 | 0 | 3 | 3 | 4 | FALSE | 119.5 | 0 | 3 | 1 | 0.988857318 | FALSE | 0 | IFNG | 2704 | 0.595477387 | gene |
| IL2 | 3.150537634 | 9.24E-05 | 0.317406143 | 0 | 3 | 3 | 4 | FALSE | 119.5 | 0 | 3 | 1 | 0.988857318 | FALSE | 0 | IL2 | 2704 | 0.595477387 | gene |
| MCL1 | 3.35483871 | 5.97E-04 | 0.298076923 | 0 | 3 | 3 | 4 | FALSE | 83 | 0 | 3 | 0 | 0.987798763 | FALSE | 0 | MCL1 | 22456 | 0.581560284 | gene |
| RB1 | 3.150537634 | 9.24E-05 | 0.317406143 | 0 | 3 | 3 | 4 | FALSE | 119.5 | 0 | 3 | 1 | 0.988857318 | FALSE | 0 | RB1 | 2704 | 0.595477387 | gene |
| IL10 | 3.150537634 | 9.24E-05 | 0.317406143 | 0 | 3 | 3 | 4 | FALSE | 119.5 | 0 | 3 | 1 | 0.988857318 | FALSE | 0 | IL10 | 2704 | 0.595477387 | gene |
| DS7 | 3.249103943 | 7.94E-05 | 0.307777165 | 0 | 3 | 3 | 5 | FALSE | 41.66666667 | 0 | 3 | 0 | 0.988346612 | FALSE | 0 | DS7 | 5442 | 0.606965174 | Danshen |
| DS2 | 3.421146953 | 8.14E-05 | 0.292299633 | 0 | 3 | 3 | 5 | FALSE | 25 | 0 | 3 | 0 | 0.987455197 | FALSE | 0 | DS2 | 3276 | 0.387096774 | Danshen |
| FGFR1 | 3.143369176 | 0.001439942 | 0.318129989 | 0 | 3 | 3 | 5 | FALSE | 105.6666667 | 0 | 3 | 0 | 0.98889446 | FALSE | 0 | FGFR1 | 36972 | 0.520729685 | gene |
| PIK3CB | 3.143369176 | 0.001439942 | 0.318129989 | 0 | 3 | 3 | 5 | FALSE | 105.6666667 | 0 | 3 | 0 | 0.98889446 | FALSE | 0 | PIK3CB | 36972 | 0.520729685 | gene |
| LCK | 3.143369176 | 0.001439942 | 0.318129989 | 0 | 3 | 3 | 5 | FALSE | 105.6666667 | 0 | 3 | 0 | 0.98889446 | FALSE | 0 | LCK | 36972 | 0.520729685 | gene |
| TSPO | 3.29390681 | 0.001068495 | 0.30359086 | 0 | 3 | 3 | 5 | FALSE | 100.3333333 | 0 | 3 | 0 | 0.988114472 | FALSE | 0 | TSPO | 23616 | 0.558052434 | gene |
| PDE4A | 3.29390681 | 0.001068495 | 0.30359086 | 0 | 3 | 3 | 5 | FALSE | 100.3333333 | 0 | 3 | 0 | 0.988114472 | FALSE | 0 | PDE4A | 23616 | 0.558052434 | gene |
| CFTR | 3.114695341 | 7.31E-04 | 0.321058688 | 0 | 3 | 3 | 5 | FALSE | 116 | 0 | 3 | 1 | 0.989043029 | FALSE | 0 | CFTR | 22540 | 0.547619048 | gene |
| ELANE | 3.29390681 | 0.001068495 | 0.30359086 | 0 | 3 | 3 | 5 | FALSE | 100.3333333 | 0 | 3 | 0 | 0.988114472 | FALSE | 0 | ELANE | 23616 | 0.558052434 | gene |
| KIT | 3.143369176 | 0.001439942 | 0.318129989 | 0 | 3 | 3 | 5 | FALSE | 105.6666667 | 0 | 3 | 0 | 0.98889446 | FALSE | 0 | KIT | 36972 | 0.520729685 | gene |
| CAPN1 | 3.29390681 | 0.001068495 | 0.30359086 | 0 | 3 | 3 | 5 | FALSE | 100.3333333 | 0 | 3 | 0 | 0.988114472 | FALSE | 0 | CAPN1 | 23616 | 0.558052434 | gene |
| CCND3 | 3.29390681 | 0.001068495 | 0.30359086 | 0 | 3 | 3 | 5 | FALSE | 100.3333333 | 0 | 3 | 0 | 0.988114472 | FALSE | 0 | CCND3 | 23616 | 0.558052434 | gene |
| FKBP1A | 3.29390681 | 0.001068495 | 0.30359086 | 0 | 3 | 3 | 5 | FALSE | 100.3333333 | 0 | 3 | 0 | 0.988114472 | FALSE | 0 | FKBP1A | 23616 | 0.558052434 | gene |
| TRPV1 | 3.29390681 | 0.001068495 | 0.30359086 | 0 | 3 | 3 | 5 | FALSE | 100.3333333 | 0 | 3 | 0 | 0.988114472 | FALSE | 0 | TRPV1 | 23616 | 0.558052434 | gene |
| CTSK | 3.29390681 | 0.001068495 | 0.30359086 | 0 | 3 | 3 | 5 | FALSE | 100.3333333 | 0 | 3 | 0 | 0.988114472 | FALSE | 0 | CTSK | 23616 | 0.558052434 | gene |
| MAPK11 | 3.29390681 | 0.001068495 | 0.30359086 | 0 | 3 | 3 | 5 | FALSE | 100.3333333 | 0 | 3 | 0 | 0.988114472 | FALSE | 0 | MAPK11 | 23616 | 0.558052434 | gene |
| PIK3CG | 2.921146953 | 0.001369844 | 0.342331288 | 0 | 3 | 3 | 5 | FALSE | 145.5 | 0 | 3 | 1 | 0.990045871 | FALSE | 0 | PIK3CG | 29826 | 0.543233083 | gene |
| MAPK14 | 3.29390681 | 0.001068495 | 0.30359086 | 0 | 3 | 3 | 5 | FALSE | 100.3333333 | 0 | 3 | 0 | 0.988114472 | FALSE | 0 | MAPK14 | 23616 | 0.558052434 | gene |
| PFKFB3 | 3.114695341 | 7.31E-04 | 0.321058688 | 0 | 3 | 3 | 5 | FALSE | 116 | 0 | 3 | 1 | 0.989043029 | FALSE | 0 | PFKFB3 | 22540 | 0.547619048 | gene |
| PDE5A | 3.29390681 | 0.001068495 | 0.30359086 | 0 | 3 | 3 | 5 | FALSE | 100.3333333 | 0 | 3 | 0 | 0.988114472 | FALSE | 0 | PDE5A | 23616 | 0.558052434 | gene |
| CRHR1 | 3.29390681 | 0.001068495 | 0.30359086 | 0 | 3 | 3 | 5 | FALSE | 100.3333333 | 0 | 3 | 0 | 0.988114472 | FALSE | 0 | CRHR1 | 23616 | 0.558052434 | gene |
| HCRTR1 | 3.29390681 | 0.001068495 | 0.30359086 | 0 | 3 | 3 | 5 | FALSE | 100.3333333 | 0 | 3 | 0 | 0.988114472 | FALSE | 0 | HCRTR1 | 23616 | 0.558052434 | gene |
| HCRTR2 | 3.29390681 | 0.001068495 | 0.30359086 | 0 | 3 | 3 | 5 | FALSE | 100.3333333 | 0 | 3 | 0 | 0.988114472 | FALSE | 0 | HCRTR2 | 23616 | 0.558052434 | gene |
| PDE10A | 3.29390681 | 0.001068495 | 0.30359086 | 0 | 3 | 3 | 5 | FALSE | 100.3333333 | 0 | 3 | 0 | 0.988114472 | FALSE | 0 | PDE10A | 23616 | 0.558052434 | gene |
| ADORA2B | 3.29390681 | 0.001068495 | 0.30359086 | 0 | 3 | 3 | 5 | FALSE | 100.3333333 | 0 | 3 | 0 | 0.988114472 | FALSE | 0 | ADORA2B | 23616 | 0.558052434 | gene |
| PGGT1B | 3.200716846 | 0.001321012 | 0.312430011 | 0 | 3 | 3 | 5 | FALSE | 101.3333333 | 0 | 3 | 0 | 0.988597322 | FALSE | 0 | PGGT1B | 32886 | 0.539426523 | gene |
| CCR1 | 3.200716846 | 0.001321012 | 0.312430011 | 0 | 3 | 3 | 5 | FALSE | 101.3333333 | 0 | 3 | 0 | 0.988597322 | FALSE | 0 | CCR1 | 32886 | 0.539426523 | gene |
| FDFT1 | 3.387096774 | 1.62E-04 | 0.295238095 | 0 | 3 | 3 | 5 | FALSE | 83.5 | 0 | 3 | 1 | 0.987631623 | FALSE | 0 | FDFT1 | 5134 | 0.615671642 | gene |
| PTGFR | 3.200716846 | 0.001321012 | 0.312430011 | 0 | 3 | 3 | 5 | FALSE | 101.3333333 | 0 | 3 | 0 | 0.988597322 | FALSE | 0 | PTGFR | 32886 | 0.539426523 | gene |
| GPBAR1 | 3.200716846 | 0.001321012 | 0.312430011 | 0 | 3 | 3 | 5 | FALSE | 101.3333333 | 0 | 3 | 0 | 0.988597322 | FALSE | 0 | GPBAR1 | 32886 | 0.539426523 | gene |
| AGTR1 | 3.200716846 | 0.001321012 | 0.312430011 | 0 | 3 | 3 | 5 | FALSE | 101.3333333 | 0 | 3 | 0 | 0.988597322 | FALSE | 0 | AGTR1 | 32886 | 0.539426523 | gene |
| PTGDR2 | 3.200716846 | 0.001321012 | 0.312430011 | 0 | 3 | 3 | 5 | FALSE | 101.3333333 | 0 | 3 | 0 | 0.988597322 | FALSE | 0 | PTGDR2 | 32886 | 0.539426523 | gene |
| FNTA | 3.200716846 | 0.001321012 | 0.312430011 | 0 | 3 | 3 | 5 | FALSE | 101.3333333 | 0 | 3 | 0 | 0.988597322 | FALSE | 0 | FNTA | 32886 | 0.539426523 | gene |
| PREP | 3.200716846 | 0.001321012 | 0.312430011 | 0 | 3 | 3 | 5 | FALSE | 101.3333333 | 0 | 3 | 0 | 0.988597322 | FALSE | 0 | PREP | 32886 | 0.539426523 | gene |
| PTGES | 3.200716846 | 0.001321012 | 0.312430011 | 0 | 3 | 3 | 5 | FALSE | 101.3333333 | 0 | 3 | 0 | 0.988597322 | FALSE | 0 | PTGES | 32886 | 0.539426523 | gene |
| CDC25B | 3.387096774 | 1.62E-04 | 0.295238095 | 0 | 3 | 3 | 5 | FALSE | 83.5 | 0 | 3 | 1 | 0.987631623 | FALSE | 0 | CDC25B | 5134 | 0.615671642 | gene |
| PTPN2 | 3.200716846 | 0.001321012 | 0.312430011 | 0 | 3 | 3 | 5 | FALSE | 101.3333333 | 0 | 3 | 0 | 0.988597322 | FALSE | 0 | PTPN2 | 32886 | 0.539426523 | gene |
| Bingpian | 3.197132616 | 0.001998373 | 0.312780269 | 0 | 2 | 2 | 5 | FALSE | 99.5 | 0 | 2 | 0 | 0.988615893 | FALSE | 0 | Bingpian | 19436 | 0.521164021 | drug |
| YWHAG | 3.440860215 | 3.33E-06 | 0.290625 | 0 | 2 | 2 | 5 | FALSE | 109.5 | 0 | 2 | 0 | 0.987353056 | FALSE | 0 | YWHAG | 244 | 0.904166667 | gene |
| WEE1 | 3.440860215 | 3.33E-06 | 0.290625 | 0 | 2 | 2 | 5 | FALSE | 109.5 | 0 | 2 | 0 | 0.987353056 | FALSE | 0 | WEE1 | 244 | 0.904166667 | gene |
| TAS2R31 | 3.440860215 | 3.33E-06 | 0.290625 | 0 | 2 | 2 | 5 | FALSE | 109.5 | 0 | 2 | 0 | 0.987353056 | FALSE | 0 | TAS2R31 | 244 | 0.904166667 | gene |
| SLC5A2 | 3.440860215 | 3.33E-06 | 0.290625 | 0 | 2 | 2 | 5 | FALSE | 109.5 | 0 | 2 | 0 | 0.987353056 | FALSE | 0 | SLC5A2 | 244 | 0.904166667 | gene |
| SIRT2 | 3.440860215 | 3.33E-06 | 0.290625 | 0 | 2 | 2 | 5 | FALSE | 109.5 | 0 | 2 | 0 | 0.987353056 | FALSE | 0 | SIRT2 | 244 | 0.904166667 | gene |
| PLA2G5 | 3.440860215 | 3.33E-06 | 0.290625 | 0 | 2 | 2 | 5 | FALSE | 109.5 | 0 | 2 | 0 | 0.987353056 | FALSE | 0 | PLA2G5 | 244 | 0.904166667 | gene |
| PLA2G2A | 3.440860215 | 3.33E-06 | 0.290625 | 0 | 2 | 2 | 5 | FALSE | 109.5 | 0 | 2 | 0 | 0.987353056 | FALSE | 0 | PLA2G2A | 244 | 0.904166667 | gene |
| PLA2G10 | 3.440860215 | 3.33E-06 | 0.290625 | 0 | 2 | 2 | 5 | FALSE | 109.5 | 0 | 2 | 0 | 0.987353056 | FALSE | 0 | PLA2G10 | 244 | 0.904166667 | gene |
| PGF | 3.440860215 | 3.33E-06 | 0.290625 | 0 | 2 | 2 | 5 | FALSE | 109.5 | 0 | 2 | 0 | 0.987353056 | FALSE | 0 | PGF | 244 | 0.904166667 | gene |
| NQO2 | 3.440860215 | 3.33E-06 | 0.290625 | 0 | 2 | 2 | 5 | FALSE | 109.5 | 0 | 2 | 0 | 0.987353056 | FALSE | 0 | NQO2 | 244 | 0.904166667 | gene |
| KLK2 | 3.440860215 | 3.33E-06 | 0.290625 | 0 | 2 | 2 | 5 | FALSE | 109.5 | 0 | 2 | 0 | 0.987353056 | FALSE | 0 | KLK2 | 244 | 0.904166667 | gene |
| KLK1 | 3.440860215 | 3.33E-06 | 0.290625 | 0 | 2 | 2 | 5 | FALSE | 109.5 | 0 | 2 | 0 | 0.987353056 | FALSE | 0 | KLK1 | 244 | 0.904166667 | gene |
| HSD17B14 | 3.440860215 | 3.33E-06 | 0.290625 | 0 | 2 | 2 | 5 | FALSE | 109.5 | 0 | 2 | 0 | 0.987353056 | FALSE | 0 | HSD17B14 | 244 | 0.904166667 | gene |
| HNF4A | 3.440860215 | 3.33E-06 | 0.290625 | 0 | 2 | 2 | 5 | FALSE | 109.5 | 0 | 2 | 0 | 0.987353056 | FALSE | 0 | HNF4A | 244 | 0.904166667 | gene |
| GSR | 3.315412186 | 1.33E-04 | 0.301621622 | 0 | 2 | 2 | 5 | FALSE | 95.5 | 0 | 2 | 0 | 0.988003046 | FALSE | 0 | GSR | 4976 | 0.605769231 | gene |
| ESRRB | 3.440860215 | 3.33E-06 | 0.290625 | 0 | 2 | 2 | 5 | FALSE | 109.5 | 0 | 2 | 0 | 0.987353056 | FALSE | 0 | ESRRB | 244 | 0.904166667 | gene |
| ERN1 | 3.440860215 | 3.33E-06 | 0.290625 | 0 | 2 | 2 | 5 | FALSE | 109.5 | 0 | 2 | 0 | 0.987353056 | FALSE | 0 | ERN1 | 244 | 0.904166667 | gene |
| DYRK1B | 3.440860215 | 3.33E-06 | 0.290625 | 0 | 2 | 2 | 5 | FALSE | 109.5 | 0 | 2 | 0 | 0.987353056 | FALSE | 0 | DYRK1B | 244 | 0.904166667 | gene |
| DYRK1A | 3.440860215 | 3.33E-06 | 0.290625 | 0 | 2 | 2 | 5 | FALSE | 109.5 | 0 | 2 | 0 | 0.987353056 | FALSE | 0 | DYRK1A | 244 | 0.904166667 | gene |
| CYP2C9 | 3.440860215 | 3.33E-06 | 0.290625 | 0 | 2 | 2 | 5 | FALSE | 109.5 | 0 | 2 | 0 | 0.987353056 | FALSE | 0 | CYP2C9 | 244 | 0.904166667 | gene |
| CLK1 | 3.440860215 | 3.33E-06 | 0.290625 | 0 | 2 | 2 | 5 | FALSE | 109.5 | 0 | 2 | 0 | 0.987353056 | FALSE | 0 | CLK1 | 244 | 0.904166667 | gene |
| CHRNA7 | 3.440860215 | 3.33E-06 | 0.290625 | 0 | 2 | 2 | 5 | FALSE | 109.5 | 0 | 2 | 0 | 0.987353056 | FALSE | 0 | CHRNA7 | 244 | 0.904166667 | gene |
| CES1 | 3.440860215 | 3.33E-06 | 0.290625 | 0 | 2 | 2 | 5 | FALSE | 109.5 | 0 | 2 | 0 | 0.987353056 | FALSE | 0 | CES1 | 244 | 0.904166667 | gene |
| CDK4 | 3.440860215 | 3.33E-06 | 0.290625 | 0 | 2 | 2 | 5 | FALSE | 109.5 | 0 | 2 | 0 | 0.987353056 | FALSE | 0 | CDK4 | 244 | 0.904166667 | gene |
| ADORA3 | 3.440860215 | 3.33E-06 | 0.290625 | 0 | 2 | 2 | 5 | FALSE | 109.5 | 0 | 2 | 0 | 0.987353056 | FALSE | 0 | ADORA3 | 244 | 0.904166667 | gene |
| THBD | 3.189964158 | 0 | 0.313483146 | 0 | 2 | 2 | 5 | FALSE | 193 | 0 | 2 | 1 | 0.988653035 | FALSE | 0 | THBD | 0 | 0 | gene |
| SULT1E1 | 3.189964158 | 0 | 0.313483146 | 0 | 2 | 2 | 5 | FALSE | 193 | 0 | 2 | 1 | 0.988653035 | FALSE | 0 | SULT1E1 | 0 | 0 | gene |
| RAF1 | 3.189964158 | 0 | 0.313483146 | 0 | 2 | 2 | 5 | FALSE | 193 | 0 | 2 | 1 | 0.988653035 | FALSE | 0 | RAF1 | 0 | 0 | gene |
| POR | 3.189964158 | 0 | 0.313483146 | 0 | 2 | 2 | 5 | FALSE | 193 | 0 | 2 | 1 | 0.988653035 | FALSE | 0 | POR | 0 | 0 | gene |
| PLAT | 3.189964158 | 0 | 0.313483146 | 0 | 2 | 2 | 5 | FALSE | 193 | 0 | 2 | 1 | 0.988653035 | FALSE | 0 | PLAT | 0 | 0 | gene |
| ODC1 | 3.189964158 | 0 | 0.313483146 | 0 | 2 | 2 | 5 | FALSE | 193 | 0 | 2 | 1 | 0.988653035 | FALSE | 0 | ODC1 | 0 | 0 | gene |
| NQO1 | 3.189964158 | 0 | 0.313483146 | 0 | 2 | 2 | 5 | FALSE | 193 | 0 | 2 | 1 | 0.988653035 | FALSE | 0 | NQO1 | 0 | 0 | gene |
| NPEPPS | 3.189964158 | 0 | 0.313483146 | 0 | 2 | 2 | 5 | FALSE | 193 | 0 | 2 | 1 | 0.988653035 | FALSE | 0 | NPEPPS | 0 | 0 | gene |
| NOS3 | 3.189964158 | 0 | 0.313483146 | 0 | 2 | 2 | 5 | FALSE | 193 | 0 | 2 | 1 | 0.988653035 | FALSE | 0 | NOS3 | 0 | 0 | gene |
| NCF1 | 3.189964158 | 0 | 0.313483146 | 0 | 2 | 2 | 5 | FALSE | 193 | 0 | 2 | 1 | 0.988653035 | FALSE | 0 | NCF1 | 0 | 0 | gene |
| MYLK | 3.189964158 | 0 | 0.313483146 | 0 | 2 | 2 | 5 | FALSE | 193 | 0 | 2 | 1 | 0.988653035 | FALSE | 0 | MYLK | 0 | 0 | gene |
| MGAM | 3.189964158 | 0 | 0.313483146 | 0 | 2 | 2 | 5 | FALSE | 193 | 0 | 2 | 1 | 0.988653035 | FALSE | 0 | MGAM | 0 | 0 | gene |
| KDM4E | 3.189964158 | 0 | 0.313483146 | 0 | 2 | 2 | 5 | FALSE | 193 | 0 | 2 | 1 | 0.988653035 | FALSE | 0 | KDM4E | 0 | 0 | gene |
| IL1B | 3.189964158 | 0 | 0.313483146 | 0 | 2 | 2 | 5 | FALSE | 193 | 0 | 2 | 1 | 0.988653035 | FALSE | 0 | IL1B | 0 | 0 | gene |
| IL1A | 3.189964158 | 0 | 0.313483146 | 0 | 2 | 2 | 5 | FALSE | 193 | 0 | 2 | 1 | 0.988653035 | FALSE | 0 | IL1A | 0 | 0 | gene |
| HSPB1 | 3.189964158 | 0 | 0.313483146 | 0 | 2 | 2 | 5 | FALSE | 193 | 0 | 2 | 1 | 0.988653035 | FALSE | 0 | HSPB1 | 0 | 0 | gene |
| HSPA5 | 3.189964158 | 0 | 0.313483146 | 0 | 2 | 2 | 5 | FALSE | 193 | 0 | 2 | 1 | 0.988653035 | FALSE | 0 | HSPA5 | 0 | 0 | gene |
| HSF1 | 3.189964158 | 0 | 0.313483146 | 0 | 2 | 2 | 5 | FALSE | 193 | 0 | 2 | 1 | 0.988653035 | FALSE | 0 | HSF1 | 0 | 0 | gene |
| HIF1A | 3.189964158 | 0 | 0.313483146 | 0 | 2 | 2 | 5 | FALSE | 193 | 0 | 2 | 1 | 0.988653035 | FALSE | 0 | HIF1A | 0 | 0 | gene |
| GJA1 | 3.189964158 | 0 | 0.313483146 | 0 | 2 | 2 | 5 | FALSE | 193 | 0 | 2 | 1 | 0.988653035 | FALSE | 0 | GJA1 | 0 | 0 | gene |
| EIF6 | 3.189964158 | 0 | 0.313483146 | 0 | 2 | 2 | 5 | FALSE | 193 | 0 | 2 | 1 | 0.988653035 | FALSE | 0 | EIF6 | 0 | 0 | gene |
| EGF | 3.189964158 | 0 | 0.313483146 | 0 | 2 | 2 | 5 | FALSE | 193 | 0 | 2 | 1 | 0.988653035 | FALSE | 0 | EGF | 0 | 0 | gene |
| CXCL10 | 3.189964158 | 0 | 0.313483146 | 0 | 2 | 2 | 5 | FALSE | 193 | 0 | 2 | 1 | 0.988653035 | FALSE | 0 | CXCL10 | 0 | 0 | gene |
| CTSD | 3.189964158 | 0 | 0.313483146 | 0 | 2 | 2 | 5 | FALSE | 193 | 0 | 2 | 1 | 0.988653035 | FALSE | 0 | CTSD | 0 | 0 | gene |
| CRP | 3.189964158 | 0 | 0.313483146 | 0 | 2 | 2 | 5 | FALSE | 193 | 0 | 2 | 1 | 0.988653035 | FALSE | 0 | CRP | 0 | 0 | gene |
| COL3A1 | 3.189964158 | 0 | 0.313483146 | 0 | 2 | 2 | 5 | FALSE | 193 | 0 | 2 | 1 | 0.988653035 | FALSE | 0 | COL3A1 | 0 | 0 | gene |
| COL1A1 | 3.189964158 | 0 | 0.313483146 | 0 | 2 | 2 | 5 | FALSE | 193 | 0 | 2 | 1 | 0.988653035 | FALSE | 0 | COL1A1 | 0 | 0 | gene |
| CHUK | 3.189964158 | 0 | 0.313483146 | 0 | 2 | 2 | 5 | FALSE | 193 | 0 | 2 | 1 | 0.988653035 | FALSE | 0 | CHUK | 0 | 0 | gene |
| CHEK2 | 3.189964158 | 0 | 0.313483146 | 0 | 2 | 2 | 5 | FALSE | 193 | 0 | 2 | 1 | 0.988653035 | FALSE | 0 | CHEK2 | 0 | 0 | gene |
| CCL2 | 3.189964158 | 0 | 0.313483146 | 0 | 2 | 2 | 5 | FALSE | 193 | 0 | 2 | 1 | 0.988653035 | FALSE | 0 | CCL2 | 0 | 0 | gene |
| APEX1 | 3.189964158 | 0 | 0.313483146 | 0 | 2 | 2 | 5 | FALSE | 193 | 0 | 2 | 1 | 0.988653035 | FALSE | 0 | APEX1 | 0 | 0 | gene |
| ACPP | 3.189964158 | 0 | 0.313483146 | 0 | 2 | 2 | 5 | FALSE | 193 | 0 | 2 | 1 | 0.988653035 | FALSE | 0 | ACPP | 0 | 0 | gene |
| ACACA | 3.189964158 | 0 | 0.313483146 | 0 | 2 | 2 | 5 | FALSE | 193 | 0 | 2 | 1 | 0.988653035 | FALSE | 0 | ACACA | 0 | 0 | gene |
| IDO1 | 3.566308244 | 3.45E-07 | 0.28040201 | 0 | 2 | 2 | 5 | FALSE | 101.5 | 0 | 2 | 0 | 0.986703066 | FALSE | 0 | IDO1 | 30 | 0.975728155 | gene |
| TACR3 | 3.566308244 | 3.45E-07 | 0.28040201 | 0 | 2 | 2 | 5 | FALSE | 101.5 | 0 | 2 | 0 | 0.986703066 | FALSE | 0 | TACR3 | 30 | 0.975728155 | gene |
| NMBR | 3.566308244 | 3.45E-07 | 0.28040201 | 0 | 2 | 2 | 5 | FALSE | 101.5 | 0 | 2 | 0 | 0.986703066 | FALSE | 0 | NMBR | 30 | 0.975728155 | gene |
| GRM4 | 3.566308244 | 3.45E-07 | 0.28040201 | 0 | 2 | 2 | 5 | FALSE | 101.5 | 0 | 2 | 0 | 0.986703066 | FALSE | 0 | GRM4 | 30 | 0.975728155 | gene |
| TDO2 | 3.566308244 | 3.45E-07 | 0.28040201 | 0 | 2 | 2 | 5 | FALSE | 101.5 | 0 | 2 | 0 | 0.986703066 | FALSE | 0 | TDO2 | 30 | 0.975728155 | gene |
| SCARB1 | 3.566308244 | 3.45E-07 | 0.28040201 | 0 | 2 | 2 | 5 | FALSE | 101.5 | 0 | 2 | 0 | 0.986703066 | FALSE | 0 | SCARB1 | 30 | 0.975728155 | gene |
| PTAFR | 3.566308244 | 3.45E-07 | 0.28040201 | 0 | 2 | 2 | 5 | FALSE | 101.5 | 0 | 2 | 0 | 0.986703066 | FALSE | 0 | PTAFR | 30 | 0.975728155 | gene |
| BCL2A1 | 3.566308244 | 3.45E-07 | 0.28040201 | 0 | 2 | 2 | 5 | FALSE | 101.5 | 0 | 2 | 0 | 0.986703066 | FALSE | 0 | BCL2A1 | 30 | 0.975728155 | gene |
| BCL2L10 | 3.566308244 | 3.45E-07 | 0.28040201 | 0 | 2 | 2 | 5 | FALSE | 101.5 | 0 | 2 | 0 | 0.986703066 | FALSE | 0 | BCL2L10 | 30 | 0.975728155 | gene |
| BCL2L2 | 3.566308244 | 3.45E-07 | 0.28040201 | 0 | 2 | 2 | 5 | FALSE | 101.5 | 0 | 2 | 0 | 0.986703066 | FALSE | 0 | BCL2L2 | 30 | 0.975728155 | gene |
| BAD | 3.566308244 | 3.45E-07 | 0.28040201 | 0 | 2 | 2 | 5 | FALSE | 101.5 | 0 | 2 | 0 | 0.986703066 | FALSE | 0 | BAD | 30 | 0.975728155 | gene |
| PDE4C | 3.566308244 | 3.45E-07 | 0.28040201 | 0 | 2 | 2 | 5 | FALSE | 101.5 | 0 | 2 | 0 | 0.986703066 | FALSE | 0 | PDE4C | 30 | 0.975728155 | gene |
| PDE4B | 3.566308244 | 3.45E-07 | 0.28040201 | 0 | 2 | 2 | 5 | FALSE | 101.5 | 0 | 2 | 0 | 0.986703066 | FALSE | 0 | PDE4B | 30 | 0.975728155 | gene |
| SLC6A9 | 3.566308244 | 3.45E-07 | 0.28040201 | 0 | 2 | 2 | 5 | FALSE | 101.5 | 0 | 2 | 0 | 0.986703066 | FALSE | 0 | SLC6A9 | 30 | 0.975728155 | gene |
| MTNR1B | 3.566308244 | 3.45E-07 | 0.28040201 | 0 | 2 | 2 | 5 | FALSE | 101.5 | 0 | 2 | 0 | 0.986703066 | FALSE | 0 | MTNR1B | 30 | 0.975728155 | gene |
| MTNR1A | 3.566308244 | 3.45E-07 | 0.28040201 | 0 | 2 | 2 | 5 | FALSE | 101.5 | 0 | 2 | 0 | 0.986703066 | FALSE | 0 | MTNR1A | 30 | 0.975728155 | gene |
| KCNN4 | 3.566308244 | 3.45E-07 | 0.28040201 | 0 | 2 | 2 | 5 | FALSE | 101.5 | 0 | 2 | 0 | 0.986703066 | FALSE | 0 | KCNN4 | 30 | 0.975728155 | gene |
| CTSV | 3.566308244 | 3.45E-07 | 0.28040201 | 0 | 2 | 2 | 5 | FALSE | 101.5 | 0 | 2 | 0 | 0.986703066 | FALSE | 0 | CTSV | 30 | 0.975728155 | gene |
| SCN2A | 3.566308244 | 3.45E-07 | 0.28040201 | 0 | 2 | 2 | 5 | FALSE | 101.5 | 0 | 2 | 0 | 0.986703066 | FALSE | 0 | SCN2A | 30 | 0.975728155 | gene |
| ABL1 | 3.566308244 | 3.45E-07 | 0.28040201 | 0 | 2 | 2 | 5 | FALSE | 101.5 | 0 | 2 | 0 | 0.986703066 | FALSE | 0 | ABL1 | 30 | 0.975728155 | gene |
| PABPC1 | 3.566308244 | 3.45E-07 | 0.28040201 | 0 | 2 | 2 | 5 | FALSE | 101.5 | 0 | 2 | 0 | 0.986703066 | FALSE | 0 | PABPC1 | 30 | 0.975728155 | gene |
| MMP7 | 3.566308244 | 3.45E-07 | 0.28040201 | 0 | 2 | 2 | 5 | FALSE | 101.5 | 0 | 2 | 0 | 0.986703066 | FALSE | 0 | MMP7 | 30 | 0.975728155 | gene |
| SLC2A2 | 3.566308244 | 3.45E-07 | 0.28040201 | 0 | 2 | 2 | 5 | FALSE | 101.5 | 0 | 2 | 0 | 0.986703066 | FALSE | 0 | SLC2A2 | 30 | 0.975728155 | gene |
| SLC2A3 | 3.566308244 | 3.45E-07 | 0.28040201 | 0 | 2 | 2 | 5 | FALSE | 101.5 | 0 | 2 | 0 | 0.986703066 | FALSE | 0 | SLC2A3 | 30 | 0.975728155 | gene |
| CMA1 | 3.566308244 | 3.45E-07 | 0.28040201 | 0 | 2 | 2 | 5 | FALSE | 101.5 | 0 | 2 | 0 | 0.986703066 | FALSE | 0 | CMA1 | 30 | 0.975728155 | gene |
| ADAM17 | 3.566308244 | 3.45E-07 | 0.28040201 | 0 | 2 | 2 | 5 | FALSE | 101.5 | 0 | 2 | 0 | 0.986703066 | FALSE | 0 | ADAM17 | 30 | 0.975728155 | gene |
| SLC2A1 | 3.566308244 | 3.45E-07 | 0.28040201 | 0 | 2 | 2 | 5 | FALSE | 101.5 | 0 | 2 | 0 | 0.986703066 | FALSE | 0 | SLC2A1 | 30 | 0.975728155 | gene |
| TRPA1 | 3.566308244 | 3.45E-07 | 0.28040201 | 0 | 2 | 2 | 5 | FALSE | 101.5 | 0 | 2 | 0 | 0.986703066 | FALSE | 0 | TRPA1 | 30 | 0.975728155 | gene |
| TRPV4 | 3.566308244 | 3.45E-07 | 0.28040201 | 0 | 2 | 2 | 5 | FALSE | 101.5 | 0 | 2 | 0 | 0.986703066 | FALSE | 0 | TRPV4 | 30 | 0.975728155 | gene |
| TRPC3 | 3.566308244 | 3.45E-07 | 0.28040201 | 0 | 2 | 2 | 5 | FALSE | 101.5 | 0 | 2 | 0 | 0.986703066 | FALSE | 0 | TRPC3 | 30 | 0.975728155 | gene |
| TRPC6 | 3.566308244 | 3.45E-07 | 0.28040201 | 0 | 2 | 2 | 5 | FALSE | 101.5 | 0 | 2 | 0 | 0.986703066 | FALSE | 0 | TRPC6 | 30 | 0.975728155 | gene |
| MEN1 | 3.566308244 | 3.45E-07 | 0.28040201 | 0 | 2 | 2 | 5 | FALSE | 101.5 | 0 | 2 | 0 | 0.986703066 | FALSE | 0 | MEN1 | 30 | 0.975728155 | gene |
| CPB1 | 3.566308244 | 3.45E-07 | 0.28040201 | 0 | 2 | 2 | 5 | FALSE | 101.5 | 0 | 2 | 0 | 0.986703066 | FALSE | 0 | CPB1 | 30 | 0.975728155 | gene |
| SCN10A | 3.566308244 | 3.45E-07 | 0.28040201 | 0 | 2 | 2 | 5 | FALSE | 101.5 | 0 | 2 | 0 | 0.986703066 | FALSE | 0 | SCN10A | 30 | 0.975728155 | gene |
| TNFRSF1A | 3.566308244 | 3.45E-07 | 0.28040201 | 0 | 2 | 2 | 5 | FALSE | 101.5 | 0 | 2 | 0 | 0.986703066 | FALSE | 0 | TNFRSF1A | 30 | 0.975728155 | gene |
| KCNA5 | 3.566308244 | 3.45E-07 | 0.28040201 | 0 | 2 | 2 | 5 | FALSE | 101.5 | 0 | 2 | 0 | 0.986703066 | FALSE | 0 | KCNA5 | 30 | 0.975728155 | gene |
| ELOVL6 | 3.566308244 | 3.45E-07 | 0.28040201 | 0 | 2 | 2 | 5 | FALSE | 101.5 | 0 | 2 | 0 | 0.986703066 | FALSE | 0 | ELOVL6 | 30 | 0.975728155 | gene |
| GABRG2 | 3.566308244 | 3.45E-07 | 0.28040201 | 0 | 2 | 2 | 5 | FALSE | 101.5 | 0 | 2 | 0 | 0.986703066 | FALSE | 0 | GABRG2 | 30 | 0.975728155 | gene |
| P2RX7 | 3.566308244 | 3.45E-07 | 0.28040201 | 0 | 2 | 2 | 5 | FALSE | 101.5 | 0 | 2 | 0 | 0.986703066 | FALSE | 0 | P2RX7 | 30 | 0.975728155 | gene |
| ACE | 3.566308244 | 3.45E-07 | 0.28040201 | 0 | 2 | 2 | 5 | FALSE | 101.5 | 0 | 2 | 0 | 0.986703066 | FALSE | 0 | ACE | 30 | 0.975728155 | gene |
| GABRA2 | 3.566308244 | 3.45E-07 | 0.28040201 | 0 | 2 | 2 | 5 | FALSE | 101.5 | 0 | 2 | 0 | 0.986703066 | FALSE | 0 | GABRA2 | 30 | 0.975728155 | gene |
| GRM2 | 3.566308244 | 3.45E-07 | 0.28040201 | 0 | 2 | 2 | 5 | FALSE | 101.5 | 0 | 2 | 0 | 0.986703066 | FALSE | 0 | GRM2 | 30 | 0.975728155 | gene |
| CTSL | 3.566308244 | 3.45E-07 | 0.28040201 | 0 | 2 | 2 | 5 | FALSE | 101.5 | 0 | 2 | 0 | 0.986703066 | FALSE | 0 | CTSL | 30 | 0.975728155 | gene |
| CTSS | 3.566308244 | 3.45E-07 | 0.28040201 | 0 | 2 | 2 | 5 | FALSE | 101.5 | 0 | 2 | 0 | 0.986703066 | FALSE | 0 | CTSS | 30 | 0.975728155 | gene |
| PRKCD | 3.566308244 | 3.45E-07 | 0.28040201 | 0 | 2 | 2 | 5 | FALSE | 101.5 | 0 | 2 | 0 | 0.986703066 | FALSE | 0 | PRKCD | 30 | 0.975728155 | gene |
| GRK6 | 3.394265233 | 0 | 0.294614572 | 0 | 2 | 2 | 5 | FALSE | 134 | 0 | 2 | 1 | 0.987594481 | FALSE | 0 | GRK6 | 0 | 0 | gene |
| AMY1A | 3.394265233 | 0 | 0.294614572 | 0 | 2 | 2 | 5 | FALSE | 134 | 0 | 2 | 1 | 0.987594481 | FALSE | 0 | AMY1A | 0 | 0 | gene |
| UGT2B7 | 3.637992832 | 0 | 0.274876847 | 0 | 2 | 2 | 5 | FALSE | 66 | 0 | 2 | 1 | 0.986331643 | FALSE | 0 | UGT2B7 | 0 | 0 | gene |
| SHH | 3.637992832 | 0 | 0.274876847 | 0 | 2 | 2 | 5 | FALSE | 66 | 0 | 2 | 1 | 0.986331643 | FALSE | 0 | SHH | 0 | 0 | gene |
| PPP3CA | 3.394265233 | 0 | 0.294614572 | 0 | 2 | 2 | 5 | FALSE | 134 | 0 | 2 | 1 | 0.987594481 | FALSE | 0 | PPP3CA | 0 | 0 | gene |
| IKBKB | 3.394265233 | 0 | 0.294614572 | 0 | 2 | 2 | 5 | FALSE | 134 | 0 | 2 | 1 | 0.987594481 | FALSE | 0 | IKBKB | 0 | 0 | gene |
| MAP2 | 3.637992832 | 0 | 0.274876847 | 0 | 2 | 2 | 5 | FALSE | 66 | 0 | 2 | 1 | 0.986331643 | FALSE | 0 | MAP2 | 0 | 0 | gene |
| DS48 | 3.252688172 | 1.69E-05 | 0.307438017 | 0 | 2 | 2 | 5 | FALSE | 59 | 0 | 2 | 0 | 0.988328041 | FALSE | 0 | DS48 | 2726 | 0.865671642 | Danshen |
| DRD5 | 3.777777778 | 2.21E-06 | 0.264705882 | 0 | 2 | 2 | 4 | FALSE | 18.5 | 0 | 2 | 0 | 0.985607369 | FALSE | 0 | DRD5 | 34 | 0.729166667 | gene |
| EDN1 | 3.720430108 | 1.33E-05 | 0.268786127 | 0 | 2 | 2 | 4 | FALSE | 26.5 | 0 | 2 | 0 | 0.985904507 | FALSE | 0 | EDN1 | 170 | 0.6375 | gene |
| DS24 | 3.252688172 | 1.69E-05 | 0.307438017 | 0 | 2 | 2 | 5 | FALSE | 59 | 0 | 2 | 0 | 0.988328041 | FALSE | 0 | DS24 | 2726 | 0.865671642 | Danshen |
| DS12 | 3.252688172 | 1.69E-05 | 0.307438017 | 0 | 2 | 2 | 5 | FALSE | 59 | 0 | 2 | 0 | 0.988328041 | FALSE | 0 | DS12 | 2726 | 0.865671642 | Danshen |
| DS4 | 3.252688172 | 1.69E-05 | 0.307438017 | 0 | 2 | 2 | 5 | FALSE | 59 | 0 | 2 | 0 | 0.988328041 | FALSE | 0 | DS4 | 2726 | 0.865671642 | Danshen |
| DS3 | 3.424731183 | 2.10E-05 | 0.291993721 | 0 | 2 | 2 | 5 | FALSE | 35 | 0 | 2 | 0 | 0.987436626 | FALSE | 0 | DS3 | 1820 | 0.548387097 | Danshen |
| LYN | 3.326164875 | 5.10E-04 | 0.300646552 | 0 | 2 | 2 | 5 | FALSE | 85.5 | 0 | 2 | 0 | 0.987947332 | FALSE | 0 | LYN | 11912 | 0.552287582 | gene |
| ITGAL | 3.620071685 | 0 | 0.276237624 | 0 | 2 | 2 | 5 | FALSE | 98 | 0 | 2 | 1 | 0.986424499 | FALSE | 0 | ITGAL | 0 | 0 | gene |
| PIK3CD | 3.620071685 | 0 | 0.276237624 | 0 | 2 | 2 | 5 | FALSE | 98 | 0 | 2 | 1 | 0.986424499 | FALSE | 0 | PIK3CD | 0 | 0 | gene |
| CASP7 | 3.387096774 | 4.29E-04 | 0.295238095 | 0 | 2 | 2 | 5 | FALSE | 72 | 0 | 2 | 0 | 0.987631623 | FALSE | 0 | CASP7 | 12120 | 0.529850746 | gene |
| FLT4 | 3.326164875 | 5.10E-04 | 0.300646552 | 0 | 2 | 2 | 5 | FALSE | 85.5 | 0 | 2 | 0 | 0.987947332 | FALSE | 0 | FLT4 | 11912 | 0.552287582 | gene |
| PDGFRB | 3.326164875 | 5.10E-04 | 0.300646552 | 0 | 2 | 2 | 5 | FALSE | 85.5 | 0 | 2 | 0 | 0.987947332 | FALSE | 0 | PDGFRB | 11912 | 0.552287582 | gene |
| THRB | 3.197132616 | 0.001998373 | 0.312780269 | 0 | 2 | 2 | 5 | FALSE | 99.5 | 0 | 2 | 0 | 0.988615893 | FALSE | 0 | THRB | 19436 | 0.521164021 | gene |
| SCD | 3.197132616 | 0.001998373 | 0.312780269 | 0 | 2 | 2 | 5 | FALSE | 99.5 | 0 | 2 | 0 | 0.988615893 | FALSE | 0 | SCD | 19436 | 0.521164021 | gene |
| MDM2 | 3.383512545 | 3.18E-04 | 0.295550847 | 0 | 2 | 2 | 5 | FALSE | 73.5 | 0 | 2 | 0 | 0.987650194 | FALSE | 0 | MDM2 | 5706 | 0.537037037 | gene |
| MAPK3 | 3.168458781 | 5.72E-04 | 0.31561086 | 0 | 2 | 2 | 5 | FALSE | 109.5 | 0 | 2 | 0 | 0.988764462 | FALSE | 0 | MAPK3 | 13548 | 0.553571429 | gene |
| TNNC1 | 3.623655914 | 0 | 0.275964392 | 0 | 1 | 1 | 5 | FALSE | 73 | 0 | 1 | 0 | 0.986405928 | FALSE | 0 | TNNC1 | 0 | 0 | gene |
| TEK | 3.623655914 | 0 | 0.275964392 | 0 | 1 | 1 | 5 | FALSE | 73 | 0 | 1 | 0 | 0.986405928 | FALSE | 0 | TEK | 0 | 0 | gene |
| SREBF1 | 3.465949821 | 0 | 0.2885212 | 0 | 1 | 1 | 5 | FALSE | 118 | 0 | 1 | 0 | 0.987223058 | FALSE | 0 | SREBF1 | 0 | 0 | gene |
| SOAT2 | 3.465949821 | 0 | 0.2885212 | 0 | 1 | 1 | 5 | FALSE | 118 | 0 | 1 | 0 | 0.987223058 | FALSE | 0 | SOAT2 | 0 | 0 | gene |
| SOAT1 | 3.465949821 | 0 | 0.2885212 | 0 | 1 | 1 | 5 | FALSE | 118 | 0 | 1 | 0 | 0.987223058 | FALSE | 0 | SOAT1 | 0 | 0 | gene |
| PLK4 | 3.623655914 | 0 | 0.275964392 | 0 | 1 | 1 | 5 | FALSE | 73 | 0 | 1 | 0 | 0.986405928 | FALSE | 0 | PLK4 | 0 | 0 | gene |
| MTTP | 3.465949821 | 0 | 0.2885212 | 0 | 1 | 1 | 5 | FALSE | 118 | 0 | 1 | 0 | 0.987223058 | FALSE | 0 | MTTP | 0 | 0 | gene |
| LDLR | 3.465949821 | 0 | 0.2885212 | 0 | 1 | 1 | 5 | FALSE | 118 | 0 | 1 | 0 | 0.987223058 | FALSE | 0 | LDLR | 0 | 0 | gene |
| HSPA1A | 3.623655914 | 0 | 0.275964392 | 0 | 1 | 1 | 5 | FALSE | 73 | 0 | 1 | 0 | 0.986405928 | FALSE | 0 | HSPA1A | 0 | 0 | gene |
| HSD17B3 | 3.623655914 | 0 | 0.275964392 | 0 | 1 | 1 | 5 | FALSE | 73 | 0 | 1 | 0 | 0.986405928 | FALSE | 0 | HSD17B3 | 0 | 0 | gene |
| GSTA2 | 3.623655914 | 0 | 0.275964392 | 0 | 1 | 1 | 5 | FALSE | 73 | 0 | 1 | 0 | 0.986405928 | FALSE | 0 | GSTA2 | 0 | 0 | gene |
| GSTA1 | 3.623655914 | 0 | 0.275964392 | 0 | 1 | 1 | 5 | FALSE | 73 | 0 | 1 | 0 | 0.986405928 | FALSE | 0 | GSTA1 | 0 | 0 | gene |
| GOT1 | 3.465949821 | 0 | 0.2885212 | 0 | 1 | 1 | 5 | FALSE | 118 | 0 | 1 | 0 | 0.987223058 | FALSE | 0 | GOT1 | 0 | 0 | gene |
| FGR | 3.623655914 | 0 | 0.275964392 | 0 | 1 | 1 | 5 | FALSE | 73 | 0 | 1 | 0 | 0.986405928 | FALSE | 0 | FGR | 0 | 0 | gene |
| EPHB4 | 3.623655914 | 0 | 0.275964392 | 0 | 1 | 1 | 5 | FALSE | 73 | 0 | 1 | 0 | 0.986405928 | FALSE | 0 | EPHB4 | 0 | 0 | gene |
| DAO | 3.623655914 | 0 | 0.275964392 | 0 | 1 | 1 | 5 | FALSE | 73 | 0 | 1 | 0 | 0.986405928 | FALSE | 0 | DAO | 0 | 0 | gene |
| CAT | 3.465949821 | 0 | 0.2885212 | 0 | 1 | 1 | 5 | FALSE | 118 | 0 | 1 | 0 | 0.987223058 | FALSE | 0 | CAT | 0 | 0 | gene |
| BRAF | 3.623655914 | 0 | 0.275964392 | 0 | 1 | 1 | 5 | FALSE | 73 | 0 | 1 | 0 | 0.986405928 | FALSE | 0 | BRAF | 0 | 0 | gene |
| ABAT | 3.465949821 | 0 | 0.2885212 | 0 | 1 | 1 | 5 | FALSE | 118 | 0 | 1 | 0 | 0.987223058 | FALSE | 0 | ABAT | 0 | 0 | gene |
| GCGR | 3.652329749 | 0 | 0.273797841 | 0 | 1 | 1 | 5 | FALSE | 62 | 0 | 1 | 0 | 0.986257359 | FALSE | 0 | GCGR | 0 | 0 | gene |
| TBXAS1 | 3.652329749 | 0 | 0.273797841 | 0 | 1 | 1 | 5 | FALSE | 62 | 0 | 1 | 0 | 0.986257359 | FALSE | 0 | TBXAS1 | 0 | 0 | gene |
| ADRB1 | 3.652329749 | 0 | 0.273797841 | 0 | 1 | 1 | 5 | FALSE | 62 | 0 | 1 | 0 | 0.986257359 | FALSE | 0 | ADRB1 | 0 | 0 | gene |
| ITGB3 | 3.756272401 | 0 | 0.266221374 | 0 | 1 | 1 | 4 | FALSE | 32 | 0 | 1 | 0 | 0.985718796 | FALSE | 0 | ITGB3 | 0 | 0 | gene |
| CALCR | 3.756272401 | 0 | 0.266221374 | 0 | 1 | 1 | 4 | FALSE | 32 | 0 | 1 | 0 | 0.985718796 | FALSE | 0 | CALCR | 0 | 0 | gene |
| ECE1 | 3.756272401 | 0 | 0.266221374 | 0 | 1 | 1 | 4 | FALSE | 32 | 0 | 1 | 0 | 0.985718796 | FALSE | 0 | ECE1 | 0 | 0 | gene |
| ADRA2B | 3.792114695 | 0 | 0.263705104 | 0 | 1 | 1 | 4 | FALSE | 22 | 0 | 1 | 0 | 0.985533084 | FALSE | 0 | ADRA2B | 0 | 0 | gene |
| STAT3 | 3.795698925 | 0 | 0.263456091 | 0 | 1 | 1 | 4 | FALSE | 21 | 0 | 1 | 0 | 0.985514513 | FALSE | 0 | STAT3 | 0 | 0 | gene |
| ADCY2 | 3.709677419 | 0 | 0.269565217 | 0 | 1 | 1 | 5 | FALSE | 46 | 0 | 1 | 0 | 0.985960221 | FALSE | 0 | ADCY2 | 0 | 0 | gene |
| IL4 | 3.709677419 | 0 | 0.269565217 | 0 | 1 | 1 | 5 | FALSE | 46 | 0 | 1 | 0 | 0.985960221 | FALSE | 0 | IL4 | 0 | 0 | gene |
| PCNA | 3.709677419 | 0 | 0.269565217 | 0 | 1 | 1 | 5 | FALSE | 46 | 0 | 1 | 0 | 0.985960221 | FALSE | 0 | PCNA | 0 | 0 | gene |
| SCN9A | 3.620071685 | 0 | 0.276237624 | 0 | 1 | 1 | 5 | FALSE | 98 | 0 | 1 | 0 | 0.986424499 | FALSE | 0 | SCN9A | 0 | 0 | gene |
| HCK | 3.620071685 | 0 | 0.276237624 | 0 | 1 | 1 | 5 | FALSE | 98 | 0 | 1 | 0 | 0.986424499 | FALSE | 0 | HCK | 0 | 0 | gene |
| BDKRB2 | 3.620071685 | 0 | 0.276237624 | 0 | 1 | 1 | 5 | FALSE | 98 | 0 | 1 | 0 | 0.986424499 | FALSE | 0 | BDKRB2 | 0 | 0 | gene |
| PDGFRA | 3.620071685 | 0 | 0.276237624 | 0 | 1 | 1 | 5 | FALSE | 98 | 0 | 1 | 0 | 0.986424499 | FALSE | 0 | PDGFRA | 0 | 0 | gene |
| DPP4 | 3.620071685 | 0 | 0.276237624 | 0 | 1 | 1 | 5 | FALSE | 98 | 0 | 1 | 0 | 0.986424499 | FALSE | 0 | DPP4 | 0 | 0 | gene |
| PDE7A | 3.620071685 | 0 | 0.276237624 | 0 | 1 | 1 | 5 | FALSE | 98 | 0 | 1 | 0 | 0.986424499 | FALSE | 0 | PDE7A | 0 | 0 | gene |
| FLT1 | 3.620071685 | 0 | 0.276237624 | 0 | 1 | 1 | 5 | FALSE | 98 | 0 | 1 | 0 | 0.986424499 | FALSE | 0 | FLT1 | 0 | 0 | gene |
| SLC8A1 | 3.620071685 | 0 | 0.276237624 | 0 | 1 | 1 | 5 | FALSE | 98 | 0 | 1 | 0 | 0.986424499 | FALSE | 0 | SLC8A1 | 0 | 0 | gene |
| ROCK2 | 3.620071685 | 0 | 0.276237624 | 0 | 1 | 1 | 5 | FALSE | 98 | 0 | 1 | 0 | 0.986424499 | FALSE | 0 | ROCK2 | 0 | 0 | gene |
| FAP | 3.620071685 | 0 | 0.276237624 | 0 | 1 | 1 | 5 | FALSE | 98 | 0 | 1 | 0 | 0.986424499 | FALSE | 0 | FAP | 0 | 0 | gene |
| FGFR2 | 3.620071685 | 0 | 0.276237624 | 0 | 1 | 1 | 5 | FALSE | 98 | 0 | 1 | 0 | 0.986424499 | FALSE | 0 | FGFR2 | 0 | 0 | gene |
| HDAC1 | 3.620071685 | 0 | 0.276237624 | 0 | 1 | 1 | 5 | FALSE | 98 | 0 | 1 | 0 | 0.986424499 | FALSE | 0 | HDAC1 | 0 | 0 | gene |
| HDAC6 | 3.620071685 | 0 | 0.276237624 | 0 | 1 | 1 | 5 | FALSE | 98 | 0 | 1 | 0 | 0.986424499 | FALSE | 0 | HDAC6 | 0 | 0 | gene |
| BTK | 3.620071685 | 0 | 0.276237624 | 0 | 1 | 1 | 5 | FALSE | 98 | 0 | 1 | 0 | 0.986424499 | FALSE | 0 | BTK | 0 | 0 | gene |
| JAK2 | 3.620071685 | 0 | 0.276237624 | 0 | 1 | 1 | 5 | FALSE | 98 | 0 | 1 | 0 | 0.986424499 | FALSE | 0 | JAK2 | 0 | 0 | gene |
| ZAP70 | 3.620071685 | 0 | 0.276237624 | 0 | 1 | 1 | 5 | FALSE | 98 | 0 | 1 | 0 | 0.986424499 | FALSE | 0 | ZAP70 | 0 | 0 | gene |
| TNIK | 3.620071685 | 0 | 0.276237624 | 0 | 1 | 1 | 5 | FALSE | 98 | 0 | 1 | 0 | 0.986424499 | FALSE | 0 | TNIK | 0 | 0 | gene |
| LIPG | 3.620071685 | 0 | 0.276237624 | 0 | 1 | 1 | 5 | FALSE | 98 | 0 | 1 | 0 | 0.986424499 | FALSE | 0 | LIPG | 0 | 0 | gene |
| HSP90AA1 | 3.620071685 | 0 | 0.276237624 | 0 | 1 | 1 | 5 | FALSE | 98 | 0 | 1 | 0 | 0.986424499 | FALSE | 0 | HSP90AA1 | 0 | 0 | gene |
| MTOR | 3.620071685 | 0 | 0.276237624 | 0 | 1 | 1 | 5 | FALSE | 98 | 0 | 1 | 0 | 0.986424499 | FALSE | 0 | MTOR | 0 | 0 | gene |
| F10 | 3.620071685 | 0 | 0.276237624 | 0 | 1 | 1 | 5 | FALSE | 98 | 0 | 1 | 0 | 0.986424499 | FALSE | 0 | F10 | 0 | 0 | gene |
| F13A1 | 3.620071685 | 0 | 0.276237624 | 0 | 1 | 1 | 5 | FALSE | 98 | 0 | 1 | 0 | 0.986424499 | FALSE | 0 | F13A1 | 0 | 0 | gene |
| TGM1 | 3.620071685 | 0 | 0.276237624 | 0 | 1 | 1 | 5 | FALSE | 98 | 0 | 1 | 0 | 0.986424499 | FALSE | 0 | TGM1 | 0 | 0 | gene |
| TGM2 | 3.620071685 | 0 | 0.276237624 | 0 | 1 | 1 | 5 | FALSE | 98 | 0 | 1 | 0 | 0.986424499 | FALSE | 0 | TGM2 | 0 | 0 | gene |
| PDPK1 | 3.620071685 | 0 | 0.276237624 | 0 | 1 | 1 | 5 | FALSE | 98 | 0 | 1 | 0 | 0.986424499 | FALSE | 0 | PDPK1 | 0 | 0 | gene |
| CFD | 3.620071685 | 0 | 0.276237624 | 0 | 1 | 1 | 5 | FALSE | 98 | 0 | 1 | 0 | 0.986424499 | FALSE | 0 | CFD | 0 | 0 | gene |
| KNG1 | 3.620071685 | 0 | 0.276237624 | 0 | 1 | 1 | 5 | FALSE | 98 | 0 | 1 | 0 | 0.986424499 | FALSE | 0 | KNG1 | 0 | 0 | gene |
| ACACB | 3.620071685 | 0 | 0.276237624 | 0 | 1 | 1 | 5 | FALSE | 98 | 0 | 1 | 0 | 0.986424499 | FALSE | 0 | ACACB | 0 | 0 | gene |
| RPS6KA2 | 3.620071685 | 0 | 0.276237624 | 0 | 1 | 1 | 5 | FALSE | 98 | 0 | 1 | 0 | 0.986424499 | FALSE | 0 | RPS6KA2 | 0 | 0 | gene |
| CCNE2 | 3.620071685 | 0 | 0.276237624 | 0 | 1 | 1 | 5 | FALSE | 98 | 0 | 1 | 0 | 0.986424499 | FALSE | 0 | CCNE2 | 0 | 0 | gene |
| CASP6 | 3.620071685 | 0 | 0.276237624 | 0 | 1 | 1 | 5 | FALSE | 98 | 0 | 1 | 0 | 0.986424499 | FALSE | 0 | CASP6 | 0 | 0 | gene |
| RPS6KA3 | 3.620071685 | 0 | 0.276237624 | 0 | 1 | 1 | 5 | FALSE | 98 | 0 | 1 | 0 | 0.986424499 | FALSE | 0 | RPS6KA3 | 0 | 0 | gene |
| MAPKAPK2 | 3.620071685 | 0 | 0.276237624 | 0 | 1 | 1 | 5 | FALSE | 98 | 0 | 1 | 0 | 0.986424499 | FALSE | 0 | MAPKAPK2 | 0 | 0 | gene |
| PTK2B | 3.620071685 | 0 | 0.276237624 | 0 | 1 | 1 | 5 | FALSE | 98 | 0 | 1 | 0 | 0.986424499 | FALSE | 0 | PTK2B | 0 | 0 | gene |
| TAOK1 | 3.620071685 | 0 | 0.276237624 | 0 | 1 | 1 | 5 | FALSE | 98 | 0 | 1 | 0 | 0.986424499 | FALSE | 0 | TAOK1 | 0 | 0 | gene |
| GCK | 3.620071685 | 0 | 0.276237624 | 0 | 1 | 1 | 5 | FALSE | 98 | 0 | 1 | 0 | 0.986424499 | FALSE | 0 | GCK | 0 | 0 | gene |
| NR5A1 | 3.620071685 | 0 | 0.276237624 | 0 | 1 | 1 | 5 | FALSE | 98 | 0 | 1 | 0 | 0.986424499 | FALSE | 0 | NR5A1 | 0 | 0 | gene |
| NTRK1 | 3.620071685 | 0 | 0.276237624 | 0 | 1 | 1 | 5 | FALSE | 98 | 0 | 1 | 0 | 0.986424499 | FALSE | 0 | NTRK1 | 0 | 0 | gene |
| CSF1R | 3.620071685 | 0 | 0.276237624 | 0 | 1 | 1 | 5 | FALSE | 98 | 0 | 1 | 0 | 0.986424499 | FALSE | 0 | CSF1R | 0 | 0 | gene |
| PORCN | 3.620071685 | 0 | 0.276237624 | 0 | 1 | 1 | 5 | FALSE | 98 | 0 | 1 | 0 | 0.986424499 | FALSE | 0 | PORCN | 0 | 0 | gene |
| AMPD3 | 3.523297491 | 0 | 0.283825025 | 0 | 1 | 1 | 5 | FALSE | 101 | 0 | 1 | 0 | 0.98692592 | FALSE | 0 | AMPD3 | 0 | 0 | gene |
| THRA | 3.523297491 | 0 | 0.283825025 | 0 | 1 | 1 | 5 | FALSE | 101 | 0 | 1 | 0 | 0.98692592 | FALSE | 0 | THRA | 0 | 0 | gene |
| ENPP2 | 3.523297491 | 0 | 0.283825025 | 0 | 1 | 1 | 5 | FALSE | 101 | 0 | 1 | 0 | 0.98692592 | FALSE | 0 | ENPP2 | 0 | 0 | gene |
| NR1H4 | 3.523297491 | 0 | 0.283825025 | 0 | 1 | 1 | 5 | FALSE | 101 | 0 | 1 | 0 | 0.98692592 | FALSE | 0 | NR1H4 | 0 | 0 | gene |
| CASR | 3.523297491 | 0 | 0.283825025 | 0 | 1 | 1 | 5 | FALSE | 101 | 0 | 1 | 0 | 0.98692592 | FALSE | 0 | CASR | 0 | 0 | gene |
| P2RX3 | 3.523297491 | 0 | 0.283825025 | 0 | 1 | 1 | 5 | FALSE | 101 | 0 | 1 | 0 | 0.98692592 | FALSE | 0 | P2RX3 | 0 | 0 | gene |
| ITGB7 | 3.523297491 | 0 | 0.283825025 | 0 | 1 | 1 | 5 | FALSE | 101 | 0 | 1 | 0 | 0.98692592 | FALSE | 0 | ITGB7 | 0 | 0 | gene |
| REN | 3.523297491 | 0 | 0.283825025 | 0 | 1 | 1 | 5 | FALSE | 101 | 0 | 1 | 0 | 0.98692592 | FALSE | 0 | REN | 0 | 0 | gene |
| OXTR | 3.523297491 | 0 | 0.283825025 | 0 | 1 | 1 | 5 | FALSE | 101 | 0 | 1 | 0 | 0.98692592 | FALSE | 0 | OXTR | 0 | 0 | gene |
| GLUL | 3.523297491 | 0 | 0.283825025 | 0 | 1 | 1 | 5 | FALSE | 101 | 0 | 1 | 0 | 0.98692592 | FALSE | 0 | GLUL | 0 | 0 | gene |
| ITGB1 | 3.523297491 | 0 | 0.283825025 | 0 | 1 | 1 | 5 | FALSE | 101 | 0 | 1 | 0 | 0.98692592 | FALSE | 0 | ITGB1 | 0 | 0 | gene |
| CNR1 | 3.523297491 | 0 | 0.283825025 | 0 | 1 | 1 | 5 | FALSE | 101 | 0 | 1 | 0 | 0.98692592 | FALSE | 0 | CNR1 | 0 | 0 | gene |
| CTSA | 3.523297491 | 0 | 0.283825025 | 0 | 1 | 1 | 5 | FALSE | 101 | 0 | 1 | 0 | 0.98692592 | FALSE | 0 | CTSA | 0 | 0 | gene |
| PRKCH | 3.523297491 | 0 | 0.283825025 | 0 | 1 | 1 | 5 | FALSE | 101 | 0 | 1 | 0 | 0.98692592 | FALSE | 0 | PRKCH | 0 | 0 | gene |
| MMP14 | 3.523297491 | 0 | 0.283825025 | 0 | 1 | 1 | 5 | FALSE | 101 | 0 | 1 | 0 | 0.98692592 | FALSE | 0 | MMP14 | 0 | 0 | gene |
| ADAMTS5 | 3.523297491 | 0 | 0.283825025 | 0 | 1 | 1 | 5 | FALSE | 101 | 0 | 1 | 0 | 0.98692592 | FALSE | 0 | ADAMTS5 | 0 | 0 | gene |
| S1PR2 | 3.523297491 | 0 | 0.283825025 | 0 | 1 | 1 | 5 | FALSE | 101 | 0 | 1 | 0 | 0.98692592 | FALSE | 0 | S1PR2 | 0 | 0 | gene |
| EDNRB | 3.523297491 | 0 | 0.283825025 | 0 | 1 | 1 | 5 | FALSE | 101 | 0 | 1 | 0 | 0.98692592 | FALSE | 0 | EDNRB | 0 | 0 | gene |
| PTGDR | 3.523297491 | 0 | 0.283825025 | 0 | 1 | 1 | 5 | FALSE | 101 | 0 | 1 | 0 | 0.98692592 | FALSE | 0 | PTGDR | 0 | 0 | gene |
| MME | 3.523297491 | 0 | 0.283825025 | 0 | 1 | 1 | 5 | FALSE | 101 | 0 | 1 | 0 | 0.98692592 | FALSE | 0 | MME | 0 | 0 | gene |
| SIGMAR1 | 3.523297491 | 0 | 0.283825025 | 0 | 1 | 1 | 5 | FALSE | 101 | 0 | 1 | 0 | 0.98692592 | FALSE | 0 | SIGMAR1 | 0 | 0 | gene |
| AMPD2 | 3.523297491 | 0 | 0.283825025 | 0 | 1 | 1 | 5 | FALSE | 101 | 0 | 1 | 0 | 0.98692592 | FALSE | 0 | AMPD2 | 0 | 0 | gene |
| PTGIR | 3.523297491 | 0 | 0.283825025 | 0 | 1 | 1 | 5 | FALSE | 101 | 0 | 1 | 0 | 0.98692592 | FALSE | 0 | PTGIR | 0 | 0 | gene |
| PTPN11 | 3.523297491 | 0 | 0.283825025 | 0 | 1 | 1 | 5 | FALSE | 101 | 0 | 1 | 0 | 0.98692592 | FALSE | 0 | PTPN11 | 0 | 0 | gene |
| PTGER4 | 3.523297491 | 0 | 0.283825025 | 0 | 1 | 1 | 5 | FALSE | 101 | 0 | 1 | 0 | 0.98692592 | FALSE | 0 | PTGER4 | 0 | 0 | gene |
| FAAH | 3.523297491 | 0 | 0.283825025 | 0 | 1 | 1 | 5 | FALSE | 101 | 0 | 1 | 0 | 0.98692592 | FALSE | 0 | FAAH | 0 | 0 | gene |
| FABP5 | 3.523297491 | 0 | 0.283825025 | 0 | 1 | 1 | 5 | FALSE | 101 | 0 | 1 | 0 | 0.98692592 | FALSE | 0 | FABP5 | 0 | 0 | gene |
| FABP3 | 3.523297491 | 0 | 0.283825025 | 0 | 1 | 1 | 5 | FALSE | 101 | 0 | 1 | 0 | 0.98692592 | FALSE | 0 | FABP3 | 0 | 0 | gene |
| FABP4 | 3.523297491 | 0 | 0.283825025 | 0 | 1 | 1 | 5 | FALSE | 101 | 0 | 1 | 0 | 0.98692592 | FALSE | 0 | FABP4 | 0 | 0 | gene |
| FABP1 | 3.523297491 | 0 | 0.283825025 | 0 | 1 | 1 | 5 | FALSE | 101 | 0 | 1 | 0 | 0.98692592 | FALSE | 0 | FABP1 | 0 | 0 | gene |
| LTB4R | 3.523297491 | 0 | 0.283825025 | 0 | 1 | 1 | 5 | FALSE | 101 | 0 | 1 | 0 | 0.98692592 | FALSE | 0 | LTB4R | 0 | 0 | gene |
| PDE4D | 3.523297491 | 0 | 0.283825025 | 0 | 1 | 1 | 5 | FALSE | 101 | 0 | 1 | 0 | 0.98692592 | FALSE | 0 | PDE4D | 0 | 0 | gene |
| ACP1 | 3.523297491 | 0 | 0.283825025 | 0 | 1 | 1 | 5 | FALSE | 101 | 0 | 1 | 0 | 0.98692592 | FALSE | 0 | ACP1 | 0 | 0 | gene |
| PTPRF | 3.523297491 | 0 | 0.283825025 | 0 | 1 | 1 | 5 | FALSE | 101 | 0 | 1 | 0 | 0.98692592 | FALSE | 0 | PTPRF | 0 | 0 | gene |
| CD81 | 3.523297491 | 0 | 0.283825025 | 0 | 1 | 1 | 5 | FALSE | 101 | 0 | 1 | 0 | 0.98692592 | FALSE | 0 | CD81 | 0 | 0 | gene |

| Table S5. Heart Failure-related genes obtained from different databases. | | | | | | | | | | | | | | | | | |
| --- | --- | --- | --- | --- | --- | --- | --- | --- | --- | --- | --- | --- | --- | --- | --- | --- | --- |
| GeneCard | PharmGKB | OMIM | DisGeNet | TTD | GeneCard\|PharmGKB | GeneCard\|OMIM | GeneCard\|DisGeNet | GeneCard\|TTD | PharmGKB\|DisGeNet | OMIM\|DisGeNet | DisGeNet\|TTD | GeneCard\|PharmGKB\|DisGeNet | GeneCard\|OMIM\|DisGeNet | GeneCard\|DisGeNet\|TTD | GeneCard\|PharmGKB\|OMIM\|DisGeNet | GeneCard\|OMIM\|DisGeNet\|TTD | GeneCard\|PharmGKB\|OMIM\|DisGeNet\|TTD |
| NKX2-5 | CLASP1 | CVDPX | Gene | CA-II | ABCC4 | GLA | GATA4 | CACNA1C | CBR3 | ILK | CRHR2 | NOS3 | TTN | RYR1 | ADRB2 | MYBPC3 | ACE |
| TBX5 | KCNIP4 | LVNC1 | UCN2 | CXCR1 |  | TAFAZZIN | JAG1 | CACNA1D |  | PDPK1 | HTR4 | GNB3 | SCN5A | KCNH2 | GRK5 | ATP2A2 | ADRB1 |
| GATA6 | CYP4A11 | SCFI | PEBP1 | D2R |  | VCL | NPPB | ADORA1 |  | ERN1 | ADORA2B | SOD2 | MYH6 | MME | PRKCA |  | AGTR1 |
| TTN-AS1 | CES1 | JDSCD | ACACA | MR |  | PFHB2 | NPPA | CACNA1A |  | YME1L1 | HDC | CYP3A4 | LMNA | PPARA |  |  | ADRA2C |
| NODAL |  | HDCA | ROCK2 | V1AR |  | MKKS | PRKAG2 | AR |  | MIR25 | PTGER4 | VKORC1 | MYH7 | MPO |  |  |  |
| TBX1 |  | POF1 | DSTN | NET |  | KCNJ11 | TBX20 | TRPV4 |  | OMA1 |  | CAT | GJA1 | XDH |  |  |  |
| FBN1 |  | PFHB1A | CIDEA | V2R |  | HEG1 | TRPM4 | SLC12A1 |  |  |  | ABCB1 | TNNT2 | APLNR |  |  |  |
| GDF1 |  | CHTD2 | FASN | MERS |  | GET1 | IL6 | CACNA1B |  |  |  | CYP2D6 | TNNI3 | CD34 |  |  |  |
| ZIC3 |  | ATRST1 | BAMBI | ADRA1D |  | PCDH7 | MYL2 | ATP1A1 |  |  |  | RYR3 | HAND2 | PDE3A |  |  |  |
| TERT |  | MFM9 | NOX1 | ENaC |  |  | KCNQ1 | CPT1B |  |  |  | UGT1A1 | ACTC1 | NPR3 |  |  |  |
| SMAD6 |  | CHDSKM | CXCL2 | SPT ATPase |  |  | MAPK1 |  |  |  |  | NQO1 | DSP | NPR1 |  |  |  |
| GJA5 |  | MUL | APCS | TN-C |  |  | BMPR2 |  |  |  |  |  | DMD | DBH |  |  |  |
| GATA5 |  | TARPS | ACLY | ASIC1 |  |  | INS |  |  |  |  |  | RYR2 | CXCL12 |  |  |  |
| DNAH1 |  | MOSPGF | ATP2A1 | SGLT2 |  |  | REN |  |  |  |  |  | HAND1 | CHRM2 |  |  |  |
| TBX4 |  | HBMS | PRKAR2B | GUCY2D |  |  | RTEL1 |  |  |  |  |  | PLN | ADORA2A |  |  |  |
| COL2A1 |  | CHTD3 | MAP2K7 | GCS |  |  | PTPN11 |  |  |  |  |  | TTR | CYP11B1 |  |  |  |
| ZFPM2 |  | MFM1 | ELOVL6 | SLCO4C1 |  |  | ABL1 |  |  |  |  |  | AGT | THRA |  |  |  |
| NKX2-6 |  | BTHS | FIP1L1 | ADORA3 |  |  | MIR21 |  |  |  |  |  | TPM1 | CXCR2 |  |  |  |
| NR5A1 |  | SHDRA | KAT8 | CYM |  |  | DES |  |  |  |  |  | APOA1 |  |  |  |  |
| KCNJ5 |  | CMD1Y | HSF1 | OPRL1 |  |  | TNF |  |  |  |  |  | ABCC9 |  |  |  |  |
| LDB3 |  | CMD1S | ALOX15 | oral |  |  | CRP |  |  |  |  |  | CSRP3 |  |  |  |  |
| CHD7 |  | BPES | SCD | ADORA1 mRNA |  |  | EDN1 |  |  |  |  |  | SGCD |  |  |  |  |
| ELN |  | PFE | MAS1 | GUCY1B1 |  |  | FLNA |  |  |  |  |  | JPH2 |  |  |  |  |
| NR2F2 |  | DSMA1 | P2RX4 | VIPR2 |  |  | IFNG |  |  |  |  |  | STAT3 |  |  |  |  |
| FLNC |  | ODG2 | FXYD1 | SGLT1 |  |  | ALB |  |  |  |  |  | GRK2 |  |  |  |  |
| MAP2K1 |  | EPM4 | KCNK2 | HSP70 |  |  | CDK8 |  |  |  |  |  | EYA4 |  |  |  |  |
| TRMU |  | SRXY3 | ARRB1 | Pro-NRG1 |  |  | RBM20 |  |  |  |  |  | PSEN2 |  |  |  |  |
| KCNJ2 |  | SPGF10 | PPP1CC | RXFP1 |  |  | NOTCH1 |  |  |  |  |  | FKTN |  |  |  |  |
| RTEL1-TNFRSF6B |  | SPGFY2 | DDR1 | mTORC1 |  |  | IL10 |  |  |  |  |  | CASP3 |  |  |  |  |
| SCN1B |  | SPGF17 | DYNLL1 | NKCC |  |  | F2 |  |  |  |  |  | GNAQ |  |  |  |  |
| TAB2 |  | RFH1 | CASP12 | CACNA1E |  |  | ENG |  |  |  |  |  | CAMK2D |  |  |  |  |
| BRAF |  | CMD1P | CHRM4 | NaC |  |  | APOE |  |  |  |  |  | SRF |  |  |  |  |
| KRAS |  | CMD1U | CHRNA4 | ADRA1 |  |  | TP53 |  |  |  |  |  | UTS2 |  |  |  |  |
| CITED2 |  | CMD1D | DIO3 | PDE3 |  |  | NBAS |  |  |  |  |  | CMA1 |  |  |  |  |
| APOB |  | GRNG | ACAN | CaC |  |  | TGFB1 |  |  |  |  |  | IL6ST |  |  |  |  |
| B3GAT3 |  | NEDBEH | ENDOG | VR |  |  | BAG3 |  |  |  |  |  | NRG1 |  |  |  |  |
| FABP3 |  | CMH4 | NPTXR | PDE |  |  | HCN4 |  |  |  |  |  | OPA1 |  |  |  |  |
| ERCC6 |  | CDSP | ALOX12 | ECE |  |  | DSG2 |  |  |  |  |  | EPHX2 |  |  |  |  |
| BRF1 |  | CCHS1 | BIRC5 | T-cells |  |  | LDLR |  |  |  |  |  | XBP1 |  |  |  |  |
| PIGL |  | GPHYSD1 | MMP13 | MIR92 |  |  | PKP2 |  |  |  |  |  | CDK9 |  |  |  |  |
| FMR1 |  | MCOPS9 | NGFR | ADCY |  |  | MIR210 |  |  |  |  |  | SUMO1 |  |  |  |  |
| ACTN2 |  | CMD1I | PLCD1 |  |  |  | HLA-DRB1 |  |  |  |  |  |  |  |  |  |  |
| PARN |  | JLNS1 | PLD2 |  |  |  | CD36 |  |  |  |  |  |  |  |  |  |  |
| ABCC8 |  | CMD1G | MED1 |  |  |  | PPARG |  |  |  |  |  |  |  |  |  |  |
| MTHFR |  | CMD1J | GRK1 |  |  |  | MYL3 |  |  |  |  |  |  |  |  |  |  |
| MAP2K2 |  | GACI2 | FOSL1 |  |  |  | PITX2 |  |  |  |  |  |  |  |  |  |  |
| RPL5 |  | CMD2B | OGT |  |  |  | VWF |  |  |  |  |  |  |  |  |  |  |
| MYPN |  | HTX1 | BECN1 |  |  |  | KCNE1 |  |  |  |  |  |  |  |  |  |  |
| CERS1 |  | CMD1A | TOMM70 |  |  |  | SERPINE1 |  |  |  |  |  |  |  |  |  |  |
| PMS2 |  | HFE2A | ZGLP1 |  |  |  | NR3C2 |  |  |  |  |  |  |  |  |  |  |
| COL4A5 |  | CMD2A | C8orf37-AS1 |  |  |  | PKD2 |  |  |  |  |  |  |  |  |  |  |
| TMEM43 |  | MFS | CERT1 |  |  |  | IGF1 |  |  |  |  |  |  |  |  |  |  |
| RAF1 |  | HHT1 | LINC01782 |  |  |  | VEGFA |  |  |  |  |  |  |  |  |  |  |
| CAV1 |  | MTTL1 | LIPC-AS1 |  |  |  | LPA |  |  |  |  |  |  |  |  |  |  |
| GAA |  | DCWHK | CXCR6 |  |  |  | ANKRD1 |  |  |  |  |  |  |  |  |  |  |
| SMAD4 |  | CMH15 | CMTM7 |  |  |  | KCNE2 |  |  |  |  |  |  |  |  |  |  |
| INPP5E |  | BMFS6 | OTUD7A |  |  |  | IL1B |  |  |  |  |  |  |  |  |  |  |
| RET |  | CMD1AA | CELSR2 |  |  |  | BRCA2 |  |  |  |  |  |  |  |  |  |  |
| WT1 |  | SCFAI | SIK3 |  |  |  | TNFRSF1A |  |  |  |  |  |  |  |  |  |  |
| CHDS2 |  | NXD | COPD |  |  |  | MIR499A |  |  |  |  |  |  |  |  |  |  |
| CHDS3 |  | RSMD1 | GCKR |  |  |  | PSEN1 |  |  |  |  |  |  |  |  |  |  |
| SOS1 |  | SAV1 | GPR42 |  |  |  | ADM |  |  |  |  |  |  |  |  |  |  |
| FANCM |  | CMH1 | GRHL1 |  |  |  | EPO |  |  |  |  |  |  |  |  |  |  |
| THBD |  | GACI1 | ITPK1 |  |  |  | CAV3 |  |  |  |  |  |  |  |  |  |  |
| RERE |  | MELAS | POLK |  |  |  | ECE1 |  |  |  |  |  |  |  |  |  |  |
| MHRT |  | MMDD | PIK3CB |  |  |  | MYL4 |  |  |  |  |  |  |  |  |  |  |
| TLL1 |  | PPH5 | PIK3CD |  |  |  | LPL |  |  |  |  |  |  |  |  |  |  |
| POLG |  | CMD1R | PLCG1 |  |  |  | LAMP2 |  |  |  |  |  |  |  |  |  |  |
| CEP290 |  | CMD1M | SEMA5B |  |  |  | PON1 |  |  |  |  |  |  |  |  |  |  |
| BMP2 |  | CMD1DD | CHDH |  |  |  | MIR155 |  |  |  |  |  |  |  |  |  |  |
| FOXL2 |  | CMD2F | MAML3 |  |  |  | TGFB2 |  |  |  |  |  |  |  |  |  |  |
| TERC |  | CMD1E | ANKS1B |  |  |  | ADIPOQ |  |  |  |  |  |  |  |  |  |  |
| XRCC2 |  | PVOD1 | ACKR3 |  |  |  | HADHA |  |  |  |  |  |  |  |  |  |  |
| HRAS |  | CMD1Z | CPNE5 |  |  |  | SCARB2 |  |  |  |  |  |  |  |  |  |  |
| NOD2 |  | CMD1O | SUGP1 |  |  |  | PKD1 |  |  |  |  |  |  |  |  |  |  |
| POF1B |  | CMD1HH | REG1A |  |  |  | SOD1 |  |  |  |  |  |  |  |  |  |  |
| MKS1 |  | AOS1 | RFC1 |  |  |  | NAGLU |  |  |  |  |  |  |  |  |  |  |
| MT-ND1 |  | PFHB1B | SSTR4 |  |  |  | DNAH8 |  |  |  |  |  |  |  |  |  |  |
| MT-CYB |  | CHIME | BRS3 |  |  |  | CPT2 |  |  |  |  |  |  |  |  |  |  |
| TCAP |  | MCOPS2 | TM7SF2 |  |  |  | HFE |  |  |  |  |  |  |  |  |  |  |
| JUP |  | HLHS1 | VPS51 |  |  |  | HAMP |  |  |  |  |  |  |  |  |  |  |
| WDPCP |  | CHTD6 | BEST1 |  |  |  | MIR126 |  |  |  |  |  |  |  |  |  |  |
| BMP15 |  | HLHS2 | CA5A |  |  |  | KNG1 |  |  |  |  |  |  |  |  |  |  |
| SURF1 |  | CHDS6 | SLC30A3 |  |  |  | GPT |  |  |  |  |  |  |  |  |  |  |
| DTNA |  | CHDTHP | FSD1 |  |  |  | MMP2 |  |  |  |  |  |  |  |  |  |  |
| GYS1 |  | CTHM | CALCR |  |  |  | ANK2 |  |  |  |  |  |  |  |  |  |  |
| SMAD2 |  | CHDS7 | C1orf21 |  |  |  | CORIN |  |  |  |  |  |  |  |  |  |  |
| F3 |  | TACHD | FSD1L |  |  |  | MIR17 |  |  |  |  |  |  |  |  |  |  |
| CHDS1 |  | CHTD4 | ZPR1 |  |  |  | CCN2 |  |  |  |  |  |  |  |  |  |  |
| ENPP1 |  | CHOPS | LPAR2 |  |  |  | MB |  |  |  |  |  |  |  |  |  |  |
| STAG3 |  | HOS | CYTH3 |  |  |  | FGFR1 |  |  |  |  |  |  |  |  |  |  |
| DNAJC21 |  | SDDHD | DECR1 |  |  |  | LGALS3 |  |  |  |  |  |  |  |  |  |  |
| RINT1 |  | CHDFIDD | CAD |  |  |  | TLR4 |  |  |  |  |  |  |  |  |  |  |
| MIR320A |  | CHDED | GOLGA6A |  |  |  | EDNRA |  |  |  |  |  |  |  |  |  |  |
| HLA-B |  | CHTD5 | MFAP1 |  |  |  | SLC17A5 |  |  |  |  |  |  |  |  |  |  |
| AFF4 |  | DCHE | MUC2 |  |  |  | KCND3 |  |  |  |  |  |  |  |  |  |  |
| FGF8 |  | CHTD7 | RCBTB1 |  |  |  | ACE2 |  |  |  |  |  |  |  |  |  |  |
| TRDN |  | CHTD8 | MARCKSL1 |  |  |  | SLC8A1 |  |  |  |  |  |  |  |  |  |  |
| MT-TL1 |  | CHDS8 | AHSA1 |  |  |  | MMP3 |  |  |  |  |  |  |  |  |  |  |
| CRELD1 |  | CHDS9 | MRGPRX3 |  |  |  | ACVRL1 |  |  |  |  |  |  |  |  |  |  |
| B2M |  | LACHT | MRGPRX4 |  |  |  | ACTA2 |  |  |  |  |  |  |  |  |  |  |
| DIPK1A |  | CRIP1 | GPR151 |  |  |  | CASQ2 |  |  |  |  |  |  |  |  |  |  |
| PTH1R |  |  | CRK |  |  |  | NPPC |  |  |  |  |  |  |  |  |  |  |
| ISL1 |  |  | OXER1 |  |  |  | MECP2 |  |  |  |  |  |  |  |  |  |  |
| SDHA |  |  | GPRC6A |  |  |  | PRDM16 |  |  |  |  |  |  |  |  |  |  |
| MT-ATP6 |  |  | LPAR3 |  |  |  | ICAM1 |  |  |  |  |  |  |  |  |  |  |
| TSFM |  |  | RNF19A |  |  |  | ABCA1 |  |  |  |  |  |  |  |  |  |  |
| KCNA5 |  |  | MRGPRX1 |  |  |  | EMD |  |  |  |  |  |  |  |  |  |  |
| LARS1 |  |  | POLDIP2 |  |  |  | CCL2 |  |  |  |  |  |  |  |  |  |  |
| CHAT |  |  | VN1R17P |  |  |  | MIR145 |  |  |  |  |  |  |  |  |  |  |
| TGFBR2 |  |  | GPR166P |  |  |  | CST3 |  |  |  |  |  |  |  |  |  |  |
| TGFBR1 |  |  | LGR6 |  |  |  | ESR1 |  |  |  |  |  |  |  |  |  |  |
| ACTA1 |  |  | AIMP2 |  |  |  | VCAM1 |  |  |  |  |  |  |  |  |  |  |
| FLT4 |  |  | GRAP2 |  |  |  | PCSK9 |  |  |  |  |  |  |  |  |  |  |
| MT-CO3 |  |  | PGR-AS1 |  |  |  | MMP9 |  |  |  |  |  |  |  |  |  |  |
| TSC1 |  |  | GABPA |  |  |  | ALDH2 |  |  |  |  |  |  |  |  |  |  |
| HADHB |  |  | UTRN |  |  |  | LEP |  |  |  |  |  |  |  |  |  |  |
| PHOX2B |  |  | SLC33A1 |  |  |  | CX3CR1 |  |  |  |  |  |  |  |  |  |  |
| MYH11 |  |  | MTCO2P12 |  |  |  | FXN |  |  |  |  |  |  |  |  |  |  |
| CLIC2 |  |  | PPARGC1B |  |  |  | FGF23 |  |  |  |  |  |  |  |  |  |  |
| MED13L |  |  | CRMP1 |  |  |  | MEF2A |  |  |  |  |  |  |  |  |  |  |
| WDR19 |  |  | CSH1 |  |  |  | FOXC1 |  |  |  |  |  |  |  |  |  |  |
| SERPINC1 |  |  | CSH2 |  |  |  | ITGB3 |  |  |  |  |  |  |  |  |  |  |
| NOBOX |  |  | DAPK2 |  |  |  | JAK2 |  |  |  |  |  |  |  |  |  |  |
| TSC2 |  |  | HSPA9 |  |  |  | MEF2C |  |  |  |  |  |  |  |  |  |  |
| NSD1 |  |  | COX2 |  |  |  | MIR208A |  |  |  |  |  |  |  |  |  |  |
| F5 |  |  | NM |  |  |  | PIK3CA |  |  |  |  |  |  |  |  |  |  |
| PPA2 |  |  | CCHCR1 |  |  |  | NOS2 |  |  |  |  |  |  |  |  |  |  |
| SARS2 |  |  | DENR |  |  |  | CFH |  |  |  |  |  |  |  |  |  |  |
| ERCC1 |  |  | SELENBP1 |  |  |  | PRKD1 |  |  |  |  |  |  |  |  |  |  |
| TKT |  |  | MIR665 |  |  |  | PDE5A |  |  |  |  |  |  |  |  |  |  |
| CFAP47 |  |  | FSTL1 |  |  |  | HMOX1 |  |  |  |  |  |  |  |  |  |  |
| SDHB |  |  | PRRT2 |  |  |  | CYP11B2 |  |  |  |  |  |  |  |  |  |  |
| COL4A4 |  |  | MMRN1 |  |  |  | COL3A1 |  |  |  |  |  |  |  |  |  |  |
| MT-CO1 |  |  | INSRR |  |  |  | CXCL8 |  |  |  |  |  |  |  |  |  |  |
| PIK3C2A |  |  | PRKAA1 |  |  |  | SMAD3 |  |  |  |  |  |  |  |  |  |  |
| CFC1 |  |  | CENPJ |  |  |  | CHEK2 |  |  |  |  |  |  |  |  |  |  |
| SHH |  |  | SDC4 |  |  |  | HP |  |  |  |  |  |  |  |  |  |  |
| KDR |  |  | TAZ |  |  |  | IL18 |  |  |  |  |  |  |  |  |  |  |
| GJD2-DT |  |  | ARHGEF5 |  |  |  | RBP4 |  |  |  |  |  |  |  |  |  |  |
| MSH5 |  |  | PPP1R2C |  |  |  | INSR |  |  |  |  |  |  |  |  |  |  |
| PMM2 |  |  | TIMELESS |  |  |  | SLC22A5 |  |  |  |  |  |  |  |  |  |  |
| BCS1L |  |  | TLX1NB |  |  |  | SELP |  |  |  |  |  |  |  |  |  |  |
| CFTR |  |  | ABCB6 |  |  |  | PTEN |  |  |  |  |  |  |  |  |  |  |
| DYNC2H1 |  |  | FSTL3 |  |  |  | AGTR2 |  |  |  |  |  |  |  |  |  |  |
| EVC2 |  |  | FST |  |  |  | PLAT |  |  |  |  |  |  |  |  |  |  |
| TGFB3 |  |  | MORF4 |  |  |  | SERPINA1 |  |  |  |  |  |  |  |  |  |  |
| MEFV |  |  | IL34 |  |  |  | TNNC1 |  |  |  |  |  |  |  |  |  |  |
| CRYAB |  |  | SMYD1 |  |  |  | MTTP |  |  |  |  |  |  |  |  |  |  |
| CDK13 |  |  | GRK3 |  |  |  | LCN2 |  |  |  |  |  |  |  |  |  |  |
| FSHR |  |  | DBP |  |  |  | SMARCA4 |  |  |  |  |  |  |  |  |  |  |
| POLR1C |  |  | AAVS1 |  |  |  | IL4 |  |  |  |  |  |  |  |  |  |  |
| CDH2 |  |  | PTK2B |  |  |  | IL1RN |  |  |  |  |  |  |  |  |  |  |
| PTCH1 |  |  | IL37 |  |  |  | AKT1 |  |  |  |  |  |  |  |  |  |  |
| TAMM41 |  |  | CHAMP1 |  |  |  | CALCA |  |  |  |  |  |  |  |  |  |  |
| IFT172 |  |  | HCRTR2 |  |  |  | KCNK3 |  |  |  |  |  |  |  |  |  |  |
| CLCN5 |  |  | NR4A1 |  |  |  | MIR92B |  |  |  |  |  |  |  |  |  |  |
| SELENON |  |  | TLX2 |  |  |  | SLC6A2 |  |  |  |  |  |  |  |  |  |  |
| TINF2 |  |  | RND3 |  |  |  | PPARGC1A |  |  |  |  |  |  |  |  |  |  |
| DZIP1 |  |  | MIR199A2 |  |  |  | FN1 |  |  |  |  |  |  |  |  |  |  |
| FOXF1 |  |  | MIR19A |  |  |  | NEXN |  |  |  |  |  |  |  |  |  |  |
| IL2 |  |  | OSM |  |  |  | BMPR1A |  |  |  |  |  |  |  |  |  |  |
| COL1A1 |  |  | P2RX1 |  |  |  | CETP |  |  |  |  |  |  |  |  |  |  |
| AARS2 |  |  | P2RX7 |  |  |  | HSPG2 |  |  |  |  |  |  |  |  |  |  |
| HJV |  |  | P2RY2 |  |  |  | SPP1 |  |  |  |  |  |  |  |  |  |  |
| SLC25A4 |  |  | TNFRSF12A |  |  |  | ELANE |  |  |  |  |  |  |  |  |  |  |
| C3 |  |  | SCARA3 |  |  |  | CP |  |  |  |  |  |  |  |  |  |  |
| MT-ND4 |  |  | PENK |  |  |  | LCAT |  |  |  |  |  |  |  |  |  |  |
| MSH4 |  |  | PIN1 |  |  |  | POMC |  |  |  |  |  |  |  |  |  |  |
| PRKAR1A |  |  | KRT20 |  |  |  | MYOCD |  |  |  |  |  |  |  |  |  |  |
| LZTR1 |  |  | RBFOX1 |  |  |  | MMP1 |  |  |  |  |  |  |  |  |  |  |
| SOX9 |  |  | PPIA |  |  |  | BDNF |  |  |  |  |  |  |  |  |  |  |
| CREBBP |  |  | PTPA |  |  |  | FLT1 |  |  |  |  |  |  |  |  |  |  |
| SPATA16 |  |  | PRKAB1 |  |  |  | IGF2 |  |  |  |  |  |  |  |  |  |  |
| HBB |  |  | MOK |  |  |  | SERPINA3 |  |  |  |  |  |  |  |  |  |  |
| PLOD1 |  |  | ROS1 |  |  |  | MIR328 |  |  |  |  |  |  |  |  |  |  |
| LAMA2 |  |  | GINGF2 |  |  |  | FOS |  |  |  |  |  |  |  |  |  |  |
| MYSM1 |  |  | PINK1 |  |  |  | CTF1 |  |  |  |  |  |  |  |  |  |  |
| MT-ND5 |  |  | THBS2 |  |  |  | BMP4 |  |  |  |  |  |  |  |  |  |  |
| IFT74 |  |  | DNER |  |  |  | AVPR2 |  |  |  |  |  |  |  |  |  |  |
| CTLA4 |  |  | C20orf181 |  |  |  | CASR |  |  |  |  |  |  |  |  |  |  |
| TMEM67 |  |  | HDAC5 |  |  |  | GDF15 |  |  |  |  |  |  |  |  |  |  |
| PLXND1 |  |  | P2RX5-TAX1BP3 |  |  |  | ATM |  |  |  |  |  |  |  |  |  |  |
| SCN4B |  |  | GDF11 |  |  |  | PON2 |  |  |  |  |  |  |  |  |  |  |
| MYOT |  |  | NOD1 |  |  |  | TLR2 |  |  |  |  |  |  |  |  |  |  |
| SHOC2 |  |  | RACK1 |  |  |  | MED12 |  |  |  |  |  |  |  |  |  |  |
| NIPBL |  |  | SPON1 |  |  |  | PPP1CB |  |  |  |  |  |  |  |  |  |  |
| KCNJ8 |  |  | ATG7 |  |  |  | EDNRB |  |  |  |  |  |  |  |  |  |  |
| ACAD9 |  |  | SLC35A1 |  |  |  | GHRL |  |  |  |  |  |  |  |  |  |  |
| MT-ND6 |  |  | SLCO1B1 |  |  |  | MIR146A |  |  |  |  |  |  |  |  |  |  |
| ABCA3 |  |  | CST12P |  |  |  | LTBP2 |  |  |  |  |  |  |  |  |  |  |
| HFM1 |  |  | RIPK3 |  |  |  | IL17A |  |  |  |  |  |  |  |  |  |  |
| CTNNA3 |  |  | UCN3 |  |  |  | IL1A |  |  |  |  |  |  |  |  |  |  |
| SCN3B |  |  | CNP |  |  |  | NPY |  |  |  |  |  |  |  |  |  |  |
| HMGCR |  |  | CPB1 |  |  |  | EHMT1 |  |  |  |  |  |  |  |  |  |  |
| GDNF |  |  | CREM |  |  |  | BRCA1 |  |  |  |  |  |  |  |  |  |  |
| IGHMBP2 |  |  | CRX |  |  |  | AVP |  |  |  |  |  |  |  |  |  |  |
| FOXH1 |  |  | CRYGC |  |  |  | SFTPB |  |  |  |  |  |  |  |  |  |  |
| SPEF2 |  |  | CTNNA1 |  |  |  | EGFR |  |  |  |  |  |  |  |  |  |  |
| F7 |  |  | TMTC3 |  |  |  | FAS |  |  |  |  |  |  |  |  |  |  |
| EVC |  |  | S1PR1 |  |  |  | MGP |  |  |  |  |  |  |  |  |  |  |
| GATA1 |  |  | ELAVL2 |  |  |  | MYLK |  |  |  |  |  |  |  |  |  |  |
| MT-CO2 |  |  | CRYGEP |  |  |  | LIPC |  |  |  |  |  |  |  |  |  |  |
| MCM8 |  |  | ENPEP |  |  |  | SLC4A1 |  |  |  |  |  |  |  |  |  |  |
| HEY2 |  |  | FBL |  |  |  | CASZ1 |  |  |  |  |  |  |  |  |  |  |
| RASA1 |  |  | F2RL1 |  |  |  | HIF1A |  |  |  |  |  |  |  |  |  |  |
| EP300 |  |  | FGFR4 |  |  |  | TET2 |  |  |  |  |  |  |  |  |  |  |
| KMT2D |  |  | VEGFD |  |  |  | PRL |  |  |  |  |  |  |  |  |  |  |
| CACNB2 |  |  | P2RX2 |  |  |  | SOX4 |  |  |  |  |  |  |  |  |  |  |
| GDF9 |  |  | MPRIP |  |  |  | APLN |  |  |  |  |  |  |  |  |  |  |
| FANCA |  |  | MLC1 |  |  |  | CHGA |  |  |  |  |  |  |  |  |  |  |
| FANCC |  |  | ANGPTL2 |  |  |  | TP63 |  |  |  |  |  |  |  |  |  |  |
| GBA1 |  |  | ABCA4 |  |  |  | CTNNB1 |  |  |  |  |  |  |  |  |  |  |
| PEX6 |  |  | GAS6 |  |  |  | BCL2 |  |  |  |  |  |  |  |  |  |  |
| ABCC6 |  |  | GHSR |  |  |  | MAPK14 |  |  |  |  |  |  |  |  |  |  |
| FLNC-AS1 |  |  | PYCARD |  |  |  | G6PD |  |  |  |  |  |  |  |  |  |  |
| CEP19 |  |  | IGFBP4 |  |  |  | RBM10 |  |  |  |  |  |  |  |  |  |  |
| MIR140 |  |  | IGHG3 |  |  |  | FKRP |  |  |  |  |  |  |  |  |  |  |
| CDKN1C |  |  | IRF1 |  |  |  | FGF2 |  |  |  |  |  |  |  |  |  |  |
| GLB1 |  |  | LUM |  |  |  | ANGPT2 |  |  |  |  |  |  |  |  |  |  |
| COQ2 |  |  | MIR130B |  |  |  | ZCCHC8 |  |  |  |  |  |  |  |  |  |  |
| SOHLH1 |  |  | MIR18A |  |  |  | CS |  |  |  |  |  |  |  |  |  |  |
| ARID1B |  |  | MIR212 |  |  |  | CSF3 |  |  |  |  |  |  |  |  |  |  |
| KAT6B |  |  | MIR216A |  |  |  | ASXL1 |  |  |  |  |  |  |  |  |  |  |
| SCN2B |  |  | MIR27B |  |  |  | HSPB7 |  |  |  |  |  |  |  |  |  |  |
| DSC2 |  |  | MIR29B2 |  |  |  | GUSB |  |  |  |  |  |  |  |  |  |  |
| MCM9 |  |  | ARRB2 |  |  |  | CALR |  |  |  |  |  |  |  |  |  |  |
| SBDS |  |  | STS |  |  |  | BAZ1B |  |  |  |  |  |  |  |  |  |  |
| ADAMTS13 |  |  | MIR340 |  |  |  | HLA-DQA1 |  |  |  |  |  |  |  |  |  |  |
| H2AC18 |  |  | MIR342 |  |  |  | LOX |  |  |  |  |  |  |  |  |  |  |
| C4A |  |  | COX1 |  |  |  | OLR1 |  |  |  |  |  |  |  |  |  |  |
| TF |  |  | CNOT3 |  |  |  | TIMP1 |  |  |  |  |  |  |  |  |  |  |
| SRP72 |  |  | CCN3 |  |  |  | NOS1 |  |  |  |  |  |  |  |  |  |  |
| RIT1 |  |  | ACR |  |  |  | TNNI3K |  |  |  |  |  |  |  |  |  |  |
| MT-ND3 |  |  | NT5E |  |  |  | VDR |  |  |  |  |  |  |  |  |  |  |
| FOXP3 |  |  | ATP2B4 |  |  |  | GH1 |  |  |  |  |  |  |  |  |  |  |
| EDN3 |  |  | TRIM72 |  |  |  | MIR423 |  |  |  |  |  |  |  |  |  |  |
| HLA-DQB1 |  |  | MIR425 |  |  |  | HMGB1 |  |  |  |  |  |  |  |  |  |  |
| LRRC56 |  |  | P2RX3 |  |  |  | RRAD |  |  |  |  |  |  |  |  |  |  |
| TBX3 |  |  | P2RX5 |  |  |  | FOXP1 |  |  |  |  |  |  |  |  |  |  |
| TMEM260 |  |  | P2RY1 |  |  |  | TNFRSF11B |  |  |  |  |  |  |  |  |  |  |
| TRIP4 |  |  | PCSK6 |  |  |  | MTOR |  |  |  |  |  |  |  |  |  |  |
| FGFR3 |  |  | DUOX2 |  |  |  | TFR2 |  |  |  |  |  |  |  |  |  |  |
| SDHD |  |  | SERPINA5 |  |  |  | SELE |  |  |  |  |  |  |  |  |  |  |
| EPHB4 |  |  | GP6 |  |  |  | MIR199A1 |  |  |  |  |  |  |  |  |  |  |
| NOTCH2 |  |  | UBR5 |  |  |  | NOS1AP |  |  |  |  |  |  |  |  |  |  |
| SCO2 |  |  | ISYNA1 |  |  |  | RETN |  |  |  |  |  |  |  |  |  |  |
| TBX2 |  |  | PDK1 |  |  |  | SOD3 |  |  |  |  |  |  |  |  |  |  |
| SALL1 |  |  | LUC7L3 |  |  |  | NFKB1 |  |  |  |  |  |  |  |  |  |  |
| DCHS1 |  |  | PIM1 |  |  |  | KCNIP2 |  |  |  |  |  |  |  |  |  |  |
| NDUFS4 |  |  | DUOX1 |  |  |  | AGER |  |  |  |  |  |  |  |  |  |  |
| MYLK2 |  |  | RNF111 |  |  |  | DYSF |  |  |  |  |  |  |  |  |  |  |
| PECAM1 |  |  | MFN1 |  |  |  | CYP3A5 |  |  |  |  |  |  |  |  |  |  |
| ERCC6L2 |  |  | ERBIN |  |  |  | HGF |  |  |  |  |  |  |  |  |  |  |
| CEP85L |  |  | BIRC6 |  |  |  | SLC9A1 |  |  |  |  |  |  |  |  |  |  |
| CCR6 |  |  | CREBZF |  |  |  | NCF1 |  |  |  |  |  |  |  |  |  |  |
| TEX15 |  |  | ROCK1 |  |  |  | CD46 |  |  |  |  |  |  |  |  |  |  |
| MYRF |  |  | BDH1 |  |  |  | GATM |  |  |  |  |  |  |  |  |  |  |
| PAH |  |  | CCL19 |  |  |  | ERBB4 |  |  |  |  |  |  |  |  |  |  |
| DNAJC19 |  |  | CCL21 |  |  |  | ADCY10 |  |  |  |  |  |  |  |  |  |  |
| NF1 |  |  | TMBIM1 |  |  |  | MIF |  |  |  |  |  |  |  |  |  |  |
| SCN10A |  |  | BMI1 |  |  |  | PTH |  |  |  |  |  |  |  |  |  |  |
| MT-ND2 |  |  | BNIP3L |  |  |  | RHOA |  |  |  |  |  |  |  |  |  |  |
| GALT |  |  | STIM1 |  |  |  | RAC1 |  |  |  |  |  |  |  |  |  |  |
| SLC2A10 |  |  | ZEB1 |  |  |  | NFATC4 |  |  |  |  |  |  |  |  |  |  |
| PLCZ1 |  |  | CAPN1 |  |  |  | CDKN2B-AS1 |  |  |  |  |  |  |  |  |  |  |
| NPHP1 |  |  | HOPX |  |  |  | AQP2 |  |  |  |  |  |  |  |  |  |  |
| MBL2 |  |  | TNFRSF10B |  |  |  | ADAM17 |  |  |  |  |  |  |  |  |  |  |
| NRAS |  |  | CCN5 |  |  |  | HADH |  |  |  |  |  |  |  |  |  |  |
| STAT1 |  |  | P2RX6 |  |  |  | GNPTAB |  |  |  |  |  |  |  |  |  |  |
| LARS2 |  |  | GAL3ST1 |  |  |  | KL |  |  |  |  |  |  |  |  |  |  |
| NBN |  |  | RNA18SN5 |  |  |  | ATP5F1A |  |  |  |  |  |  |  |  |  |  |
| FHL1 |  |  | NR2E3 |  |  |  | FKBP1B |  |  |  |  |  |  |  |  |  |  |
| ASPH |  |  | MIR675 |  |  |  | UMOD |  |  |  |  |  |  |  |  |  |  |
| IFT140 |  |  | MIR147B |  |  |  | ACTB |  |  |  |  |  |  |  |  |  |  |
| FRAXA |  |  | MIR744 |  |  |  | PNPLA2 |  |  |  |  |  |  |  |  |  |  |
| CFAP43 |  |  | HDAC6 |  |  |  | DNMT3A |  |  |  |  |  |  |  |  |  |  |
| DNAH10 |  |  | TRAP |  |  |  | GPD1L |  |  |  |  |  |  |  |  |  |  |
| FCGR2A |  |  | HLP |  |  |  | BGLAP |  |  |  |  |  |  |  |  |  |  |
| ZMPSTE24 |  |  | MIR1306 |  |  |  | NSD2 |  |  |  |  |  |  |  |  |  |  |
| APOC3 |  |  | NPPA-AS1 |  |  |  | CD40 |  |  |  |  |  |  |  |  |  |  |
| DIAPH2 |  |  | TMX2-CTNND1 |  |  |  | F9 |  |  |  |  |  |  |  |  |  |  |
| FOXC2 |  |  | PPR1 |  |  |  | ERBB2 |  |  |  |  |  |  |  |  |  |  |
| ERCC4 |  |  | KLRC4-KLRK1 |  |  |  | ARSB |  |  |  |  |  |  |  |  |  |  |
| PRKG1 |  |  | FAME3 |  |  |  | NPR2 |  |  |  |  |  |  |  |  |  |  |
| SEMA3E |  |  | MIR4491 |  |  |  | NFE2L2 |  |  |  |  |  |  |  |  |  |  |
| COL1A2 |  |  | NR1H3 |  |  |  | MDM2 |  |  |  |  |  |  |  |  |  |  |
| ITGAM |  |  | COX17 |  |  |  | PDGFRA |  |  |  |  |  |  |  |  |  |  |
| MMACHC |  |  | DPP3 |  |  |  | HSPD1 |  |  |  |  |  |  |  |  |  |  |
| RAI1 |  |  | LINC-ROR |  |  |  | ADD1 |  |  |  |  |  |  |  |  |  |  |
| CYP11A1 |  |  | NPY4R2 |  |  |  | MIR34A |  |  |  |  |  |  |  |  |  |  |
| DNAH5 |  |  | PPIF |  |  |  | TIMP4 |  |  |  |  |  |  |  |  |  |  |
| SOX17 |  |  | OPN1MW3 |  |  |  | ARID1A |  |  |  |  |  |  |  |  |  |  |
| CYCS |  |  | HIPK3 |  |  |  | VCP |  |  |  |  |  |  |  |  |  |  |
| SMPD1 |  |  | FRY |  |  |  | MIR142 |  |  |  |  |  |  |  |  |  |  |
| COX5A |  |  | CDH15 |  |  |  | AMPD1 |  |  |  |  |  |  |  |  |  |  |
| AKAP9 |  |  | RASA4 |  |  |  | UBE2T |  |  |  |  |  |  |  |  |  |  |
| TWNK |  |  | ALYREF |  |  |  | PF4 |  |  |  |  |  |  |  |  |  |  |
| MPV17 |  |  | AK6 |  |  |  | PDE9A |  |  |  |  |  |  |  |  |  |  |
| FANCI |  |  | CALCOCO2 |  |  |  | TCF7L2 |  |  |  |  |  |  |  |  |  |  |
| SGCB |  |  | STUB1 |  |  |  | GATA3 |  |  |  |  |  |  |  |  |  |  |
| PACS1 |  |  | MARCHF6 |  |  |  | TFRC |  |  |  |  |  |  |  |  |  |  |
| KIT |  |  | CNPY2 |  |  |  | POSTN |  |  |  |  |  |  |  |  |  |  |
| GLI3 |  |  | KLF2 |  |  |  | SYCP3 |  |  |  |  |  |  |  |  |  |  |
| CC2D2A |  |  | CDS1 |  |  |  | GSN |  |  |  |  |  |  |  |  |  |  |
| HYLS1 |  |  | PRMT5 |  |  |  | GAPDH |  |  |  |  |  |  |  |  |  |  |
| BCOR |  |  | LINC02210-CRHR1 |  |  |  | CYP2C19 |  |  |  |  |  |  |  |  |  |  |
| SLC34A1 |  |  | CIB1 |  |  |  | TPI1 |  |  |  |  |  |  |  |  |  |  |
| NPHP3 |  |  | PERCC1 |  |  |  | BMP10 |  |  |  |  |  |  |  |  |  |  |
| MYH7B |  |  | CXCL13 |  |  |  | CSF2 |  |  |  |  |  |  |  |  |  |  |
| GBE1 |  |  | SLU7 |  |  |  | GSR |  |  |  |  |  |  |  |  |  |  |
| GLI2 |  |  | GNLY |  |  |  | MIR195 |  |  |  |  |  |  |  |  |  |  |
| NDUFV1 |  |  | SCGN |  |  |  | TIMP2 |  |  |  |  |  |  |  |  |  |  |
| MYOZ2 |  |  | TCFL5 |  |  |  | ACADS |  |  |  |  |  |  |  |  |  |  |
| COX10 |  |  | CGB3 |  |  |  | VIP |  |  |  |  |  |  |  |  |  |  |
| FIGLA |  |  | PDE10A |  |  |  | NLRP3 |  |  |  |  |  |  |  |  |  |  |
| LRP2 |  |  | LILRB1 |  |  |  | MIR223 |  |  |  |  |  |  |  |  |  |  |
| SCO1 |  |  | SUGT1 |  |  |  | HLA-C |  |  |  |  |  |  |  |  |  |  |
| SMG9 |  |  | FASTK |  |  |  | CCL5 |  |  |  |  |  |  |  |  |  |  |
| LTA |  |  | PRDX3 |  |  |  | LEPR |  |  |  |  |  |  |  |  |  |  |
| ALMS1 |  |  | CKAP4 |  |  |  | APRT |  |  |  |  |  |  |  |  |  |  |
| VPS33A |  |  | PDXDC2P |  |  |  | LTBP3 |  |  |  |  |  |  |  |  |  |  |
| HNF1B |  |  | FERMT2 |  |  |  | MIR182 |  |  |  |  |  |  |  |  |  |  |
| DACT1 |  |  | LILRB4 |  |  |  | MYC |  |  |  |  |  |  |  |  |  |  |
| FIG4 |  |  | LIAS |  |  |  | COL11A2 |  |  |  |  |  |  |  |  |  |  |
| MIR196A2 |  |  | CHGB |  |  |  | KMT2A |  |  |  |  |  |  |  |  |  |  |
| TNFSF11 |  |  | WDHD1 |  |  |  | HTR2A |  |  |  |  |  |  |  |  |  |  |
| RPL36A-HNRNPH2 |  |  | MAP4K5 |  |  |  | DLD |  |  |  |  |  |  |  |  |  |  |
| RPGRIP1L |  |  | USP18 |  |  |  | CCR5 |  |  |  |  |  |  |  |  |  |  |
| WDR35 |  |  | LYST |  |  |  | MIR22 |  |  |  |  |  |  |  |  |  |  |
| SEPTIN12 |  |  | ECD |  |  |  | PTGS1 |  |  |  |  |  |  |  |  |  |  |
| EPAS1 |  |  | ACOT7 |  |  |  | GDF2 |  |  |  |  |  |  |  |  |  |  |
| ADA |  |  | AZIN2 |  |  |  | CXCL10 |  |  |  |  |  |  |  |  |  |  |
| FANCD2 |  |  | TXNRD3 |  |  |  | NNT |  |  |  |  |  |  |  |  |  |  |
| FGA |  |  | FHAD1 |  |  |  | ERCC8 |  |  |  |  |  |  |  |  |  |  |
| SFTPC |  |  | C1QTNF1 |  |  |  | GCG |  |  |  |  |  |  |  |  |  |  |
| INVS |  |  | CIRBP |  |  |  | TFPI |  |  |  |  |  |  |  |  |  |  |
| HLA-A |  |  | CISH |  |  |  | FASLG |  |  |  |  |  |  |  |  |  |  |
| BRDT |  |  | ADCYAP1 |  |  |  | SETD2 |  |  |  |  |  |  |  |  |  |  |
| AGXT |  |  | ADCYAP1R1 |  |  |  | RAPGEF3 |  |  |  |  |  |  |  |  |  |  |
| SLC40A1 |  |  | JAML |  |  |  | TRIM32 |  |  |  |  |  |  |  |  |  |  |
| RAD51C |  |  | JDP2 |  |  |  | IKBKG |  |  |  |  |  |  |  |  |  |  |
| CD40LG |  |  | CCR4 |  |  |  | MOV10L1 |  |  |  |  |  |  |  |  |  |  |
| FBN2 |  |  | CCR7 |  |  |  | TWIST1 |  |  |  |  |  |  |  |  |  |  |
| NEU1 |  |  | CNN1 |  |  |  | TFAM |  |  |  |  |  |  |  |  |  |  |
| SMC1A |  |  | CNR2 |  |  |  | ETS1 |  |  |  |  |  |  |  |  |  |  |
| RRAS2 |  |  | MYOM3 |  |  |  | MSTN |  |  |  |  |  |  |  |  |  |  |
| NPC1 |  |  | CMPK2 |  |  |  | ACADM |  |  |  |  |  |  |  |  |  |  |
| MLXIPL |  |  | RBM45 |  |  |  | MYL7 |  |  |  |  |  |  |  |  |  |  |
| MEIOB |  |  | KLF6 |  |  |  | MIR143 |  |  |  |  |  |  |  |  |  |  |
| MEN1 |  |  | EMB |  |  |  | SEMA4D |  |  |  |  |  |  |  |  |  |  |
| HBA1 |  |  | SLCO6A1 |  |  |  | TH |  |  |  |  |  |  |  |  |  |  |
| GATAD1 |  |  | KLF14 |  |  |  | PTGS2 |  |  |  |  |  |  |  |  |  |  |
| MRAS |  |  | CRABP1 |  |  |  | NAMPT |  |  |  |  |  |  |  |  |  |  |
| FKBP6 |  |  | FUNDC1 |  |  |  | ACTG2 |  |  |  |  |  |  |  |  |  |  |
| IDS |  |  | CRHR1 |  |  |  | HEAT2 |  |  |  |  |  |  |  |  |  |  |
| SUN5 |  |  | ASB14 |  |  |  | CXCR4 |  |  |  |  |  |  |  |  |  |  |
| PDX1 |  |  | CSE1L |  |  |  | WNT5A |  |  |  |  |  |  |  |  |  |  |
| SYCE1 |  |  | LGALS16 |  |  |  | DNAAF1 |  |  |  |  |  |  |  |  |  |  |
| CBS |  |  | CTNND1 |  |  |  | ENO2 |  |  |  |  |  |  |  |  |  |  |
| NUP155 |  |  | CTRL |  |  |  | SHBG |  |  |  |  |  |  |  |  |  |  |
| CFAP44 |  |  | GPBAR1 |  |  |  | HDAC4 |  |  |  |  |  |  |  |  |  |  |
| SETBP1 |  |  | RMDN2 |  |  |  | PGF |  |  |  |  |  |  |  |  |  |  |
| NEB |  |  | CTSS |  |  |  | LMOD2 |  |  |  |  |  |  |  |  |  |  |
| CFAP70 |  |  | PPM1K |  |  |  | SLC6A4 |  |  |  |  |  |  |  |  |  |  |
| MIR204 |  |  | CYP1A1 |  |  |  | ITGA2B |  |  |  |  |  |  |  |  |  |  |
| COMT |  |  | AMZ1 |  |  |  | NR1H4 |  |  |  |  |  |  |  |  |  |  |
| LTBP4 |  |  | DAO |  |  |  | IL33 |  |  |  |  |  |  |  |  |  |  |
| PTPN22 |  |  | WBP2NL |  |  |  | FABP4 |  |  |  |  |  |  |  |  |  |  |
| SOS2 |  |  | GADD45A |  |  |  | BSG |  |  |  |  |  |  |  |  |  |  |
| KIF7 |  |  | DDT |  |  |  | MFN2 |  |  |  |  |  |  |  |  |  |  |
| TTC21B |  |  | TIMM8A |  |  |  | PPIG |  |  |  |  |  |  |  |  |  |  |
| C14orf39 |  |  | SLC30A8 |  |  |  | ANGPT1 |  |  |  |  |  |  |  |  |  |  |
| NDUFAF2 |  |  | HFM |  |  |  | CPT1A |  |  |  |  |  |  |  |  |  |  |
| ACTL9 |  |  | ADAMTS16 |  |  |  | BVES |  |  |  |  |  |  |  |  |  |  |
| FLNB |  |  | PPP1R18 |  |  |  | MIR208B |  |  |  |  |  |  |  |  |  |  |
| PLG |  |  | NLRP6 |  |  |  | IGFBP7 |  |  |  |  |  |  |  |  |  |  |
| LIPA |  |  | DUSP2 |  |  |  | BGN |  |  |  |  |  |  |  |  |  |  |
| FGF10 |  |  | E2F6 |  |  |  | HTR2B |  |  |  |  |  |  |  |  |  |  |
| SCNN1A |  |  | EGR1 |  |  |  | ACHE |  |  |  |  |  |  |  |  |  |  |
| PEX5 |  |  | EIF4EBP1 |  |  |  | MTO1 |  |  |  |  |  |  |  |  |  |  |
| TRAF7 |  |  | ELAVL1 |  |  |  | MIR214 |  |  |  |  |  |  |  |  |  |  |
| SEMA3A |  |  | DHRS7C |  |  |  | PRTN3 |  |  |  |  |  |  |  |  |  |  |
| BBS10 |  |  | CTTN |  |  |  | NUBPL |  |  |  |  |  |  |  |  |  |  |
| ATP13A3 |  |  | PRSS55 |  |  |  | SLC2A1 |  |  |  |  |  |  |  |  |  |  |
| DPY19L2 |  |  | EPHA3 |  |  |  | FBLN5 |  |  |  |  |  |  |  |  |  |  |
| POLG2 |  |  | ETV3 |  |  |  | LRPPRC |  |  |  |  |  |  |  |  |  |  |
| ASXL3 |  |  | ALCAM |  |  |  | MIR23A |  |  |  |  |  |  |  |  |  |  |
| FANCL |  |  | FBLN1 |  |  |  | SPTBN1 |  |  |  |  |  |  |  |  |  |  |
| IGFBP3 |  |  | TPCN2 |  |  |  | CELSR1 |  |  |  |  |  |  |  |  |  |  |
| TRIM37 |  |  | FDPS |  |  |  | MAPK8 |  |  |  |  |  |  |  |  |  |  |
| FANCG |  |  | FDXR |  |  |  | UCP2 |  |  |  |  |  |  |  |  |  |  |
| TK2 |  |  | FGF4 |  |  |  | CTSD |  |  |  |  |  |  |  |  |  |  |
| SGSH |  |  | FKBP1AP1 |  |  |  | MEG3 |  |  |  |  |  |  |  |  |  |  |
| MTR |  |  | FKBP1AP2 |  |  |  | NEDD4L |  |  |  |  |  |  |  |  |  |  |
| DNAH17 |  |  | FKBP1AP3 |  |  |  | NOTCH3 |  |  |  |  |  |  |  |  |  |  |
| FHOD3 |  |  | FKBP1AP4 |  |  |  | FTO |  |  |  |  |  |  |  |  |  |  |
| ZEB2 |  |  | KLRK1 |  |  |  | PAPPA |  |  |  |  |  |  |  |  |  |  |
| MT-TW |  |  | MON2 |  |  |  | ATP6AP2 |  |  |  |  |  |  |  |  |  |  |
| LZTFL1 |  |  | PDS5B |  |  |  | DMPK |  |  |  |  |  |  |  |  |  |  |
| GHR |  |  | FOXM1 |  |  |  | ZFHX3 |  |  |  |  |  |  |  |  |  |  |
| PKHD1 |  |  | ARC |  |  |  | IGFBP1 |  |  |  |  |  |  |  |  |  |  |
| MT-TK |  |  | PDS5A |  |  |  | BAX |  |  |  |  |  |  |  |  |  |  |
| MT-ATP8 |  |  | FMOD |  |  |  | PDE4A |  |  |  |  |  |  |  |  |  |  |
| SMAD9 |  |  | UFL1 |  |  |  | PICK1 |  |  |  |  |  |  |  |  |  |  |
| PRODH |  |  | TRS-AGA2-3 |  |  |  | GTPBP3 |  |  |  |  |  |  |  |  |  |  |
| OXTR |  |  | POFUT1 |  |  |  | F10 |  |  |  |  |  |  |  |  |  |  |
| OFD1 |  |  | SLC39A14 |  |  |  | AHSG |  |  |  |  |  |  |  |  |  |  |
| LOC110806306 |  |  | SMUG1 |  |  |  | MYOD1 |  |  |  |  |  |  |  |  |  |  |
| DNAH2 |  |  | PHLDA3 |  |  |  | PTHLH |  |  |  |  |  |  |  |  |  |  |
| RASA2 |  |  | BACE1 |  |  |  | INSL6 |  |  |  |  |  |  |  |  |  |  |
| IDUA |  |  | OSBP2 |  |  |  | CRH |  |  |  |  |  |  |  |  |  |  |
| TEX11 |  |  | IL17RA |  |  |  | SIRT1 |  |  |  |  |  |  |  |  |  |  |
| ACADVL |  |  | DIANPH |  |  |  | RPGR |  |  |  |  |  |  |  |  |  |  |
| GTF2IRD1 |  |  | BRD1 |  |  |  | SLC12A2 |  |  |  |  |  |  |  |  |  |  |
| BBS2 |  |  | RICTOR |  |  |  | MMP14 |  |  |  |  |  |  |  |  |  |  |
| NEK1 |  |  | GAB1 |  |  |  | PRKCD |  |  |  |  |  |  |  |  |  |  |
| KLHL10 |  |  | TXN2 |  |  |  | TAC1 |  |  |  |  |  |  |  |  |  |  |
| IFIH1 |  |  | PART1 |  |  |  | MIR144 |  |  |  |  |  |  |  |  |  |  |
| MAP3K7 |  |  | GALNT1 |  |  |  | SIRT6 |  |  |  |  |  |  |  |  |  |  |
| VHL |  |  | TOR1AIP1 |  |  |  | IL6R |  |  |  |  |  |  |  |  |  |  |
| TALDO1 |  |  | LRIT1 |  |  |  | MMUT |  |  |  |  |  |  |  |  |  |  |
| CFAP58 |  |  | OPTC |  |  |  | BRD4 |  |  |  |  |  |  |  |  |  |  |
| MT-TS1 |  |  | GCH1 |  |  |  | PARP1 |  |  |  |  |  |  |  |  |  |  |
| HSD11B2 |  |  | OPN1MW |  |  |  | THBS1 |  |  |  |  |  |  |  |  |  |  |
| BBS1 |  |  | OPLAH |  |  |  | DNASE1 |  |  |  |  |  |  |  |  |  |  |
| CYP2C9 |  |  | CBLIF |  |  |  | TNFRSF1B |  |  |  |  |  |  |  |  |  |  |
| ABCB11 |  |  | VPS4A |  |  |  | DNM1L |  |  |  |  |  |  |  |  |  |  |
| STX1A |  |  | OXGR1 |  |  |  | MIR24-1 |  |  |  |  |  |  |  |  |  |  |
| FGFR2 |  |  | EIF3K |  |  |  | MDH2 |  |  |  |  |  |  |  |  |  |  |
| MYH3 |  |  | MAT2B |  |  |  | CLCNKA |  |  |  |  |  |  |  |  |  |  |
| QRICH2 |  |  | TOR2A |  |  |  | TRPC6 |  |  |  |  |  |  |  |  |  |  |
| ARHGAP31 |  |  | GNA12 |  |  |  | CX3CL1 |  |  |  |  |  |  |  |  |  |  |
| PEX7 |  |  | ABO |  |  |  | ADRB3 |  |  |  |  |  |  |  |  |  |  |
| PRF1 |  |  | GOLGB1 |  |  |  | CYBB |  |  |  |  |  |  |  |  |  |  |
| NFATC1 |  |  | SLCO1B3 |  |  |  | PIK3CG |  |  |  |  |  |  |  |  |  |  |
| SLC25A13 |  |  | NEAT1 |  |  |  | PTX3 |  |  |  |  |  |  |  |  |  |  |
| NDUFS2 |  |  | GPR17 |  |  |  | DCN |  |  |  |  |  |  |  |  |  |  |
| F13A1 |  |  | SYPL2 |  |  |  | DNMT1 |  |  |  |  |  |  |  |  |  |  |
| NDUFB11 |  |  | BTBD8 |  |  |  | NR3C1 |  |  |  |  |  |  |  |  |  |  |
| NR0B1 |  |  | SLC9C1 |  |  |  | MAPK3 |  |  |  |  |  |  |  |  |  |  |
| SLC26A8 |  |  | HIPK2 |  |  |  | NOX4 |  |  |  |  |  |  |  |  |  |  |
| CACNA2D1 |  |  | ANPEP |  |  |  | TNFSF12 |  |  |  |  |  |  |  |  |  |  |
| IFT122 |  |  | LGALS13 |  |  |  | SPTAN1 |  |  |  |  |  |  |  |  |  |  |
| RPS19 |  |  | CXCL1 |  |  |  | RNLS |  |  |  |  |  |  |  |  |  |  |
| GCK |  |  | GSK3A |  |  |  | MC4R |  |  |  |  |  |  |  |  |  |  |
| ARMC2 |  |  | GUCA2B |  |  |  | ESR2 |  |  |  |  |  |  |  |  |  |  |
| KCNQ1OT1 |  |  | GNL2 |  |  |  | MIR150 |  |  |  |  |  |  |  |  |  |  |
| GNA11 |  |  | RMC1 |  |  |  | JARID2 |  |  |  |  |  |  |  |  |  |  |
| SON |  |  | MDFIC |  |  |  | HSPA4 |  |  |  |  |  |  |  |  |  |  |
| CFAP251 |  |  | H2AX |  |  |  | S100A1 |  |  |  |  |  |  |  |  |  |  |
| B3GALT6 |  |  | KCNIP3 |  |  |  | MIR20A |  |  |  |  |  |  |  |  |  |  |
| KDM6A |  |  | EHD3 |  |  |  | GPR35 |  |  |  |  |  |  |  |  |  |  |
| BBS9 |  |  | EHD2 |  |  |  | PAFAH1B1 |  |  |  |  |  |  |  |  |  |  |
| LRP6 |  |  | HK2 |  |  |  | SLC39A8 |  |  |  |  |  |  |  |  |  |  |
| COL4A3 |  |  | HMBS |  |  |  | CDKN1A |  |  |  |  |  |  |  |  |  |  |
| MCTP2 |  |  | HMGB2 |  |  |  | S100B |  |  |  |  |  |  |  |  |  |  |
| RNU4ATAC |  |  | HNRNPD |  |  |  | ACTG1 |  |  |  |  |  |  |  |  |  |  |
| FANCF |  |  | APOF |  |  |  | SLC4A3 |  |  |  |  |  |  |  |  |  |  |
| KMT2C |  |  | HRH2 |  |  |  | MAP3K20 |  |  |  |  |  |  |  |  |  |  |
| DLL1 |  |  | PRMT1 |  |  |  | PPBP |  |  |  |  |  |  |  |  |  |  |
| CALM1 |  |  | HSPA5 |  |  |  | HRC |  |  |  |  |  |  |  |  |  |  |
| ASAH1 |  |  | FFAR4 |  |  |  | SLC27A6 |  |  |  |  |  |  |  |  |  |  |
| RAD51 |  |  | C1QTNF9 |  |  |  | DNM2 |  |  |  |  |  |  |  |  |  |  |
| FAH |  |  | ID2 |  |  |  | GFAP |  |  |  |  |  |  |  |  |  |  |
| MEGF8 |  |  | NRBP2 |  |  |  | IL1RL1 |  |  |  |  |  |  |  |  |  |  |
| RRM2B |  |  | COL6A4P1 |  |  |  | ADAMTSL1 |  |  |  |  |  |  |  |  |  |  |
| TDRD9 |  |  | ACTBL2 |  |  |  | PDSS2 |  |  |  |  |  |  |  |  |  |  |
| IGF1R |  |  | IGFBP5 |  |  |  | SLC9A3 |  |  |  |  |  |  |  |  |  |  |
| TMPO |  |  | IL4R |  |  |  | ADRA2B |  |  |  |  |  |  |  |  |  |  |
| ROBO1 |  |  | IL13RA1 |  |  |  | MMP12 |  |  |  |  |  |  |  |  |  |  |
| DYNC2I1 |  |  | ILF3 |  |  |  | CD14 |  |  |  |  |  |  |  |  |  |  |
| MIR29A |  |  | ITGB2 |  |  |  | ANXA5 |  |  |  |  |  |  |  |  |  |  |
| IL1R1 |  |  | ITPR2 |  |  |  | CASP1 |  |  |  |  |  |  |  |  |  |  |
| FOXRED1 |  |  | GSTK1 |  |  |  | ALDH1A2 |  |  |  |  |  |  |  |  |  |  |
| TLR7 |  |  | KCP |  |  |  | CYP2J2 |  |  |  |  |  |  |  |  |  |  |
| DNAI1 |  |  | ENHO |  |  |  | MTPN |  |  |  |  |  |  |  |  |  |  |
| PALB2 |  |  | MALAT1 |  |  |  | DPP4 |  |  |  |  |  |  |  |  |  |  |
| HGSNAT |  |  | ARG1 |  |  |  | MPI |  |  |  |  |  |  |  |  |  |  |
| SMARCA2 |  |  | NPSR1 |  |  |  | AMACR |  |  |  |  |  |  |  |  |  |  |
| EPG5 |  |  | LAD1 |  |  |  | DAG1 |  |  |  |  |  |  |  |  |  |  |
| AGK |  |  | LGALS1 |  |  |  | PDE4D |  |  |  |  |  |  |  |  |  |  |
| CELA2A |  |  | LY75 |  |  |  | FURIN |  |  |  |  |  |  |  |  |  |  |
| LOC110806263 |  |  | MIR100 |  |  |  | YY1 |  |  |  |  |  |  |  |  |  |  |
| SMARCAL1 |  |  | MIR127 |  |  |  | PPP3CA |  |  |  |  |  |  |  |  |  |  |
| SPAG17 |  |  | MIR134 |  |  |  | HSPB1 |  |  |  |  |  |  |  |  |  |  |
| TBX18 |  |  | MIR137 |  |  |  | IGF2R |  |  |  |  |  |  |  |  |  |  |
| SETD5 |  |  | MIR148A |  |  |  | RAMP2 |  |  |  |  |  |  |  |  |  |  |
| FLCN |  |  | MIR183 |  |  |  | MST1 |  |  |  |  |  |  |  |  |  |  |
| IL2RA |  |  | MIR185 |  |  |  | UCN |  |  |  |  |  |  |  |  |  |  |
| SST |  |  | MIR197 |  |  |  | TUBB |  |  |  |  |  |  |  |  |  |  |
| APOA5 |  |  | MIR199B |  |  |  | COL18A1 |  |  |  |  |  |  |  |  |  |  |
| PREPL |  |  | MIR200B |  |  |  | YWHAE |  |  |  |  |  |  |  |  |  |  |
| H19 |  |  | MIR296 |  |  |  | IKBKB |  |  |  |  |  |  |  |  |  |  |
| BCL7B |  |  | MIR30C1 |  |  |  | MEOX1 |  |  |  |  |  |  |  |  |  |  |
| TCTN3 |  |  | MIR30C2 |  |  |  | SGCA |  |  |  |  |  |  |  |  |  |  |
| SLC25A20 |  |  | MIR30E |  |  |  | CCR2 |  |  |  |  |  |  |  |  |  |  |
| NDUFA13 |  |  | MIR33A |  |  |  | MIR29B1 |  |  |  |  |  |  |  |  |  |  |
| MARS1 |  |  | MIR93 |  |  |  | CXCL9 |  |  |  |  |  |  |  |  |  |  |
| TBL2 |  |  | MIR99A |  |  |  | PCK1 |  |  |  |  |  |  |  |  |  |  |
| RBCK1 |  |  | TNFSF12-TNFSF13 |  |  |  | GP1BA |  |  |  |  |  |  |  |  |  |  |
| SALL4 |  |  | MAP6 |  |  |  | F8 |  |  |  |  |  |  |  |  |  |  |
| RPS24 |  |  | ARSD |  |  |  | CTCF |  |  |  |  |  |  |  |  |  |  |
| FHL2 |  |  | MATN1 |  |  |  | MAOA |  |  |  |  |  |  |  |  |  |  |
| KANSL1 |  |  | SMCP |  |  |  | CLU |  |  |  |  |  |  |  |  |  |  |
| SEMA3C |  |  | ME1 |  |  |  | SCN8A |  |  |  |  |  |  |  |  |  |  |
| DKC1 |  |  | MGAT1 |  |  |  | CD68 |  |  |  |  |  |  |  |  |  |  |
| BRIP1 |  |  | MICE |  |  |  | TGM2 |  |  |  |  |  |  |  |  |  |  |
| HAND2-AS1 |  |  | MITF |  |  |  | TLR3 |  |  |  |  |  |  |  |  |  |  |
| NDUFS7 |  |  | MAP3K11 |  |  |  | NGF |  |  |  |  |  |  |  |  |  |  |
| DYNC2LI1 |  |  | AFDN |  |  |  | SPEG |  |  |  |  |  |  |  |  |  |  |
| PMFBP1 |  |  | MMP7 |  |  |  | FKBP1A |  |  |  |  |  |  |  |  |  |  |
| TGIF1 |  |  | MRC1 |  |  |  | APP |  |  |  |  |  |  |  |  |  |  |
| ETFDH |  |  | PLIN5 |  |  |  | SFTPD |  |  |  |  |  |  |  |  |  |  |
| CFAP65 |  |  | POTEKP |  |  |  | ATP2B1 |  |  |  |  |  |  |  |  |  |  |
| LTBP1 |  |  | MIR148B |  |  |  | HCN2 |  |  |  |  |  |  |  |  |  |  |
| CHRND |  |  | MIR324 |  |  |  | SUCLA2 |  |  |  |  |  |  |  |  |  |  |
| FASTKD2 |  |  | TRNT |  |  |  | HDAC9 |  |  |  |  |  |  |  |  |  |  |
| PGBD3 |  |  | MYF6 |  |  |  | TXN |  |  |  |  |  |  |  |  |  |  |
| COX15 |  |  | MYOC |  |  |  | P4HB |  |  |  |  |  |  |  |  |  |  |
| CLCNKB |  |  | PPP1R12A |  |  |  | MIR132 |  |  |  |  |  |  |  |  |  |  |
| SCN4A |  |  | ATF3 |  |  |  | DRD2 |  |  |  |  |  |  |  |  |  |  |
| MSH5-SAPCD1 |  |  | NDUFAB1 |  |  |  | LONP1 |  |  |  |  |  |  |  |  |  |  |
| GTF2IRD2 |  |  | NFE2L1 |  |  |  | SLPI |  |  |  |  |  |  |  |  |  |  |
| SLX4 |  |  | NFIL3 |  |  |  | MUC16 |  |  |  |  |  |  |  |  |  |  |
| BUD23 |  |  | NHS |  |  |  | HAVCR1 |  |  |  |  |  |  |  |  |  |  |
| STAR |  |  | NME3 |  |  |  | DUSP1 |  |  |  |  |  |  |  |  |  |  |
| MPL |  |  | NNMT |  |  |  | MIR30A |  |  |  |  |  |  |  |  |  |  |
| LIMK1 |  |  | ATP2A3 |  |  |  | CSF1 |  |  |  |  |  |  |  |  |  |  |
| PKD1L1 |  |  | NRF1 |  |  |  | XIAP |  |  |  |  |  |  |  |  |  |  |
| IFT80 |  |  | MIR375 |  |  |  | RNASE3 |  |  |  |  |  |  |  |  |  |  |
| RPL11 |  |  | OGN |  |  |  | CD163 |  |  |  |  |  |  |  |  |  |  |
| MYOM2 |  |  | OPRD1 |  |  |  | TAGLN |  |  |  |  |  |  |  |  |  |  |
| NDUFAF6 |  |  | ORM1 |  |  |  | RLN2 |  |  |  |  |  |  |  |  |  |  |
| NDUFAF5 |  |  | ALDH7A1 |  |  |  | CYP1B1 |  |  |  |  |  |  |  |  |  |  |
| SOX11 |  |  | NOX3 |  |  |  | IGFBP2 |  |  |  |  |  |  |  |  |  |  |
| SLC19A2 |  |  | IL22 |  |  |  | AGGF1 |  |  |  |  |  |  |  |  |  |  |
| SDCCAG8 |  |  | PAM |  |  |  | SSB |  |  |  |  |  |  |  |  |  |  |
| FANCE |  |  | AK3 |  |  |  | MAP3K5 |  |  |  |  |  |  |  |  |  |  |
| GJB2 |  |  | NTM |  |  |  | FZD4 |  |  |  |  |  |  |  |  |  |  |
| CD4 |  |  | APIP |  |  |  | IL18BP |  |  |  |  |  |  |  |  |  |  |
| PNLDC1 |  |  | PCMT1 |  |  |  | STAT5B |  |  |  |  |  |  |  |  |  |  |
| STRA6 |  |  | RMDN1 |  |  |  | ITGB1 |  |  |  |  |  |  |  |  |  |  |
| STAT4 |  |  | ANGPTL4 |  |  |  | NCAM1 |  |  |  |  |  |  |  |  |  |  |
| MID1 |  |  | UBAP1 |  |  |  | FOXO3 |  |  |  |  |  |  |  |  |  |  |
| PAX2 |  |  | BFAR |  |  |  | ABCB7 |  |  |  |  |  |  |  |  |  |  |
| FRAS1 |  |  | PDC |  |  |  | AKR1B1 |  |  |  |  |  |  |  |  |  |  |
| NFIX |  |  | PDE1C |  |  |  | ADRA1A |  |  |  |  |  |  |  |  |  |  |
| CHST3 |  |  | TRPV2 |  |  |  | MIR221 |  |  |  |  |  |  |  |  |  |  |
| BNC1 |  |  | SUCO |  |  |  | PPP1R3A |  |  |  |  |  |  |  |  |  |  |
| GJC1 |  |  | UFM1 |  |  |  | MYLK3 |  |  |  |  |  |  |  |  |  |  |
| PTGIS |  |  | GDE1 |  |  |  | LIF |  |  |  |  |  |  |  |  |  |  |
| NPHS2 |  |  | NLK |  |  |  | AMBP |  |  |  |  |  |  |  |  |  |  |
| KCNE3 |  |  | CMPK1 |  |  |  | RBM24 |  |  |  |  |  |  |  |  |  |  |
| TTC21A |  |  | PFN1 |  |  |  | FTH1 |  |  |  |  |  |  |  |  |  |  |
| ADAMTSL2 |  |  | PIGF |  |  |  | TBK1 |  |  |  |  |  |  |  |  |  |  |
| LMOD3 |  |  | PITX3 |  |  |  | SRI |  |  |  |  |  |  |  |  |  |  |
| CATSPER1 |  |  | TPCN1 |  |  |  | CLCN3 |  |  |  |  |  |  |  |  |  |  |
| PEX2 |  |  | CNTN5 |  |  |  | PTPN1 |  |  |  |  |  |  |  |  |  |  |
| DNAH9 |  |  | TLR9 |  |  |  | TIMP3 |  |  |  |  |  |  |  |  |  |  |
| DNAH11 |  |  | CYTL1 |  |  |  | SF3B1 |  |  |  |  |  |  |  |  |  |  |
| HLA-DPB1 |  |  | DDIT4 |  |  |  | BIN1 |  |  |  |  |  |  |  |  |  |  |
| GTF2I |  |  | EGLN1 |  |  |  | ATXN1 |  |  |  |  |  |  |  |  |  |  |
| PLEC |  |  | PPA1 |  |  |  | PGK1 |  |  |  |  |  |  |  |  |  |  |
| LIG4 |  |  | PPARD |  |  |  | SLC5A2 |  |  |  |  |  |  |  |  |  |  |
| NDUFAF3 |  |  | PPID |  |  |  | LAMC2 |  |  |  |  |  |  |  |  |  |  |
| BSCL2 |  |  | MARCHF1 |  |  |  | ESRRA |  |  |  |  |  |  |  |  |  |  |
| PLA2G7 |  |  | MOCOS |  |  |  | GAD1 |  |  |  |  |  |  |  |  |  |  |
| SLC25A26 |  |  | WIPI1 |  |  |  | TNC |  |  |  |  |  |  |  |  |  |  |
| ABCG5 |  |  | SLC52A1 |  |  |  | GSTP1 |  |  |  |  |  |  |  |  |  |  |
| STAG2 |  |  | PPP1R7 |  |  |  | CTSK |  |  |  |  |  |  |  |  |  |  |
| SOX2 |  |  | RMDN3 |  |  |  | MCIDAS |  |  |  |  |  |  |  |  |  |  |
| DNAJC30 |  |  | PPP2R1A |  |  |  | PRKAA2 |  |  |  |  |  |  |  |  |  |  |
| AGPAT2 |  |  | ADI1 |  |  |  | CD44 |  |  |  |  |  |  |  |  |  |  |
| SOX10 |  |  | AVPR1B |  |  |  | HSF2 |  |  |  |  |  |  |  |  |  |  |
| PDHA2 |  |  | PPP5C |  |  |  | RBFOX2 |  |  |  |  |  |  |  |  |  |  |
| SCYL1 |  |  | IMPACT |  |  |  | ACP5 |  |  |  |  |  |  |  |  |  |  |
| GNRH1 |  |  | PPT1 |  |  |  | GC |  |  |  |  |  |  |  |  |  |  |
| TXNRD2 |  |  | NPY4R |  |  |  | ATP1A3 |  |  |  |  |  |  |  |  |  |  |
| TFAP2B |  |  | SRGN |  |  |  | CFLAR |  |  |  |  |  |  |  |  |  |  |
| MSX1 |  |  | SLC30A10 |  |  |  | CTSB |  |  |  |  |  |  |  |  |  |  |
| TTC29 |  |  | SYBU |  |  |  | APOC1 |  |  |  |  |  |  |  |  |  |  |
| MT-TN |  |  | ENAH |  |  |  | ST2 |  |  |  |  |  |  |  |  |  |  |
| RRAS |  |  | DCAF6 |  |  |  | OGDH |  |  |  |  |  |  |  |  |  |  |
| HIRA |  |  | PKN1 |  |  |  | PLAU |  |  |  |  |  |  |  |  |  |  |
| NUP188 |  |  | USE1 |  |  |  | AVPR1A |  |  |  |  |  |  |  |  |  |  |
| BBS7 |  |  | BEX1 |  |  |  | ITPR1 |  |  |  |  |  |  |  |  |  |  |
| TDGF1 |  |  | PKN2 |  |  |  | ITGB1BP2 |  |  |  |  |  |  |  |  |  |  |
| TNXB |  |  | ZKSCAN7 |  |  |  | HSD11B1 |  |  |  |  |  |  |  |  |  |  |
| SLC29A3 |  |  | ZC4H2 |  |  |  | FGF13 |  |  |  |  |  |  |  |  |  |  |
| CFAP69 |  |  | MAPK9 |  |  |  | PLA2G2A |  |  |  |  |  |  |  |  |  |  |
| FANCB |  |  | MAP2K3 |  |  |  | MS4A1 |  |  |  |  |  |  |  |  |  |  |
| SNTA1 |  |  | YLPM1 |  |  |  | POLR2A |  |  |  |  |  |  |  |  |  |  |
| BBIP1 |  |  | METTL3 |  |  |  | UCP1 |  |  |  |  |  |  |  |  |  |  |
| HBA2 |  |  | KCNK13 |  |  |  | MMP8 |  |  |  |  |  |  |  |  |  |  |
| EPRS1 |  |  | MAP3K7CL |  |  |  | PRKCB |  |  |  |  |  |  |  |  |  |  |
| ELAC2 |  |  | FAM20C |  |  |  | BACE1-AS |  |  |  |  |  |  |  |  |  |  |
| WFS1 |  |  | CHPT1 |  |  |  | RBM25 |  |  |  |  |  |  |  |  |  |  |
| SMC3 |  |  | PLSCR4 |  |  |  | ADCY6 |  |  |  |  |  |  |  |  |  |  |
| GLI1 |  |  | BAD |  |  |  | GLP1R |  |  |  |  |  |  |  |  |  |  |
| ANO5 |  |  | MIR409 |  |  |  | NRIP1 |  |  |  |  |  |  |  |  |  |  |
| NONO |  |  | NLN |  |  |  | IL11 |  |  |  |  |  |  |  |  |  |  |
| MTHFD1 |  |  | KCNT1 |  |  |  | RGS4 |  |  |  |  |  |  |  |  |  |  |
| HDAC8 |  |  | DPP10 |  |  |  | S100A12 |  |  |  |  |  |  |  |  |  |  |
| ARVCF |  |  | PTN |  |  |  | NRON |  |  |  |  |  |  |  |  |  |  |
| BMP6 |  |  | PURB |  |  |  | SLC24A3 |  |  |  |  |  |  |  |  |  |  |
| MT-TE |  |  | PVALB |  |  |  | OPRM1 |  |  |  |  |  |  |  |  |  |  |
| FH |  |  | CXCL16 |  |  |  | NISCH |  |  |  |  |  |  |  |  |  |  |
| LOC102723566 |  |  | RHOU |  |  |  | KLF13 |  |  |  |  |  |  |  |  |  |  |
| TTC8 |  |  | RAB1A |  |  |  | CYP2B6 |  |  |  |  |  |  |  |  |  |  |
| NKX2-1 |  |  | RARRES2 |  |  |  | CYP2E1 |  |  |  |  |  |  |  |  |  |  |
| GALC |  |  | CACNG6 |  |  |  | EMC10 |  |  |  |  |  |  |  |  |  |  |
| BBS4 |  |  | BCL2A1 |  |  |  | NFATC2 |  |  |  |  |  |  |  |  |  |  |
| HBN1 |  |  | RELA |  |  |  | SIRT3 |  |  |  |  |  |  |  |  |  |  |
| CCNH |  |  | RENBP |  |  |  | UTS2R |  |  |  |  |  |  |  |  |  |  |
| PTPRC |  |  | RHD |  |  |  | GPX4 |  |  |  |  |  |  |  |  |  |  |
| SKIC2 |  |  | RORC |  |  |  | QRSL1 |  |  |  |  |  |  |  |  |  |  |
| RFC2 |  |  | RPE65 |  |  |  | SLC5A1 |  |  |  |  |  |  |  |  |  |  |
| TMEM216 |  |  | RPL32 |  |  |  | GAL |  |  |  |  |  |  |  |  |  |  |
| GNB2 |  |  | S100A8 |  |  |  | PMP22 |  |  |  |  |  |  |  |  |  |  |
| MTX2 |  |  | SAT1 |  |  |  | ARSL |  |  |  |  |  |  |  |  |  |  |
| M1AP |  |  | SCN7A |  |  |  | ANXA6 |  |  |  |  |  |  |  |  |  |  |
| LOC107988032 |  |  | SDC1 |  |  |  | DGAT1 |  |  |  |  |  |  |  |  |  |  |
| COG2 |  |  | POTEM |  |  |  | XRCC6 |  |  |  |  |  |  |  |  |  |  |
| CBL |  |  | SGTA |  |  |  | TFEB |  |  |  |  |  |  |  |  |  |  |
| CLIP2 |  |  | GIGYF1 |  |  |  | CTSG |  |  |  |  |  |  |  |  |  |  |
| MT-TV |  |  | GORASP1 |  |  |  | FOXO1 |  |  |  |  |  |  |  |  |  |  |
| NPHS1 |  |  | UBE2Z |  |  |  | YAP1 |  |  |  |  |  |  |  |  |  |  |
| IFT27 |  |  | SLC18A3 |  |  |  | KCNA2 |  |  |  |  |  |  |  |  |  |  |
| CCDC40 |  |  | SMPD2 |  |  |  | WNK1 |  |  |  |  |  |  |  |  |  |  |
| TNFSF4 |  |  | SUMO3 |  |  |  | OGA |  |  |  |  |  |  |  |  |  |  |
| IL7 |  |  | SUMO2 |  |  |  | CTH |  |  |  |  |  |  |  |  |  |  |
| TNNT1 |  |  | SOAT1 |  |  |  | ITPR3 |  |  |  |  |  |  |  |  |  |  |
| AOPEP |  |  | MIR539 |  |  |  | FADS2 |  |  |  |  |  |  |  |  |  |  |
| MYH9 |  |  | SPARC |  |  |  | ALAS2 |  |  |  |  |  |  |  |  |  |  |
| BCHE |  |  | SPRR2B |  |  |  | VEGFB |  |  |  |  |  |  |  |  |  |  |
| DGKE |  |  | STAT5A |  |  |  | PDK4 |  |  |  |  |  |  |  |  |  |  |
| VPS37D |  |  | STC1 |  |  |  | DDAH1 |  |  |  |  |  |  |  |  |  |  |
| MIR122 |  |  | SULT1E1 |  |  |  | CXCR3 |  |  |  |  |  |  |  |  |  |  |
| MT-TI |  |  | STK11 |  |  |  | CFD |  |  |  |  |  |  |  |  |  |  |
| TPM3 |  |  | STRN |  |  |  | MCU |  |  |  |  |  |  |  |  |  |  |
| EIF4H |  |  | SYK |  |  |  | RGS2 |  |  |  |  |  |  |  |  |  |  |
| AURKC |  |  | BTD |  |  |  | ABCG2 |  |  |  |  |  |  |  |  |  |  |
| MIR133A1 |  |  | TEAD1 |  |  |  | GRIN1 |  |  |  |  |  |  |  |  |  |  |
| CCL3 |  |  | TERF2 |  |  |  | KLF15 |  |  |  |  |  |  |  |  |  |  |
| HNRNPA1 |  |  | TSPO |  |  |  | UCP3 |  |  |  |  |  |  |  |  |  |  |
| PRDM6 |  |  | TMSB4X |  |  |  | HDAC2 |  |  |  |  |  |  |  |  |  |  |
| WRN |  |  | TOP2B |  |  |  | TRPV1 |  |  |  |  |  |  |  |  |  |  |
| ERCC2 |  |  | TPT1 |  |  |  | DSPP |  |  |  |  |  |  |  |  |  |  |
| CEP112 |  |  | CRISP2 |  |  |  | PROC |  |  |  |  |  |  |  |  |  |  |
| HACD1 |  |  | TRAF2 |  |  |  | FADS1 |  |  |  |  |  |  |  |  |  |  |
| DAZ1 |  |  | TRAF3 |  |  |  | TRPC3 |  |  |  |  |  |  |  |  |  |  |
| ALPL |  |  | C3AR1 |  |  |  | TRPC1 |  |  |  |  |  |  |  |  |  |  |
| SREBF1 |  |  | MIR650 |  |  |  | SGK1 |  |  |  |  |  |  |  |  |  |  |
| CDH13 |  |  | MIR652 |  |  |  | CDK5RAP3 |  |  |  |  |  |  |  |  |  |  |
| EFEMP2 |  |  | C5AR1 |  |  |  | TIMM50 |  |  |  |  |  |  |  |  |  |  |
| MIPEP |  |  | OPN1MW2 |  |  |  | ANG |  |  |  |  |  |  |  |  |  |  |
| DISP1 |  |  | UGT2B7 |  |  |  | SLN |  |  |  |  |  |  |  |  |  |  |
| PPP1R13L |  |  | UVRAG |  |  |  | GSK3B |  |  |  |  |  |  |  |  |  |  |
| LBR |  |  | VDI |  |  |  | ADM2 |  |  |  |  |  |  |  |  |  |  |
| HSPA1A |  |  | VSNL1 |  |  |  | MIR19B1 |  |  |  |  |  |  |  |  |  |  |
| DGUOK |  |  | WNT1 |  |  |  | SYT1 |  |  |  |  |  |  |  |  |  |  |
| RBPJ |  |  | WNT2 |  |  |  | FRZB |  |  |  |  |  |  |  |  |  |  |
| CUBN |  |  | XIST |  |  |  | SIX1 |  |  |  |  |  |  |  |  |  |  |
| CTC1 |  |  | XRCC1 |  |  |  | CHI3L1 |  |  |  |  |  |  |  |  |  |  |
| NPHP4 |  |  | CA3 |  |  |  | BNIP3 |  |  |  |  |  |  |  |  |  |  |
| VIM |  |  | PCGF2 |  |  |  | CCK |  |  |  |  |  |  |  |  |  |  |
| ADA2 |  |  | ZBTB17 |  |  |  | GHRH |  |  |  |  |  |  |  |  |  |  |
| AK7 |  |  | MAP3K12 |  |  |  | MIR106B |  |  |  |  |  |  |  |  |  |  |
| ASCL1 |  |  | SCG2 |  |  |  | PPP1R1A |  |  |  |  |  |  |  |  |  |  |
| CHRNA1 |  |  | SLMAP |  |  |  | SLC6A8 |  |  |  |  |  |  |  |  |  |  |
| PSAP |  |  | MANF |  |  |  | TRAF6 |  |  |  |  |  |  |  |  |  |  |
| MIB1 |  |  | REEP5 |  |  |  | AXL |  |  |  |  |  |  |  |  |  |  |
| GAS1 |  |  | TMEM109 |  |  |  | TJP1 |  |  |  |  |  |  |  |  |  |  |
| DHCR7 |  |  | MMP28 |  |  |  | KMT2B |  |  |  |  |  |  |  |  |  |  |
| CAVIN1 |  |  | LAP |  |  |  | ANXA2 |  |  |  |  |  |  |  |  |  |  |
| COX4I1 |  |  | MYH14 |  |  |  | FRMD4B |  |  |  |  |  |  |  |  |  |  |
| MTRR |  |  | SHCBP1 |  |  |  |  |  |  |  |  |  |  |  |  |  |  |
| COX6B1 |  |  | CAAP1 |  |  |  |  |  |  |  |  |  |  |  |  |  |  |
| MAD2L2 |  |  | DGLUCY |  |  |  |  |  |  |  |  |  |  |  |  |  |  |
| APOA2 |  |  | ZC3H12A |  |  |  |  |  |  |  |  |  |  |  |  |  |  |
| CLCN1 |  |  | TCL1A |  |  |  |  |  |  |  |  |  |  |  |  |  |  |
| F12 |  |  | TXNDC5 |  |  |  |  |  |  |  |  |  |  |  |  |  |  |
| IL13 |  |  | UNC93B1 |  |  |  |  |  |  |  |  |  |  |  |  |  |  |
| NHLRC2 |  |  | AKAP1 |  |  |  |  |  |  |  |  |  |  |  |  |  |  |
| ACVR2B |  |  | CAPG |  |  |  |  |  |  |  |  |  |  |  |  |  |  |
| SPTB |  |  | CAST |  |  |  |  |  |  |  |  |  |  |  |  |  |  |
| HYDIN |  |  | BCL2L12 |  |  |  |  |  |  |  |  |  |  |  |  |  |  |
| CFB |  |  | EVA1A |  |  |  |  |  |  |  |  |  |  |  |  |  |  |
| GCNA |  |  | TCHP |  |  |  |  |  |  |  |  |  |  |  |  |  |  |
| SQSTM1 |  |  | SPZ1 |  |  |  |  |  |  |  |  |  |  |  |  |  |  |
| IFNA1 |  |  | ATP5MD |  |  |  |  |  |  |  |  |  |  |  |  |  |  |
| KCNE5 |  |  | ORAI1 |  |  |  |  |  |  |  |  |  |  |  |  |  |  |
| EIF2B2 |  |  | ADO |  |  |  |  |  |  |  |  |  |  |  |  |  |  |
| MYD88 |  |  | PLPP3 |  |  |  |  |  |  |  |  |  |  |  |  |  |  |
| TMEM70 |  |  | STC2 |  |  |  |  |  |  |  |  |  |  |  |  |  |  |
| KIAA0586 |  |  | PSMG1 |  |  |  |  |  |  |  |  |  |  |  |  |  |  |
| CALM3 |  |  | URI1 |  |  |  |  |  |  |  |  |  |  |  |  |  |  |
| PEX1 |  |  | TNFSF10 |  |  |  |  |  |  |  |  |  |  |  |  |  |  |
| AHI1 |  |  | CDS2 |  |  |  |  |  |  |  |  |  |  |  |  |  |  |
| XRCC4 |  |  | PROM1 |  |  |  |  |  |  |  |  |  |  |  |  |  |  |
| DOLK |  |  | NR1I2 |  |  |  |  |  |  |  |  |  |  |  |  |  |  |
| NAA10 |  |  | SPHK1 |  |  |  |  |  |  |  |  |  |  |  |  |  |  |
| JUN |  |  | NAE1 |  |  |  |  |  |  |  |  |  |  |  |  |  |  |
| MC2R |  |  | WASF1 |  |  |  |  |  |  |  |  |  |  |  |  |  |  |
| DNMT3B |  |  | MLIP |  |  |  |  |  |  |  |  |  |  |  |  |  |  |
| SGO1 |  |  | CLDN10 |  |  |  |  |  |  |  |  |  |  |  |  |  |  |
| CACNA1S |  |  | DPP9 |  |  |  |  |  |  |  |  |  |  |  |  |  |  |
| MDM4 |  |  | HGS |  |  |  |  |  |  |  |  |  |  |  |  |  |  |
| CAPN3 |  |  | MAP3K13 |  |  |  |  |  |  |  |  |  |  |  |  |  |  |
| ATP1B1 |  |  | XPR1 |  |  |  |  |  |  |  |  |  |  |  |  |  |  |
| PPP2R3C |  |  | MTG1 |  |  |  |  |  |  |  |  |  |  |  |  |  |  |
| SCNN1B |  |  | IL32 |  |  |  |  |  |  |  |  |  |  |  |  |  |  |
| PROKR2 |  |  | MSC |  |  |  |  |  |  |  |  |  |  |  |  |  |  |
| RPA1 |  |  | ORAI3 |  |  |  |  |  |  |  |  |  |  |  |  |  |  |
| MIR378A |  |  | CGB5 |  |  |  |  |  |  |  |  |  |  |  |  |  |  |
| SIX3 |  |  | TSIX |  |  |  |  |  |  |  |  |  |  |  |  |  |  |
| SOX3 |  |  | CGB8 |  |  |  |  |  |  |  |  |  |  |  |  |  |  |
| ATP8B1 |  |  | OPN4 |  |  |  |  |  |  |  |  |  |  |  |  |  |  |
| KCNAB2 |  |  | TNFRSF8 |  |  |  |  |  |  |  |  |  |  |  |  |  |  |
| IRF5 |  |  | FHL5 |  |  |  |  |  |  |  |  |  |  |  |  |  |  |
| RREB1 |  |  | TBPL1 |  |  |  |  |  |  |  |  |  |  |  |  |  |  |
| IRX4 |  |  | BCAR1 |  |  |  |  |  |  |  |  |  |  |  |  |  |  |
| GNAS |  |  | AKAP12 |  |  |  |  |  |  |  |  |  |  |  |  |  |  |
| TRIM63 |  |  | ISG15 |  |  |  |  |  |  |  |  |  |  |  |  |  |  |
| TACO1 |  |  | TGS1 |  |  |  |  |  |  |  |  |  |  |  |  |  |  |
| MVK |  |  | FAM53B |  |  |  |  |  |  |  |  |  |  |  |  |  |  |
| ZMYND15 |  |  | CD69 |  |  |  |  |  |  |  |  |  |  |  |  |  |  |
| TERB1 |  |  | MLEC |  |  |  |  |  |  |  |  |  |  |  |  |  |  |
| CDON |  |  | PIEZO1 |  |  |  |  |  |  |  |  |  |  |  |  |  |  |
| METTL27 |  |  | BMS1 |  |  |  |  |  |  |  |  |  |  |  |  |  |  |
| KIF1B |  |  | CCS |  |  |  |  |  |  |  |  |  |  |  |  |  |  |
| AMHR2 |  |  |  |  |  |  |  |  |  |  |  |  |  |  |  |  |  |
| NDUFAF1 |  |  |  |  |  |  |  |  |  |  |  |  |  |  |  |  |  |
| DYRK1A |  |  |  |  |  |  |  |  |  |  |  |  |  |  |  |  |  |
| TEK |  |  |  |  |  |  |  |  |  |  |  |  |  |  |  |  |  |
| ALG9 |  |  |  |  |  |  |  |  |  |  |  |  |  |  |  |  |  |
| HNRNPA2B1 |  |  |  |  |  |  |  |  |  |  |  |  |  |  |  |  |  |
| BBS5 |  |  |  |  |  |  |  |  |  |  |  |  |  |  |  |  |  |
| CPS1 |  |  |  |  |  |  |  |  |  |  |  |  |  |  |  |  |  |
| BTK |  |  |  |  |  |  |  |  |  |  |  |  |  |  |  |  |  |
| DLL4 |  |  |  |  |  |  |  |  |  |  |  |  |  |  |  |  |  |
| FBXL4 |  |  |  |  |  |  |  |  |  |  |  |  |  |  |  |  |  |
| APOH |  |  |  |  |  |  |  |  |  |  |  |  |  |  |  |  |  |
| NDUFS1 |  |  |  |  |  |  |  |  |  |  |  |  |  |  |  |  |  |
| CATIP |  |  |  |  |  |  |  |  |  |  |  |  |  |  |  |  |  |
| DARS2 |  |  |  |  |  |  |  |  |  |  |  |  |  |  |  |  |  |
| IRS1 |  |  |  |  |  |  |  |  |  |  |  |  |  |  |  |  |  |
| CTNS |  |  |  |  |  |  |  |  |  |  |  |  |  |  |  |  |  |
| RAPSN |  |  |  |  |  |  |  |  |  |  |  |  |  |  |  |  |  |
| C9orf72 |  |  |  |  |  |  |  |  |  |  |  |  |  |  |  |  |  |
| CXADR |  |  |  |  |  |  |  |  |  |  |  |  |  |  |  |  |  |
| DSG2-AS1 |  |  |  |  |  |  |  |  |  |  |  |  |  |  |  |  |  |
| RPS6KA3 |  |  |  |  |  |  |  |  |  |  |  |  |  |  |  |  |  |
| TPM2 |  |  |  |  |  |  |  |  |  |  |  |  |  |  |  |  |  |
| CTBP1 |  |  |  |  |  |  |  |  |  |  |  |  |  |  |  |  |  |
| SLC12A3 |  |  |  |  |  |  |  |  |  |  |  |  |  |  |  |  |  |
| COX8A |  |  |  |  |  |  |  |  |  |  |  |  |  |  |  |  |  |
| TLR5 |  |  |  |  |  |  |  |  |  |  |  |  |  |  |  |  |  |
| G6PC3 |  |  |  |  |  |  |  |  |  |  |  |  |  |  |  |  |  |
| ARID2 |  |  |  |  |  |  |  |  |  |  |  |  |  |  |  |  |  |
| NFU1 |  |  |  |  |  |  |  |  |  |  |  |  |  |  |  |  |  |
| CFAP53 |  |  |  |  |  |  |  |  |  |  |  |  |  |  |  |  |  |
| PEX26 |  |  |  |  |  |  |  |  |  |  |  |  |  |  |  |  |  |
| MMVP1 |  |  |  |  |  |  |  |  |  |  |  |  |  |  |  |  |  |
| THPO |  |  |  |  |  |  |  |  |  |  |  |  |  |  |  |  |  |
| NDUFA1 |  |  |  |  |  |  |  |  |  |  |  |  |  |  |  |  |  |
| DNAH8-AS1 |  |  |  |  |  |  |  |  |  |  |  |  |  |  |  |  |  |
| MTFMT |  |  |  |  |  |  |  |  |  |  |  |  |  |  |  |  |  |
| SRC |  |  |  |  |  |  |  |  |  |  |  |  |  |  |  |  |  |
| DNASE1L1 |  |  |  |  |  |  |  |  |  |  |  |  |  |  |  |  |  |
| GALNS |  |  |  |  |  |  |  |  |  |  |  |  |  |  |  |  |  |
| TMEM270 |  |  |  |  |  |  |  |  |  |  |  |  |  |  |  |  |  |
| NHP2 |  |  |  |  |  |  |  |  |  |  |  |  |  |  |  |  |  |
| ZFPM2-AS1 |  |  |  |  |  |  |  |  |  |  |  |  |  |  |  |  |  |
| ACVR1 |  |  |  |  |  |  |  |  |  |  |  |  |  |  |  |  |  |
| ALDH18A1 |  |  |  |  |  |  |  |  |  |  |  |  |  |  |  |  |  |
| SKI |  |  |  |  |  |  |  |  |  |  |  |  |  |  |  |  |  |
| BOLA3 |  |  |  |  |  |  |  |  |  |  |  |  |  |  |  |  |  |
| CEP120 |  |  |  |  |  |  |  |  |  |  |  |  |  |  |  |  |  |
| XYLT1 |  |  |  |  |  |  |  |  |  |  |  |  |  |  |  |  |  |
| GP1BB |  |  |  |  |  |  |  |  |  |  |  |  |  |  |  |  |  |
| CFAP91 |  |  |  |  |  |  |  |  |  |  |  |  |  |  |  |  |  |
| TCF4 |  |  |  |  |  |  |  |  |  |  |  |  |  |  |  |  |  |
| PHYH |  |  |  |  |  |  |  |  |  |  |  |  |  |  |  |  |  |
| PIGG |  |  |  |  |  |  |  |  |  |  |  |  |  |  |  |  |  |
| LEMD3 |  |  |  |  |  |  |  |  |  |  |  |  |  |  |  |  |  |
| SYCP2 |  |  |  |  |  |  |  |  |  |  |  |  |  |  |  |  |  |
| RPL3L |  |  |  |  |  |  |  |  |  |  |  |  |  |  |  |  |  |
| PET100 |  |  |  |  |  |  |  |  |  |  |  |  |  |  |  |  |  |
| TSGA10 |  |  |  |  |  |  |  |  |  |  |  |  |  |  |  |  |  |
| SPINK2 |  |  |  |  |  |  |  |  |  |  |  |  |  |  |  |  |  |
| SUCLG1 |  |  |  |  |  |  |  |  |  |  |  |  |  |  |  |  |  |
| ATRX |  |  |  |  |  |  |  |  |  |  |  |  |  |  |  |  |  |
| ZIC2 |  |  |  |  |  |  |  |  |  |  |  |  |  |  |  |  |  |
| DNAAF11 |  |  |  |  |  |  |  |  |  |  |  |  |  |  |  |  |  |
| DVL3 |  |  |  |  |  |  |  |  |  |  |  |  |  |  |  |  |  |
| SPEN |  |  |  |  |  |  |  |  |  |  |  |  |  |  |  |  |  |
| DYNC2I2 |  |  |  |  |  |  |  |  |  |  |  |  |  |  |  |  |  |
| AMH |  |  |  |  |  |  |  |  |  |  |  |  |  |  |  |  |  |
| GPC3 |  |  |  |  |  |  |  |  |  |  |  |  |  |  |  |  |  |
| WRAP53 |  |  |  |  |  |  |  |  |  |  |  |  |  |  |  |  |  |
| SNRPN |  |  |  |  |  |  |  |  |  |  |  |  |  |  |  |  |  |
| PURA |  |  |  |  |  |  |  |  |  |  |  |  |  |  |  |  |  |
| ODAD2 |  |  |  |  |  |  |  |  |  |  |  |  |  |  |  |  |  |
| PBX1 |  |  |  |  |  |  |  |  |  |  |  |  |  |  |  |  |  |
| SNCA |  |  |  |  |  |  |  |  |  |  |  |  |  |  |  |  |  |
| SUFU |  |  |  |  |  |  |  |  |  |  |  |  |  |  |  |  |  |
| NANOS1 |  |  |  |  |  |  |  |  |  |  |  |  |  |  |  |  |  |
| ATP7B |  |  |  |  |  |  |  |  |  |  |  |  |  |  |  |  |  |
| FLI1 |  |  |  |  |  |  |  |  |  |  |  |  |  |  |  |  |  |
| SEC24C |  |  |  |  |  |  |  |  |  |  |  |  |  |  |  |  |  |
| MIAT |  |  |  |  |  |  |  |  |  |  |  |  |  |  |  |  |  |
| MATR3 |  |  |  |  |  |  |  |  |  |  |  |  |  |  |  |  |  |
| TEX14 |  |  |  |  |  |  |  |  |  |  |  |  |  |  |  |  |  |
| NDE1 |  |  |  |  |  |  |  |  |  |  |  |  |  |  |  |  |  |
| RAB3GAP2 |  |  |  |  |  |  |  |  |  |  |  |  |  |  |  |  |  |
| IFT43 |  |  |  |  |  |  |  |  |  |  |  |  |  |  |  |  |  |
| RPS10 |  |  |  |  |  |  |  |  |  |  |  |  |  |  |  |  |  |
| EYA1 |  |  |  |  |  |  |  |  |  |  |  |  |  |  |  |  |  |
| ATP1A2 |  |  |  |  |  |  |  |  |  |  |  |  |  |  |  |  |  |
| MYL1 |  |  |  |  |  |  |  |  |  |  |  |  |  |  |  |  |  |
| MYL9 |  |  |  |  |  |  |  |  |  |  |  |  |  |  |  |  |  |
| HCCS |  |  |  |  |  |  |  |  |  |  |  |  |  |  |  |  |  |
| ETV2 |  |  |  |  |  |  |  |  |  |  |  |  |  |  |  |  |  |
| BLK |  |  |  |  |  |  |  |  |  |  |  |  |  |  |  |  |  |
| COL6A1 |  |  |  |  |  |  |  |  |  |  |  |  |  |  |  |  |  |
| MTM1 |  |  |  |  |  |  |  |  |  |  |  |  |  |  |  |  |  |
| SCN1A |  |  |  |  |  |  |  |  |  |  |  |  |  |  |  |  |  |
| MCM4 |  |  |  |  |  |  |  |  |  |  |  |  |  |  |  |  |  |
| PIEZO2 |  |  |  |  |  |  |  |  |  |  |  |  |  |  |  |  |  |
| CCDC65 |  |  |  |  |  |  |  |  |  |  |  |  |  |  |  |  |  |
| GPX3 |  |  |  |  |  |  |  |  |  |  |  |  |  |  |  |  |  |
| RFWD3 |  |  |  |  |  |  |  |  |  |  |  |  |  |  |  |  |  |
| CYBA |  |  |  |  |  |  |  |  |  |  |  |  |  |  |  |  |  |
| PAX6 |  |  |  |  |  |  |  |  |  |  |  |  |  |  |  |  |  |
| RPL10L |  |  |  |  |  |  |  |  |  |  |  |  |  |  |  |  |  |
| FSIP2 |  |  |  |  |  |  |  |  |  |  |  |  |  |  |  |  |  |
| MUSK |  |  |  |  |  |  |  |  |  |  |  |  |  |  |  |  |  |
| LGALS2 |  |  |  |  |  |  |  |  |  |  |  |  |  |  |  |  |  |
| UFD1 |  |  |  |  |  |  |  |  |  |  |  |  |  |  |  |  |  |
| TMEM126B |  |  |  |  |  |  |  |  |  |  |  |  |  |  |  |  |  |
| HMGA2 |  |  |  |  |  |  |  |  |  |  |  |  |  |  |  |  |  |
| OTUD6B |  |  |  |  |  |  |  |  |  |  |  |  |  |  |  |  |  |
| SMARCB1 |  |  |  |  |  |  |  |  |  |  |  |  |  |  |  |  |  |
| CYP17A1 |  |  |  |  |  |  |  |  |  |  |  |  |  |  |  |  |  |
| PUS3 |  |  |  |  |  |  |  |  |  |  |  |  |  |  |  |  |  |
| AFP |  |  |  |  |  |  |  |  |  |  |  |  |  |  |  |  |  |
| ITGA7 |  |  |  |  |  |  |  |  |  |  |  |  |  |  |  |  |  |
| YY1AP1 |  |  |  |  |  |  |  |  |  |  |  |  |  |  |  |  |  |
| EGF |  |  |  |  |  |  |  |  |  |  |  |  |  |  |  |  |  |
| PIGA |  |  |  |  |  |  |  |  |  |  |  |  |  |  |  |  |  |
| GUCY2C |  |  |  |  |  |  |  |  |  |  |  |  |  |  |  |  |  |
| CKM |  |  |  |  |  |  |  |  |  |  |  |  |  |  |  |  |  |
| ETFA |  |  |  |  |  |  |  |  |  |  |  |  |  |  |  |  |  |
| ETFB |  |  |  |  |  |  |  |  |  |  |  |  |  |  |  |  |  |
| HSF2BP |  |  |  |  |  |  |  |  |  |  |  |  |  |  |  |  |  |
| DNAAF3 |  |  |  |  |  |  |  |  |  |  |  |  |  |  |  |  |  |
| BBS12 |  |  |  |  |  |  |  |  |  |  |  |  |  |  |  |  |  |
| USP9Y |  |  |  |  |  |  |  |  |  |  |  |  |  |  |  |  |  |
| LEPQTL1 |  |  |  |  |  |  |  |  |  |  |  |  |  |  |  |  |  |
| UQCRFS1 |  |  |  |  |  |  |  |  |  |  |  |  |  |  |  |  |  |
| GCLC |  |  |  |  |  |  |  |  |  |  |  |  |  |  |  |  |  |
| LAMA4 |  |  |  |  |  |  |  |  |  |  |  |  |  |  |  |  |  |
| PDPN |  |  |  |  |  |  |  |  |  |  |  |  |  |  |  |  |  |
| LDLRAP1 |  |  |  |  |  |  |  |  |  |  |  |  |  |  |  |  |  |
| DGCR8 |  |  |  |  |  |  |  |  |  |  |  |  |  |  |  |  |  |
| PEX3 |  |  |  |  |  |  |  |  |  |  |  |  |  |  |  |  |  |
| SKIC3 |  |  |  |  |  |  |  |  |  |  |  |  |  |  |  |  |  |
| CD55 |  |  |  |  |  |  |  |  |  |  |  |  |  |  |  |  |  |
| ERBB3 |  |  |  |  |  |  |  |  |  |  |  |  |  |  |  |  |  |
| EIF2AK4 |  |  |  |  |  |  |  |  |  |  |  |  |  |  |  |  |  |
| MADD |  |  |  |  |  |  |  |  |  |  |  |  |  |  |  |  |  |
| HSD17B4 |  |  |  |  |  |  |  |  |  |  |  |  |  |  |  |  |  |
| HOTAIR |  |  |  |  |  |  |  |  |  |  |  |  |  |  |  |  |  |
| LRP8 |  |  |  |  |  |  |  |  |  |  |  |  |  |  |  |  |  |
| DLK1 |  |  |  |  |  |  |  |  |  |  |  |  |  |  |  |  |  |
| MT-TF |  |  |  |  |  |  |  |  |  |  |  |  |  |  |  |  |  |
| MYH8 |  |  |  |  |  |  |  |  |  |  |  |  |  |  |  |  |  |
| CCDC39 |  |  |  |  |  |  |  |  |  |  |  |  |  |  |  |  |  |
| TMEM237 |  |  |  |  |  |  |  |  |  |  |  |  |  |  |  |  |  |
| FREM2 |  |  |  |  |  |  |  |  |  |  |  |  |  |  |  |  |  |
| AKR1D1 |  |  |  |  |  |  |  |  |  |  |  |  |  |  |  |  |  |
| GATA2 |  |  |  |  |  |  |  |  |  |  |  |  |  |  |  |  |  |
| CD19 |  |  |  |  |  |  |  |  |  |  |  |  |  |  |  |  |  |
| ABCG8 |  |  |  |  |  |  |  |  |  |  |  |  |  |  |  |  |  |
| PON3 |  |  |  |  |  |  |  |  |  |  |  |  |  |  |  |  |  |
| NEK10 |  |  |  |  |  |  |  |  |  |  |  |  |  |  |  |  |  |
| CLN3 |  |  |  |  |  |  |  |  |  |  |  |  |  |  |  |  |  |
| BMP7 |  |  |  |  |  |  |  |  |  |  |  |  |  |  |  |  |  |
| PEX13 |  |  |  |  |  |  |  |  |  |  |  |  |  |  |  |  |  |
| TNFAIP3 |  |  |  |  |  |  |  |  |  |  |  |  |  |  |  |  |  |
| UBE4B |  |  |  |  |  |  |  |  |  |  |  |  |  |  |  |  |  |
| KLHL40 |  |  |  |  |  |  |  |  |  |  |  |  |  |  |  |  |  |
| DCTN1 |  |  |  |  |  |  |  |  |  |  |  |  |  |  |  |  |  |
| ALPK3 |  |  |  |  |  |  |  |  |  |  |  |  |  |  |  |  |  |
| KIAA0753 |  |  |  |  |  |  |  |  |  |  |  |  |  |  |  |  |  |
| CHRNG |  |  |  |  |  |  |  |  |  |  |  |  |  |  |  |  |  |
| MEGF10 |  |  |  |  |  |  |  |  |  |  |  |  |  |  |  |  |  |
| KITLG |  |  |  |  |  |  |  |  |  |  |  |  |  |  |  |  |  |
| ABCB4 |  |  |  |  |  |  |  |  |  |  |  |  |  |  |  |  |  |
| PRDM9 |  |  |  |  |  |  |  |  |  |  |  |  |  |  |  |  |  |
| TRPM3 |  |  |  |  |  |  |  |  |  |  |  |  |  |  |  |  |  |
| SLC22A12 |  |  |  |  |  |  |  |  |  |  |  |  |  |  |  |  |  |
| CALM2 |  |  |  |  |  |  |  |  |  |  |  |  |  |  |  |  |  |
| DNHD1 |  |  |  |  |  |  |  |  |  |  |  |  |  |  |  |  |  |
| PEX16 |  |  |  |  |  |  |  |  |  |  |  |  |  |  |  |  |  |
| TCOF1 |  |  |  |  |  |  |  |  |  |  |  |  |  |  |  |  |  |
| CYP24A1 |  |  |  |  |  |  |  |  |  |  |  |  |  |  |  |  |  |
| PIK3C2G |  |  |  |  |  |  |  |  |  |  |  |  |  |  |  |  |  |
| RPL26 |  |  |  |  |  |  |  |  |  |  |  |  |  |  |  |  |  |
| CRKL |  |  |  |  |  |  |  |  |  |  |  |  |  |  |  |  |  |
| HNF1A |  |  |  |  |  |  |  |  |  |  |  |  |  |  |  |  |  |
| SPRED2 |  |  |  |  |  |  |  |  |  |  |  |  |  |  |  |  |  |
| EFTUD2 |  |  |  |  |  |  |  |  |  |  |  |  |  |  |  |  |  |
| IL7R |  |  |  |  |  |  |  |  |  |  |  |  |  |  |  |  |  |
| SMN1 |  |  |  |  |  |  |  |  |  |  |  |  |  |  |  |  |  |
| ADCY5 |  |  |  |  |  |  |  |  |  |  |  |  |  |  |  |  |  |
| DOCK6 |  |  |  |  |  |  |  |  |  |  |  |  |  |  |  |  |  |
| LEFTY2 |  |  |  |  |  |  |  |  |  |  |  |  |  |  |  |  |  |
| CFI |  |  |  |  |  |  |  |  |  |  |  |  |  |  |  |  |  |
| NOP10 |  |  |  |  |  |  |  |  |  |  |  |  |  |  |  |  |  |
| CLDN16 |  |  |  |  |  |  |  |  |  |  |  |  |  |  |  |  |  |
| POU5F1 |  |  |  |  |  |  |  |  |  |  |  |  |  |  |  |  |  |
| FOXE3 |  |  |  |  |  |  |  |  |  |  |  |  |  |  |  |  |  |
| SAA1 |  |  |  |  |  |  |  |  |  |  |  |  |  |  |  |  |  |
| CASP9 |  |  |  |  |  |  |  |  |  |  |  |  |  |  |  |  |  |
| MMP21 |  |  |  |  |  |  |  |  |  |  |  |  |  |  |  |  |  |
| KCNH1 |  |  |  |  |  |  |  |  |  |  |  |  |  |  |  |  |  |
| MT-TQ |  |  |  |  |  |  |  |  |  |  |  |  |  |  |  |  |  |
| FBXO43 |  |  |  |  |  |  |  |  |  |  |  |  |  |  |  |  |  |
| SLC25A3 |  |  |  |  |  |  |  |  |  |  |  |  |  |  |  |  |  |
| RGS5 |  |  |  |  |  |  |  |  |  |  |  |  |  |  |  |  |  |
| ANK1 |  |  |  |  |  |  |  |  |  |  |  |  |  |  |  |  |  |
| COX7B |  |  |  |  |  |  |  |  |  |  |  |  |  |  |  |  |  |
| CARD14 |  |  |  |  |  |  |  |  |  |  |  |  |  |  |  |  |  |
| PLCE1 |  |  |  |  |  |  |  |  |  |  |  |  |  |  |  |  |  |
| LRP5 |  |  |  |  |  |  |  |  |  |  |  |  |  |  |  |  |  |
| FGF21 |  |  |  |  |  |  |  |  |  |  |  |  |  |  |  |  |  |
| ATP6V0A2 |  |  |  |  |  |  |  |  |  |  |  |  |  |  |  |  |  |
| MAX |  |  |  |  |  |  |  |  |  |  |  |  |  |  |  |  |  |
| XYLT2 |  |  |  |  |  |  |  |  |  |  |  |  |  |  |  |  |  |
| APELA |  |  |  |  |  |  |  |  |  |  |  |  |  |  |  |  |  |
| LMX1B |  |  |  |  |  |  |  |  |  |  |  |  |  |  |  |  |  |
| SCNN1G |  |  |  |  |  |  |  |  |  |  |  |  |  |  |  |  |  |
| CYP27B1 |  |  |  |  |  |  |  |  |  |  |  |  |  |  |  |  |  |
| SDHAF1 |  |  |  |  |  |  |  |  |  |  |  |  |  |  |  |  |  |
| CCL11 |  |  |  |  |  |  |  |  |  |  |  |  |  |  |  |  |  |
| PTK2 |  |  |  |  |  |  |  |  |  |  |  |  |  |  |  |  |  |
| TCTN2 |  |  |  |  |  |  |  |  |  |  |  |  |  |  |  |  |  |
| TRIM21 |  |  |  |  |  |  |  |  |  |  |  |  |  |  |  |  |  |
| SMO |  |  |  |  |  |  |  |  |  |  |  |  |  |  |  |  |  |
| CASP8 |  |  |  |  |  |  |  |  |  |  |  |  |  |  |  |  |  |
| DSCAM |  |  |  |  |  |  |  |  |  |  |  |  |  |  |  |  |  |
| DAZL |  |  |  |  |  |  |  |  |  |  |  |  |  |  |  |  |  |
| TRPM6 |  |  |  |  |  |  |  |  |  |  |  |  |  |  |  |  |  |
| NDUFS8 |  |  |  |  |  |  |  |  |  |  |  |  |  |  |  |  |  |
| CD79A |  |  |  |  |  |  |  |  |  |  |  |  |  |  |  |  |  |
| RARB |  |  |  |  |  |  |  |  |  |  |  |  |  |  |  |  |  |
| GLE1 |  |  |  |  |  |  |  |  |  |  |  |  |  |  |  |  |  |
| GNE |  |  |  |  |  |  |  |  |  |  |  |  |  |  |  |  |  |
| PTF1A |  |  |  |  |  |  |  |  |  |  |  |  |  |  |  |  |  |
| LOC107133510 |  |  |  |  |  |  |  |  |  |  |  |  |  |  |  |  |  |
| CYP19A1 |  |  |  |  |  |  |  |  |  |  |  |  |  |  |  |  |  |
| UBE3B |  |  |  |  |  |  |  |  |  |  |  |  |  |  |  |  |  |
| HCFC1 |  |  |  |  |  |  |  |  |  |  |  |  |  |  |  |  |  |
| MYCN |  |  |  |  |  |  |  |  |  |  |  |  |  |  |  |  |  |
| UQCRB |  |  |  |  |  |  |  |  |  |  |  |  |  |  |  |  |  |
| DNAAF4 |  |  |  |  |  |  |  |  |  |  |  |  |  |  |  |  |  |
| MIR124-1 |  |  |  |  |  |  |  |  |  |  |  |  |  |  |  |  |  |
| PEX10 |  |  |  |  |  |  |  |  |  |  |  |  |  |  |  |  |  |
| BSND |  |  |  |  |  |  |  |  |  |  |  |  |  |  |  |  |  |
| TMEM231 |  |  |  |  |  |  |  |  |  |  |  |  |  |  |  |  |  |
| PEX12 |  |  |  |  |  |  |  |  |  |  |  |  |  |  |  |  |  |
| RAD21 |  |  |  |  |  |  |  |  |  |  |  |  |  |  |  |  |  |
| SPOP |  |  |  |  |  |  |  |  |  |  |  |  |  |  |  |  |  |
| GYG1 |  |  |  |  |  |  |  |  |  |  |  |  |  |  |  |  |  |
| TRPS1 |  |  |  |  |  |  |  |  |  |  |  |  |  |  |  |  |  |
| PUF60 |  |  |  |  |  |  |  |  |  |  |  |  |  |  |  |  |  |
| SLC2A4 |  |  |  |  |  |  |  |  |  |  |  |  |  |  |  |  |  |
| ANOS1 |  |  |  |  |  |  |  |  |  |  |  |  |  |  |  |  |  |
| CFHR5 |  |  |  |  |  |  |  |  |  |  |  |  |  |  |  |  |  |
| COX6A2 |  |  |  |  |  |  |  |  |  |  |  |  |  |  |  |  |  |
| FGD1 |  |  |  |  |  |  |  |  |  |  |  |  |  |  |  |  |  |
| CCDC141 |  |  |  |  |  |  |  |  |  |  |  |  |  |  |  |  |  |
| STN1 |  |  |  |  |  |  |  |  |  |  |  |  |  |  |  |  |  |
| SPECC1L |  |  |  |  |  |  |  |  |  |  |  |  |  |  |  |  |  |
| CCND1 |  |  |  |  |  |  |  |  |  |  |  |  |  |  |  |  |  |
| CLCN7 |  |  |  |  |  |  |  |  |  |  |  |  |  |  |  |  |  |
| B4GALT7 |  |  |  |  |  |  |  |  |  |  |  |  |  |  |  |  |  |
| PLAGL1 |  |  |  |  |  |  |  |  |  |  |  |  |  |  |  |  |  |
| MEIS2 |  |  |  |  |  |  |  |  |  |  |  |  |  |  |  |  |  |
| FCN3 |  |  |  |  |  |  |  |  |  |  |  |  |  |  |  |  |  |
| NDUFV2 |  |  |  |  |  |  |  |  |  |  |  |  |  |  |  |  |  |
| NEK8 |  |  |  |  |  |  |  |  |  |  |  |  |  |  |  |  |  |
| IL2RB |  |  |  |  |  |  |  |  |  |  |  |  |  |  |  |  |  |
| MESP1 |  |  |  |  |  |  |  |  |  |  |  |  |  |  |  |  |  |
| MT-TS2 |  |  |  |  |  |  |  |  |  |  |  |  |  |  |  |  |  |
| BCR |  |  |  |  |  |  |  |  |  |  |  |  |  |  |  |  |  |
| CSPP1 |  |  |  |  |  |  |  |  |  |  |  |  |  |  |  |  |  |
| AGL |  |  |  |  |  |  |  |  |  |  |  |  |  |  |  |  |  |
| MLYCD |  |  |  |  |  |  |  |  |  |  |  |  |  |  |  |  |  |
| GCLM |  |  |  |  |  |  |  |  |  |  |  |  |  |  |  |  |  |
| ACD |  |  |  |  |  |  |  |  |  |  |  |  |  |  |  |  |  |
| CR2 |  |  |  |  |  |  |  |  |  |  |  |  |  |  |  |  |  |
| ZPBP |  |  |  |  |  |  |  |  |  |  |  |  |  |  |  |  |  |
| PIGO |  |  |  |  |  |  |  |  |  |  |  |  |  |  |  |  |  |
| ANKRD11 |  |  |  |  |  |  |  |  |  |  |  |  |  |  |  |  |  |
| CYC1 |  |  |  |  |  |  |  |  |  |  |  |  |  |  |  |  |  |
| FLAD1 |  |  |  |  |  |  |  |  |  |  |  |  |  |  |  |  |  |
| HES7 |  |  |  |  |  |  |  |  |  |  |  |  |  |  |  |  |  |
| SLC2A9 |  |  |  |  |  |  |  |  |  |  |  |  |  |  |  |  |  |
| DNAJB11 |  |  |  |  |  |  |  |  |  |  |  |  |  |  |  |  |  |
| CDKN2A |  |  |  |  |  |  |  |  |  |  |  |  |  |  |  |  |  |
| KCNJ1 |  |  |  |  |  |  |  |  |  |  |  |  |  |  |  |  |  |
| LOC110121269 |  |  |  |  |  |  |  |  |  |  |  |  |  |  |  |  |  |
| PEX19 |  |  |  |  |  |  |  |  |  |  |  |  |  |  |  |  |  |
| NDUFB3 |  |  |  |  |  |  |  |  |  |  |  |  |  |  |  |  |  |
| PSMA6 |  |  |  |  |  |  |  |  |  |  |  |  |  |  |  |  |  |
| TOM1 |  |  |  |  |  |  |  |  |  |  |  |  |  |  |  |  |  |
| RPS20 |  |  |  |  |  |  |  |  |  |  |  |  |  |  |  |  |  |
| INTU |  |  |  |  |  |  |  |  |  |  |  |  |  |  |  |  |  |
| HNRNPK |  |  |  |  |  |  |  |  |  |  |  |  |  |  |  |  |  |
| COL5A2 |  |  |  |  |  |  |  |  |  |  |  |  |  |  |  |  |  |
| FOXJ1 |  |  |  |  |  |  |  |  |  |  |  |  |  |  |  |  |  |
| CEP164 |  |  |  |  |  |  |  |  |  |  |  |  |  |  |  |  |  |
| TMEM94 |  |  |  |  |  |  |  |  |  |  |  |  |  |  |  |  |  |
| CIITA |  |  |  |  |  |  |  |  |  |  |  |  |  |  |  |  |  |
| SETX |  |  |  |  |  |  |  |  |  |  |  |  |  |  |  |  |  |
| ACTN4 |  |  |  |  |  |  |  |  |  |  |  |  |  |  |  |  |  |
| SMARCE1 |  |  |  |  |  |  |  |  |  |  |  |  |  |  |  |  |  |
| HESX1 |  |  |  |  |  |  |  |  |  |  |  |  |  |  |  |  |  |
| KCND2 |  |  |  |  |  |  |  |  |  |  |  |  |  |  |  |  |  |
| MASP2 |  |  |  |  |  |  |  |  |  |  |  |  |  |  |  |  |  |
| HBG2 |  |  |  |  |  |  |  |  |  |  |  |  |  |  |  |  |  |
| BANF1 |  |  |  |  |  |  |  |  |  |  |  |  |  |  |  |  |  |
| ODAD3 |  |  |  |  |  |  |  |  |  |  |  |  |  |  |  |  |  |
| PHOX2B-AS1 |  |  |  |  |  |  |  |  |  |  |  |  |  |  |  |  |  |
| NDUFA11 |  |  |  |  |  |  |  |  |  |  |  |  |  |  |  |  |  |
| EFL1 |  |  |  |  |  |  |  |  |  |  |  |  |  |  |  |  |  |
| PEX14 |  |  |  |  |  |  |  |  |  |  |  |  |  |  |  |  |  |
| SPIDR |  |  |  |  |  |  |  |  |  |  |  |  |  |  |  |  |  |
| NEBL |  |  |  |  |  |  |  |  |  |  |  |  |  |  |  |  |  |
| SH3PXD2B |  |  |  |  |  |  |  |  |  |  |  |  |  |  |  |  |  |
| RPL15 |  |  |  |  |  |  |  |  |  |  |  |  |  |  |  |  |  |
| HYAL2 |  |  |  |  |  |  |  |  |  |  |  |  |  |  |  |  |  |
| COL5A1 |  |  |  |  |  |  |  |  |  |  |  |  |  |  |  |  |  |
| SNAP29 |  |  |  |  |  |  |  |  |  |  |  |  |  |  |  |  |  |
| ATXN2 |  |  |  |  |  |  |  |  |  |  |  |  |  |  |  |  |  |
| ADNP |  |  |  |  |  |  |  |  |  |  |  |  |  |  |  |  |  |
| HYMAI |  |  |  |  |  |  |  |  |  |  |  |  |  |  |  |  |  |
| IL12B |  |  |  |  |  |  |  |  |  |  |  |  |  |  |  |  |  |
| LMOD1 |  |  |  |  |  |  |  |  |  |  |  |  |  |  |  |  |  |
| GGT1 |  |  |  |  |  |  |  |  |  |  |  |  |  |  |  |  |  |
| NDUFS6 |  |  |  |  |  |  |  |  |  |  |  |  |  |  |  |  |  |
| MAP1B |  |  |  |  |  |  |  |  |  |  |  |  |  |  |  |  |  |
| FCGR3B |  |  |  |  |  |  |  |  |  |  |  |  |  |  |  |  |  |
| SLCO2A1 |  |  |  |  |  |  |  |  |  |  |  |  |  |  |  |  |  |
| PLAUR |  |  |  |  |  |  |  |  |  |  |  |  |  |  |  |  |  |
| LAMB2 |  |  |  |  |  |  |  |  |  |  |  |  |  |  |  |  |  |
| AUTS2 |  |  |  |  |  |  |  |  |  |  |  |  |  |  |  |  |  |
| TECRL |  |  |  |  |  |  |  |  |  |  |  |  |  |  |  |  |  |
| ALG8 |  |  |  |  |  |  |  |  |  |  |  |  |  |  |  |  |  |
| LETM1 |  |  |  |  |  |  |  |  |  |  |  |  |  |  |  |  |  |
| DNAJB13 |  |  |  |  |  |  |  |  |  |  |  |  |  |  |  |  |  |
| IFNB1 |  |  |  |  |  |  |  |  |  |  |  |  |  |  |  |  |  |
| TNFRSF11A |  |  |  |  |  |  |  |  |  |  |  |  |  |  |  |  |  |
| NDUFS3 |  |  |  |  |  |  |  |  |  |  |  |  |  |  |  |  |  |
| TCIRG1 |  |  |  |  |  |  |  |  |  |  |  |  |  |  |  |  |  |
| NELFA |  |  |  |  |  |  |  |  |  |  |  |  |  |  |  |  |  |
| SGCG |  |  |  |  |  |  |  |  |  |  |  |  |  |  |  |  |  |
| RAG1 |  |  |  |  |  |  |  |  |  |  |  |  |  |  |  |  |  |
| PCCA |  |  |  |  |  |  |  |  |  |  |  |  |  |  |  |  |  |
| LOC110011216 |  |  |  |  |  |  |  |  |  |  |  |  |  |  |  |  |  |
| RPS26 |  |  |  |  |  |  |  |  |  |  |  |  |  |  |  |  |  |
| RSPH4A |  |  |  |  |  |  |  |  |  |  |  |  |  |  |  |  |  |
| BAAT |  |  |  |  |  |  |  |  |  |  |  |  |  |  |  |  |  |
| TAF4B |  |  |  |  |  |  |  |  |  |  |  |  |  |  |  |  |  |
| C2CD6 |  |  |  |  |  |  |  |  |  |  |  |  |  |  |  |  |  |
| PQBP1 |  |  |  |  |  |  |  |  |  |  |  |  |  |  |  |  |  |
| GFM1 |  |  |  |  |  |  |  |  |  |  |  |  |  |  |  |  |  |
| LOC106099062 |  |  |  |  |  |  |  |  |  |  |  |  |  |  |  |  |  |
| PDCD1 |  |  |  |  |  |  |  |  |  |  |  |  |  |  |  |  |  |
| SLC2A2 |  |  |  |  |  |  |  |  |  |  |  |  |  |  |  |  |  |
| TG |  |  |  |  |  |  |  |  |  |  |  |  |  |  |  |  |  |
| C4B |  |  |  |  |  |  |  |  |  |  |  |  |  |  |  |  |  |
| NDUFB8 |  |  |  |  |  |  |  |  |  |  |  |  |  |  |  |  |  |
| MT-TH |  |  |  |  |  |  |  |  |  |  |  |  |  |  |  |  |  |
| B9D1 |  |  |  |  |  |  |  |  |  |  |  |  |  |  |  |  |  |
| DSE |  |  |  |  |  |  |  |  |  |  |  |  |  |  |  |  |  |
| HMGCL |  |  |  |  |  |  |  |  |  |  |  |  |  |  |  |  |  |
| EIF2AK3 |  |  |  |  |  |  |  |  |  |  |  |  |  |  |  |  |  |
| HPDL |  |  |  |  |  |  |  |  |  |  |  |  |  |  |  |  |  |
| DNAI2 |  |  |  |  |  |  |  |  |  |  |  |  |  |  |  |  |  |
| DHCR24 |  |  |  |  |  |  |  |  |  |  |  |  |  |  |  |  |  |
| MT-TG |  |  |  |  |  |  |  |  |  |  |  |  |  |  |  |  |  |
| LMBRD1 |  |  |  |  |  |  |  |  |  |  |  |  |  |  |  |  |  |
| NEK9 |  |  |  |  |  |  |  |  |  |  |  |  |  |  |  |  |  |
| RMRP |  |  |  |  |  |  |  |  |  |  |  |  |  |  |  |  |  |
| CYP21A2 |  |  |  |  |  |  |  |  |  |  |  |  |  |  |  |  |  |
| MAFB |  |  |  |  |  |  |  |  |  |  |  |  |  |  |  |  |  |
| PYGM |  |  |  |  |  |  |  |  |  |  |  |  |  |  |  |  |  |
| ZSWIM7 |  |  |  |  |  |  |  |  |  |  |  |  |  |  |  |  |  |
| PLD1 |  |  |  |  |  |  |  |  |  |  |  |  |  |  |  |  |  |
| OCRL |  |  |  |  |  |  |  |  |  |  |  |  |  |  |  |  |  |
| MIR590 |  |  |  |  |  |  |  |  |  |  |  |  |  |  |  |  |  |
| ATXN7 |  |  |  |  |  |  |  |  |  |  |  |  |  |  |  |  |  |
| SH2B1 |  |  |  |  |  |  |  |  |  |  |  |  |  |  |  |  |  |
| PRKCZ |  |  |  |  |  |  |  |  |  |  |  |  |  |  |  |  |  |
| KIF6 |  |  |  |  |  |  |  |  |  |  |  |  |  |  |  |  |  |
| GUCY1A1 |  |  |  |  |  |  |  |  |  |  |  |  |  |  |  |  |  |
| INHA |  |  |  |  |  |  |  |  |  |  |  |  |  |  |  |  |  |
| FUCA1 |  |  |  |  |  |  |  |  |  |  |  |  |  |  |  |  |  |
| MYOZ1 |  |  |  |  |  |  |  |  |  |  |  |  |  |  |  |  |  |
| CALCRL |  |  |  |  |  |  |  |  |  |  |  |  |  |  |  |  |  |
| IFT81 |  |  |  |  |  |  |  |  |  |  |  |  |  |  |  |  |  |
| NDUFA6 |  |  |  |  |  |  |  |  |  |  |  |  |  |  |  |  |  |
| UQCRC2 |  |  |  |  |  |  |  |  |  |  |  |  |  |  |  |  |  |
| TJP2 |  |  |  |  |  |  |  |  |  |  |  |  |  |  |  |  |  |
| XK |  |  |  |  |  |  |  |  |  |  |  |  |  |  |  |  |  |
| IRAK1 |  |  |  |  |  |  |  |  |  |  |  |  |  |  |  |  |  |
| ALOX5 |  |  |  |  |  |  |  |  |  |  |  |  |  |  |  |  |  |
| TTC26 |  |  |  |  |  |  |  |  |  |  |  |  |  |  |  |  |  |
| AHCY |  |  |  |  |  |  |  |  |  |  |  |  |  |  |  |  |  |
| RNF212 |  |  |  |  |  |  |  |  |  |  |  |  |  |  |  |  |  |
| ZNF469 |  |  |  |  |  |  |  |  |  |  |  |  |  |  |  |  |  |
| ABCD4 |  |  |  |  |  |  |  |  |  |  |  |  |  |  |  |  |  |
| TREX1 |  |  |  |  |  |  |  |  |  |  |  |  |  |  |  |  |  |
| VANGL1 |  |  |  |  |  |  |  |  |  |  |  |  |  |  |  |  |  |
| SGO2 |  |  |  |  |  |  |  |  |  |  |  |  |  |  |  |  |  |
| MESD |  |  |  |  |  |  |  |  |  |  |  |  |  |  |  |  |  |
| CHST14 |  |  |  |  |  |  |  |  |  |  |  |  |  |  |  |  |  |
| ALG1 |  |  |  |  |  |  |  |  |  |  |  |  |  |  |  |  |  |
| NDUFB9 |  |  |  |  |  |  |  |  |  |  |  |  |  |  |  |  |  |
| HSD3B7 |  |  |  |  |  |  |  |  |  |  |  |  |  |  |  |  |  |
| MT-TT |  |  |  |  |  |  |  |  |  |  |  |  |  |  |  |  |  |
| HNF4A |  |  |  |  |  |  |  |  |  |  |  |  |  |  |  |  |  |
| TRPM7 |  |  |  |  |  |  |  |  |  |  |  |  |  |  |  |  |  |
| SPRED1 |  |  |  |  |  |  |  |  |  |  |  |  |  |  |  |  |  |
| COL4A1 |  |  |  |  |  |  |  |  |  |  |  |  |  |  |  |  |  |
| KASH5 |  |  |  |  |  |  |  |  |  |  |  |  |  |  |  |  |  |
| AHDC1 |  |  |  |  |  |  |  |  |  |  |  |  |  |  |  |  |  |
| CD8A |  |  |  |  |  |  |  |  |  |  |  |  |  |  |  |  |  |
| TCN2 |  |  |  |  |  |  |  |  |  |  |  |  |  |  |  |  |  |
| TXNL4A |  |  |  |  |  |  |  |  |  |  |  |  |  |  |  |  |  |
| DRC1 |  |  |  |  |  |  |  |  |  |  |  |  |  |  |  |  |  |
| DPF2 |  |  |  |  |  |  |  |  |  |  |  |  |  |  |  |  |  |
| CDH23 |  |  |  |  |  |  |  |  |  |  |  |  |  |  |  |  |  |
| RUNX2 |  |  |  |  |  |  |  |  |  |  |  |  |  |  |  |  |  |
| ROR2 |  |  |  |  |  |  |  |  |  |  |  |  |  |  |  |  |  |
| ADAMTS10 |  |  |  |  |  |  |  |  |  |  |  |  |  |  |  |  |  |
| APOL1 |  |  |  |  |  |  |  |  |  |  |  |  |  |  |  |  |  |
| OTX2 |  |  |  |  |  |  |  |  |  |  |  |  |  |  |  |  |  |
| LRRC37A2 |  |  |  |  |  |  |  |  |  |  |  |  |  |  |  |  |  |
| VPS33B |  |  |  |  |  |  |  |  |  |  |  |  |  |  |  |  |  |
| HFE-AS1 |  |  |  |  |  |  |  |  |  |  |  |  |  |  |  |  |  |
| ZFP57 |  |  |  |  |  |  |  |  |  |  |  |  |  |  |  |  |  |
| VEGFC |  |  |  |  |  |  |  |  |  |  |  |  |  |  |  |  |  |
| NPC1L1 |  |  |  |  |  |  |  |  |  |  |  |  |  |  |  |  |  |
| PKD2L2-DT |  |  |  |  |  |  |  |  |  |  |  |  |  |  |  |  |  |
| MAPK10 |  |  |  |  |  |  |  |  |  |  |  |  |  |  |  |  |  |
| MMP23B |  |  |  |  |  |  |  |  |  |  |  |  |  |  |  |  |  |
| TYMP |  |  |  |  |  |  |  |  |  |  |  |  |  |  |  |  |  |
| STIL |  |  |  |  |  |  |  |  |  |  |  |  |  |  |  |  |  |
| ODAD4 |  |  |  |  |  |  |  |  |  |  |  |  |  |  |  |  |  |
| WNT4 |  |  |  |  |  |  |  |  |  |  |  |  |  |  |  |  |  |
| ESCO2 |  |  |  |  |  |  |  |  |  |  |  |  |  |  |  |  |  |
| IQCB1 |  |  |  |  |  |  |  |  |  |  |  |  |  |  |  |  |  |
| B9D2 |  |  |  |  |  |  |  |  |  |  |  |  |  |  |  |  |  |
| RELN |  |  |  |  |  |  |  |  |  |  |  |  |  |  |  |  |  |
| UBR1 |  |  |  |  |  |  |  |  |  |  |  |  |  |  |  |  |  |
| TAPVR1 |  |  |  |  |  |  |  |  |  |  |  |  |  |  |  |  |  |
| VIPAS39 |  |  |  |  |  |  |  |  |  |  |  |  |  |  |  |  |  |
| MIR1-2 |  |  |  |  |  |  |  |  |  |  |  |  |  |  |  |  |  |
| OXT |  |  |  |  |  |  |  |  |  |  |  |  |  |  |  |  |  |
| JMJD1C |  |  |  |  |  |  |  |  |  |  |  |  |  |  |  |  |  |
| VANGL2 |  |  |  |  |  |  |  |  |  |  |  |  |  |  |  |  |  |
| POPDC2 |  |  |  |  |  |  |  |  |  |  |  |  |  |  |  |  |  |
| MUC1 |  |  |  |  |  |  |  |  |  |  |  |  |  |  |  |  |  |
| LUZP1 |  |  |  |  |  |  |  |  |  |  |  |  |  |  |  |  |  |
| PCCB |  |  |  |  |  |  |  |  |  |  |  |  |  |  |  |  |  |
| SMARCC2 |  |  |  |  |  |  |  |  |  |  |  |  |  |  |  |  |  |
| RCAN1 |  |  |  |  |  |  |  |  |  |  |  |  |  |  |  |  |  |
| SDHC |  |  |  |  |  |  |  |  |  |  |  |  |  |  |  |  |  |
| DVL1 |  |  |  |  |  |  |  |  |  |  |  |  |  |  |  |  |  |
| GOPC |  |  |  |  |  |  |  |  |  |  |  |  |  |  |  |  |  |
| COA3 |  |  |  |  |  |  |  |  |  |  |  |  |  |  |  |  |  |
| PEX11B |  |  |  |  |  |  |  |  |  |  |  |  |  |  |  |  |  |
| COA8 |  |  |  |  |  |  |  |  |  |  |  |  |  |  |  |  |  |
| CCDC62 |  |  |  |  |  |  |  |  |  |  |  |  |  |  |  |  |  |
| ATP7A |  |  |  |  |  |  |  |  |  |  |  |  |  |  |  |  |  |
| CAMK2G |  |  |  |  |  |  |  |  |  |  |  |  |  |  |  |  |  |
| POGZ |  |  |  |  |  |  |  |  |  |  |  |  |  |  |  |  |  |
| SELL |  |  |  |  |  |  |  |  |  |  |  |  |  |  |  |  |  |
| CHD4 |  |  |  |  |  |  |  |  |  |  |  |  |  |  |  |  |  |
| EDN2 |  |  |  |  |  |  |  |  |  |  |  |  |  |  |  |  |  |
| TACR3 |  |  |  |  |  |  |  |  |  |  |  |  |  |  |  |  |  |
| VAC14 |  |  |  |  |  |  |  |  |  |  |  |  |  |  |  |  |  |
| LMNB1 |  |  |  |  |  |  |  |  |  |  |  |  |  |  |  |  |  |
| RECQL4 |  |  |  |  |  |  |  |  |  |  |  |  |  |  |  |  |  |
| SMARCD1 |  |  |  |  |  |  |  |  |  |  |  |  |  |  |  |  |  |
| KYNU |  |  |  |  |  |  |  |  |  |  |  |  |  |  |  |  |  |
| PDLIM5 |  |  |  |  |  |  |  |  |  |  |  |  |  |  |  |  |  |
| MSX2 |  |  |  |  |  |  |  |  |  |  |  |  |  |  |  |  |  |
| OBSCN |  |  |  |  |  |  |  |  |  |  |  |  |  |  |  |  |  |
| ALG12 |  |  |  |  |  |  |  |  |  |  |  |  |  |  |  |  |  |
| MYH4 |  |  |  |  |  |  |  |  |  |  |  |  |  |  |  |  |  |
| SRP54 |  |  |  |  |  |  |  |  |  |  |  |  |  |  |  |  |  |
| ABCD1 |  |  |  |  |  |  |  |  |  |  |  |  |  |  |  |  |  |
| BMPR1B |  |  |  |  |  |  |  |  |  |  |  |  |  |  |  |  |  |
| PGR |  |  |  |  |  |  |  |  |  |  |  |  |  |  |  |  |  |
| COG1 |  |  |  |  |  |  |  |  |  |  |  |  |  |  |  |  |  |
| FUZ |  |  |  |  |  |  |  |  |  |  |  |  |  |  |  |  |  |
| KDM4C |  |  |  |  |  |  |  |  |  |  |  |  |  |  |  |  |  |
| B3GLCT |  |  |  |  |  |  |  |  |  |  |  |  |  |  |  |  |  |
| IMMT |  |  |  |  |  |  |  |  |  |  |  |  |  |  |  |  |  |
| CASK |  |  |  |  |  |  |  |  |  |  |  |  |  |  |  |  |  |
| AMN |  |  |  |  |  |  |  |  |  |  |  |  |  |  |  |  |  |
| TERB2 |  |  |  |  |  |  |  |  |  |  |  |  |  |  |  |  |  |
| NEUROD1 |  |  |  |  |  |  |  |  |  |  |  |  |  |  |  |  |  |
| PNPLA8 |  |  |  |  |  |  |  |  |  |  |  |  |  |  |  |  |  |
| BLM |  |  |  |  |  |  |  |  |  |  |  |  |  |  |  |  |  |
| RPL35A |  |  |  |  |  |  |  |  |  |  |  |  |  |  |  |  |  |
| TMPRSS15 |  |  |  |  |  |  |  |  |  |  |  |  |  |  |  |  |  |
| KCNQ1-AS1 |  |  |  |  |  |  |  |  |  |  |  |  |  |  |  |  |  |
| ARL6 |  |  |  |  |  |  |  |  |  |  |  |  |  |  |  |  |  |
| COQ4 |  |  |  |  |  |  |  |  |  |  |  |  |  |  |  |  |  |
| GPC4 |  |  |  |  |  |  |  |  |  |  |  |  |  |  |  |  |  |
| CPLANE1 |  |  |  |  |  |  |  |  |  |  |  |  |  |  |  |  |  |
| NPM1 |  |  |  |  |  |  |  |  |  |  |  |  |  |  |  |  |  |
| LOC110006319 |  |  |  |  |  |  |  |  |  |  |  |  |  |  |  |  |  |
| GNRHR |  |  |  |  |  |  |  |  |  |  |  |  |  |  |  |  |  |
| THBS4 |  |  |  |  |  |  |  |  |  |  |  |  |  |  |  |  |  |
| PDGFB |  |  |  |  |  |  |  |  |  |  |  |  |  |  |  |  |  |
| TRAF3IP1 |  |  |  |  |  |  |  |  |  |  |  |  |  |  |  |  |  |
| LEMD2 |  |  |  |  |  |  |  |  |  |  |  |  |  |  |  |  |  |
| TMEM127 |  |  |  |  |  |  |  |  |  |  |  |  |  |  |  |  |  |
| PRKACA |  |  |  |  |  |  |  |  |  |  |  |  |  |  |  |  |  |
| CFHR1 |  |  |  |  |  |  |  |  |  |  |  |  |  |  |  |  |  |
| RANGRF |  |  |  |  |  |  |  |  |  |  |  |  |  |  |  |  |  |
| IBA57 |  |  |  |  |  |  |  |  |  |  |  |  |  |  |  |  |  |
| DDC |  |  |  |  |  |  |  |  |  |  |  |  |  |  |  |  |  |
| INPPL1 |  |  |  |  |  |  |  |  |  |  |  |  |  |  |  |  |  |
| RSPH3 |  |  |  |  |  |  |  |  |  |  |  |  |  |  |  |  |  |
| LOC119369037 |  |  |  |  |  |  |  |  |  |  |  |  |  |  |  |  |  |
| CRYAA |  |  |  |  |  |  |  |  |  |  |  |  |  |  |  |  |  |
| ROBO4 |  |  |  |  |  |  |  |  |  |  |  |  |  |  |  |  |  |
| COG7 |  |  |  |  |  |  |  |  |  |  |  |  |  |  |  |  |  |
| CHCHD10 |  |  |  |  |  |  |  |  |  |  |  |  |  |  |  |  |  |
| PRR12 |  |  |  |  |  |  |  |  |  |  |  |  |  |  |  |  |  |
| HPGD |  |  |  |  |  |  |  |  |  |  |  |  |  |  |  |  |  |
| KIF20A |  |  |  |  |  |  |  |  |  |  |  |  |  |  |  |  |  |
| PDHA1 |  |  |  |  |  |  |  |  |  |  |  |  |  |  |  |  |  |
| LOC113939944 |  |  |  |  |  |  |  |  |  |  |  |  |  |  |  |  |  |
| SDHAF2 |  |  |  |  |  |  |  |  |  |  |  |  |  |  |  |  |  |
| SLC4A4 |  |  |  |  |  |  |  |  |  |  |  |  |  |  |  |  |  |
| CHRM3 |  |  |  |  |  |  |  |  |  |  |  |  |  |  |  |  |  |
| EPOR |  |  |  |  |  |  |  |  |  |  |  |  |  |  |  |  |  |
| IL15 |  |  |  |  |  |  |  |  |  |  |  |  |  |  |  |  |  |
| FOXP2 |  |  |  |  |  |  |  |  |  |  |  |  |  |  |  |  |  |
| SAR1B |  |  |  |  |  |  |  |  |  |  |  |  |  |  |  |  |  |
| SERAC1 |  |  |  |  |  |  |  |  |  |  |  |  |  |  |  |  |  |
| CELF2 |  |  |  |  |  |  |  |  |  |  |  |  |  |  |  |  |  |
| LOC113687175 |  |  |  |  |  |  |  |  |  |  |  |  |  |  |  |  |  |
| ALK |  |  |  |  |  |  |  |  |  |  |  |  |  |  |  |  |  |
| NFS1 |  |  |  |  |  |  |  |  |  |  |  |  |  |  |  |  |  |
| TRMT10C |  |  |  |  |  |  |  |  |  |  |  |  |  |  |  |  |  |
| MAGEL2 |  |  |  |  |  |  |  |  |  |  |  |  |  |  |  |  |  |
| ADK |  |  |  |  |  |  |  |  |  |  |  |  |  |  |  |  |  |
| CPLX1 |  |  |  |  |  |  |  |  |  |  |  |  |  |  |  |  |  |
| SRY |  |  |  |  |  |  |  |  |  |  |  |  |  |  |  |  |  |
| TMCO1 |  |  |  |  |  |  |  |  |  |  |  |  |  |  |  |  |  |
| APOA4 |  |  |  |  |  |  |  |  |  |  |  |  |  |  |  |  |  |
| PIGV |  |  |  |  |  |  |  |  |  |  |  |  |  |  |  |  |  |
| CASQ1 |  |  |  |  |  |  |  |  |  |  |  |  |  |  |  |  |  |
| BCKDHB |  |  |  |  |  |  |  |  |  |  |  |  |  |  |  |  |  |
| CCDC103 |  |  |  |  |  |  |  |  |  |  |  |  |  |  |  |  |  |
| ODAD1 |  |  |  |  |  |  |  |  |  |  |  |  |  |  |  |  |  |
| RSPO2 |  |  |  |  |  |  |  |  |  |  |  |  |  |  |  |  |  |
| GABRD |  |  |  |  |  |  |  |  |  |  |  |  |  |  |  |  |  |
| NDUFC2 |  |  |  |  |  |  |  |  |  |  |  |  |  |  |  |  |  |
| ARFGEF2 |  |  |  |  |  |  |  |  |  |  |  |  |  |  |  |  |  |
| NDUFAF4 |  |  |  |  |  |  |  |  |  |  |  |  |  |  |  |  |  |
| CCT7 |  |  |  |  |  |  |  |  |  |  |  |  |  |  |  |  |  |
| HPRT1 |  |  |  |  |  |  |  |  |  |  |  |  |  |  |  |  |  |
| FDFT1 |  |  |  |  |  |  |  |  |  |  |  |  |  |  |  |  |  |
| WASHC5 |  |  |  |  |  |  |  |  |  |  |  |  |  |  |  |  |  |
| YARS2 |  |  |  |  |  |  |  |  |  |  |  |  |  |  |  |  |  |
| SOX18 |  |  |  |  |  |  |  |  |  |  |  |  |  |  |  |  |  |
| BAG5 |  |  |  |  |  |  |  |  |  |  |  |  |  |  |  |  |  |
| IQSEC2 |  |  |  |  |  |  |  |  |  |  |  |  |  |  |  |  |  |
| TSR2 |  |  |  |  |  |  |  |  |  |  |  |  |  |  |  |  |  |
| TGDS |  |  |  |  |  |  |  |  |  |  |  |  |  |  |  |  |  |
| SYNE1 |  |  |  |  |  |  |  |  |  |  |  |  |  |  |  |  |  |
| RAD54L |  |  |  |  |  |  |  |  |  |  |  |  |  |  |  |  |  |
| MYBPC1 |  |  |  |  |  |  |  |  |  |  |  |  |  |  |  |  |  |
| NUP107 |  |  |  |  |  |  |  |  |  |  |  |  |  |  |  |  |  |
| FGF1 |  |  |  |  |  |  |  |  |  |  |  |  |  |  |  |  |  |
| EPM2A |  |  |  |  |  |  |  |  |  |  |  |  |  |  |  |  |  |
| CYP27A1 |  |  |  |  |  |  |  |  |  |  |  |  |  |  |  |  |  |
| PGM1 |  |  |  |  |  |  |  |  |  |  |  |  |  |  |  |  |  |
| RPS17 |  |  |  |  |  |  |  |  |  |  |  |  |  |  |  |  |  |
| ATRIP |  |  |  |  |  |  |  |  |  |  |  |  |  |  |  |  |  |
| AKT3 |  |  |  |  |  |  |  |  |  |  |  |  |  |  |  |  |  |
| SPAG1 |  |  |  |  |  |  |  |  |  |  |  |  |  |  |  |  |  |
| DHFR |  |  |  |  |  |  |  |  |  |  |  |  |  |  |  |  |  |
| ATP6V1A |  |  |  |  |  |  |  |  |  |  |  |  |  |  |  |  |  |
| NAGA |  |  |  |  |  |  |  |  |  |  |  |  |  |  |  |  |  |
| WWOX |  |  |  |  |  |  |  |  |  |  |  |  |  |  |  |  |  |
| ERCC5 |  |  |  |  |  |  |  |  |  |  |  |  |  |  |  |  |  |
| PPCS |  |  |  |  |  |  |  |  |  |  |  |  |  |  |  |  |  |
| MICOS13 |  |  |  |  |  |  |  |  |  |  |  |  |  |  |  |  |  |
| GAS2L2 |  |  |  |  |  |  |  |  |  |  |  |  |  |  |  |  |  |
| FECH |  |  |  |  |  |  |  |  |  |  |  |  |  |  |  |  |  |
| SLC7A7 |  |  |  |  |  |  |  |  |  |  |  |  |  |  |  |  |  |
| TCTN1 |  |  |  |  |  |  |  |  |  |  |  |  |  |  |  |  |  |
| JAK3 |  |  |  |  |  |  |  |  |  |  |  |  |  |  |  |  |  |
| MMAA |  |  |  |  |  |  |  |  |  |  |  |  |  |  |  |  |  |
| C12orf60 |  |  |  |  |  |  |  |  |  |  |  |  |  |  |  |  |  |
| DDX3Y |  |  |  |  |  |  |  |  |  |  |  |  |  |  |  |  |  |
| PSMD12 |  |  |  |  |  |  |  |  |  |  |  |  |  |  |  |  |  |
| HS6ST1 |  |  |  |  |  |  |  |  |  |  |  |  |  |  |  |  |  |
| ARX |  |  |  |  |  |  |  |  |  |  |  |  |  |  |  |  |  |
| SYNE2 |  |  |  |  |  |  |  |  |  |  |  |  |  |  |  |  |  |
| RTL1 |  |  |  |  |  |  |  |  |  |  |  |  |  |  |  |  |  |
| PLA2G6 |  |  |  |  |  |  |  |  |  |  |  |  |  |  |  |  |  |
| TARDBP |  |  |  |  |  |  |  |  |  |  |  |  |  |  |  |  |  |
| CFAP298 |  |  |  |  |  |  |  |  |  |  |  |  |  |  |  |  |  |
| FAN1 |  |  |  |  |  |  |  |  |  |  |  |  |  |  |  |  |  |
| GDF6 |  |  |  |  |  |  |  |  |  |  |  |  |  |  |  |  |  |
| KCNMA1 |  |  |  |  |  |  |  |  |  |  |  |  |  |  |  |  |  |
| FAT4 |  |  |  |  |  |  |  |  |  |  |  |  |  |  |  |  |  |
| CMH21 |  |  |  |  |  |  |  |  |  |  |  |  |  |  |  |  |  |
| POLR3A |  |  |  |  |  |  |  |  |  |  |  |  |  |  |  |  |  |
| CHRNE |  |  |  |  |  |  |  |  |  |  |  |  |  |  |  |  |  |
| SMCHD1 |  |  |  |  |  |  |  |  |  |  |  |  |  |  |  |  |  |
| IARS2 |  |  |  |  |  |  |  |  |  |  |  |  |  |  |  |  |  |
| TNNI1 |  |  |  |  |  |  |  |  |  |  |  |  |  |  |  |  |  |
| REC8 |  |  |  |  |  |  |  |  |  |  |  |  |  |  |  |  |  |
| NSMCE2 |  |  |  |  |  |  |  |  |  |  |  |  |  |  |  |  |  |
| CTSA |  |  |  |  |  |  |  |  |  |  |  |  |  |  |  |  |  |
| DDX11 |  |  |  |  |  |  |  |  |  |  |  |  |  |  |  |  |  |
| HOXD13 |  |  |  |  |  |  |  |  |  |  |  |  |  |  |  |  |  |
| OSTM1 |  |  |  |  |  |  |  |  |  |  |  |  |  |  |  |  |  |
| PGAP3 |  |  |  |  |  |  |  |  |  |  |  |  |  |  |  |  |  |
| MLX |  |  |  |  |  |  |  |  |  |  |  |  |  |  |  |  |  |
| P2RY12 |  |  |  |  |  |  |  |  |  |  |  |  |  |  |  |  |  |
| CCDC22 |  |  |  |  |  |  |  |  |  |  |  |  |  |  |  |  |  |
| HSPA8 |  |  |  |  |  |  |  |  |  |  |  |  |  |  |  |  |  |
| MYOG |  |  |  |  |  |  |  |  |  |  |  |  |  |  |  |  |  |
| TIMMDC1 |  |  |  |  |  |  |  |  |  |  |  |  |  |  |  |  |  |
| MFAP5 |  |  |  |  |  |  |  |  |  |  |  |  |  |  |  |  |  |
| RAG2 |  |  |  |  |  |  |  |  |  |  |  |  |  |  |  |  |  |
| ERCC3 |  |  |  |  |  |  |  |  |  |  |  |  |  |  |  |  |  |
| VPS13B |  |  |  |  |  |  |  |  |  |  |  |  |  |  |  |  |  |
| COX16 |  |  |  |  |  |  |  |  |  |  |  |  |  |  |  |  |  |
| NDUFAF8 |  |  |  |  |  |  |  |  |  |  |  |  |  |  |  |  |  |
| U2AF1 |  |  |  |  |  |  |  |  |  |  |  |  |  |  |  |  |  |
| ZNF462 |  |  |  |  |  |  |  |  |  |  |  |  |  |  |  |  |  |
| FABP1 |  |  |  |  |  |  |  |  |  |  |  |  |  |  |  |  |  |
| SF3B4 |  |  |  |  |  |  |  |  |  |  |  |  |  |  |  |  |  |
| COX4I2 |  |  |  |  |  |  |  |  |  |  |  |  |  |  |  |  |  |
| ASCC1 |  |  |  |  |  |  |  |  |  |  |  |  |  |  |  |  |  |
| HGD |  |  |  |  |  |  |  |  |  |  |  |  |  |  |  |  |  |
| PSMB8 |  |  |  |  |  |  |  |  |  |  |  |  |  |  |  |  |  |
| APOA1-AS |  |  |  |  |  |  |  |  |  |  |  |  |  |  |  |  |  |
| MRAP |  |  |  |  |  |  |  |  |  |  |  |  |  |  |  |  |  |
| CUL3 |  |  |  |  |  |  |  |  |  |  |  |  |  |  |  |  |  |
| ADH5 |  |  |  |  |  |  |  |  |  |  |  |  |  |  |  |  |  |
| GAREM2 |  |  |  |  |  |  |  |  |  |  |  |  |  |  |  |  |  |
| MRPS34 |  |  |  |  |  |  |  |  |  |  |  |  |  |  |  |  |  |
| DLAT |  |  |  |  |  |  |  |  |  |  |  |  |  |  |  |  |  |
| RPGRIP1 |  |  |  |  |  |  |  |  |  |  |  |  |  |  |  |  |  |
| ALG2 |  |  |  |  |  |  |  |  |  |  |  |  |  |  |  |  |  |
| DYNLT2B |  |  |  |  |  |  |  |  |  |  |  |  |  |  |  |  |  |
| GSTM1 |  |  |  |  |  |  |  |  |  |  |  |  |  |  |  |  |  |
| COQ9 |  |  |  |  |  |  |  |  |  |  |  |  |  |  |  |  |  |
| OTC |  |  |  |  |  |  |  |  |  |  |  |  |  |  |  |  |  |
| POLA1 |  |  |  |  |  |  |  |  |  |  |  |  |  |  |  |  |  |
| YWHAG |  |  |  |  |  |  |  |  |  |  |  |  |  |  |  |  |  |
| UBE3A |  |  |  |  |  |  |  |  |  |  |  |  |  |  |  |  |  |
| GAS8 |  |  |  |  |  |  |  |  |  |  |  |  |  |  |  |  |  |
| IKZF1 |  |  |  |  |  |  |  |  |  |  |  |  |  |  |  |  |  |
| GRIP1 |  |  |  |  |  |  |  |  |  |  |  |  |  |  |  |  |  |
| PIBF1 |  |  |  |  |  |  |  |  |  |  |  |  |  |  |  |  |  |
| TPO |  |  |  |  |  |  |  |  |  |  |  |  |  |  |  |  |  |
| USP9X |  |  |  |  |  |  |  |  |  |  |  |  |  |  |  |  |  |
| DCDC2 |  |  |  |  |  |  |  |  |  |  |  |  |  |  |  |  |  |
| SLC3A1 |  |  |  |  |  |  |  |  |  |  |  |  |  |  |  |  |  |
| NPC2 |  |  |  |  |  |  |  |  |  |  |  |  |  |  |  |  |  |
| GLUL |  |  |  |  |  |  |  |  |  |  |  |  |  |  |  |  |  |
| ZMIZ1 |  |  |  |  |  |  |  |  |  |  |  |  |  |  |  |  |  |
| HIBCH |  |  |  |  |  |  |  |  |  |  |  |  |  |  |  |  |  |
| ABCC2 |  |  |  |  |  |  |  |  |  |  |  |  |  |  |  |  |  |
| EMG1 |  |  |  |  |  |  |  |  |  |  |  |  |  |  |  |  |  |
| BTNL2 |  |  |  |  |  |  |  |  |  |  |  |  |  |  |  |  |  |
| LRRC10 |  |  |  |  |  |  |  |  |  |  |  |  |  |  |  |  |  |
| MESP2 |  |  |  |  |  |  |  |  |  |  |  |  |  |  |  |  |  |
| CFAP92 |  |  |  |  |  |  |  |  |  |  |  |  |  |  |  |  |  |
| CHD3 |  |  |  |  |  |  |  |  |  |  |  |  |  |  |  |  |  |
| RSPH9 |  |  |  |  |  |  |  |  |  |  |  |  |  |  |  |  |  |
| PNPLA6 |  |  |  |  |  |  |  |  |  |  |  |  |  |  |  |  |  |
| ATAD1 |  |  |  |  |  |  |  |  |  |  |  |  |  |  |  |  |  |
| POMT1 |  |  |  |  |  |  |  |  |  |  |  |  |  |  |  |  |  |
| SCGB1A1 |  |  |  |  |  |  |  |  |  |  |  |  |  |  |  |  |  |
| AKAP13 |  |  |  |  |  |  |  |  |  |  |  |  |  |  |  |  |  |
| PCNT |  |  |  |  |  |  |  |  |  |  |  |  |  |  |  |  |  |
| LAMA3 |  |  |  |  |  |  |  |  |  |  |  |  |  |  |  |  |  |
| SMN2 |  |  |  |  |  |  |  |  |  |  |  |  |  |  |  |  |  |
| TXNDC15 |  |  |  |  |  |  |  |  |  |  |  |  |  |  |  |  |  |
| NALCN |  |  |  |  |  |  |  |  |  |  |  |  |  |  |  |  |  |
| ANKS6 |  |  |  |  |  |  |  |  |  |  |  |  |  |  |  |  |  |
| SI |  |  |  |  |  |  |  |  |  |  |  |  |  |  |  |  |  |
| AFG3L2 |  |  |  |  |  |  |  |  |  |  |  |  |  |  |  |  |  |
| TTC12 |  |  |  |  |  |  |  |  |  |  |  |  |  |  |  |  |  |
| CFAP300 |  |  |  |  |  |  |  |  |  |  |  |  |  |  |  |  |  |
| IFNA2 |  |  |  |  |  |  |  |  |  |  |  |  |  |  |  |  |  |
| ACAD8 |  |  |  |  |  |  |  |  |  |  |  |  |  |  |  |  |  |
| HLA-G |  |  |  |  |  |  |  |  |  |  |  |  |  |  |  |  |  |
| PROK2 |  |  |  |  |  |  |  |  |  |  |  |  |  |  |  |  |  |
| MRPL44 |  |  |  |  |  |  |  |  |  |  |  |  |  |  |  |  |  |
| NDUFB10 |  |  |  |  |  |  |  |  |  |  |  |  |  |  |  |  |  |
| DNAL1 |  |  |  |  |  |  |  |  |  |  |  |  |  |  |  |  |  |
| COG4 |  |  |  |  |  |  |  |  |  |  |  |  |  |  |  |  |  |
| PHEX |  |  |  |  |  |  |  |  |  |  |  |  |  |  |  |  |  |
| RAB23 |  |  |  |  |  |  |  |  |  |  |  |  |  |  |  |  |  |
| ECHS1 |  |  |  |  |  |  |  |  |  |  |  |  |  |  |  |  |  |
| GALNT11 |  |  |  |  |  |  |  |  |  |  |  |  |  |  |  |  |  |
| C2CD3 |  |  |  |  |  |  |  |  |  |  |  |  |  |  |  |  |  |
| PI4KA |  |  |  |  |  |  |  |  |  |  |  |  |  |  |  |  |  |
| POR |  |  |  |  |  |  |  |  |  |  |  |  |  |  |  |  |  |
| UQCRQ |  |  |  |  |  |  |  |  |  |  |  |  |  |  |  |  |  |
| CCBE1 |  |  |  |  |  |  |  |  |  |  |  |  |  |  |  |  |  |
| IRX5 |  |  |  |  |  |  |  |  |  |  |  |  |  |  |  |  |  |
| IFT52 |  |  |  |  |  |  |  |  |  |  |  |  |  |  |  |  |  |
| HSPB2 |  |  |  |  |  |  |  |  |  |  |  |  |  |  |  |  |  |
| APC |  |  |  |  |  |  |  |  |  |  |  |  |  |  |  |  |  |
| PRKCE |  |  |  |  |  |  |  |  |  |  |  |  |  |  |  |  |  |
| EOGT |  |  |  |  |  |  |  |  |  |  |  |  |  |  |  |  |  |
| TNFRSF13B |  |  |  |  |  |  |  |  |  |  |  |  |  |  |  |  |  |
| DKK1 |  |  |  |  |  |  |  |  |  |  |  |  |  |  |  |  |  |
| PDHX |  |  |  |  |  |  |  |  |  |  |  |  |  |  |  |  |  |
| SRCAP |  |  |  |  |  |  |  |  |  |  |  |  |  |  |  |  |  |
| TRMT5 |  |  |  |  |  |  |  |  |  |  |  |  |  |  |  |  |  |
| FLRT3 |  |  |  |  |  |  |  |  |  |  |  |  |  |  |  |  |  |
| PLK4 |  |  |  |  |  |  |  |  |  |  |  |  |  |  |  |  |  |
| DOK7 |  |  |  |  |  |  |  |  |  |  |  |  |  |  |  |  |  |
| POLR1A |  |  |  |  |  |  |  |  |  |  |  |  |  |  |  |  |  |
| COG6 |  |  |  |  |  |  |  |  |  |  |  |  |  |  |  |  |  |
| CFHR3 |  |  |  |  |  |  |  |  |  |  |  |  |  |  |  |  |  |
| BICRA |  |  |  |  |  |  |  |  |  |  |  |  |  |  |  |  |  |
| GCDH |  |  |  |  |  |  |  |  |  |  |  |  |  |  |  |  |  |
| TTC7A |  |  |  |  |  |  |  |  |  |  |  |  |  |  |  |  |  |
| DZIP1L |  |  |  |  |  |  |  |  |  |  |  |  |  |  |  |  |  |
| CEP57 |  |  |  |  |  |  |  |  |  |  |  |  |  |  |  |  |  |
| SLC26A1 |  |  |  |  |  |  |  |  |  |  |  |  |  |  |  |  |  |
| NARS2 |  |  |  |  |  |  |  |  |  |  |  |  |  |  |  |  |  |
| ACVR1B |  |  |  |  |  |  |  |  |  |  |  |  |  |  |  |  |  |
| SEMA5A |  |  |  |  |  |  |  |  |  |  |  |  |  |  |  |  |  |
| PLEKHM2 |  |  |  |  |  |  |  |  |  |  |  |  |  |  |  |  |  |
| LPIN1 |  |  |  |  |  |  |  |  |  |  |  |  |  |  |  |  |  |
| ITGA8 |  |  |  |  |  |  |  |  |  |  |  |  |  |  |  |  |  |
| GNB1 |  |  |  |  |  |  |  |  |  |  |  |  |  |  |  |  |  |
| MRPL3 |  |  |  |  |  |  |  |  |  |  |  |  |  |  |  |  |  |
| HEXB |  |  |  |  |  |  |  |  |  |  |  |  |  |  |  |  |  |
| MT-TL2 |  |  |  |  |  |  |  |  |  |  |  |  |  |  |  |  |  |
| TDRKH |  |  |  |  |  |  |  |  |  |  |  |  |  |  |  |  |  |
| COQ8B |  |  |  |  |  |  |  |  |  |  |  |  |  |  |  |  |  |
| ZNF276 |  |  |  |  |  |  |  |  |  |  |  |  |  |  |  |  |  |
| CFAP418 |  |  |  |  |  |  |  |  |  |  |  |  |  |  |  |  |  |
| EPCAM |  |  |  |  |  |  |  |  |  |  |  |  |  |  |  |  |  |
| FGF12 |  |  |  |  |  |  |  |  |  |  |  |  |  |  |  |  |  |
| AFF2 |  |  |  |  |  |  |  |  |  |  |  |  |  |  |  |  |  |
| DNAAF5 |  |  |  |  |  |  |  |  |  |  |  |  |  |  |  |  |  |
| CCDC115 |  |  |  |  |  |  |  |  |  |  |  |  |  |  |  |  |  |
| SPTA1 |  |  |  |  |  |  |  |  |  |  |  |  |  |  |  |  |  |
| PPP2R2B |  |  |  |  |  |  |  |  |  |  |  |  |  |  |  |  |  |
| CHKB |  |  |  |  |  |  |  |  |  |  |  |  |  |  |  |  |  |
| RSPH1 |  |  |  |  |  |  |  |  |  |  |  |  |  |  |  |  |  |
| AUH |  |  |  |  |  |  |  |  |  |  |  |  |  |  |  |  |  |
| ATP6AP1 |  |  |  |  |  |  |  |  |  |  |  |  |  |  |  |  |  |
| CDKN3 |  |  |  |  |  |  |  |  |  |  |  |  |  |  |  |  |  |
| EZH2 |  |  |  |  |  |  |  |  |  |  |  |  |  |  |  |  |  |
| LOC110121486 |  |  |  |  |  |  |  |  |  |  |  |  |  |  |  |  |  |
| MYOM1 |  |  |  |  |  |  |  |  |  |  |  |  |  |  |  |  |  |
| LOC106627981 |  |  |  |  |  |  |  |  |  |  |  |  |  |  |  |  |  |
| PRICKLE1 |  |  |  |  |  |  |  |  |  |  |  |  |  |  |  |  |  |
| EBP |  |  |  |  |  |  |  |  |  |  |  |  |  |  |  |  |  |
| TMPRSS2 |  |  |  |  |  |  |  |  |  |  |  |  |  |  |  |  |  |
| SLC34A2 |  |  |  |  |  |  |  |  |  |  |  |  |  |  |  |  |  |
| L1CAM |  |  |  |  |  |  |  |  |  |  |  |  |  |  |  |  |  |
| FBLN2 |  |  |  |  |  |  |  |  |  |  |  |  |  |  |  |  |  |
| DUSP6 |  |  |  |  |  |  |  |  |  |  |  |  |  |  |  |  |  |
| KCNJ6 |  |  |  |  |  |  |  |  |  |  |  |  |  |  |  |  |  |
| SCN2A |  |  |  |  |  |  |  |  |  |  |  |  |  |  |  |  |  |
| NME8 |  |  |  |  |  |  |  |  |  |  |  |  |  |  |  |  |  |
| MAF |  |  |  |  |  |  |  |  |  |  |  |  |  |  |  |  |  |
| KDM5D |  |  |  |  |  |  |  |  |  |  |  |  |  |  |  |  |  |
| HBEGF |  |  |  |  |  |  |  |  |  |  |  |  |  |  |  |  |  |
| TTC19 |  |  |  |  |  |  |  |  |  |  |  |  |  |  |  |  |  |
| SHOX2 |  |  |  |  |  |  |  |  |  |  |  |  |  |  |  |  |  |
| PRKN |  |  |  |  |  |  |  |  |  |  |  |  |  |  |  |  |  |
| NDUFA12 |  |  |  |  |  |  |  |  |  |  |  |  |  |  |  |  |  |
| DNAAF2 |  |  |  |  |  |  |  |  |  |  |  |  |  |  |  |  |  |
| RMND1 |  |  |  |  |  |  |  |  |  |  |  |  |  |  |  |  |  |
| RELB |  |  |  |  |  |  |  |  |  |  |  |  |  |  |  |  |  |
| PHGDH |  |  |  |  |  |  |  |  |  |  |  |  |  |  |  |  |  |
| DMC1 |  |  |  |  |  |  |  |  |  |  |  |  |  |  |  |  |  |
| LOC106050102 |  |  |  |  |  |  |  |  |  |  |  |  |  |  |  |  |  |
| DAXX |  |  |  |  |  |  |  |  |  |  |  |  |  |  |  |  |  |
| LIPE |  |  |  |  |  |  |  |  |  |  |  |  |  |  |  |  |  |
| RB1 |  |  |  |  |  |  |  |  |  |  |  |  |  |  |  |  |  |
| KCNA7 |  |  |  |  |  |  |  |  |  |  |  |  |  |  |  |  |  |
| LHX4 |  |  |  |  |  |  |  |  |  |  |  |  |  |  |  |  |  |
| IFNGR1 |  |  |  |  |  |  |  |  |  |  |  |  |  |  |  |  |  |
| SLC26A3 |  |  |  |  |  |  |  |  |  |  |  |  |  |  |  |  |  |
| CCNO |  |  |  |  |  |  |  |  |  |  |  |  |  |  |  |  |  |
| SORT1 |  |  |  |  |  |  |  |  |  |  |  |  |  |  |  |  |  |
| CSNK2A1 |  |  |  |  |  |  |  |  |  |  |  |  |  |  |  |  |  |
| AKT2 |  |  |  |  |  |  |  |  |  |  |  |  |  |  |  |  |  |
| PRG4 |  |  |  |  |  |  |  |  |  |  |  |  |  |  |  |  |  |
| DMRT1 |  |  |  |  |  |  |  |  |  |  |  |  |  |  |  |  |  |
| IRF4 |  |  |  |  |  |  |  |  |  |  |  |  |  |  |  |  |  |
| IL3 |  |  |  |  |  |  |  |  |  |  |  |  |  |  |  |  |  |
| POLR1D |  |  |  |  |  |  |  |  |  |  |  |  |  |  |  |  |  |
| NHLRC1 |  |  |  |  |  |  |  |  |  |  |  |  |  |  |  |  |  |
| NDUFA4 |  |  |  |  |  |  |  |  |  |  |  |  |  |  |  |  |  |
| TAF1 |  |  |  |  |  |  |  |  |  |  |  |  |  |  |  |  |  |
| RPS4Y2 |  |  |  |  |  |  |  |  |  |  |  |  |  |  |  |  |  |
| CA2 |  |  |  |  |  |  |  |  |  |  |  |  |  |  |  |  |  |
| XIRP1 |  |  |  |  |  |  |  |  |  |  |  |  |  |  |  |  |  |
| TRIM55 |  |  |  |  |  |  |  |  |  |  |  |  |  |  |  |  |  |
| DYNC1H1 |  |  |  |  |  |  |  |  |  |  |  |  |  |  |  |  |  |
| NDUFA8 |  |  |  |  |  |  |  |  |  |  |  |  |  |  |  |  |  |
| SLC1A2 |  |  |  |  |  |  |  |  |  |  |  |  |  |  |  |  |  |
| PRNP |  |  |  |  |  |  |  |  |  |  |  |  |  |  |  |  |  |
| SATB2 |  |  |  |  |  |  |  |  |  |  |  |  |  |  |  |  |  |
| GZMB |  |  |  |  |  |  |  |  |  |  |  |  |  |  |  |  |  |
| DGCR2 |  |  |  |  |  |  |  |  |  |  |  |  |  |  |  |  |  |
| MIR27A |  |  |  |  |  |  |  |  |  |  |  |  |  |  |  |  |  |
| MMADHC |  |  |  |  |  |  |  |  |  |  |  |  |  |  |  |  |  |
| CHKA |  |  |  |  |  |  |  |  |  |  |  |  |  |  |  |  |  |
| IL17RD |  |  |  |  |  |  |  |  |  |  |  |  |  |  |  |  |  |
| EPX |  |  |  |  |  |  |  |  |  |  |  |  |  |  |  |  |  |
| DCAF8 |  |  |  |  |  |  |  |  |  |  |  |  |  |  |  |  |  |
| ALOX5AP |  |  |  |  |  |  |  |  |  |  |  |  |  |  |  |  |  |
| LYZ |  |  |  |  |  |  |  |  |  |  |  |  |  |  |  |  |  |
| FABP2 |  |  |  |  |  |  |  |  |  |  |  |  |  |  |  |  |  |
| CTNND2 |  |  |  |  |  |  |  |  |  |  |  |  |  |  |  |  |  |
| SHOX |  |  |  |  |  |  |  |  |  |  |  |  |  |  |  |  |  |
| LRP1 |  |  |  |  |  |  |  |  |  |  |  |  |  |  |  |  |  |
| TGM1 |  |  |  |  |  |  |  |  |  |  |  |  |  |  |  |  |  |
| ANKRD31 |  |  |  |  |  |  |  |  |  |  |  |  |  |  |  |  |  |
| BDKRB2 |  |  |  |  |  |  |  |  |  |  |  |  |  |  |  |  |  |
| EXT2 |  |  |  |  |  |  |  |  |  |  |  |  |  |  |  |  |  |
| COL7A1 |  |  |  |  |  |  |  |  |  |  |  |  |  |  |  |  |  |
| NPHP3-ACAD11 |  |  |  |  |  |  |  |  |  |  |  |  |  |  |  |  |  |
| PGM3 |  |  |  |  |  |  |  |  |  |  |  |  |  |  |  |  |  |
| HAX1 |  |  |  |  |  |  |  |  |  |  |  |  |  |  |  |  |  |
| COX7A1 |  |  |  |  |  |  |  |  |  |  |  |  |  |  |  |  |  |
| CFAP410 |  |  |  |  |  |  |  |  |  |  |  |  |  |  |  |  |  |
| LACTB |  |  |  |  |  |  |  |  |  |  |  |  |  |  |  |  |  |
| GLS |  |  |  |  |  |  |  |  |  |  |  |  |  |  |  |  |  |
| MT-RNR1 |  |  |  |  |  |  |  |  |  |  |  |  |  |  |  |  |  |
| FLII |  |  |  |  |  |  |  |  |  |  |  |  |  |  |  |  |  |
| DMP1 |  |  |  |  |  |  |  |  |  |  |  |  |  |  |  |  |  |
| EDAR |  |  |  |  |  |  |  |  |  |  |  |  |  |  |  |  |  |
| CHUK |  |  |  |  |  |  |  |  |  |  |  |  |  |  |  |  |  |
| AMER1 |  |  |  |  |  |  |  |  |  |  |  |  |  |  |  |  |  |
| CDC42 |  |  |  |  |  |  |  |  |  |  |  |  |  |  |  |  |  |
| STK36 |  |  |  |  |  |  |  |  |  |  |  |  |  |  |  |  |  |
| MRPS7 |  |  |  |  |  |  |  |  |  |  |  |  |  |  |  |  |  |
| PIGT |  |  |  |  |  |  |  |  |  |  |  |  |  |  |  |  |  |
| GJA4 |  |  |  |  |  |  |  |  |  |  |  |  |  |  |  |  |  |
| APBB1 |  |  |  |  |  |  |  |  |  |  |  |  |  |  |  |  |  |
| CAPN15 |  |  |  |  |  |  |  |  |  |  |  |  |  |  |  |  |  |
| TBC1D4 |  |  |  |  |  |  |  |  |  |  |  |  |  |  |  |  |  |
| SLC37A4 |  |  |  |  |  |  |  |  |  |  |  |  |  |  |  |  |  |
| CFAP221 |  |  |  |  |  |  |  |  |  |  |  |  |  |  |  |  |  |
| LARS2-AS1 |  |  |  |  |  |  |  |  |  |  |  |  |  |  |  |  |  |
| MYH10 |  |  |  |  |  |  |  |  |  |  |  |  |  |  |  |  |  |
| CDKL5 |  |  |  |  |  |  |  |  |  |  |  |  |  |  |  |  |  |
| RPS29 |  |  |  |  |  |  |  |  |  |  |  |  |  |  |  |  |  |
| STXBP1 |  |  |  |  |  |  |  |  |  |  |  |  |  |  |  |  |  |
| WNT9B |  |  |  |  |  |  |  |  |  |  |  |  |  |  |  |  |  |
| SCARB1 |  |  |  |  |  |  |  |  |  |  |  |  |  |  |  |  |  |
| TMEM107 |  |  |  |  |  |  |  |  |  |  |  |  |  |  |  |  |  |
| FENDRR |  |  |  |  |  |  |  |  |  |  |  |  |  |  |  |  |  |
| COG8 |  |  |  |  |  |  |  |  |  |  |  |  |  |  |  |  |  |
| F2R |  |  |  |  |  |  |  |  |  |  |  |  |  |  |  |  |  |
| WDR11 |  |  |  |  |  |  |  |  |  |  |  |  |  |  |  |  |  |
| WNT11 |  |  |  |  |  |  |  |  |  |  |  |  |  |  |  |  |  |
| SAMD9 |  |  |  |  |  |  |  |  |  |  |  |  |  |  |  |  |  |
| GHRHR |  |  |  |  |  |  |  |  |  |  |  |  |  |  |  |  |  |
| ALAD |  |  |  |  |  |  |  |  |  |  |  |  |  |  |  |  |  |
| AARS1 |  |  |  |  |  |  |  |  |  |  |  |  |  |  |  |  |  |
| CKB |  |  |  |  |  |  |  |  |  |  |  |  |  |  |  |  |  |
| THRB |  |  |  |  |  |  |  |  |  |  |  |  |  |  |  |  |  |
| RUNX1 |  |  |  |  |  |  |  |  |  |  |  |  |  |  |  |  |  |
| CREB1 |  |  |  |  |  |  |  |  |  |  |  |  |  |  |  |  |  |
| FCGR2B |  |  |  |  |  |  |  |  |  |  |  |  |  |  |  |  |  |
| GNB5 |  |  |  |  |  |  |  |  |  |  |  |  |  |  |  |  |  |
| GLIS2 |  |  |  |  |  |  |  |  |  |  |  |  |  |  |  |  |  |
| MYH1 |  |  |  |  |  |  |  |  |  |  |  |  |  |  |  |  |  |
| IL12A |  |  |  |  |  |  |  |  |  |  |  |  |  |  |  |  |  |
| ENO1 |  |  |  |  |  |  |  |  |  |  |  |  |  |  |  |  |  |
| PIGQ |  |  |  |  |  |  |  |  |  |  |  |  |  |  |  |  |  |
| MAT2A |  |  |  |  |  |  |  |  |  |  |  |  |  |  |  |  |  |
| NFATC3 |  |  |  |  |  |  |  |  |  |  |  |  |  |  |  |  |  |
| NDUFA9 |  |  |  |  |  |  |  |  |  |  |  |  |  |  |  |  |  |
| CIROP |  |  |  |  |  |  |  |  |  |  |  |  |  |  |  |  |  |
| THOC6 |  |  |  |  |  |  |  |  |  |  |  |  |  |  |  |  |  |
| SNAP25 |  |  |  |  |  |  |  |  |  |  |  |  |  |  |  |  |  |
| NDUFA10 |  |  |  |  |  |  |  |  |  |  |  |  |  |  |  |  |  |
| ABCA12 |  |  |  |  |  |  |  |  |  |  |  |  |  |  |  |  |  |
| PRKAR1B |  |  |  |  |  |  |  |  |  |  |  |  |  |  |  |  |  |
| MASP1 |  |  |  |  |  |  |  |  |  |  |  |  |  |  |  |  |  |
| ADAR |  |  |  |  |  |  |  |  |  |  |  |  |  |  |  |  |  |
| KCNA1 |  |  |  |  |  |  |  |  |  |  |  |  |  |  |  |  |  |
| KLHL7 |  |  |  |  |  |  |  |  |  |  |  |  |  |  |  |  |  |
| SUMF1 |  |  |  |  |  |  |  |  |  |  |  |  |  |  |  |  |  |
| KRT19 |  |  |  |  |  |  |  |  |  |  |  |  |  |  |  |  |  |
| MBNL1 |  |  |  |  |  |  |  |  |  |  |  |  |  |  |  |  |  |
| LYRM7 |  |  |  |  |  |  |  |  |  |  |  |  |  |  |  |  |  |
| HYT1 |  |  |  |  |  |  |  |  |  |  |  |  |  |  |  |  |  |
| HYT2 |  |  |  |  |  |  |  |  |  |  |  |  |  |  |  |  |  |
| HYT3 |  |  |  |  |  |  |  |  |  |  |  |  |  |  |  |  |  |
| HYT4 |  |  |  |  |  |  |  |  |  |  |  |  |  |  |  |  |  |
| HYT5 |  |  |  |  |  |  |  |  |  |  |  |  |  |  |  |  |  |
| HYT6 |  |  |  |  |  |  |  |  |  |  |  |  |  |  |  |  |  |
| HYT7 |  |  |  |  |  |  |  |  |  |  |  |  |  |  |  |  |  |
| HYT8 |  |  |  |  |  |  |  |  |  |  |  |  |  |  |  |  |  |
| FSIP2-AS1 |  |  |  |  |  |  |  |  |  |  |  |  |  |  |  |  |  |
| PTGDS |  |  |  |  |  |  |  |  |  |  |  |  |  |  |  |  |  |
| HEY1 |  |  |  |  |  |  |  |  |  |  |  |  |  |  |  |  |  |
| PORCN |  |  |  |  |  |  |  |  |  |  |  |  |  |  |  |  |  |
| DCLRE1C |  |  |  |  |  |  |  |  |  |  |  |  |  |  |  |  |  |
| FZD2 |  |  |  |  |  |  |  |  |  |  |  |  |  |  |  |  |  |
| NTRK3 |  |  |  |  |  |  |  |  |  |  |  |  |  |  |  |  |  |
| TBR1 |  |  |  |  |  |  |  |  |  |  |  |  |  |  |  |  |  |
| IGHE |  |  |  |  |  |  |  |  |  |  |  |  |  |  |  |  |  |
| SNX10 |  |  |  |  |  |  |  |  |  |  |  |  |  |  |  |  |  |
| TBC1D24 |  |  |  |  |  |  |  |  |  |  |  |  |  |  |  |  |  |
| TMPPE |  |  |  |  |  |  |  |  |  |  |  |  |  |  |  |  |  |
| LRBA |  |  |  |  |  |  |  |  |  |  |  |  |  |  |  |  |  |
| GRM7 |  |  |  |  |  |  |  |  |  |  |  |  |  |  |  |  |  |
| ELP1 |  |  |  |  |  |  |  |  |  |  |  |  |  |  |  |  |  |
| ISCA2 |  |  |  |  |  |  |  |  |  |  |  |  |  |  |  |  |  |
| ZNF699 |  |  |  |  |  |  |  |  |  |  |  |  |  |  |  |  |  |
| TYMS |  |  |  |  |  |  |  |  |  |  |  |  |  |  |  |  |  |
| UMPS |  |  |  |  |  |  |  |  |  |  |  |  |  |  |  |  |  |
| ATP5F1E |  |  |  |  |  |  |  |  |  |  |  |  |  |  |  |  |  |
| IRS2 |  |  |  |  |  |  |  |  |  |  |  |  |  |  |  |  |  |
| ITGA2 |  |  |  |  |  |  |  |  |  |  |  |  |  |  |  |  |  |
| FCN2 |  |  |  |  |  |  |  |  |  |  |  |  |  |  |  |  |  |
| MIR1-1 |  |  |  |  |  |  |  |  |  |  |  |  |  |  |  |  |  |
| GRHPR |  |  |  |  |  |  |  |  |  |  |  |  |  |  |  |  |  |
| CARS1 |  |  |  |  |  |  |  |  |  |  |  |  |  |  |  |  |  |
| HNRNPUL2-BSCL2 |  |  |  |  |  |  |  |  |  |  |  |  |  |  |  |  |  |
| SPINK5 |  |  |  |  |  |  |  |  |  |  |  |  |  |  |  |  |  |
| ZRSR2 |  |  |  |  |  |  |  |  |  |  |  |  |  |  |  |  |  |
| SEC23B |  |  |  |  |  |  |  |  |  |  |  |  |  |  |  |  |  |
| TSHR |  |  |  |  |  |  |  |  |  |  |  |  |  |  |  |  |  |
| PLXNA2 |  |  |  |  |  |  |  |  |  |  |  |  |  |  |  |  |  |
| WNT3 |  |  |  |  |  |  |  |  |  |  |  |  |  |  |  |  |  |
| C1S |  |  |  |  |  |  |  |  |  |  |  |  |  |  |  |  |  |
| PIK3R1 |  |  |  |  |  |  |  |  |  |  |  |  |  |  |  |  |  |
| TSPYL1 |  |  |  |  |  |  |  |  |  |  |  |  |  |  |  |  |  |
| MYMK |  |  |  |  |  |  |  |  |  |  |  |  |  |  |  |  |  |
| INF2 |  |  |  |  |  |  |  |  |  |  |  |  |  |  |  |  |  |
| TGFA |  |  |  |  |  |  |  |  |  |  |  |  |  |  |  |  |  |
| AIRE |  |  |  |  |  |  |  |  |  |  |  |  |  |  |  |  |  |
| LIPI |  |  |  |  |  |  |  |  |  |  |  |  |  |  |  |  |  |
| CWC27 |  |  |  |  |  |  |  |  |  |  |  |  |  |  |  |  |  |
| MFF-DT |  |  |  |  |  |  |  |  |  |  |  |  |  |  |  |  |  |
| DDX25 |  |  |  |  |  |  |  |  |  |  |  |  |  |  |  |  |  |
| ASS1 |  |  |  |  |  |  |  |  |  |  |  |  |  |  |  |  |  |
| OBSL1 |  |  |  |  |  |  |  |  |  |  |  |  |  |  |  |  |  |
| ARL13B |  |  |  |  |  |  |  |  |  |  |  |  |  |  |  |  |  |
| RANBP2 |  |  |  |  |  |  |  |  |  |  |  |  |  |  |  |  |  |
| CATSPER2 |  |  |  |  |  |  |  |  |  |  |  |  |  |  |  |  |  |
| PRKDC |  |  |  |  |  |  |  |  |  |  |  |  |  |  |  |  |  |
| OTULIN |  |  |  |  |  |  |  |  |  |  |  |  |  |  |  |  |  |
| ARMC2-AS1 |  |  |  |  |  |  |  |  |  |  |  |  |  |  |  |  |  |
| MBP |  |  |  |  |  |  |  |  |  |  |  |  |  |  |  |  |  |
| MGME1 |  |  |  |  |  |  |  |  |  |  |  |  |  |  |  |  |  |
| ELN-AS1 |  |  |  |  |  |  |  |  |  |  |  |  |  |  |  |  |  |
| HTT |  |  |  |  |  |  |  |  |  |  |  |  |  |  |  |  |  |
| RBBP8 |  |  |  |  |  |  |  |  |  |  |  |  |  |  |  |  |  |
| LIPT1 |  |  |  |  |  |  |  |  |  |  |  |  |  |  |  |  |  |
| IL5 |  |  |  |  |  |  |  |  |  |  |  |  |  |  |  |  |  |
| POMT2 |  |  |  |  |  |  |  |  |  |  |  |  |  |  |  |  |  |
| MRPS22 |  |  |  |  |  |  |  |  |  |  |  |  |  |  |  |  |  |
| TTC21B-AS1 |  |  |  |  |  |  |  |  |  |  |  |  |  |  |  |  |  |
| DGCR5 |  |  |  |  |  |  |  |  |  |  |  |  |  |  |  |  |  |
| MCAM |  |  |  |  |  |  |  |  |  |  |  |  |  |  |  |  |  |
| AOC3 |  |  |  |  |  |  |  |  |  |  |  |  |  |  |  |  |  |
| HHEX |  |  |  |  |  |  |  |  |  |  |  |  |  |  |  |  |  |
| MPDU1 |  |  |  |  |  |  |  |  |  |  |  |  |  |  |  |  |  |
| BICC1 |  |  |  |  |  |  |  |  |  |  |  |  |  |  |  |  |  |
| HPD |  |  |  |  |  |  |  |  |  |  |  |  |  |  |  |  |  |
| ATPAF2 |  |  |  |  |  |  |  |  |  |  |  |  |  |  |  |  |  |
| NFKB2 |  |  |  |  |  |  |  |  |  |  |  |  |  |  |  |  |  |
| SH2B3 |  |  |  |  |  |  |  |  |  |  |  |  |  |  |  |  |  |
| DPYD |  |  |  |  |  |  |  |  |  |  |  |  |  |  |  |  |  |
| DLST |  |  |  |  |  |  |  |  |  |  |  |  |  |  |  |  |  |
| MAGI2 |  |  |  |  |  |  |  |  |  |  |  |  |  |  |  |  |  |
| RTTN |  |  |  |  |  |  |  |  |  |  |  |  |  |  |  |  |  |
| IL23R |  |  |  |  |  |  |  |  |  |  |  |  |  |  |  |  |  |
| CDH5 |  |  |  |  |  |  |  |  |  |  |  |  |  |  |  |  |  |
| RPS28 |  |  |  |  |  |  |  |  |  |  |  |  |  |  |  |  |  |
| SYNPO2L |  |  |  |  |  |  |  |  |  |  |  |  |  |  |  |  |  |
| STRADA |  |  |  |  |  |  |  |  |  |  |  |  |  |  |  |  |  |
| USP53 |  |  |  |  |  |  |  |  |  |  |  |  |  |  |  |  |  |
| IDH2 |  |  |  |  |  |  |  |  |  |  |  |  |  |  |  |  |  |
| PDGFRB |  |  |  |  |  |  |  |  |  |  |  |  |  |  |  |  |  |
| LYRM4 |  |  |  |  |  |  |  |  |  |  |  |  |  |  |  |  |  |
| CRB2 |  |  |  |  |  |  |  |  |  |  |  |  |  |  |  |  |  |
| AQP1 |  |  |  |  |  |  |  |  |  |  |  |  |  |  |  |  |  |
| AKAP6 |  |  |  |  |  |  |  |  |  |  |  |  |  |  |  |  |  |
| LIFR |  |  |  |  |  |  |  |  |  |  |  |  |  |  |  |  |  |
| GGN |  |  |  |  |  |  |  |  |  |  |  |  |  |  |  |  |  |
| CLPP |  |  |  |  |  |  |  |  |  |  |  |  |  |  |  |  |  |
| ACSF3 |  |  |  |  |  |  |  |  |  |  |  |  |  |  |  |  |  |
| USF1 |  |  |  |  |  |  |  |  |  |  |  |  |  |  |  |  |  |
| AK1 |  |  |  |  |  |  |  |  |  |  |  |  |  |  |  |  |  |
| FAHD1 |  |  |  |  |  |  |  |  |  |  |  |  |  |  |  |  |  |
| DDAH2 |  |  |  |  |  |  |  |  |  |  |  |  |  |  |  |  |  |
| MAN2B1 |  |  |  |  |  |  |  |  |  |  |  |  |  |  |  |  |  |
| FUS |  |  |  |  |  |  |  |  |  |  |  |  |  |  |  |  |  |
| GPC6 |  |  |  |  |  |  |  |  |  |  |  |  |  |  |  |  |  |
| UNC80 |  |  |  |  |  |  |  |  |  |  |  |  |  |  |  |  |  |
| NDUFA2 |  |  |  |  |  |  |  |  |  |  |  |  |  |  |  |  |  |
| FREM1 |  |  |  |  |  |  |  |  |  |  |  |  |  |  |  |  |  |
| KLF4 |  |  |  |  |  |  |  |  |  |  |  |  |  |  |  |  |  |
| KLK3 |  |  |  |  |  |  |  |  |  |  |  |  |  |  |  |  |  |
| PARVA |  |  |  |  |  |  |  |  |  |  |  |  |  |  |  |  |  |
| BCL11A |  |  |  |  |  |  |  |  |  |  |  |  |  |  |  |  |  |
| CLDN19 |  |  |  |  |  |  |  |  |  |  |  |  |  |  |  |  |  |
| SP1 |  |  |  |  |  |  |  |  |  |  |  |  |  |  |  |  |  |
| TRAPPC11 |  |  |  |  |  |  |  |  |  |  |  |  |  |  |  |  |  |
| COL6A2 |  |  |  |  |  |  |  |  |  |  |  |  |  |  |  |  |  |
| ELP4 |  |  |  |  |  |  |  |  |  |  |  |  |  |  |  |  |  |
| CCL4 |  |  |  |  |  |  |  |  |  |  |  |  |  |  |  |  |  |
| CATIP-AS2 |  |  |  |  |  |  |  |  |  |  |  |  |  |  |  |  |  |
| PROCR |  |  |  |  |  |  |  |  |  |  |  |  |  |  |  |  |  |
| IL1RAPL2 |  |  |  |  |  |  |  |  |  |  |  |  |  |  |  |  |  |
| PROX1 |  |  |  |  |  |  |  |  |  |  |  |  |  |  |  |  |  |
| ABCD3 |  |  |  |  |  |  |  |  |  |  |  |  |  |  |  |  |  |
| CLMP |  |  |  |  |  |  |  |  |  |  |  |  |  |  |  |  |  |
| RPS7 |  |  |  |  |  |  |  |  |  |  |  |  |  |  |  |  |  |
| EXOSC8 |  |  |  |  |  |  |  |  |  |  |  |  |  |  |  |  |  |
| CDC45 |  |  |  |  |  |  |  |  |  |  |  |  |  |  |  |  |  |
| GATB |  |  |  |  |  |  |  |  |  |  |  |  |  |  |  |  |  |
| SOCS1 |  |  |  |  |  |  |  |  |  |  |  |  |  |  |  |  |  |
| KCNB1 |  |  |  |  |  |  |  |  |  |  |  |  |  |  |  |  |  |
| ERF |  |  |  |  |  |  |  |  |  |  |  |  |  |  |  |  |  |
| SERPINF2 |  |  |  |  |  |  |  |  |  |  |  |  |  |  |  |  |  |
| CAMKMT |  |  |  |  |  |  |  |  |  |  |  |  |  |  |  |  |  |
| PROP1 |  |  |  |  |  |  |  |  |  |  |  |  |  |  |  |  |  |
| DNAAF6 |  |  |  |  |  |  |  |  |  |  |  |  |  |  |  |  |  |
| DAW1 |  |  |  |  |  |  |  |  |  |  |  |  |  |  |  |  |  |
| ZNFX1 |  |  |  |  |  |  |  |  |  |  |  |  |  |  |  |  |  |
| ESS2 |  |  |  |  |  |  |  |  |  |  |  |  |  |  |  |  |  |
| NSUN2 |  |  |  |  |  |  |  |  |  |  |  |  |  |  |  |  |  |
| HCN1 |  |  |  |  |  |  |  |  |  |  |  |  |  |  |  |  |  |
| MIR486-1 |  |  |  |  |  |  |  |  |  |  |  |  |  |  |  |  |  |
| SOX6 |  |  |  |  |  |  |  |  |  |  |  |  |  |  |  |  |  |
| KCNN4 |  |  |  |  |  |  |  |  |  |  |  |  |  |  |  |  |  |
| CDIN1 |  |  |  |  |  |  |  |  |  |  |  |  |  |  |  |  |  |
| CKMT2 |  |  |  |  |  |  |  |  |  |  |  |  |  |  |  |  |  |
| NXF5 |  |  |  |  |  |  |  |  |  |  |  |  |  |  |  |  |  |
| IL12RB1 |  |  |  |  |  |  |  |  |  |  |  |  |  |  |  |  |  |
| MIR15B |  |  |  |  |  |  |  |  |  |  |  |  |  |  |  |  |  |
| DVL2 |  |  |  |  |  |  |  |  |  |  |  |  |  |  |  |  |  |
| PRDX1 |  |  |  |  |  |  |  |  |  |  |  |  |  |  |  |  |  |
| AGRN |  |  |  |  |  |  |  |  |  |  |  |  |  |  |  |  |  |
| WDR72 |  |  |  |  |  |  |  |  |  |  |  |  |  |  |  |  |  |
| PKD1-AS1 |  |  |  |  |  |  |  |  |  |  |  |  |  |  |  |  |  |
| SH2D1A |  |  |  |  |  |  |  |  |  |  |  |  |  |  |  |  |  |
| AXIN1 |  |  |  |  |  |  |  |  |  |  |  |  |  |  |  |  |  |
| TCF20 |  |  |  |  |  |  |  |  |  |  |  |  |  |  |  |  |  |
| ICOSLG |  |  |  |  |  |  |  |  |  |  |  |  |  |  |  |  |  |
| SSBP1 |  |  |  |  |  |  |  |  |  |  |  |  |  |  |  |  |  |
| XPNPEP3 |  |  |  |  |  |  |  |  |  |  |  |  |  |  |  |  |  |
| PIGP |  |  |  |  |  |  |  |  |  |  |  |  |  |  |  |  |  |
| CD59 |  |  |  |  |  |  |  |  |  |  |  |  |  |  |  |  |  |
| RTN4 |  |  |  |  |  |  |  |  |  |  |  |  |  |  |  |  |  |
| NTRK2 |  |  |  |  |  |  |  |  |  |  |  |  |  |  |  |  |  |
| FGF7 |  |  |  |  |  |  |  |  |  |  |  |  |  |  |  |  |  |
| PLP1 |  |  |  |  |  |  |  |  |  |  |  |  |  |  |  |  |  |
| FUT8 |  |  |  |  |  |  |  |  |  |  |  |  |  |  |  |  |  |
| ZMYND10 |  |  |  |  |  |  |  |  |  |  |  |  |  |  |  |  |  |
| HENMT1 |  |  |  |  |  |  |  |  |  |  |  |  |  |  |  |  |  |
| CACNA1H |  |  |  |  |  |  |  |  |  |  |  |  |  |  |  |  |  |
| PEPD |  |  |  |  |  |  |  |  |  |  |  |  |  |  |  |  |  |
| TARID |  |  |  |  |  |  |  |  |  |  |  |  |  |  |  |  |  |
| SLC1A3 |  |  |  |  |  |  |  |  |  |  |  |  |  |  |  |  |  |
| CD3D |  |  |  |  |  |  |  |  |  |  |  |  |  |  |  |  |  |
| EPB41L4A |  |  |  |  |  |  |  |  |  |  |  |  |  |  |  |  |  |
| EEF1A2 |  |  |  |  |  |  |  |  |  |  |  |  |  |  |  |  |  |
| SMG8 |  |  |  |  |  |  |  |  |  |  |  |  |  |  |  |  |  |
| MAPT |  |  |  |  |  |  |  |  |  |  |  |  |  |  |  |  |  |
| KRT5 |  |  |  |  |  |  |  |  |  |  |  |  |  |  |  |  |  |
| MRE11 |  |  |  |  |  |  |  |  |  |  |  |  |  |  |  |  |  |
| KAT5 |  |  |  |  |  |  |  |  |  |  |  |  |  |  |  |  |  |
| CEP104 |  |  |  |  |  |  |  |  |  |  |  |  |  |  |  |  |  |
| A4GALT |  |  |  |  |  |  |  |  |  |  |  |  |  |  |  |  |  |
| FKBP14 |  |  |  |  |  |  |  |  |  |  |  |  |  |  |  |  |  |
| AK2 |  |  |  |  |  |  |  |  |  |  |  |  |  |  |  |  |  |
| TRIP11 |  |  |  |  |  |  |  |  |  |  |  |  |  |  |  |  |  |
| LARGE1 |  |  |  |  |  |  |  |  |  |  |  |  |  |  |  |  |  |
| MIR30B |  |  |  |  |  |  |  |  |  |  |  |  |  |  |  |  |  |
| RBM8A |  |  |  |  |  |  |  |  |  |  |  |  |  |  |  |  |  |
| MAOB |  |  |  |  |  |  |  |  |  |  |  |  |  |  |  |  |  |
| PYGL |  |  |  |  |  |  |  |  |  |  |  |  |  |  |  |  |  |
| KLHL41 |  |  |  |  |  |  |  |  |  |  |  |  |  |  |  |  |  |
| MGAM |  |  |  |  |  |  |  |  |  |  |  |  |  |  |  |  |  |
| GANAB |  |  |  |  |  |  |  |  |  |  |  |  |  |  |  |  |  |
| ALG13 |  |  |  |  |  |  |  |  |  |  |  |  |  |  |  |  |  |
| H19-ICR |  |  |  |  |  |  |  |  |  |  |  |  |  |  |  |  |  |
| OPTN |  |  |  |  |  |  |  |  |  |  |  |  |  |  |  |  |  |
| BCL6 |  |  |  |  |  |  |  |  |  |  |  |  |  |  |  |  |  |
| OTUD5 |  |  |  |  |  |  |  |  |  |  |  |  |  |  |  |  |  |
| ROBO2 |  |  |  |  |  |  |  |  |  |  |  |  |  |  |  |  |  |
| TPP1 |  |  |  |  |  |  |  |  |  |  |  |  |  |  |  |  |  |
| GFRA1 |  |  |  |  |  |  |  |  |  |  |  |  |  |  |  |  |  |
| SLC19A1 |  |  |  |  |  |  |  |  |  |  |  |  |  |  |  |  |  |
| MIR149 |  |  |  |  |  |  |  |  |  |  |  |  |  |  |  |  |  |
| CSF2RA |  |  |  |  |  |  |  |  |  |  |  |  |  |  |  |  |  |
| LTC4S |  |  |  |  |  |  |  |  |  |  |  |  |  |  |  |  |  |
| AFF3 |  |  |  |  |  |  |  |  |  |  |  |  |  |  |  |  |  |
| NAGS |  |  |  |  |  |  |  |  |  |  |  |  |  |  |  |  |  |
| PDLIM3 |  |  |  |  |  |  |  |  |  |  |  |  |  |  |  |  |  |
| DDX3X |  |  |  |  |  |  |  |  |  |  |  |  |  |  |  |  |  |
| HOXA13 |  |  |  |  |  |  |  |  |  |  |  |  |  |  |  |  |  |
| EXTL3 |  |  |  |  |  |  |  |  |  |  |  |  |  |  |  |  |  |
| CCL26 |  |  |  |  |  |  |  |  |  |  |  |  |  |  |  |  |  |
| TP73 |  |  |  |  |  |  |  |  |  |  |  |  |  |  |  |  |  |
| TUSC7 |  |  |  |  |  |  |  |  |  |  |  |  |  |  |  |  |  |
| CHMP2B |  |  |  |  |  |  |  |  |  |  |  |  |  |  |  |  |  |
| DAND5 |  |  |  |  |  |  |  |  |  |  |  |  |  |  |  |  |  |
| CD96 |  |  |  |  |  |  |  |  |  |  |  |  |  |  |  |  |  |
| NDN |  |  |  |  |  |  |  |  |  |  |  |  |  |  |  |  |  |
| SF3B2 |  |  |  |  |  |  |  |  |  |  |  |  |  |  |  |  |  |
| TBL1X |  |  |  |  |  |  |  |  |  |  |  |  |  |  |  |  |  |
| PLVAP |  |  |  |  |  |  |  |  |  |  |  |  |  |  |  |  |  |
| FOXG1 |  |  |  |  |  |  |  |  |  |  |  |  |  |  |  |  |  |
| MIR494 |  |  |  |  |  |  |  |  |  |  |  |  |  |  |  |  |  |
| STRC |  |  |  |  |  |  |  |  |  |  |  |  |  |  |  |  |  |
| GH-LCR |  |  |  |  |  |  |  |  |  |  |  |  |  |  |  |  |  |
| TBXT |  |  |  |  |  |  |  |  |  |  |  |  |  |  |  |  |  |
| ISCU |  |  |  |  |  |  |  |  |  |  |  |  |  |  |  |  |  |
| KCNQ2 |  |  |  |  |  |  |  |  |  |  |  |  |  |  |  |  |  |
| EPB42 |  |  |  |  |  |  |  |  |  |  |  |  |  |  |  |  |  |
| LOC106029312 |  |  |  |  |  |  |  |  |  |  |  |  |  |  |  |  |  |
| IDH1 |  |  |  |  |  |  |  |  |  |  |  |  |  |  |  |  |  |
| ADSL |  |  |  |  |  |  |  |  |  |  |  |  |  |  |  |  |  |
| JAZF1 |  |  |  |  |  |  |  |  |  |  |  |  |  |  |  |  |  |
| ACOX1 |  |  |  |  |  |  |  |  |  |  |  |  |  |  |  |  |  |
| IRF6 |  |  |  |  |  |  |  |  |  |  |  |  |  |  |  |  |  |
| CCL17 |  |  |  |  |  |  |  |  |  |  |  |  |  |  |  |  |  |
| ATP6V1B1 |  |  |  |  |  |  |  |  |  |  |  |  |  |  |  |  |  |
| SLC19A3 |  |  |  |  |  |  |  |  |  |  |  |  |  |  |  |  |  |
| SUN2 |  |  |  |  |  |  |  |  |  |  |  |  |  |  |  |  |  |
| TGFBR3 |  |  |  |  |  |  |  |  |  |  |  |  |  |  |  |  |  |
| MT-LIPCAR |  |  |  |  |  |  |  |  |  |  |  |  |  |  |  |  |  |
| RAB3GAP1 |  |  |  |  |  |  |  |  |  |  |  |  |  |  |  |  |  |
| STAT6 |  |  |  |  |  |  |  |  |  |  |  |  |  |  |  |  |  |
| MGAT2 |  |  |  |  |  |  |  |  |  |  |  |  |  |  |  |  |  |
| SFTPA2 |  |  |  |  |  |  |  |  |  |  |  |  |  |  |  |  |  |
| MIR483 |  |  |  |  |  |  |  |  |  |  |  |  |  |  |  |  |  |
| MRPL12 |  |  |  |  |  |  |  |  |  |  |  |  |  |  |  |  |  |
| ITGA4 |  |  |  |  |  |  |  |  |  |  |  |  |  |  |  |  |  |
| GMPPB |  |  |  |  |  |  |  |  |  |  |  |  |  |  |  |  |  |
| SLC35A2 |  |  |  |  |  |  |  |  |  |  |  |  |  |  |  |  |  |
| CFAP52 |  |  |  |  |  |  |  |  |  |  |  |  |  |  |  |  |  |
| THSD1 |  |  |  |  |  |  |  |  |  |  |  |  |  |  |  |  |  |
| PPP3CB |  |  |  |  |  |  |  |  |  |  |  |  |  |  |  |  |  |
| XIRP2 |  |  |  |  |  |  |  |  |  |  |  |  |  |  |  |  |  |
| IL2RG |  |  |  |  |  |  |  |  |  |  |  |  |  |  |  |  |  |
| PPP2R5D |  |  |  |  |  |  |  |  |  |  |  |  |  |  |  |  |  |
| DGCR6 |  |  |  |  |  |  |  |  |  |  |  |  |  |  |  |  |  |
| PC |  |  |  |  |  |  |  |  |  |  |  |  |  |  |  |  |  |
| SCN3A |  |  |  |  |  |  |  |  |  |  |  |  |  |  |  |  |  |
| SYNGAP1 |  |  |  |  |  |  |  |  |  |  |  |  |  |  |  |  |  |
| ITCH |  |  |  |  |  |  |  |  |  |  |  |  |  |  |  |  |  |
| SLC10A2 |  |  |  |  |  |  |  |  |  |  |  |  |  |  |  |  |  |
| NUS1 |  |  |  |  |  |  |  |  |  |  |  |  |  |  |  |  |  |
| SLC39A13 |  |  |  |  |  |  |  |  |  |  |  |  |  |  |  |  |  |
| WNT7B |  |  |  |  |  |  |  |  |  |  |  |  |  |  |  |  |  |
| COA6 |  |  |  |  |  |  |  |  |  |  |  |  |  |  |  |  |  |
| C12orf29 |  |  |  |  |  |  |  |  |  |  |  |  |  |  |  |  |  |
| STAMBP |  |  |  |  |  |  |  |  |  |  |  |  |  |  |  |  |  |
| LOC105371046 |  |  |  |  |  |  |  |  |  |  |  |  |  |  |  |  |  |
| PTCH2 |  |  |  |  |  |  |  |  |  |  |  |  |  |  |  |  |  |
| PALLD |  |  |  |  |  |  |  |  |  |  |  |  |  |  |  |  |  |
| LOC109280163 |  |  |  |  |  |  |  |  |  |  |  |  |  |  |  |  |  |
| DDOST |  |  |  |  |  |  |  |  |  |  |  |  |  |  |  |  |  |
| FGFRL1 |  |  |  |  |  |  |  |  |  |  |  |  |  |  |  |  |  |
| SLC41A1 |  |  |  |  |  |  |  |  |  |  |  |  |  |  |  |  |  |
| AHSP |  |  |  |  |  |  |  |  |  |  |  |  |  |  |  |  |  |
| GREB1L |  |  |  |  |  |  |  |  |  |  |  |  |  |  |  |  |  |
| LCK |  |  |  |  |  |  |  |  |  |  |  |  |  |  |  |  |  |
| ALG3 |  |  |  |  |  |  |  |  |  |  |  |  |  |  |  |  |  |
| NCAPH2 |  |  |  |  |  |  |  |  |  |  |  |  |  |  |  |  |  |
| PRKCQ |  |  |  |  |  |  |  |  |  |  |  |  |  |  |  |  |  |
| ASL |  |  |  |  |  |  |  |  |  |  |  |  |  |  |  |  |  |
| MPDZ |  |  |  |  |  |  |  |  |  |  |  |  |  |  |  |  |  |
| MIR222 |  |  |  |  |  |  |  |  |  |  |  |  |  |  |  |  |  |
| CNTROB |  |  |  |  |  |  |  |  |  |  |  |  |  |  |  |  |  |
| KAT2B |  |  |  |  |  |  |  |  |  |  |  |  |  |  |  |  |  |
| NRP1 |  |  |  |  |  |  |  |  |  |  |  |  |  |  |  |  |  |
| CPOX |  |  |  |  |  |  |  |  |  |  |  |  |  |  |  |  |  |
| FANCD2OS |  |  |  |  |  |  |  |  |  |  |  |  |  |  |  |  |  |
| VLDLR |  |  |  |  |  |  |  |  |  |  |  |  |  |  |  |  |  |
| PDE6D |  |  |  |  |  |  |  |  |  |  |  |  |  |  |  |  |  |
| CDKN1B |  |  |  |  |  |  |  |  |  |  |  |  |  |  |  |  |  |
| HBB-LCR |  |  |  |  |  |  |  |  |  |  |  |  |  |  |  |  |  |
| PGAP1 |  |  |  |  |  |  |  |  |  |  |  |  |  |  |  |  |  |
| MKRN3 |  |  |  |  |  |  |  |  |  |  |  |  |  |  |  |  |  |
| GNPTG |  |  |  |  |  |  |  |  |  |  |  |  |  |  |  |  |  |
| PDSS1 |  |  |  |  |  |  |  |  |  |  |  |  |  |  |  |  |  |
| MT-TA |  |  |  |  |  |  |  |  |  |  |  |  |  |  |  |  |  |
| CCR1 |  |  |  |  |  |  |  |  |  |  |  |  |  |  |  |  |  |
| PIGN |  |  |  |  |  |  |  |  |  |  |  |  |  |  |  |  |  |
| LAMB3 |  |  |  |  |  |  |  |  |  |  |  |  |  |  |  |  |  |
| PDE11A |  |  |  |  |  |  |  |  |  |  |  |  |  |  |  |  |  |
| TUBA1A |  |  |  |  |  |  |  |  |  |  |  |  |  |  |  |  |  |
| VASP |  |  |  |  |  |  |  |  |  |  |  |  |  |  |  |  |  |
| RIPK4 |  |  |  |  |  |  |  |  |  |  |  |  |  |  |  |  |  |
| APTX |  |  |  |  |  |  |  |  |  |  |  |  |  |  |  |  |  |
| C12orf57 |  |  |  |  |  |  |  |  |  |  |  |  |  |  |  |  |  |
| COPB2 |  |  |  |  |  |  |  |  |  |  |  |  |  |  |  |  |  |
| GBA2 |  |  |  |  |  |  |  |  |  |  |  |  |  |  |  |  |  |
| RNPC3 |  |  |  |  |  |  |  |  |  |  |  |  |  |  |  |  |  |
| OCA2 |  |  |  |  |  |  |  |  |  |  |  |  |  |  |  |  |  |
| WNT10B |  |  |  |  |  |  |  |  |  |  |  |  |  |  |  |  |  |
| TRAF1 |  |  |  |  |  |  |  |  |  |  |  |  |  |  |  |  |  |
| FARSB |  |  |  |  |  |  |  |  |  |  |  |  |  |  |  |  |  |
| MYO1H |  |  |  |  |  |  |  |  |  |  |  |  |  |  |  |  |  |
| IL21 |  |  |  |  |  |  |  |  |  |  |  |  |  |  |  |  |  |
| NTS |  |  |  |  |  |  |  |  |  |  |  |  |  |  |  |  |  |
| SEMA3D |  |  |  |  |  |  |  |  |  |  |  |  |  |  |  |  |  |
| LRIG2 |  |  |  |  |  |  |  |  |  |  |  |  |  |  |  |  |  |
| DPM2 |  |  |  |  |  |  |  |  |  |  |  |  |  |  |  |  |  |
| KISS1R |  |  |  |  |  |  |  |  |  |  |  |  |  |  |  |  |  |
| MLH1 |  |  |  |  |  |  |  |  |  |  |  |  |  |  |  |  |  |
| MSH3 |  |  |  |  |  |  |  |  |  |  |  |  |  |  |  |  |  |
| RPS27 |  |  |  |  |  |  |  |  |  |  |  |  |  |  |  |  |  |
| DPP6 |  |  |  |  |  |  |  |  |  |  |  |  |  |  |  |  |  |
| SLC10A7 |  |  |  |  |  |  |  |  |  |  |  |  |  |  |  |  |  |
| MCMDC2 |  |  |  |  |  |  |  |  |  |  |  |  |  |  |  |  |  |
| ANTXR1 |  |  |  |  |  |  |  |  |  |  |  |  |  |  |  |  |  |
| KCNAB1 |  |  |  |  |  |  |  |  |  |  |  |  |  |  |  |  |  |
| ZP2 |  |  |  |  |  |  |  |  |  |  |  |  |  |  |  |  |  |
| MIB2 |  |  |  |  |  |  |  |  |  |  |  |  |  |  |  |  |  |
| COQ8A |  |  |  |  |  |  |  |  |  |  |  |  |  |  |  |  |  |
| NOL3 |  |  |  |  |  |  |  |  |  |  |  |  |  |  |  |  |  |
| KIAA0319L |  |  |  |  |  |  |  |  |  |  |  |  |  |  |  |  |  |
| GNS |  |  |  |  |  |  |  |  |  |  |  |  |  |  |  |  |  |
| NIPA1 |  |  |  |  |  |  |  |  |  |  |  |  |  |  |  |  |  |
| AQP4 |  |  |  |  |  |  |  |  |  |  |  |  |  |  |  |  |  |
| EIF2B4 |  |  |  |  |  |  |  |  |  |  |  |  |  |  |  |  |  |
| COQ7 |  |  |  |  |  |  |  |  |  |  |  |  |  |  |  |  |  |
| NBPF12 |  |  |  |  |  |  |  |  |  |  |  |  |  |  |  |  |  |
| SCARF2 |  |  |  |  |  |  |  |  |  |  |  |  |  |  |  |  |  |
| ICOS |  |  |  |  |  |  |  |  |  |  |  |  |  |  |  |  |  |
| ALG11 |  |  |  |  |  |  |  |  |  |  |  |  |  |  |  |  |  |
| RFT1 |  |  |  |  |  |  |  |  |  |  |  |  |  |  |  |  |  |
| BUB1 |  |  |  |  |  |  |  |  |  |  |  |  |  |  |  |  |  |
| PAX3 |  |  |  |  |  |  |  |  |  |  |  |  |  |  |  |  |  |
| PFAS |  |  |  |  |  |  |  |  |  |  |  |  |  |  |  |  |  |
| DCC |  |  |  |  |  |  |  |  |  |  |  |  |  |  |  |  |  |
| DPAGT1 |  |  |  |  |  |  |  |  |  |  |  |  |  |  |  |  |  |
| CBY1 |  |  |  |  |  |  |  |  |  |  |  |  |  |  |  |  |  |
| KATNIP |  |  |  |  |  |  |  |  |  |  |  |  |  |  |  |  |  |
| SSR4 |  |  |  |  |  |  |  |  |  |  |  |  |  |  |  |  |  |
| MYO18B |  |  |  |  |  |  |  |  |  |  |  |  |  |  |  |  |  |
| CFHR4 |  |  |  |  |  |  |  |  |  |  |  |  |  |  |  |  |  |
| ALDOB |  |  |  |  |  |  |  |  |  |  |  |  |  |  |  |  |  |
| PLTP |  |  |  |  |  |  |  |  |  |  |  |  |  |  |  |  |  |
| SP7 |  |  |  |  |  |  |  |  |  |  |  |  |  |  |  |  |  |
| STAC3 |  |  |  |  |  |  |  |  |  |  |  |  |  |  |  |  |  |
| HSD3B1 |  |  |  |  |  |  |  |  |  |  |  |  |  |  |  |  |  |
| MNS1 |  |  |  |  |  |  |  |  |  |  |  |  |  |  |  |  |  |
| PRKCH |  |  |  |  |  |  |  |  |  |  |  |  |  |  |  |  |  |
| BCL2L1 |  |  |  |  |  |  |  |  |  |  |  |  |  |  |  |  |  |
| ABCC1 |  |  |  |  |  |  |  |  |  |  |  |  |  |  |  |  |  |
| XRCC5 |  |  |  |  |  |  |  |  |  |  |  |  |  |  |  |  |  |
| PIGY |  |  |  |  |  |  |  |  |  |  |  |  |  |  |  |  |  |
| TNFRSF13C |  |  |  |  |  |  |  |  |  |  |  |  |  |  |  |  |  |
| SEC31A |  |  |  |  |  |  |  |  |  |  |  |  |  |  |  |  |  |
| TAF6 |  |  |  |  |  |  |  |  |  |  |  |  |  |  |  |  |  |
| SYT2 |  |  |  |  |  |  |  |  |  |  |  |  |  |  |  |  |  |
| SLC6A3 |  |  |  |  |  |  |  |  |  |  |  |  |  |  |  |  |  |
| WNT2B |  |  |  |  |  |  |  |  |  |  |  |  |  |  |  |  |  |
| TFAP2A |  |  |  |  |  |  |  |  |  |  |  |  |  |  |  |  |  |
| PDE4B |  |  |  |  |  |  |  |  |  |  |  |  |  |  |  |  |  |
| COL11A1 |  |  |  |  |  |  |  |  |  |  |  |  |  |  |  |  |  |
| CASP7 |  |  |  |  |  |  |  |  |  |  |  |  |  |  |  |  |  |
| LBP |  |  |  |  |  |  |  |  |  |  |  |  |  |  |  |  |  |
| ABHD5 |  |  |  |  |  |  |  |  |  |  |  |  |  |  |  |  |  |
| C11orf65 |  |  |  |  |  |  |  |  |  |  |  |  |  |  |  |  |  |
| TUBG1 |  |  |  |  |  |  |  |  |  |  |  |  |  |  |  |  |  |
| SLC25A11 |  |  |  |  |  |  |  |  |  |  |  |  |  |  |  |  |  |
| GARS1 |  |  |  |  |  |  |  |  |  |  |  |  |  |  |  |  |  |
| CSF2RB |  |  |  |  |  |  |  |  |  |  |  |  |  |  |  |  |  |
| SGO1-AS1 |  |  |  |  |  |  |  |  |  |  |  |  |  |  |  |  |  |
| AAAS |  |  |  |  |  |  |  |  |  |  |  |  |  |  |  |  |  |
| CD81 |  |  |  |  |  |  |  |  |  |  |  |  |  |  |  |  |  |
| NCAPG2 |  |  |  |  |  |  |  |  |  |  |  |  |  |  |  |  |  |
| MIR196A1 |  |  |  |  |  |  |  |  |  |  |  |  |  |  |  |  |  |
| TBX6 |  |  |  |  |  |  |  |  |  |  |  |  |  |  |  |  |  |
| STT3B |  |  |  |  |  |  |  |  |  |  |  |  |  |  |  |  |  |
| HUWE1 |  |  |  |  |  |  |  |  |  |  |  |  |  |  |  |  |  |
| DNASE1L3 |  |  |  |  |  |  |  |  |  |  |  |  |  |  |  |  |  |
| TLR8 |  |  |  |  |  |  |  |  |  |  |  |  |  |  |  |  |  |
| INSL3 |  |  |  |  |  |  |  |  |  |  |  |  |  |  |  |  |  |
| NOG |  |  |  |  |  |  |  |  |  |  |  |  |  |  |  |  |  |
| STK4 |  |  |  |  |  |  |  |  |  |  |  |  |  |  |  |  |  |
| C5 |  |  |  |  |  |  |  |  |  |  |  |  |  |  |  |  |  |
| FCGR3A |  |  |  |  |  |  |  |  |  |  |  |  |  |  |  |  |  |
| SLC22A4 |  |  |  |  |  |  |  |  |  |  |  |  |  |  |  |  |  |
| CALB2 |  |  |  |  |  |  |  |  |  |  |  |  |  |  |  |  |  |
| SRPRA |  |  |  |  |  |  |  |  |  |  |  |  |  |  |  |  |  |
| ANK3 |  |  |  |  |  |  |  |  |  |  |  |  |  |  |  |  |  |
| BPTF |  |  |  |  |  |  |  |  |  |  |  |  |  |  |  |  |  |
| AIP |  |  |  |  |  |  |  |  |  |  |  |  |  |  |  |  |  |
| CCDC28B |  |  |  |  |  |  |  |  |  |  |  |  |  |  |  |  |  |
| AP1B1 |  |  |  |  |  |  |  |  |  |  |  |  |  |  |  |  |  |
| PDLIM1 |  |  |  |  |  |  |  |  |  |  |  |  |  |  |  |  |  |
| HCRT |  |  |  |  |  |  |  |  |  |  |  |  |  |  |  |  |  |
| IRF2BP2 |  |  |  |  |  |  |  |  |  |  |  |  |  |  |  |  |  |
| KRT18 |  |  |  |  |  |  |  |  |  |  |  |  |  |  |  |  |  |
| SLC11A1 |  |  |  |  |  |  |  |  |  |  |  |  |  |  |  |  |  |
| DEAF1 |  |  |  |  |  |  |  |  |  |  |  |  |  |  |  |  |  |
| CLTC |  |  |  |  |  |  |  |  |  |  |  |  |  |  |  |  |  |
| TRIO |  |  |  |  |  |  |  |  |  |  |  |  |  |  |  |  |  |
| LOC107303338 |  |  |  |  |  |  |  |  |  |  |  |  |  |  |  |  |  |
| CYP7B1 |  |  |  |  |  |  |  |  |  |  |  |  |  |  |  |  |  |
| NOTCH4 |  |  |  |  |  |  |  |  |  |  |  |  |  |  |  |  |  |
| ITGAL |  |  |  |  |  |  |  |  |  |  |  |  |  |  |  |  |  |
| CFAP298-TCP10L |  |  |  |  |  |  |  |  |  |  |  |  |  |  |  |  |  |
| ANTXR2 |  |  |  |  |  |  |  |  |  |  |  |  |  |  |  |  |  |
| SCAPER |  |  |  |  |  |  |  |  |  |  |  |  |  |  |  |  |  |
| CHIT1 |  |  |  |  |  |  |  |  |  |  |  |  |  |  |  |  |  |
| ATG5 |  |  |  |  |  |  |  |  |  |  |  |  |  |  |  |  |  |
| PIK3R2 |  |  |  |  |  |  |  |  |  |  |  |  |  |  |  |  |  |
| SHC1 |  |  |  |  |  |  |  |  |  |  |  |  |  |  |  |  |  |
| TMEM165 |  |  |  |  |  |  |  |  |  |  |  |  |  |  |  |  |  |
| COX14 |  |  |  |  |  |  |  |  |  |  |  |  |  |  |  |  |  |
| RLBP1 |  |  |  |  |  |  |  |  |  |  |  |  |  |  |  |  |  |
| ATP12A |  |  |  |  |  |  |  |  |  |  |  |  |  |  |  |  |  |
| CANT1 |  |  |  |  |  |  |  |  |  |  |  |  |  |  |  |  |  |
| ALG6 |  |  |  |  |  |  |  |  |  |  |  |  |  |  |  |  |  |
| MMAB |  |  |  |  |  |  |  |  |  |  |  |  |  |  |  |  |  |
| IAPP |  |  |  |  |  |  |  |  |  |  |  |  |  |  |  |  |  |
| PYCR1 |  |  |  |  |  |  |  |  |  |  |  |  |  |  |  |  |  |
| SLC52A3 |  |  |  |  |  |  |  |  |  |  |  |  |  |  |  |  |  |
| MED23 |  |  |  |  |  |  |  |  |  |  |  |  |  |  |  |  |  |
| RARS2 |  |  |  |  |  |  |  |  |  |  |  |  |  |  |  |  |  |
| NTRK1 |  |  |  |  |  |  |  |  |  |  |  |  |  |  |  |  |  |
| NFKBIA |  |  |  |  |  |  |  |  |  |  |  |  |  |  |  |  |  |
| PARD3 |  |  |  |  |  |  |  |  |  |  |  |  |  |  |  |  |  |
| FGF17 |  |  |  |  |  |  |  |  |  |  |  |  |  |  |  |  |  |
| COL6A3 |  |  |  |  |  |  |  |  |  |  |  |  |  |  |  |  |  |
| RIPK1 |  |  |  |  |  |  |  |  |  |  |  |  |  |  |  |  |  |
| KIF24 |  |  |  |  |  |  |  |  |  |  |  |  |  |  |  |  |  |
| ARSA |  |  |  |  |  |  |  |  |  |  |  |  |  |  |  |  |  |
| CENPT |  |  |  |  |  |  |  |  |  |  |  |  |  |  |  |  |  |
| HIC1 |  |  |  |  |  |  |  |  |  |  |  |  |  |  |  |  |  |
| MICA |  |  |  |  |  |  |  |  |  |  |  |  |  |  |  |  |  |
| RPS14 |  |  |  |  |  |  |  |  |  |  |  |  |  |  |  |  |  |
| MAPRE2 |  |  |  |  |  |  |  |  |  |  |  |  |  |  |  |  |  |
| CDKN2B |  |  |  |  |  |  |  |  |  |  |  |  |  |  |  |  |  |
| ATP6V1E1 |  |  |  |  |  |  |  |  |  |  |  |  |  |  |  |  |  |
| COL12A1 |  |  |  |  |  |  |  |  |  |  |  |  |  |  |  |  |  |
| FLRT2 |  |  |  |  |  |  |  |  |  |  |  |  |  |  |  |  |  |
| TRIM54 |  |  |  |  |  |  |  |  |  |  |  |  |  |  |  |  |  |
| FADD |  |  |  |  |  |  |  |  |  |  |  |  |  |  |  |  |  |
| ITGA3 |  |  |  |  |  |  |  |  |  |  |  |  |  |  |  |  |  |
| HERC2 |  |  |  |  |  |  |  |  |  |  |  |  |  |  |  |  |  |
| KCNN2 |  |  |  |  |  |  |  |  |  |  |  |  |  |  |  |  |  |
| DNAH7 |  |  |  |  |  |  |  |  |  |  |  |  |  |  |  |  |  |
| EXOC6B |  |  |  |  |  |  |  |  |  |  |  |  |  |  |  |  |  |
| NACC1 |  |  |  |  |  |  |  |  |  |  |  |  |  |  |  |  |  |
| SRSF2 |  |  |  |  |  |  |  |  |  |  |  |  |  |  |  |  |  |
| MAPKBP1 |  |  |  |  |  |  |  |  |  |  |  |  |  |  |  |  |  |
| CD274 |  |  |  |  |  |  |  |  |  |  |  |  |  |  |  |  |  |
| ESRRG |  |  |  |  |  |  |  |  |  |  |  |  |  |  |  |  |  |
| RIGI |  |  |  |  |  |  |  |  |  |  |  |  |  |  |  |  |  |
| ATN1 |  |  |  |  |  |  |  |  |  |  |  |  |  |  |  |  |  |
| HSP90AA1 |  |  |  |  |  |  |  |  |  |  |  |  |  |  |  |  |  |
| POMGNT1 |  |  |  |  |  |  |  |  |  |  |  |  |  |  |  |  |  |
| KHDRBS1 |  |  |  |  |  |  |  |  |  |  |  |  |  |  |  |  |  |
| NEUROG3 |  |  |  |  |  |  |  |  |  |  |  |  |  |  |  |  |  |
| MECOM |  |  |  |  |  |  |  |  |  |  |  |  |  |  |  |  |  |
| TNFRSF25 |  |  |  |  |  |  |  |  |  |  |  |  |  |  |  |  |  |
| KCNK1 |  |  |  |  |  |  |  |  |  |  |  |  |  |  |  |  |  |
| SRP19 |  |  |  |  |  |  |  |  |  |  |  |  |  |  |  |  |  |
| COQ5 |  |  |  |  |  |  |  |  |  |  |  |  |  |  |  |  |  |
| SMAD1 |  |  |  |  |  |  |  |  |  |  |  |  |  |  |  |  |  |
| NES |  |  |  |  |  |  |  |  |  |  |  |  |  |  |  |  |  |
| AMPD3 |  |  |  |  |  |  |  |  |  |  |  |  |  |  |  |  |  |
| NIPSNAP3B |  |  |  |  |  |  |  |  |  |  |  |  |  |  |  |  |  |
| NRTN |  |  |  |  |  |  |  |  |  |  |  |  |  |  |  |  |  |
| CCR3 |  |  |  |  |  |  |  |  |  |  |  |  |  |  |  |  |  |
| PCSK1 |  |  |  |  |  |  |  |  |  |  |  |  |  |  |  |  |  |
| LOC107982234 |  |  |  |  |  |  |  |  |  |  |  |  |  |  |  |  |  |
| S100A9 |  |  |  |  |  |  |  |  |  |  |  |  |  |  |  |  |  |
| LOC107303340 |  |  |  |  |  |  |  |  |  |  |  |  |  |  |  |  |  |
| ADRA2A |  |  |  |  |  |  |  |  |  |  |  |  |  |  |  |  |  |
| SEC24D |  |  |  |  |  |  |  |  |  |  |  |  |  |  |  |  |  |
| NUP93 |  |  |  |  |  |  |  |  |  |  |  |  |  |  |  |  |  |
| DPM3 |  |  |  |  |  |  |  |  |  |  |  |  |  |  |  |  |  |
| ATFB1 |  |  |  |  |  |  |  |  |  |  |  |  |  |  |  |  |  |
| NSDHL |  |  |  |  |  |  |  |  |  |  |  |  |  |  |  |  |  |
| KAT6A |  |  |  |  |  |  |  |  |  |  |  |  |  |  |  |  |  |
| MET |  |  |  |  |  |  |  |  |  |  |  |  |  |  |  |  |  |
| KMT5B |  |  |  |  |  |  |  |  |  |  |  |  |  |  |  |  |  |
| TMEM138 |  |  |  |  |  |  |  |  |  |  |  |  |  |  |  |  |  |
| M6PR |  |  |  |  |  |  |  |  |  |  |  |  |  |  |  |  |  |
| RPL18 |  |  |  |  |  |  |  |  |  |  |  |  |  |  |  |  |  |
| MIR101-1 |  |  |  |  |  |  |  |  |  |  |  |  |  |  |  |  |  |
| MILR1 |  |  |  |  |  |  |  |  |  |  |  |  |  |  |  |  |  |
| ATXN10 |  |  |  |  |  |  |  |  |  |  |  |  |  |  |  |  |  |
| CALD1 |  |  |  |  |  |  |  |  |  |  |  |  |  |  |  |  |  |
| MUS81 |  |  |  |  |  |  |  |  |  |  |  |  |  |  |  |  |  |
| QDPR |  |  |  |  |  |  |  |  |  |  |  |  |  |  |  |  |  |
| PFKM |  |  |  |  |  |  |  |  |  |  |  |  |  |  |  |  |  |
| KCNE4 |  |  |  |  |  |  |  |  |  |  |  |  |  |  |  |  |  |
| PLEK |  |  |  |  |  |  |  |  |  |  |  |  |  |  |  |  |  |
| HTRA1 |  |  |  |  |  |  |  |  |  |  |  |  |  |  |  |  |  |
| AXDND1 |  |  |  |  |  |  |  |  |  |  |  |  |  |  |  |  |  |
| HMCN1 |  |  |  |  |  |  |  |  |  |  |  |  |  |  |  |  |  |
| KDM6B |  |  |  |  |  |  |  |  |  |  |  |  |  |  |  |  |  |
| RLN1 |  |  |  |  |  |  |  |  |  |  |  |  |  |  |  |  |  |
| DNAH6 |  |  |  |  |  |  |  |  |  |  |  |  |  |  |  |  |  |
| CEP41 |  |  |  |  |  |  |  |  |  |  |  |  |  |  |  |  |  |
| ATP5F1D |  |  |  |  |  |  |  |  |  |  |  |  |  |  |  |  |  |
| PGAP2 |  |  |  |  |  |  |  |  |  |  |  |  |  |  |  |  |  |
| UQCC2 |  |  |  |  |  |  |  |  |  |  |  |  |  |  |  |  |  |
| CNR1 |  |  |  |  |  |  |  |  |  |  |  |  |  |  |  |  |  |
| GDF3 |  |  |  |  |  |  |  |  |  |  |  |  |  |  |  |  |  |
| RPL31 |  |  |  |  |  |  |  |  |  |  |  |  |  |  |  |  |  |
| MIR191 |  |  |  |  |  |  |  |  |  |  |  |  |  |  |  |  |  |
| KLKB1 |  |  |  |  |  |  |  |  |  |  |  |  |  |  |  |  |  |
| COL17A1 |  |  |  |  |  |  |  |  |  |  |  |  |  |  |  |  |  |
| UBE2L3 |  |  |  |  |  |  |  |  |  |  |  |  |  |  |  |  |  |
| ATR |  |  |  |  |  |  |  |  |  |  |  |  |  |  |  |  |  |
| CFAP45 |  |  |  |  |  |  |  |  |  |  |  |  |  |  |  |  |  |
| SIN3A |  |  |  |  |  |  |  |  |  |  |  |  |  |  |  |  |  |
| DRD4 |  |  |  |  |  |  |  |  |  |  |  |  |  |  |  |  |  |
| TOPORS |  |  |  |  |  |  |  |  |  |  |  |  |  |  |  |  |  |
| SPRY4 |  |  |  |  |  |  |  |  |  |  |  |  |  |  |  |  |  |
| PNKP |  |  |  |  |  |  |  |  |  |  |  |  |  |  |  |  |  |
| RPS10-NUDT3 |  |  |  |  |  |  |  |  |  |  |  |  |  |  |  |  |  |
| RO60 |  |  |  |  |  |  |  |  |  |  |  |  |  |  |  |  |  |
| NIPA2 |  |  |  |  |  |  |  |  |  |  |  |  |  |  |  |  |  |
| ATAD3A |  |  |  |  |  |  |  |  |  |  |  |  |  |  |  |  |  |
| VAPB |  |  |  |  |  |  |  |  |  |  |  |  |  |  |  |  |  |
| PYCR2 |  |  |  |  |  |  |  |  |  |  |  |  |  |  |  |  |  |
| CRTAP |  |  |  |  |  |  |  |  |  |  |  |  |  |  |  |  |  |
| ACSL4 |  |  |  |  |  |  |  |  |  |  |  |  |  |  |  |  |  |
| D2HGDH |  |  |  |  |  |  |  |  |  |  |  |  |  |  |  |  |  |
| HEATR3 |  |  |  |  |  |  |  |  |  |  |  |  |  |  |  |  |  |
| SLC7A9 |  |  |  |  |  |  |  |  |  |  |  |  |  |  |  |  |  |
| DEL16P13.3 |  |  |  |  |  |  |  |  |  |  |  |  |  |  |  |  |  |
| PPM1B |  |  |  |  |  |  |  |  |  |  |  |  |  |  |  |  |  |
| CA12 |  |  |  |  |  |  |  |  |  |  |  |  |  |  |  |  |  |
| PTLS |  |  |  |  |  |  |  |  |  |  |  |  |  |  |  |  |  |
| SLC26A4 |  |  |  |  |  |  |  |  |  |  |  |  |  |  |  |  |  |
| WAC |  |  |  |  |  |  |  |  |  |  |  |  |  |  |  |  |  |
| DSTYK |  |  |  |  |  |  |  |  |  |  |  |  |  |  |  |  |  |
| EXT1 |  |  |  |  |  |  |  |  |  |  |  |  |  |  |  |  |  |
| KRIT1 |  |  |  |  |  |  |  |  |  |  |  |  |  |  |  |  |  |
| CYSLTR1 |  |  |  |  |  |  |  |  |  |  |  |  |  |  |  |  |  |
| TULP3 |  |  |  |  |  |  |  |  |  |  |  |  |  |  |  |  |  |
| CGA |  |  |  |  |  |  |  |  |  |  |  |  |  |  |  |  |  |
| MBD5 |  |  |  |  |  |  |  |  |  |  |  |  |  |  |  |  |  |
| FPGT-TNNI3K |  |  |  |  |  |  |  |  |  |  |  |  |  |  |  |  |  |
| TBCE |  |  |  |  |  |  |  |  |  |  |  |  |  |  |  |  |  |
| ACO2 |  |  |  |  |  |  |  |  |  |  |  |  |  |  |  |  |  |
| RNF8 |  |  |  |  |  |  |  |  |  |  |  |  |  |  |  |  |  |
| JAK1 |  |  |  |  |  |  |  |  |  |  |  |  |  |  |  |  |  |
| FTL |  |  |  |  |  |  |  |  |  |  |  |  |  |  |  |  |  |
| LRRK2 |  |  |  |  |  |  |  |  |  |  |  |  |  |  |  |  |  |
| FGF16 |  |  |  |  |  |  |  |  |  |  |  |  |  |  |  |  |  |
| FBXO32 |  |  |  |  |  |  |  |  |  |  |  |  |  |  |  |  |  |
| CARS2 |  |  |  |  |  |  |  |  |  |  |  |  |  |  |  |  |  |
| PAPPA2 |  |  |  |  |  |  |  |  |  |  |  |  |  |  |  |  |  |
| NADSYN1 |  |  |  |  |  |  |  |  |  |  |  |  |  |  |  |  |  |
| GLRX5 |  |  |  |  |  |  |  |  |  |  |  |  |  |  |  |  |  |
| UBAC2 |  |  |  |  |  |  |  |  |  |  |  |  |  |  |  |  |  |
| KDM5C |  |  |  |  |  |  |  |  |  |  |  |  |  |  |  |  |  |
| DOCK8 |  |  |  |  |  |  |  |  |  |  |  |  |  |  |  |  |  |
| LOC112486223 |  |  |  |  |  |  |  |  |  |  |  |  |  |  |  |  |  |
| ASTN2 |  |  |  |  |  |  |  |  |  |  |  |  |  |  |  |  |  |
| LRP4 |  |  |  |  |  |  |  |  |  |  |  |  |  |  |  |  |  |
| SOCS3 |  |  |  |  |  |  |  |  |  |  |  |  |  |  |  |  |  |
| DDB1 |  |  |  |  |  |  |  |  |  |  |  |  |  |  |  |  |  |
| SIX5 |  |  |  |  |  |  |  |  |  |  |  |  |  |  |  |  |  |
| ADIPOR1 |  |  |  |  |  |  |  |  |  |  |  |  |  |  |  |  |  |
| ADAMTS19 |  |  |  |  |  |  |  |  |  |  |  |  |  |  |  |  |  |
| SMAD7 |  |  |  |  |  |  |  |  |  |  |  |  |  |  |  |  |  |
| RGS3 |  |  |  |  |  |  |  |  |  |  |  |  |  |  |  |  |  |
| RPL10 |  |  |  |  |  |  |  |  |  |  |  |  |  |  |  |  |  |
| EFNB2 |  |  |  |  |  |  |  |  |  |  |  |  |  |  |  |  |  |
| TASP1 |  |  |  |  |  |  |  |  |  |  |  |  |  |  |  |  |  |
| NAXE |  |  |  |  |  |  |  |  |  |  |  |  |  |  |  |  |  |
| MUC5B |  |  |  |  |  |  |  |  |  |  |  |  |  |  |  |  |  |
| EDARADD |  |  |  |  |  |  |  |  |  |  |  |  |  |  |  |  |  |
| FOXL1 |  |  |  |  |  |  |  |  |  |  |  |  |  |  |  |  |  |
| STX3 |  |  |  |  |  |  |  |  |  |  |  |  |  |  |  |  |  |
| MX1 |  |  |  |  |  |  |  |  |  |  |  |  |  |  |  |  |  |
| TYK2 |  |  |  |  |  |  |  |  |  |  |  |  |  |  |  |  |  |
| PAX4 |  |  |  |  |  |  |  |  |  |  |  |  |  |  |  |  |  |
| CNOT1 |  |  |  |  |  |  |  |  |  |  |  |  |  |  |  |  |  |
| ARHGAP24 |  |  |  |  |  |  |  |  |  |  |  |  |  |  |  |  |  |
| RXRA |  |  |  |  |  |  |  |  |  |  |  |  |  |  |  |  |  |
| TUFM |  |  |  |  |  |  |  |  |  |  |  |  |  |  |  |  |  |
| MIR23B |  |  |  |  |  |  |  |  |  |  |  |  |  |  |  |  |  |
| DDX59 |  |  |  |  |  |  |  |  |  |  |  |  |  |  |  |  |  |
| MIR125B1 |  |  |  |  |  |  |  |  |  |  |  |  |  |  |  |  |  |
| TNFSF13B |  |  |  |  |  |  |  |  |  |  |  |  |  |  |  |  |  |
| CSNK2A2 |  |  |  |  |  |  |  |  |  |  |  |  |  |  |  |  |  |
| CD2AP |  |  |  |  |  |  |  |  |  |  |  |  |  |  |  |  |  |
| PLA2G1B |  |  |  |  |  |  |  |  |  |  |  |  |  |  |  |  |  |
| PNP |  |  |  |  |  |  |  |  |  |  |  |  |  |  |  |  |  |
| ATP6V1B2 |  |  |  |  |  |  |  |  |  |  |  |  |  |  |  |  |  |
| ACTL6A |  |  |  |  |  |  |  |  |  |  |  |  |  |  |  |  |  |
| HPSE2 |  |  |  |  |  |  |  |  |  |  |  |  |  |  |  |  |  |
| SLC25A15 |  |  |  |  |  |  |  |  |  |  |  |  |  |  |  |  |  |
| AXIN2 |  |  |  |  |  |  |  |  |  |  |  |  |  |  |  |  |  |
| CLDN5 |  |  |  |  |  |  |  |  |  |  |  |  |  |  |  |  |  |
| ADD3 |  |  |  |  |  |  |  |  |  |  |  |  |  |  |  |  |  |
| SLC46A1 |  |  |  |  |  |  |  |  |  |  |  |  |  |  |  |  |  |
| ZNF423 |  |  |  |  |  |  |  |  |  |  |  |  |  |  |  |  |  |
| EHHADH |  |  |  |  |  |  |  |  |  |  |  |  |  |  |  |  |  |
| JAGN1 |  |  |  |  |  |  |  |  |  |  |  |  |  |  |  |  |  |
| WBP11 |  |  |  |  |  |  |  |  |  |  |  |  |  |  |  |  |  |
| NDP |  |  |  |  |  |  |  |  |  |  |  |  |  |  |  |  |  |
| FAM111A |  |  |  |  |  |  |  |  |  |  |  |  |  |  |  |  |  |
| CLPB |  |  |  |  |  |  |  |  |  |  |  |  |  |  |  |  |  |
| SNIP1 |  |  |  |  |  |  |  |  |  |  |  |  |  |  |  |  |  |
| NRAP |  |  |  |  |  |  |  |  |  |  |  |  |  |  |  |  |  |
| PUS1 |  |  |  |  |  |  |  |  |  |  |  |  |  |  |  |  |  |
| COL13A1 |  |  |  |  |  |  |  |  |  |  |  |  |  |  |  |  |  |
| NSMF |  |  |  |  |  |  |  |  |  |  |  |  |  |  |  |  |  |
| LMNB2 |  |  |  |  |  |  |  |  |  |  |  |  |  |  |  |  |  |
| VRK1 |  |  |  |  |  |  |  |  |  |  |  |  |  |  |  |  |  |
| DHDDS |  |  |  |  |  |  |  |  |  |  |  |  |  |  |  |  |  |
| CHRNA7 |  |  |  |  |  |  |  |  |  |  |  |  |  |  |  |  |  |
| P3H1 |  |  |  |  |  |  |  |  |  |  |  |  |  |  |  |  |  |
| ORC4 |  |  |  |  |  |  |  |  |  |  |  |  |  |  |  |  |  |
| KISS1 |  |  |  |  |  |  |  |  |  |  |  |  |  |  |  |  |  |
| ADAMTS9 |  |  |  |  |  |  |  |  |  |  |  |  |  |  |  |  |  |
| MTNR1B |  |  |  |  |  |  |  |  |  |  |  |  |  |  |  |  |  |
| ADAMTS3 |  |  |  |  |  |  |  |  |  |  |  |  |  |  |  |  |  |
| GOT2 |  |  |  |  |  |  |  |  |  |  |  |  |  |  |  |  |  |
| ABCC3 |  |  |  |  |  |  |  |  |  |  |  |  |  |  |  |  |  |
| USH2A |  |  |  |  |  |  |  |  |  |  |  |  |  |  |  |  |  |
| PDGFA |  |  |  |  |  |  |  |  |  |  |  |  |  |  |  |  |  |
| VDAC1 |  |  |  |  |  |  |  |  |  |  |  |  |  |  |  |  |  |
| EDA |  |  |  |  |  |  |  |  |  |  |  |  |  |  |  |  |  |
| ARAF |  |  |  |  |  |  |  |  |  |  |  |  |  |  |  |  |  |
| LHCGR |  |  |  |  |  |  |  |  |  |  |  |  |  |  |  |  |  |
| PAX5 |  |  |  |  |  |  |  |  |  |  |  |  |  |  |  |  |  |
| SMYD2 |  |  |  |  |  |  |  |  |  |  |  |  |  |  |  |  |  |
| PAEP |  |  |  |  |  |  |  |  |  |  |  |  |  |  |  |  |  |
| FGB |  |  |  |  |  |  |  |  |  |  |  |  |  |  |  |  |  |
| BLOC1S1 |  |  |  |  |  |  |  |  |  |  |  |  |  |  |  |  |  |
| LTF |  |  |  |  |  |  |  |  |  |  |  |  |  |  |  |  |  |
| ERMARD |  |  |  |  |  |  |  |  |  |  |  |  |  |  |  |  |  |
| AEBP1 |  |  |  |  |  |  |  |  |  |  |  |  |  |  |  |  |  |
| IFT88 |  |  |  |  |  |  |  |  |  |  |  |  |  |  |  |  |  |
| APC2 |  |  |  |  |  |  |  |  |  |  |  |  |  |  |  |  |  |
| CYP2A6 |  |  |  |  |  |  |  |  |  |  |  |  |  |  |  |  |  |
| LOC107548112 |  |  |  |  |  |  |  |  |  |  |  |  |  |  |  |  |  |
| PTDSS1 |  |  |  |  |  |  |  |  |  |  |  |  |  |  |  |  |  |
| RPL27 |  |  |  |  |  |  |  |  |  |  |  |  |  |  |  |  |  |
| TRIP13 |  |  |  |  |  |  |  |  |  |  |  |  |  |  |  |  |  |
| ARNT2 |  |  |  |  |  |  |  |  |  |  |  |  |  |  |  |  |  |
| CD80 |  |  |  |  |  |  |  |  |  |  |  |  |  |  |  |  |  |
| SLC26A2 |  |  |  |  |  |  |  |  |  |  |  |  |  |  |  |  |  |
| NLGN4X |  |  |  |  |  |  |  |  |  |  |  |  |  |  |  |  |  |
| PPP1CA |  |  |  |  |  |  |  |  |  |  |  |  |  |  |  |  |  |
| GLUD1 |  |  |  |  |  |  |  |  |  |  |  |  |  |  |  |  |  |
| USP7 |  |  |  |  |  |  |  |  |  |  |  |  |  |  |  |  |  |
| LFNG |  |  |  |  |  |  |  |  |  |  |  |  |  |  |  |  |  |
| CDAN1 |  |  |  |  |  |  |  |  |  |  |  |  |  |  |  |  |  |
| PRKCSH |  |  |  |  |  |  |  |  |  |  |  |  |  |  |  |  |  |
| PSMB4 |  |  |  |  |  |  |  |  |  |  |  |  |  |  |  |  |  |
| CHN1 |  |  |  |  |  |  |  |  |  |  |  |  |  |  |  |  |  |
| TAC3 |  |  |  |  |  |  |  |  |  |  |  |  |  |  |  |  |  |
| TSEN54 |  |  |  |  |  |  |  |  |  |  |  |  |  |  |  |  |  |
| UBE2A |  |  |  |  |  |  |  |  |  |  |  |  |  |  |  |  |  |
| RNU7-1 |  |  |  |  |  |  |  |  |  |  |  |  |  |  |  |  |  |
| LDHA |  |  |  |  |  |  |  |  |  |  |  |  |  |  |  |  |  |
| PNMT |  |  |  |  |  |  |  |  |  |  |  |  |  |  |  |  |  |
| DTNBP1 |  |  |  |  |  |  |  |  |  |  |  |  |  |  |  |  |  |
| TMOD1 |  |  |  |  |  |  |  |  |  |  |  |  |  |  |  |  |  |
| SRA1 |  |  |  |  |  |  |  |  |  |  |  |  |  |  |  |  |  |
| CMYA5 |  |  |  |  |  |  |  |  |  |  |  |  |  |  |  |  |  |
| MIR424 |  |  |  |  |  |  |  |  |  |  |  |  |  |  |  |  |  |
| GPX1 |  |  |  |  |  |  |  |  |  |  |  |  |  |  |  |  |  |
| RASGRP1 |  |  |  |  |  |  |  |  |  |  |  |  |  |  |  |  |  |
| TBX22 |  |  |  |  |  |  |  |  |  |  |  |  |  |  |  |  |  |
| SLC13A5 |  |  |  |  |  |  |  |  |  |  |  |  |  |  |  |  |  |
| TNIP1 |  |  |  |  |  |  |  |  |  |  |  |  |  |  |  |  |  |
| SERPINH1 |  |  |  |  |  |  |  |  |  |  |  |  |  |  |  |  |  |
| AIFM1 |  |  |  |  |  |  |  |  |  |  |  |  |  |  |  |  |  |
| FZD1 |  |  |  |  |  |  |  |  |  |  |  |  |  |  |  |  |  |
| ATP5MK |  |  |  |  |  |  |  |  |  |  |  |  |  |  |  |  |  |
| CPE |  |  |  |  |  |  |  |  |  |  |  |  |  |  |  |  |  |
| RAB7A |  |  |  |  |  |  |  |  |  |  |  |  |  |  |  |  |  |
| FNDC5 |  |  |  |  |  |  |  |  |  |  |  |  |  |  |  |  |  |
| NUP62 |  |  |  |  |  |  |  |  |  |  |  |  |  |  |  |  |  |
| CEBPA |  |  |  |  |  |  |  |  |  |  |  |  |  |  |  |  |  |
| CR1 |  |  |  |  |  |  |  |  |  |  |  |  |  |  |  |  |  |
| CHRNB1 |  |  |  |  |  |  |  |  |  |  |  |  |  |  |  |  |  |
| HLA-DPA1 |  |  |  |  |  |  |  |  |  |  |  |  |  |  |  |  |  |
| FOXN1 |  |  |  |  |  |  |  |  |  |  |  |  |  |  |  |  |  |
| SLC9A6 |  |  |  |  |  |  |  |  |  |  |  |  |  |  |  |  |  |
| SULT1A3 |  |  |  |  |  |  |  |  |  |  |  |  |  |  |  |  |  |
| MCCC2 |  |  |  |  |  |  |  |  |  |  |  |  |  |  |  |  |  |
| PPP1R1B |  |  |  |  |  |  |  |  |  |  |  |  |  |  |  |  |  |
| SMPX |  |  |  |  |  |  |  |  |  |  |  |  |  |  |  |  |  |
| SLC51A |  |  |  |  |  |  |  |  |  |  |  |  |  |  |  |  |  |
| TOP3A |  |  |  |  |  |  |  |  |  |  |  |  |  |  |  |  |  |
| DPF3 |  |  |  |  |  |  |  |  |  |  |  |  |  |  |  |  |  |
| DPM1 |  |  |  |  |  |  |  |  |  |  |  |  |  |  |  |  |  |
| NR1H2 |  |  |  |  |  |  |  |  |  |  |  |  |  |  |  |  |  |
| ITGB4 |  |  |  |  |  |  |  |  |  |  |  |  |  |  |  |  |  |
| G6PC1 |  |  |  |  |  |  |  |  |  |  |  |  |  |  |  |  |  |
| KCNJ10 |  |  |  |  |  |  |  |  |  |  |  |  |  |  |  |  |  |
| PXK |  |  |  |  |  |  |  |  |  |  |  |  |  |  |  |  |  |
| PNPLA3 |  |  |  |  |  |  |  |  |  |  |  |  |  |  |  |  |  |
| ACOX2 |  |  |  |  |  |  |  |  |  |  |  |  |  |  |  |  |  |
| PCK2 |  |  |  |  |  |  |  |  |  |  |  |  |  |  |  |  |  |

| Table S6. Overlapping genes between GXST target genes and Heart Failure-related genes. | | |
| --- | --- | --- |
| GXST | HF | GXST\|HF |
| CHRM1 | NKX2-5 | PTGS1 |
| HTR3A | TTN | CHRM3 |
| GABRA1 | GATA4 | SCN5A |
| NCOA1 | MYH6 | PTGS2 |
| IGHG1 | TBX5 | RXRA |
| PRSS1 | JAG1 | OPRD1 |
| TOP1 | NPPB | PDE3A |
| PCNA | LMNA | ADRA1A |
| MCL1 | MYH7 | SLC6A3 |
| TYR | NPPA | ADRB2 |
| TOP2A | GATA6 | OPRM1 |
| ADCY2 | MYBPC3 | SLC6A4 |
| CCNA2 | PRKAG2 | PGR |
| CHRNA2 | TTN-AS1 | NR3C2 |
| DRD5 | TNNT2 | ACHE |
| CYP1A2 | TBX20 | DRD2 |
| AKR1B10 | TNNI3 | ESR1 |
| POLB | TRPM4 | AR |
| PTPN2 | HAND2 | AKR1B1 |
| CDC25B | MYL2 | RELA |
| PTPRF | NODAL | EGFR |
| ACP1 | KCNQ1 | AKT1 |
| PTGES | ACTC1 | VEGFA |
| CES2 | DSP | CCND1 |
| PREP | BMPR2 | BCL2L1 |
| LTB4R | DMD | CDKN1A |
| FNTA | TBX1 | MMP2 |
| PTPN6 | FBN1 | MMP9 |
| CDC25A | GDF1 | MAPK1 |
| SERPINA6 | INS | IL10 |
| CYP51A1 | ZIC3 | RB1 |
| PTGER2 | RTEL1 | TNF |
| FABP5 | SMAD6 | JUN |
| FAAH | MIR21 | IL6 |
| PTGER1 | GJA5 | CASP3 |
| PTGIR | DES | TP53 |
| PTGDR2 | RYR2 | NFKBIA |
| AMPD2 | HAND1 | XDH |
| SIGMAR1 | PLN | MDM2 |
| PTGDR | FLNA | APP |
| PTGER3 | GATA5 | MMP1 |
| AURKB | DNAH1 | ERBB2 |
| AURKA | RYR1 | PPARG |
| PTGFR | AGT | HMOX1 |
| S1PR2 | ALB | CASP7 |
| ADAMTS5 | CDK8 | ICAM1 |
| RORA | TBX4 | BIRC5 |
| ITGB7 | COL2A1 | IL2 |
| PGGT1B | TPM1 | IFNG |
| ENPP2 | RBM20 | IL4 |
| CSF1R | ZFPM2 | GSTP1 |
| HCRTR1 | NKX2-6 | INSR |
| TAOK1 | NOTCH1 | CD40LG |
| MAPKAPK2 | KCNJ5 | MET |
| CASP6 | LDB3 | ADRA2A |
| PFKFB3 | CHD7 | NOS2 |
| CCNE2 | ENG | ESR2 |
| RPS6KA2 | APOE | GSK3B |
| MAPK11 | ELN | NR3C1 |
| ACACB | NBAS | KCNH2 |
| LIPG | NR2F2 | STAT3 |
| TNIK | CACNA1C | EDN1 |
| ZAP70 | FLNC | F7 |
| CCND3 | MAP2K1 | SLC6A2 |
| HDAC1 | TRMU | ADRA2B |
| FAP | KCNJ2 | BCL2 |
| PDE7A | APOA1 | FOS |
| HCK | RTEL1-TNFRSF6B | FASN |
| LYN | SCN1B | EDNRA |
| SCN9A | TAB2 | CYP3A4 |
| CDK2 | GLA | MYC |
| CDK1 | KRAS | CYP1A1 |
| MAP2 | CITED2 | NR1I2 |
| AHR | APOB | ECE1 |
| NR1I3 | ABCC9 | CALCR |
| GSTM2 | B3GAT3 | ITGB3 |
| AKR1C3 | BAG3 | CD81 |
| SREBF2 | HCN4 | PTPN1 |
| GLRA1 | DSG2 | HSD11B1 |
| SQLE | ERCC6 | RORC |
| FLT3 | BRF1 | PDE4D |
| CA7 | PIGL | PLA2G1B |
| HSD17B2 | CSRP3 | HMGCR |
| HSD17B1 | FMR1 | HSD11B2 |
| CA4 | PKP2 | FABP1 |
| GLO1 | ACTN2 | BACE1 |
| PTPRS | PARN | SHBG |
| CDK5R1 | MIR210 | G6PD |
| CCNB3 | ABCC8 | FABP4 |
| DAPK1 | HLA-DRB1 | PPARA |
| MPG | CD36 | FABP3 |
| TNKS2 | MTHFR | TERT |
| TNKS | MAP2K2 | PPARD |
| CDK6 | MYL3 | ALOX5 |
| CA1 | RPL5 | PTGER4 |
| PLK1 | PITX2 | PTPN11 |
| CA6 | MYPN | NR1H3 |
| CA14 | VWF | NPC1L1 |
| CA9 | KCNE1 | CYP17A1 |
| NEK2 | TAFAZZIN | MME |
| CAMK2B | CERS1 | AGTR1 |
| NEK6 | VCL | GPBAR1 |
| NUAK1 | PMS2 | MAPK3 |
| AKR1C2 | COL4A5 | MMP3 |
| AKR1C1 | PKD2 | EDNRB |
| AKR1C4 | TMEM43 | CASP8 |
| CA13 | CAV1 | MMP14 |
| AKR1A1 | IGF1 | PRKCH |
| CD38 | GAA | CTSA |
| AMY1A | SMAD4 | SCD |
| GRK6 | INPP5E | CNR1 |
| CTSL | LPA | ITGB1 |
| GRM2 | ANKRD1 | GLUL |
| CCNE1 | KCNE2 | FDFT1 |
| GABRB3 | RET | OXTR |
| GABRA2 | WT1 | CCR1 |
| GABRG2 | BRCA2 | REN |
| MAP3K8 | SGCD | P2RX3 |
| TRPA1 | CHDS2 | PIK3CA |
| GRM5 | CHDS3 | CASR |
| SLC2A3 | SOS1 | NR1H4 |
| PABPC1 | MIR499A | THRA |
| CTSV | FANCM | THRB |
| MTNR1A | PSEN1 | PIM1 |
| SLC6A9 | RERE | AMPD3 |
| PDE4C | ADM | ADORA1 |
| BCL2L2 | MHRT | ADORA2B |
| BCL2L10 | EPO | MMP13 |
| PTAFR | TLL1 | PDE10A |
| TDO2 | POLG | PORCN |
| GRM4 | CEP290 | PDGFRB |
| NMBR | BMP2 | FLT4 |
| IDO1 | FOXL2 | SYK |
| TBXAS1 | TERC | HCRTR2 |
| GCGR | CAV3 | SRC |
| ABAT | XRCC2 | NTRK1 |
| ACPP | HRAS | CRHR1 |
| APEX1 | MYL4 | NR5A1 |
| CA5B | LPL | GCK |
| CBR1 | LAMP2 | PTK2B |
| CDK4 | NOD2 | RPS6KA3 |
| CLK1 | POF1B | PDE5A |
| DYRK1B | MKS1 | KDR |
| EIF6 | MT-ND1 | PIK3CD |
| ESRRB | MT-CYB | PARP1 |
| FGR | MIR155 | MAPK14 |
| GOT1 | TCAP | PIK3R1 |
| GSTA1 | JUP | PIK3CG |
| GSTA2 | WDPCP | PTK2 |
| HSD17B14 | TGFB2 | KNG1 |
| HSD17B3 | ADIPOQ | CFD |
| KDM4E | BMP15 | PDPK1 |
| KLK1 | SURF1 | TGM2 |
| KLK2 | DTNA | TGM1 |
| NPEPPS | HADHA | F13A1 |
| NQO2 | SCARB2 | F10 |
| ODC1 | PKD1 | CTSK |
| PLA2G10 | GYS1 | MTOR |
| PLA2G5 | SMAD2 | HSP90AA1 |
| SIRT2 | NAGLU | CSNK2A1 |
| SOAT2 | DNAH8 | TRPV1 |
| TAS2R31 | CPT2 | FKBP1A |
| WEE1 | CHDS1 | JAK2 |
|  | HFE | BTK |
|  | ENPP1 | HDAC6 |
|  | STAG3 | FGFR2 |
|  | DNAJC21 | ROCK2 |
|  | HAMP | CAPN1 |
|  | RINT1 | SLC8A1 |
|  | MIR126 | FLT1 |
|  | MIR320A | KIT |
|  | HLA-B | ELANE |
|  | AFF4 | CFTR |
|  | GPT | ITGAL |
|  | FGF8 | PDE4A |
|  | PFHB2 | DPP4 |
|  | TRDN | TSPO |
|  | MT-TL1 | PDGFRA |
|  | CRELD1 | LCK |
|  | ANK2 | PIK3CB |
|  | CORIN | BDKRB2 |
|  | MIR17 | FGFR1 |
|  | B2M | ABCB1 |
|  | JPH2 | PRKCA |
|  | DIPK1A | TGFB1 |
|  | PTH1R | PON1 |
|  | ISL1 | IKBKB |
|  | SDHA | MAPK8 |
|  | CCN2 | SELE |
|  | MB | VCAM1 |
|  | MT-ATP6 | CYP1B1 |
|  | MKKS | PPP3CA |
|  | LGALS3 | GSTM1 |
|  | TSFM | PLAU |
|  | TLR4 | MAOB |
|  | LARS1 | MAOA |
|  | CHAT | ADRB1 |
|  | TGFBR2 | CYP19A1 |
|  | SLC17A5 | CYP2C19 |
|  | TGFBR1 | BCHE |
|  | ACTA1 | CHRM2 |
|  | MT-CO3 | VDR |
|  | TSC1 | NR1H2 |
|  | KCND3 | DHCR7 |
|  | HADHB | SHH |
|  | PHOX2B | UGT2B7 |
|  | ACE2 | NOX4 |
|  | MYH11 | CA2 |
|  | CLIC2 | ABCC1 |
|  | MED13L | CA12 |
|  | KCNJ11 | ESRRA |
|  | ACVRL1 | ABCG2 |
|  | WDR19 | ALOX15 |
|  | SERPINC1 | ALOX12 |
|  | NOBOX | ADORA2A |
|  | ACTA2 | ARG1 |
|  | TSC2 | GPR35 |
|  | NSD1 | SLC22A12 |
|  | F5 | TTR |
|  | PPA2 | AVPR2 |
|  | SARS2 | IGF1R |
|  | ATP2A2 | F2 |
|  | ERCC1 | DRD4 |
|  | CASQ2 | MPO |
|  | NPPC | PYGL |
|  | MECP2 | CA3 |
|  | TKT | PKN1 |
|  | PRDM16 | CXCR1 |
|  | CFAP47 | ALK |
|  | SDHB | CA5A |
|  | COL4A4 | AXL |
|  | MT-CO1 | MMP12 |
|  | PIK3C2A | MAPT |
|  | ABCA1 | PRKCD |
|  | EMD | CTSS |
|  | CFC1 | CTSB |
|  | MIR145 | ACE |
|  | CST3 | P2RX7 |
|  | GJD2-DT | ELOVL6 |
|  | MSH5 | KCNA5 |
|  | PMM2 | TNFRSF1A |
|  | BCS1L | SCN10A |
|  | DYNC2H1 | CPB1 |
|  | EVC2 | MEN1 |
|  | GRK2 | TRPC6 |
|  | PCSK9 | TRPC3 |
|  | TGFB3 | TRPV4 |
|  | ALDH2 | SLC2A1 |
|  | LEP | ADAM17 |
|  | CX3CR1 | CMA1 |
|  | FXN | SLC2A2 |
|  | MEFV | MMP7 |
|  | FGF23 | ABL1 |
|  | MEF2A | SCN2A |
|  | FOXC1 | KCNN4 |
|  | CRYAB | VCP |
|  | EYA4 | MTNR1B |
|  | CDK13 | PDE4B |
|  | FSHR | BAD |
|  | POLR1C | BCL2A1 |
|  | CDH2 | SCARB1 |
|  | PTCH1 | TACR3 |
|  | TAMM41 | ACACA |
|  | IFT172 | ADORA3 |
|  | MEF2C | BRAF |
|  | CLCN5 | CAT |
|  | MIR208A | CCL2 |
|  | SELENON | CES1 |
|  | TINF2 | CHEK2 |
|  | DZIP1 | CHRNA7 |
|  | FOXF1 | CHUK |
|  | CFH | COL1A1 |
|  | AARS2 | COL3A1 |
|  | HJV | CRP |
|  | SLC25A4 | CTSD |
|  | PRKD1 | CXCL10 |
|  | C3 | CXCL8 |
|  | CYP11B2 | CYP2C9 |
|  | MT-ND4 | DAO |
|  | MSH4 | DYRK1A |
|  | PRKAR1A | EGF |
|  | LZTR1 | EPHB4 |
|  | SOX9 | ERN1 |
|  | SMAD3 | F3 |
|  | CREBBP | GJA1 |
|  | SPATA16 | GSR |
|  | HBB | HIF1A |
|  | PLOD1 | HNF4A |
|  | LAMA2 | HSF1 |
|  | HP | HSPA1A |
|  | MYSM1 | HSPA5 |
|  | MT-ND5 | HSPB1 |
|  | IFT74 | IGFBP3 |
|  | IL18 | IL1A |
|  | CTLA4 | IL1B |
|  | TMEM67 | LDLR |
|  | PLXND1 | MGAM |
|  | SCN4B | MTTP |
|  | PSEN2 | MYLK |
|  | RBP4 | NCF1 |
|  | SLC22A5 | NOS3 |
|  | SELP | NQO1 |
|  | PTEN | PGF |
|  | MYOT | PLA2G2A |
|  | AGTR2 | PLAT |
|  | SHOC2 | PLK4 |
|  | HEG1 | POR |
|  | NIPBL | PRKCB |
|  | KCNJ8 | RAF1 |
|  | ACAD9 | SERPINE1 |
|  | MT-ND6 | SLC5A2 |
|  | SERPINA1 | SNCA |
|  | LCN2 | SOAT1 |
|  | ABCA3 | SOD1 |
|  | HFM1 | SREBF1 |
|  | SMARCA4 | SULT1E1 |
|  | CTNNA3 | TEK |
|  | IL1RN | THBD |
|  | SCN3B | TNNC1 |
|  | GDNF | YWHAG |
|  | CALCA |  |
|  | IGHMBP2 |  |
|  | KCNK3 |  |
|  | FOXH1 |  |
|  | SPEF2 |  |
|  | EVC |  |
|  | GATA1 |  |
|  | MT-CO2 |  |
|  | MCM8 |  |
|  | HEY2 |  |
|  | MIR92B |  |
|  | RASA1 |  |
|  | GNB3 |  |
|  | PPARGC1A |  |
|  | EP300 |  |
|  | KMT2D |  |
|  | CACNB2 |  |
|  | FN1 |  |
|  | GDF9 |  |
|  | FANCA |  |
|  | NEXN |  |
|  | FANCC |  |
|  | GBA1 |  |
|  | PEX6 |  |
|  | BMPR1A |  |
|  | CETP |  |
|  | ABCC6 |  |
|  | FLNC-AS1 |  |
|  | CEP19 |  |
|  | MIR140 |  |
|  | CDKN1C |  |
|  | HSPG2 |  |
|  | SPP1 |  |
|  | GLB1 |  |
|  | CP |  |
|  | COQ2 |  |
|  | SOHLH1 |  |
|  | FKTN |  |
|  | LCAT |  |
|  | POMC |  |
|  | ARID1B |  |
|  | KAT6B |  |
|  | SCN2B |  |
|  | DSC2 |  |
|  | MCM9 |  |
|  | SBDS |  |
|  | MYOCD |  |
|  | ADAMTS13 |  |
|  | H2AC18 |  |
|  | C4A |  |
|  | TF |  |
|  | SRP72 |  |
|  | BDNF |  |
|  | RIT1 |  |
|  | MT-ND3 |  |
|  | FOXP3 |  |
|  | IGF2 |  |
|  | SERPINA3 |  |
|  | EDN3 |  |
|  | HLA-DQB1 |  |
|  | LRRC56 |  |
|  | MIR328 |  |
|  | TBX3 |  |
|  | TMEM260 |  |
|  | CTF1 |  |
|  | BMP4 |  |
|  | TRIP4 |  |
|  | FGFR3 |  |
|  | SDHD |  |
|  | GDF15 |  |
|  | ATM |  |
|  | NOTCH2 |  |
|  | SCO2 |  |
|  | PON2 |  |
|  | TLR2 |  |
|  | TBX2 |  |
|  | SALL1 |  |
|  | DCHS1 |  |
|  | MED12 |  |
|  | NDUFS4 |  |
|  | PPP1CB |  |
|  | MYLK2 |  |
|  | PECAM1 |  |
|  | GHRL |  |
|  | APLNR |  |
|  | ERCC6L2 |  |
|  | MIR146A |  |
|  | CEP85L |  |
|  | CCR6 |  |
|  | TEX15 |  |
|  | MYRF |  |
|  | PAH |  |
|  | LTBP2 |  |
|  | IL17A |  |
|  | DNAJC19 |  |
|  | NF1 |  |
|  | MT-ND2 |  |
|  | GALT |  |
|  | SLC2A10 |  |
|  | PLCZ1 |  |
|  | NPHP1 |  |
|  | MBL2 |  |
|  | NRAS |  |
|  | STAT1 |  |
|  | GRK5 |  |
|  | LARS2 |  |
|  | NBN |  |
|  | NPY |  |
|  | EHMT1 |  |
|  | FHL1 |  |
|  | BRCA1 |  |
|  | AVP |  |
|  | SFTPB |  |
|  | ASPH |  |
|  | IFT140 |  |
|  | FRAXA |  |
|  | CFAP43 |  |
|  | DNAH10 |  |
|  | FCGR2A |  |
|  | ZMPSTE24 |  |
|  | CD34 |  |
|  | APOC3 |  |
|  | FAS |  |
|  | DIAPH2 |  |
|  | FOXC2 |  |
|  | ERCC4 |  |
|  | PRKG1 |  |
|  | SEMA3E |  |
|  | MGP |  |
|  | COL1A2 |  |
|  | ITGAM |  |
|  | LIPC |  |
|  | MMACHC |  |
|  | RAI1 |  |
|  | SLC4A1 |  |
|  | CASZ1 |  |
|  | CYP11A1 |  |
|  | DNAH5 |  |
|  | SOX17 |  |
|  | TET2 |  |
|  | CYCS |  |
|  | GNAQ |  |
|  | PRL |  |
|  | SMPD1 |  |
|  | COX5A |  |
|  | SOX4 |  |
|  | AKAP9 |  |
|  | APLN |  |
|  | TWNK |  |
|  | MPV17 |  |
|  | FANCI |  |
|  | SGCB |  |
|  | PACS1 |  |
|  | GLI3 |  |
|  | CC2D2A |  |
|  | HYLS1 |  |
|  | BCOR |  |
|  | CHGA |  |
|  | SLC34A1 |  |
|  | NPHP3 |  |
|  | MYH7B |  |
|  | TP63 |  |
|  | GBE1 |  |
|  | GLI2 |  |
|  | CTNNB1 |  |
|  | NDUFV1 |  |
|  | MYOZ2 |  |
|  | COX10 |  |
|  | FIGLA |  |
|  | LRP2 |  |
|  | SCO1 |  |
|  | SMG9 |  |
|  | LTA |  |
|  | ALMS1 |  |
|  | VPS33A |  |
|  | HNF1B |  |
|  | DACT1 |  |
|  | FIG4 |  |
|  | RBM10 |  |
|  | MIR196A2 |  |
|  | TNFSF11 |  |
|  | RPL36A-HNRNPH2 |  |
|  | RPGRIP1L |  |
|  | WDR35 |  |
|  | SEPTIN12 |  |
|  | EPAS1 |  |
|  | FKRP |  |
|  | ADA |  |
|  | FANCD2 |  |
|  | FGA |  |
|  | SFTPC |  |
|  | FGF2 |  |
|  | INVS |  |
|  | HLA-A |  |
|  | CACNA1D |  |
|  | CAMK2D |  |
|  | BRDT |  |
|  | AGXT |  |
|  | SLC40A1 |  |
|  | ANGPT2 |  |
|  | RAD51C |  |
|  | FBN2 |  |
|  | ZCCHC8 |  |
|  | CS |  |
|  | NEU1 |  |
|  | SMC1A |  |
|  | CSF3 |  |
|  | RRAS2 |  |
|  | NPC1 |  |
|  | MLXIPL |  |
|  | MEIOB |  |
|  | ASXL1 |  |
|  | HBA1 |  |
|  | GATAD1 |  |
|  | MRAS |  |
|  | FKBP6 |  |
|  | IDS |  |
|  | HSPB7 |  |
|  | SUN5 |  |
|  | GUSB |  |
|  | CALR |  |
|  | BAZ1B |  |
|  | PDX1 |  |
|  | SYCE1 |  |
|  | CBS |  |
|  | NUP155 |  |
|  | CFAP44 |  |
|  | SETBP1 |  |
|  | NEB |  |
|  | CFAP70 |  |
|  | MIR204 |  |
|  | COMT |  |
|  | LTBP4 |  |
|  | PTPN22 |  |
|  | SOS2 |  |
|  | KIF7 |  |
|  | TTC21B |  |
|  | C14orf39 |  |
|  | HLA-DQA1 |  |
|  | LOX |  |
|  | NDUFAF2 |  |
|  | ACTL9 |  |
|  | OLR1 |  |
|  | FLNB |  |
|  | PLG |  |
|  | LIPA |  |
|  | TIMP1 |  |
|  | FGF10 |  |
|  | SCNN1A |  |
|  | PEX5 |  |
|  | TRAF7 |  |
|  | NOS1 |  |
|  | SEMA3A |  |
|  | TNNI3K |  |
|  | BBS10 |  |
|  | ATP13A3 |  |
|  | GH1 |  |
|  | DPY19L2 |  |
|  | POLG2 |  |
|  | ASXL3 |  |
|  | FANCL |  |
|  | MIR423 |  |
|  | TRIM37 |  |
|  | FANCG |  |
|  | TK2 |  |
|  | SGSH |  |
|  | MTR |  |
|  | DNAH17 |  |
|  | NPR3 |  |
|  | FHOD3 |  |
|  | ZEB2 |  |
|  | MT-TW |  |
|  | LZTFL1 |  |
|  | GHR |  |
|  | HMGB1 |  |
|  | RRAD |  |
|  | PKHD1 |  |
|  | ADRA2C |  |
|  | MT-TK |  |
|  | MT-ATP8 |  |
|  | NPR1 |  |
|  | SMAD9 |  |
|  | SOD2 |  |
|  | PRODH |  |
|  | FOXP1 |  |
|  | SRF |  |
|  | OFD1 |  |
|  | TNFRSF11B |  |
|  | LOC110806306 |  |
|  | DNAH2 |  |
|  | RASA2 |  |
|  | IDUA |  |
|  | TEX11 |  |
|  | UTS2 |  |
|  | ACADVL |  |
|  | GTF2IRD1 |  |
|  | BBS2 |  |
|  | NEK1 |  |
|  | KLHL10 |  |
|  | IFIH1 |  |
|  | MAP3K7 |  |
|  | VHL |  |
|  | TFR2 |  |
|  | MIR199A1 |  |
|  | TALDO1 |  |
|  | CFAP58 |  |
|  | MT-TS1 |  |
|  | NOS1AP |  |
|  | RETN |  |
|  | BBS1 |  |
|  | ABCB11 |  |
|  | STX1A |  |
|  | MYH3 |  |
|  | QRICH2 |  |
|  | ARHGAP31 |  |
|  | PEX7 |  |
|  | PRF1 |  |
|  | NFATC1 |  |
|  | SLC25A13 |  |
|  | NDUFS2 |  |
|  | SOD3 |  |
|  | NFKB1 |  |
|  | NDUFB11 |  |
|  | NR0B1 |  |
|  | KCNIP2 |  |
|  | AGER |  |
|  | SLC26A8 |  |
|  | CACNA2D1 |  |
|  | IFT122 |  |
|  | DYSF |  |
|  | RPS19 |  |
|  | CYP3A5 |  |
|  | HGF |  |
|  | SLC9A1 |  |
|  | ARMC2 |  |
|  | CD46 |  |
|  | KCNQ1OT1 |  |
|  | GATM |  |
|  | ERBB4 |  |
|  | GNA11 |  |
|  | SON |  |
|  | ADCY10 |  |
|  | CFAP251 |  |
|  | B3GALT6 |  |
|  | MIF |  |
|  | KDM6A |  |
|  | BBS9 |  |
|  | PTH |  |
|  | LRP6 |  |
|  | COL4A3 |  |
|  | MCTP2 |  |
|  | RHOA |  |
|  | RNU4ATAC |  |
|  | RAC1 |  |
|  | FANCF |  |
|  | KMT2C |  |
|  | DLL1 |  |
|  | CALM1 |  |
|  | NFATC4 |  |
|  | CDKN2B-AS1 |  |
|  | ASAH1 |  |
|  | RAD51 |  |
|  | IL6ST |  |
|  | FAH |  |
|  | MEGF8 |  |
|  | RRM2B |  |
|  | TDRD9 |  |
|  | AQP2 |  |
|  | TMPO |  |
|  | ROBO1 |  |
|  | DYNC2I1 |  |
|  | MIR29A |  |
|  | IL1R1 |  |
|  | FOXRED1 |  |
|  | HADH |  |
|  | TLR7 |  |
|  | DNAI1 |  |
|  | PALB2 |  |
|  | HGSNAT |  |
|  | SMARCA2 |  |
|  | EPG5 |  |
|  | AGK |  |
|  | CELA2A |  |
|  | LOC110806263 |  |
|  | SMARCAL1 |  |
|  | GNPTAB |  |
|  | KL |  |
|  | ATP5F1A |  |
|  | SPAG17 |  |
|  | FKBP1B |  |
|  | UMOD |  |
|  | TBX18 |  |
|  | SETD5 |  |
|  | ACTB |  |
|  | FLCN |  |
|  | IL2RA |  |
|  | SST |  |
|  | APOA5 |  |
|  | PREPL |  |
|  | H19 |  |
|  | BCL7B |  |
|  | TCTN3 |  |
|  | SLC25A20 |  |
|  | PNPLA2 |  |
|  | NDUFA13 |  |
|  | MARS1 |  |
|  | TBL2 |  |
|  | RBCK1 |  |
|  | SALL4 |  |
|  | DNMT3A |  |
|  | RPS24 |  |
|  | FHL2 |  |
|  | KANSL1 |  |
|  | SEMA3C |  |
|  | GPD1L |  |
|  | DKC1 |  |
|  | BGLAP |  |
|  | NSD2 |  |
|  | BRIP1 |  |
|  | CD40 |  |
|  | HAND2-AS1 |  |
|  | F9 |  |
|  | NDUFS7 |  |
|  | DYNC2LI1 |  |
|  | PMFBP1 |  |
|  | TGIF1 |  |
|  | ETFDH |  |
|  | CFAP65 |  |
|  | LTBP1 |  |
|  | CHRND |  |
|  | ARSB |  |
|  | FASTKD2 |  |
|  | PGBD3 |  |
|  | COX15 |  |
|  | DBH |  |
|  | CLCNKB |  |
|  | SCN4A |  |
|  | NPR2 |  |
|  | MSH5-SAPCD1 |  |
|  | NFE2L2 |  |
|  | GTF2IRD2 |  |
|  | SLX4 |  |
|  | BUD23 |  |
|  | STAR |  |
|  | MPL |  |
|  | LIMK1 |  |
|  | PKD1L1 |  |
|  | IFT80 |  |
|  | RPL11 |  |
|  | MYOM2 |  |
|  | NDUFAF6 |  |
|  | NDUFAF5 |  |
|  | HSPD1 |  |
|  | ADD1 |  |
|  | SOX11 |  |
|  | MIR34A |  |
|  | SLC19A2 |  |
|  | SDCCAG8 |  |
|  | FANCE |  |
|  | GJB2 |  |
|  | CD4 |  |
|  | PNLDC1 |  |
|  | STRA6 |  |
|  | STAT4 |  |
|  | TIMP4 |  |
|  | MID1 |  |
|  | PAX2 |  |
|  | FRAS1 |  |
|  | NFIX |  |
|  | CHST3 |  |
|  | BNC1 |  |
|  | GJC1 |  |
|  | ARID1A |  |
|  | PTGIS |  |
|  | NPHS2 |  |
|  | KCNE3 |  |
|  | TTC21A |  |
|  | ADAMTSL2 |  |
|  | LMOD3 |  |
|  | CATSPER1 |  |
|  | PEX2 |  |
|  | DNAH9 |  |
|  | DNAH11 |  |
|  | HLA-DPB1 |  |
|  | GTF2I |  |
|  | PLEC |  |
|  | LIG4 |  |
|  | NDUFAF3 |  |
|  | MIR142 |  |
|  | BSCL2 |  |
|  | PLA2G7 |  |
|  | SLC25A26 |  |
|  | ABCG5 |  |
|  | STAG2 |  |
|  | SOX2 |  |
|  | DNAJC30 |  |
|  | AGPAT2 |  |
|  | SOX10 |  |
|  | PDHA2 |  |
|  | SCYL1 |  |
|  | GNRH1 |  |
|  | AMPD1 |  |
|  | UBE2T |  |
|  | TXNRD2 |  |
|  | TFAP2B |  |
|  | MSX1 |  |
|  | TTC29 |  |
|  | MT-TN |  |
|  | PF4 |  |
|  | PDE9A |  |
|  | RRAS |  |
|  | HIRA |  |
|  | NUP188 |  |
|  | TCF7L2 |  |
|  | BBS7 |  |
|  | GATA3 |  |
|  | TDGF1 |  |
|  | NRG1 |  |
|  | TNXB |  |
|  | SLC29A3 |  |
|  | CFAP69 |  |
|  | TFRC |  |
|  | POSTN |  |
|  | VKORC1 |  |
|  | FANCB |  |
|  | SNTA1 |  |
|  | BBIP1 |  |
|  | HBA2 |  |
|  | EPRS1 |  |
|  | ELAC2 |  |
|  | WFS1 |  |
|  | SMC3 |  |
|  | GLI1 |  |
|  | ANO5 |  |
|  | NONO |  |
|  | MTHFD1 |  |
|  | HDAC8 |  |
|  | ARVCF |  |
|  | BMP6 |  |
|  | MT-TE |  |
|  | FH |  |
|  | LOC102723566 |  |
|  | TTC8 |  |
|  | SYCP3 |  |
|  | NKX2-1 |  |
|  | CACNA1A |  |
|  | GALC |  |
|  | BBS4 |  |
|  | HBN1 |  |
|  | GSN |  |
|  | GAPDH |  |
|  | CCNH |  |
|  | PTPRC |  |
|  | SKIC2 |  |
|  | RFC2 |  |
|  | TMEM216 |  |
|  | GNB2 |  |
|  | MTX2 |  |
|  | M1AP |  |
|  | LOC107988032 |  |
|  | COG2 |  |
|  | CBL |  |
|  | CLIP2 |  |
|  | MT-TV |  |
|  | NPHS1 |  |
|  | IFT27 |  |
|  | CCDC40 |  |
|  | TNFSF4 |  |
|  | IL7 |  |
|  | TPI1 |  |
|  | TNNT1 |  |
|  | AOPEP |  |
|  | BMP10 |  |
|  | MYH9 |  |
|  | CSF2 |  |
|  | DGKE |  |
|  | VPS37D |  |
|  | MIR195 |  |
|  | MIR122 |  |
|  | MT-TI |  |
|  | TIMP2 |  |
|  | TPM3 |  |
|  | EIF4H |  |
|  | AURKC |  |
|  | MIR133A1 |  |
|  | CCL3 |  |
|  | ACADS |  |
|  | HNRNPA1 |  |
|  | PRDM6 |  |
|  | WRN |  |
|  | ERCC2 |  |
|  | CEP112 |  |
|  | HACD1 |  |
|  | VIP |  |
|  | DAZ1 |  |
|  | NLRP3 |  |
|  | ALPL |  |
|  | MIR223 |  |
|  | CDH13 |  |
|  | EFEMP2 |  |
|  | MIPEP |  |
|  | DISP1 |  |
|  | PPP1R13L |  |
|  | LBR |  |
|  | HLA-C |  |
|  | DGUOK |  |
|  | RBPJ |  |
|  | CUBN |  |
|  | CCL5 |  |
|  | CTC1 |  |
|  | GET1 |  |
|  | NPHP4 |  |
|  | LEPR |  |
|  | VIM |  |
|  | ADA2 |  |
|  | AK7 |  |
|  | APRT |  |
|  | ASCL1 |  |
|  | CHRNA1 |  |
|  | PSAP |  |
|  | MIB1 |  |
|  | LTBP3 |  |
|  | MIR182 |  |
|  | GAS1 |  |
|  | CAVIN1 |  |
|  | COX4I1 |  |
|  | COL11A2 |  |
|  | CXCL12 |  |
|  | MTRR |  |
|  | COX6B1 |  |
|  | KMT2A |  |
|  | MAD2L2 |  |
|  | APOA2 |  |
|  | CLCN1 |  |
|  | HTR2A |  |
|  | F12 |  |
|  | IL13 |  |
|  | NHLRC2 |  |
|  | ACVR2B |  |
|  | DLD |  |
|  | SPTB |  |
|  | HYDIN |  |
|  | CFB |  |
|  | GCNA |  |
|  | CCR5 |  |
|  | MIR22 |  |
|  | SQSTM1 |  |
|  | IFNA1 |  |
|  | KCNE5 |  |
|  | EIF2B2 |  |
|  | MYD88 |  |
|  | TMEM70 |  |
|  | KIAA0586 |  |
|  | CALM3 |  |
|  | PEX1 |  |
|  | GDF2 |  |
|  | AHI1 |  |
|  | XRCC4 |  |
|  | DOLK |  |
|  | NAA10 |  |
|  | MC2R |  |
|  | DNMT3B |  |
|  | NNT |  |
|  | SGO1 |  |
|  | CACNA1S |  |
|  | MDM4 |  |
|  | CAPN3 |  |
|  | ERCC8 |  |
|  | ATP1B1 |  |
|  | PPP2R3C |  |
|  | SCNN1B |  |
|  | PROKR2 |  |
|  | GCG |  |
|  | RPA1 |  |
|  | MIR378A |  |
|  | SIX3 |  |
|  | SOX3 |  |
|  | ATP8B1 |  |
|  | TFPI |  |
|  | KCNAB2 |  |
|  | IRF5 |  |
|  | FASLG |  |
|  | RREB1 |  |
|  | IRX4 |  |
|  | SETD2 |  |
|  | RAPGEF3 |  |
|  | GNAS |  |
|  | TRIM63 |  |
|  | TACO1 |  |
|  | TRIM32 |  |
|  | MVK |  |
|  | ZMYND15 |  |
|  | IKBKG |  |
|  | TERB1 |  |
|  | CDON |  |
|  | METTL27 |  |
|  | KIF1B |  |
|  | MOV10L1 |  |
|  | TWIST1 |  |
|  | AMHR2 |  |
|  | NDUFAF1 |  |
|  | ALG9 |  |
|  | HNRNPA2B1 |  |
|  | BBS5 |  |
|  | CPS1 |  |
|  | TFAM |  |
|  | DLL4 |  |
|  | FBXL4 |  |
|  | APOH |  |
|  | NDUFS1 |  |
|  | CATIP |  |
|  | ETS1 |  |
|  | DARS2 |  |
|  | IRS1 |  |
|  | MSTN |  |
|  | CTNS |  |
|  | RAPSN |  |
|  | C9orf72 |  |
|  | CXADR |  |
|  | ACADM |  |
|  | DSG2-AS1 |  |
|  | TPM2 |  |
|  | CTBP1 |  |
|  | MYL7 |  |
|  | SLC12A3 |  |
|  | COX8A |  |
|  | TLR5 |  |
|  | MIR143 |  |
|  | G6PC3 |  |
|  | ARID2 |  |
|  | NFU1 |  |
|  | CFAP53 |  |
|  | PEX26 |  |
|  | MMVP1 |  |
|  | THPO |  |
|  | NDUFA1 |  |
|  | DNAH8-AS1 |  |
|  | MTFMT |  |
|  | DNASE1L1 |  |
|  | GALNS |  |
|  | TMEM270 |  |
|  | NHP2 |  |
|  | SEMA4D |  |
|  | ZFPM2-AS1 |  |
|  | TH |  |
|  | ACVR1 |  |
|  | ALDH18A1 |  |
|  | NAMPT |  |
|  | SKI |  |
|  | BOLA3 |  |
|  | CEP120 |  |
|  | XYLT1 |  |
|  | ACTG2 |  |
|  | HEAT2 |  |
|  | GP1BB |  |
|  | CFAP91 |  |
|  | TCF4 |  |
|  | PHYH |  |
|  | PIGG |  |
|  | LEMD3 |  |
|  | CXCR4 |  |
|  | SYCP2 |  |
|  | RPL3L |  |
|  | PET100 |  |
|  | TSGA10 |  |
|  | WNT5A |  |
|  | DNAAF1 |  |
|  | SPINK2 |  |
|  | SUCLG1 |  |
|  | ENO2 |  |
|  | ATRX |  |
|  | HDAC4 |  |
|  | ZIC2 |  |
|  | DNAAF11 |  |
|  | DVL3 |  |
|  | SPEN |  |
|  | DYNC2I2 |  |
|  | AMH |  |
|  | GPC3 |  |
|  | WRAP53 |  |
|  | SNRPN |  |
|  | PURA |  |
|  | ODAD2 |  |
|  | PBX1 |  |
|  | SUFU |  |
|  | NANOS1 |  |
|  | LMOD2 |  |
|  | ITGA2B |  |
|  | ATP7B |  |
|  | FLI1 |  |
|  | IL33 |  |
|  | SEC24C |  |
|  | MIAT |  |
|  | MATR3 |  |
|  | TEX14 |  |
|  | BSG |  |
|  | NDE1 |  |
|  | MFN2 |  |
|  | RAB3GAP2 |  |
|  | PPIG |  |
|  | IFT43 |  |
|  | ANGPT1 |  |
|  | RPS10 |  |
|  | EYA1 |  |
|  | ATP1A2 |  |
|  | MYL1 |  |
|  | MYL9 |  |
|  | HCCS |  |
|  | CPT1A |  |
|  | BVES |  |
|  | MIR208B |  |
|  | ETV2 |  |
|  | BLK |  |
|  | IGFBP7 |  |
|  | COL6A1 |  |
|  | MTM1 |  |
|  | SCN1A |  |
|  | BGN |  |
|  | MCM4 |  |
|  | HTR2B |  |
|  | PIEZO2 |  |
|  | CCDC65 |  |
|  | GPX3 |  |
|  | MTO1 |  |
|  | RFWD3 |  |
|  | CYBA |  |
|  | PAX6 |  |
|  | MIR214 |  |
|  | RPL10L |  |
|  | FSIP2 |  |
|  | MUSK |  |
|  | PRTN3 |  |
|  | LGALS2 |  |
|  | UFD1 |  |
|  | CYP2D6 |  |
|  | TMEM126B |  |
|  | HMGA2 |  |
|  | OTUD6B |  |
|  | SMARCB1 |  |
|  | NUBPL |  |
|  | PUS3 |  |
|  | AFP |  |
|  | ITGA7 |  |
|  | YY1AP1 |  |
|  | PIGA |  |
|  | GUCY2C |  |
|  | CKM |  |
|  | ETFA |  |
|  | ETFB |  |
|  | HSF2BP |  |
|  | FBLN5 |  |
|  | DNAAF3 |  |
|  | LRPPRC |  |
|  | MIR23A |  |
|  | SPTBN1 |  |
|  | BBS12 |  |
|  | CELSR1 |  |
|  | USP9Y |  |
|  | LEPQTL1 |  |
|  | UCP2 |  |
|  | UQCRFS1 |  |
|  | GCLC |  |
|  | LAMA4 |  |
|  | PDPN |  |
|  | LDLRAP1 |  |
|  | MEG3 |  |
|  | DGCR8 |  |
|  | NEDD4L |  |
|  | PEX3 |  |
|  | NOTCH3 |  |
|  | SKIC3 |  |
|  | CD55 |  |
|  | ERBB3 |  |
|  | OPA1 |  |
|  | EIF2AK4 |  |
|  | MADD |  |
|  | HSD17B4 |  |
|  | HOTAIR |  |
|  | FTO |  |
|  | LRP8 |  |
|  | DLK1 |  |
|  | PAPPA |  |
|  | MT-TF |  |
|  | MYH8 |  |
|  | CCDC39 |  |
|  | TMEM237 |  |
|  | FREM2 |  |
|  | ATP6AP2 |  |
|  | SLC12A1 |  |
|  | AKR1D1 |  |
|  | GATA2 |  |
|  | CD19 |  |
|  | ABCG8 |  |
|  | PON3 |  |
|  | NEK10 |  |
|  | CLN3 |  |
|  | BMP7 |  |
|  | PEX13 |  |
|  | TNFAIP3 |  |
|  | UBE4B |  |
|  | KLHL40 |  |
|  | DCTN1 |  |
|  | ALPK3 |  |
|  | KIAA0753 |  |
|  | CHRNG |  |
|  | MEGF10 |  |
|  | KITLG |  |
|  | ABCB4 |  |
|  | PRDM9 |  |
|  | TRPM3 |  |
|  | DMPK |  |
|  | ZFHX3 |  |
|  | CALM2 |  |
|  | DNHD1 |  |
|  | PEX16 |  |
|  | TCOF1 |  |
|  | CYP24A1 |  |
|  | PIK3C2G |  |
|  | IGFBP1 |  |
|  | RPL26 |  |
|  | CRKL |  |
|  | HNF1A |  |
|  | SPRED2 |  |
|  | EFTUD2 |  |
|  | IL7R |  |
|  | SMN1 |  |
|  | ADCY5 |  |
|  | DOCK6 |  |
|  | LEFTY2 |  |
|  | CFI |  |
|  | NOP10 |  |
|  | BAX |  |
|  | CLDN16 |  |
|  | PICK1 |  |
|  | POU5F1 |  |
|  | FOXE3 |  |
|  | SAA1 |  |
|  | CASP9 |  |
|  | MMP21 |  |
|  | KCNH1 |  |
|  | GTPBP3 |  |
|  | MT-TQ |  |
|  | FBXO43 |  |
|  | SLC25A3 |  |
|  | RGS5 |  |
|  | ANK1 |  |
|  | COX7B |  |
|  | CARD14 |  |
|  | PLCE1 |  |
|  | LRP5 |  |
|  | FGF21 |  |
|  | AHSG |  |
|  | ATP6V0A2 |  |
|  | MAX |  |
|  | MYOD1 |  |
|  | XYLT2 |  |
|  | APELA |  |
|  | PTHLH |  |
|  | INSL6 |  |
|  | LMX1B |  |
|  | SCNN1G |  |
|  | CYP27B1 |  |
|  | SDHAF1 |  |
|  | CCL11 |  |
|  | TCTN2 |  |
|  | CRH |  |
|  | TRIM21 |  |
|  | SMO |  |
|  | DSCAM |  |
|  | DAZL |  |
|  | TRPM6 |  |
|  | NDUFS8 |  |
|  | CD79A |  |
|  | RARB |  |
|  | GLE1 |  |
|  | GNE |  |
|  | PTF1A |  |
|  | LOC107133510 |  |
|  | SIRT1 |  |
|  | RPGR |  |
|  | UBE3B |  |
|  | HCFC1 |  |
|  | MYCN |  |
|  | UQCRB |  |
|  | DNAAF4 |  |
|  | MIR124-1 |  |
|  | PEX10 |  |
|  | BSND |  |
|  | TMEM231 |  |
|  | SLC12A2 |  |
|  | PEX12 |  |
|  | RAD21 |  |
|  | SPOP |  |
|  | GYG1 |  |
|  | TRPS1 |  |
|  | PUF60 |  |
|  | SLC2A4 |  |
|  | ANOS1 |  |
|  | CFHR5 |  |
|  | COX6A2 |  |
|  | FGD1 |  |
|  | CCDC141 |  |
|  | STN1 |  |
|  | SPECC1L |  |
|  | CLCN7 |  |
|  | TAC1 |  |
|  | B4GALT7 |  |
|  | MIR144 |  |
|  | PLAGL1 |  |
|  | MEIS2 |  |
|  | FCN3 |  |
|  | NDUFV2 |  |
|  | NEK8 |  |
|  | IL2RB |  |
|  | MESP1 |  |
|  | MT-TS2 |  |
|  | BCR |  |
|  | CSPP1 |  |
|  | AGL |  |
|  | SIRT6 |  |
|  | MLYCD |  |
|  | GCLM |  |
|  | IL6R |  |
|  | ACD |  |
|  | MMUT |  |
|  | CR2 |  |
|  | ZPBP |  |
|  | PIGO |  |
|  | ANKRD11 |  |
|  | CYC1 |  |
|  | FLAD1 |  |
|  | HES7 |  |
|  | BRD4 |  |
|  | SLC2A9 |  |
|  | DNAJB11 |  |
|  | CDKN2A |  |
|  | KCNJ1 |  |
|  | LOC110121269 |  |
|  | PEX19 |  |
|  | NDUFB3 |  |
|  | PSMA6 |  |
|  | TOM1 |  |
|  | RPS20 |  |
|  | INTU |  |
|  | HNRNPK |  |
|  | COL5A2 |  |
|  | FOXJ1 |  |
|  | CEP164 |  |
|  | TMEM94 |  |
|  | CIITA |  |
|  | SETX |  |
|  | ACTN4 |  |
|  | SMARCE1 |  |
|  | HESX1 |  |
|  | KCND2 |  |
|  | THBS1 |  |
|  | MASP2 |  |
|  | HBG2 |  |
|  | BANF1 |  |
|  | ODAD3 |  |
|  | PHOX2B-AS1 |  |
|  | DNASE1 |  |
|  | NDUFA11 |  |
|  | TNFRSF1B |  |
|  | EFL1 |  |
|  | DNM1L |  |
|  | PEX14 |  |
|  | MIR24-1 |  |
|  | SPIDR |  |
|  | NEBL |  |
|  | SH3PXD2B |  |
|  | RPL15 |  |
|  | HYAL2 |  |
|  | COL5A1 |  |
|  | SNAP29 |  |
|  | MDH2 |  |
|  | ATXN2 |  |
|  | CLCNKA |  |
|  | ADNP |  |
|  | HYMAI |  |
|  | IL12B |  |
|  | LMOD1 |  |
|  | GGT1 |  |
|  | NDUFS6 |  |
|  | MAP1B |  |
|  | FCGR3B |  |
|  | SLCO2A1 |  |
|  | PLAUR |  |
|  | LAMB2 |  |
|  | CX3CL1 |  |
|  | AUTS2 |  |
|  | TECRL |  |
|  | ALG8 |  |
|  | LETM1 |  |
|  | DNAJB13 |  |
|  | ADRB3 |  |
|  | IFNB1 |  |
|  | CYBB |  |
|  | TNFRSF11A |  |
|  | PTX3 |  |
|  | NDUFS3 |  |
|  | DCN |  |
|  | TCIRG1 |  |
|  | NELFA |  |
|  | SGCG |  |
|  | RAG1 |  |
|  | PCCA |  |
|  | LOC110011216 |  |
|  | RPS26 |  |
|  | RSPH4A |  |
|  | BAAT |  |
|  | TAF4B |  |
|  | C2CD6 |  |
|  | DNMT1 |  |
|  | PQBP1 |  |
|  | GFM1 |  |
|  | LOC106099062 |  |
|  | PDCD1 |  |
|  | TG |  |
|  | C4B |  |
|  | NDUFB8 |  |
|  | MT-TH |  |
|  | B9D1 |  |
|  | DSE |  |
|  | HMGCL |  |
|  | EIF2AK3 |  |
|  | HPDL |  |
|  | DNAI2 |  |
|  | DHCR24 |  |
|  | MT-TG |  |
|  | TNFSF12 |  |
|  | LMBRD1 |  |
|  | SPTAN1 |  |
|  | NEK9 |  |
|  | RMRP |  |
|  | CYP21A2 |  |
|  | MAFB |  |
|  | PYGM |  |
|  | ZSWIM7 |  |
|  | RNLS |  |
|  | MC4R |  |
|  | PLD1 |  |
|  | OCRL |  |
|  | MIR590 |  |
|  | MIR150 |  |
|  | ATXN7 |  |
|  | SH2B1 |  |
|  | PRKCZ |  |
|  | JARID2 |  |
|  | KIF6 |  |
|  | GUCY1A1 |  |
|  | HSPA4 |  |
|  | S100A1 |  |
|  | INHA |  |
|  | MIR20A |  |
|  | FUCA1 |  |
|  | MYOZ1 |  |
|  | CALCRL |  |
|  | IFT81 |  |
|  | NDUFA6 |  |
|  | UQCRC2 |  |
|  | TJP2 |  |
|  | PAFAH1B1 |  |
|  | XK |  |
|  | IRAK1 |  |
|  | TTC26 |  |
|  | AHCY |  |
|  | RNF212 |  |
|  | ZNF469 |  |
|  | ABCD4 |  |
|  | TREX1 |  |
|  | SLC39A8 |  |
|  | VANGL1 |  |
|  | SGO2 |  |
|  | MESD |  |
|  | CHST14 |  |
|  | ALG1 |  |
|  | NDUFB9 |  |
|  | HSD3B7 |  |
|  | S100B |  |
|  | MT-TT |  |
|  | TRPM7 |  |
|  | SPRED1 |  |
|  | ACTG1 |  |
|  | COL4A1 |  |
|  | SLC4A3 |  |
|  | KASH5 |  |
|  | AHDC1 |  |
|  | CD8A |  |
|  | TCN2 |  |
|  | TXNL4A |  |
|  | DRC1 |  |
|  | DPF2 |  |
|  | CDH23 |  |
|  | MAP3K20 |  |
|  | RUNX2 |  |
|  | ROR2 |  |
|  | ADAMTS10 |  |
|  | APOL1 |  |
|  | OTX2 |  |
|  | LRRC37A2 |  |
|  | VPS33B |  |
|  | PPBP |  |
|  | HFE-AS1 |  |
|  | ZFP57 |  |
|  | VEGFC |  |
|  | PKD2L2-DT |  |
|  | MAPK10 |  |
|  | MMP23B |  |
|  | TYMP |  |
|  | STIL |  |
|  | ODAD4 |  |
|  | WNT4 |  |
|  | HRC |  |
|  | ESCO2 |  |
|  | IQCB1 |  |
|  | SLC27A6 |  |
|  | B9D2 |  |
|  | DNM2 |  |
|  | RELN |  |
|  | UBR1 |  |
|  | TAPVR1 |  |
|  | VIPAS39 |  |
|  | MIR1-2 |  |
|  | OXT |  |
|  | GFAP |  |
|  | JMJD1C |  |
|  | VANGL2 |  |
|  | POPDC2 |  |
|  | MUC1 |  |
|  | IL1RL1 |  |
|  | LUZP1 |  |
|  | ADAMTSL1 |  |
|  | PCCB |  |
|  | SMARCC2 |  |
|  | RCAN1 |  |
|  | SDHC |  |
|  | PDSS2 |  |
|  | DVL1 |  |
|  | GOPC |  |
|  | COA3 |  |
|  | PEX11B |  |
|  | COA8 |  |
|  | CCDC62 |  |
|  | ATP7A |  |
|  | CAMK2G |  |
|  | POGZ |  |
|  | SELL |  |
|  | SLC9A3 |  |
|  | CHD4 |  |
|  | EDN2 |  |
|  | VAC14 |  |
|  | LMNB1 |  |
|  | RECQL4 |  |
|  | SMARCD1 |  |
|  | KYNU |  |
|  | PDLIM5 |  |
|  | MSX2 |  |
|  | OBSCN |  |
|  | ALG12 |  |
|  | MYH4 |  |
|  | SRP54 |  |
|  | CD14 |  |
|  | ANXA5 |  |
|  | ABCD1 |  |
|  | BMPR1B |  |
|  | CASP1 |  |
|  | COG1 |  |
|  | ALDH1A2 |  |
|  | FUZ |  |
|  | KDM4C |  |
|  | B3GLCT |  |
|  | IMMT |  |
|  | CASK |  |
|  | AMN |  |
|  | TERB2 |  |
|  | NEUROD1 |  |
|  | PNPLA8 |  |
|  | BLM |  |
|  | RPL35A |  |
|  | TMPRSS15 |  |
|  | KCNQ1-AS1 |  |
|  | ARL6 |  |
|  | COQ4 |  |
|  | GPC4 |  |
|  | CPLANE1 |  |
|  | NPM1 |  |
|  | CYP2J2 |  |
|  | MTPN |  |
|  | LOC110006319 |  |
|  | GNRHR |  |
|  | THBS4 |  |
|  | PDGFB |  |
|  | TRAF3IP1 |  |
|  | LEMD2 |  |
|  | TMEM127 |  |
|  | PRKACA |  |
|  | CFHR1 |  |
|  | RANGRF |  |
|  | IBA57 |  |
|  | DDC |  |
|  | INPPL1 |  |
|  | RSPH3 |  |
|  | LOC119369037 |  |
|  | CRYAA |  |
|  | ROBO4 |  |
|  | COG7 |  |
|  | CHCHD10 |  |
|  | PRR12 |  |
|  | HPGD |  |
|  | KIF20A |  |
|  | PDHA1 |  |
|  | MPI |  |
|  | LOC113939944 |  |
|  | SDHAF2 |  |
|  | SLC4A4 |  |
|  | AMACR |  |
|  | DAG1 |  |
|  | EPOR |  |
|  | IL15 |  |
|  | FOXP2 |  |
|  | SAR1B |  |
|  | FURIN |  |
|  | YY1 |  |
|  | SERAC1 |  |
|  | CELF2 |  |
|  | IGF2R |  |
|  | LOC113687175 |  |
|  | RAMP2 |  |
|  | NFS1 |  |
|  | TRMT10C |  |
|  | MAGEL2 |  |
|  | MST1 |  |
|  | ADK |  |
|  | CPLX1 |  |
|  | SRY |  |
|  | TMCO1 |  |
|  | APOA4 |  |
|  | RYR3 |  |
|  | UCN |  |
|  | PIGV |  |
|  | CASQ1 |  |
|  | TUBB |  |
|  | BCKDHB |  |
|  | CCDC103 |  |
|  | COL18A1 |  |
|  | YWHAE |  |
|  | ODAD1 |  |
|  | RSPO2 |  |
|  | GABRD |  |
|  | NDUFC2 |  |
|  | ARFGEF2 |  |
|  | NDUFAF4 |  |
|  | CCT7 |  |
|  | HPRT1 |  |
|  | MEOX1 |  |
|  | WASHC5 |  |
|  | YARS2 |  |
|  | SOX18 |  |
|  | BAG5 |  |
|  | IQSEC2 |  |
|  | TSR2 |  |
|  | TGDS |  |
|  | SYNE1 |  |
|  | RAD54L |  |
|  | SGCA |  |
|  | MYBPC1 |  |
|  | NUP107 |  |
|  | FGF1 |  |
|  | EPM2A |  |
|  | CYP27A1 |  |
|  | CCR2 |  |
|  | PGM1 |  |
|  | RPS17 |  |
|  | ATRIP |  |
|  | AKT3 |  |
|  | MIR29B1 |  |
|  | SPAG1 |  |
|  | DHFR |  |
|  | ATP6V1A |  |
|  | NAGA |  |
|  | WWOX |  |
|  | ERCC5 |  |
|  | PPCS |  |
|  | CXCL9 |  |
|  | MICOS13 |  |
|  | GAS2L2 |  |
|  | FECH |  |
|  | SLC7A7 |  |
|  | TCTN1 |  |
|  | PCK1 |  |
|  | JAK3 |  |
|  | MMAA |  |
|  | C12orf60 |  |
|  | DDX3Y |  |
|  | GP1BA |  |
|  | PSMD12 |  |
|  | F8 |  |
|  | HS6ST1 |  |
|  | ARX |  |
|  | SYNE2 |  |
|  | RTL1 |  |
|  | PLA2G6 |  |
|  | TARDBP |  |
|  | CFAP298 |  |
|  | CTCF |  |
|  | FAN1 |  |
|  | GDF6 |  |
|  | KCNMA1 |  |
|  | FAT4 |  |
|  | CMH21 |  |
|  | POLR3A |  |
|  | CHRNE |  |
|  | SMCHD1 |  |
|  | IARS2 |  |
|  | TNNI1 |  |
|  | REC8 |  |
|  | NSMCE2 |  |
|  | DDX11 |  |
|  | CLU |  |
|  | HOXD13 |  |
|  | OSTM1 |  |
|  | PGAP3 |  |
|  | MLX |  |
|  | P2RY12 |  |
|  | CCDC22 |  |
|  | SCN8A |  |
|  | HSPA8 |  |
|  | MYOG |  |
|  | CD68 |  |
|  | TIMMDC1 |  |
|  | MFAP5 |  |
|  | RAG2 |  |
|  | ERCC3 |  |
|  | VPS13B |  |
|  | COX16 |  |
|  | NDUFAF8 |  |
|  | U2AF1 |  |
|  | ZNF462 |  |
|  | TLR3 |  |
|  | SF3B4 |  |
|  | NGF |  |
|  | COX4I2 |  |
|  | ASCC1 |  |
|  | HGD |  |
|  | PSMB8 |  |
|  | SPEG |  |
|  | APOA1-AS |  |
|  | MRAP |  |
|  | CUL3 |  |
|  | ADH5 |  |
|  | GAREM2 |  |
|  | MRPS34 |  |
|  | DLAT |  |
|  | RPGRIP1 |  |
|  | SFTPD |  |
|  | ALG2 |  |
|  | DYNLT2B |  |
|  | COQ9 |  |
|  | OTC |  |
|  | POLA1 |  |
|  | UBE3A |  |
|  | ATP2B1 |  |
|  | GAS8 |  |
|  | HCN2 |  |
|  | SUCLA2 |  |
|  | IKZF1 |  |
|  | GRIP1 |  |
|  | PIBF1 |  |
|  | TPO |  |
|  | USP9X |  |
|  | HDAC9 |  |
|  | TXN |  |
|  | DCDC2 |  |
|  | SLC3A1 |  |
|  | NPC2 |  |
|  | P4HB |  |
|  | ZMIZ1 |  |
|  | HIBCH |  |
|  | ABCC2 |  |
|  | EMG1 |  |
|  | BTNL2 |  |
|  | CYP11B1 |  |
|  | LRRC10 |  |
|  | MESP2 |  |
|  | CFAP92 |  |
|  | CHD3 |  |
|  | RSPH9 |  |
|  | PNPLA6 |  |
|  | MIR132 |  |
|  | ATAD1 |  |
|  | POMT1 |  |
|  | SCGB1A1 |  |
|  | AKAP13 |  |
|  | PCNT |  |
|  | LAMA3 |  |
|  | SMN2 |  |
|  | TXNDC15 |  |
|  | NALCN |  |
|  | ANKS6 |  |
|  | SI |  |
|  | AFG3L2 |  |
|  | TTC12 |  |
|  | CFAP300 |  |
|  | IFNA2 |  |
|  | ACAD8 |  |
|  | HLA-G |  |
|  | CACNA1B |  |
|  | PROK2 |  |
|  | MRPL44 |  |
|  | NDUFB10 |  |
|  | DNAL1 |  |
|  | COG4 |  |
|  | PHEX |  |
|  | RAB23 |  |
|  | ECHS1 |  |
|  | GALNT11 |  |
|  | C2CD3 |  |
|  | PI4KA |  |
|  | UQCRQ |  |
|  | CCBE1 |  |
|  | IRX5 |  |
|  | IFT52 |  |
|  | HSPB2 |  |
|  | APC |  |
|  | PRKCE |  |
|  | LONP1 |  |
|  | EOGT |  |
|  | TNFRSF13B |  |
|  | DKK1 |  |
|  | PDHX |  |
|  | SRCAP |  |
|  | SLPI |  |
|  | MUC16 |  |
|  | TRMT5 |  |
|  | FLRT3 |  |
|  | DOK7 |  |
|  | POLR1A |  |
|  | COG6 |  |
|  | CFHR3 |  |
|  | BICRA |  |
|  | ATP1A1 |  |
|  | GCDH |  |
|  | HAVCR1 |  |
|  | TTC7A |  |
|  | DZIP1L |  |
|  | CEP57 |  |
|  | SLC26A1 |  |
|  | NARS2 |  |
|  | ACVR1B |  |
|  | SEMA5A |  |
|  | PLEKHM2 |  |
|  | LPIN1 |  |
|  | ITGA8 |  |
|  | GNB1 |  |
|  | MRPL3 |  |
|  | HEXB |  |
|  | MT-TL2 |  |
|  | TDRKH |  |
|  | DUSP1 |  |
|  | COQ8B |  |
|  | ZNF276 |  |
|  | CFAP418 |  |
|  | MIR30A |  |
|  | EPCAM |  |
|  | FGF12 |  |
|  | AFF2 |  |
|  | CSF1 |  |
|  | DNAAF5 |  |
|  | XIAP |  |
|  | RNASE3 |  |
|  | CD163 |  |
|  | CCDC115 |  |
|  | SPTA1 |  |
|  | TAGLN |  |
|  | PPP2R2B |  |
|  | CHKB |  |
|  | RSPH1 |  |
|  | AUH |  |
|  | ATP6AP1 |  |
|  | CDKN3 |  |
|  | EZH2 |  |
|  | LOC110121486 |  |
|  | MYOM1 |  |
|  | LOC106627981 |  |
|  | PRICKLE1 |  |
|  | EBP |  |
|  | TMPRSS2 |  |
|  | SLC34A2 |  |
|  | L1CAM |  |
|  | FBLN2 |  |
|  | DUSP6 |  |
|  | KCNJ6 |  |
|  | NME8 |  |
|  | MAF |  |
|  | KDM5D |  |
|  | HBEGF |  |
|  | TTC19 |  |
|  | SHOX2 |  |
|  | PRKN |  |
|  | RLN2 |  |
|  | NDUFA12 |  |
|  | DNAAF2 |  |
|  | RMND1 |  |
|  | RELB |  |
|  | PHGDH |  |
|  | DMC1 |  |
|  | LOC106050102 |  |
|  | DAXX |  |
|  | LIPE |  |
|  | IGFBP2 |  |
|  | AGGF1 |  |
|  | KCNA7 |  |
|  | LHX4 |  |
|  | IFNGR1 |  |
|  | SLC26A3 |  |
|  | CCNO |  |
|  | SORT1 |  |
|  | AKT2 |  |
|  | PRG4 |  |
|  | DMRT1 |  |
|  | IRF4 |  |
|  | IL3 |  |
|  | POLR1D |  |
|  | NHLRC1 |  |
|  | NDUFA4 |  |
|  | TAF1 |  |
|  | RPS4Y2 |  |
|  | XIRP1 |  |
|  | TRIM55 |  |
|  | DYNC1H1 |  |
|  | NDUFA8 |  |
|  | SSB |  |
|  | SLC1A2 |  |
|  | PRNP |  |
|  | MAP3K5 |  |
|  | SATB2 |  |
|  | GZMB |  |
|  | DGCR2 |  |
|  | MIR27A |  |
|  | MMADHC |  |
|  | CHKA |  |
|  | IL17RD |  |
|  | EPX |  |
|  | DCAF8 |  |
|  | FZD4 |  |
|  | ALOX5AP |  |
|  | LYZ |  |
|  | FABP2 |  |
|  | CTNND2 |  |
|  | SHOX |  |
|  | IL18BP |  |
|  | LRP1 |  |
|  | STAT5B |  |
|  | ANKRD31 |  |
|  | EXT2 |  |
|  | COL7A1 |  |
|  | NPHP3-ACAD11 |  |
|  | PGM3 |  |
|  | HAX1 |  |
|  | COX7A1 |  |
|  | CFAP410 |  |
|  | LACTB |  |
|  | GLS |  |
|  | MT-RNR1 |  |
|  | FLII |  |
|  | DMP1 |  |
|  | EDAR |  |
|  | NCAM1 |  |
|  | AMER1 |  |
|  | CDC42 |  |
|  | STK36 |  |
|  | MRPS7 |  |
|  | PIGT |  |
|  | GJA4 |  |
|  | APBB1 |  |
|  | CAPN15 |  |
|  | TBC1D4 |  |
|  | UGT1A1 |  |
|  | SLC37A4 |  |
|  | CFAP221 |  |
|  | LARS2-AS1 |  |
|  | MYH10 |  |
|  | CDKL5 |  |
|  | RPS29 |  |
|  | STXBP1 |  |
|  | WNT9B |  |
|  | TMEM107 |  |
|  | FOXO3 |  |
|  | FENDRR |  |
|  | COG8 |  |
|  | F2R |  |
|  | ABCB7 |  |
|  | WDR11 |  |
|  | WNT11 |  |
|  | SAMD9 |  |
|  | GHRHR |  |
|  | ALAD |  |
|  | AARS1 |  |
|  | CKB |  |
|  | RUNX1 |  |
|  | CREB1 |  |
|  | FCGR2B |  |
|  | GNB5 |  |
|  | MIR221 |  |
|  | GLIS2 |  |
|  | MYH1 |  |
|  | IL12A |  |
|  | ENO1 |  |
|  | PIGQ |  |
|  | MAT2A |  |
|  | NFATC3 |  |
|  | NDUFA9 |  |
|  | CIROP |  |
|  | THOC6 |  |
|  | SNAP25 |  |
|  | NDUFA10 |  |
|  | PPP1R3A |  |
|  | ABCA12 |  |
|  | PRKAR1B |  |
|  | MASP1 |  |
|  | ADAR |  |
|  | MYLK3 |  |
|  | KCNA1 |  |
|  | LIF |  |
|  | KLHL7 |  |
|  | SUMF1 |  |
|  | AMBP |  |
|  | KRT19 |  |
|  | MBNL1 |  |
|  | LYRM7 |  |
|  | HYT1 |  |
|  | HYT2 |  |
|  | HYT3 |  |
|  | HYT4 |  |
|  | HYT5 |  |
|  | HYT6 |  |
|  | HYT7 |  |
|  | HYT8 |  |
|  | FSIP2-AS1 |  |
|  | RBM24 |  |
|  | PTGDS |  |
|  | HEY1 |  |
|  | DCLRE1C |  |
|  | FZD2 |  |
|  | NTRK3 |  |
|  | TBR1 |  |
|  | IGHE |  |
|  | SNX10 |  |
|  | TBC1D24 |  |
|  | TMPPE |  |
|  | LRBA |  |
|  | GRM7 |  |
|  | ELP1 |  |
|  | ISCA2 |  |
|  | ZNF699 |  |
|  | TYMS |  |
|  | UMPS |  |
|  | FTH1 |  |
|  | ATP5F1E |  |
|  | IRS2 |  |
|  | ITGA2 |  |
|  | FCN2 |  |
|  | MIR1-1 |  |
|  | GRHPR |  |
|  | CARS1 |  |
|  | HNRNPUL2-BSCL2 |  |
|  | SPINK5 |  |
|  | ZRSR2 |  |
|  | TBK1 |  |
|  | SEC23B |  |
|  | TSHR |  |
|  | PLXNA2 |  |
|  | WNT3 |  |
|  | C1S |  |
|  | TSPYL1 |  |
|  | MYMK |  |
|  | INF2 |  |
|  | TGFA |  |
|  | AIRE |  |
|  | LIPI |  |
|  | CWC27 |  |
|  | MFF-DT |  |
|  | SRI |  |
|  | CLCN3 |  |
|  | DDX25 |  |
|  | ASS1 |  |
|  | OBSL1 |  |
|  | ARL13B |  |
|  | RANBP2 |  |
|  | CATSPER2 |  |
|  | PRKDC |  |
|  | OTULIN |  |
|  | ARMC2-AS1 |  |
|  | MBP |  |
|  | MGME1 |  |
|  | ELN-AS1 |  |
|  | HTT |  |
|  | RBBP8 |  |
|  | LIPT1 |  |
|  | IL5 |  |
|  | POMT2 |  |
|  | MRPS22 |  |
|  | TTC21B-AS1 |  |
|  | DGCR5 |  |
|  | MCAM |  |
|  | TIMP3 |  |
|  | SF3B1 |  |
|  | AOC3 |  |
|  | HHEX |  |
|  | MPDU1 |  |
|  | BICC1 |  |
|  | HPD |  |
|  | ATPAF2 |  |
|  | NFKB2 |  |
|  | BIN1 |  |
|  | SH2B3 |  |
|  | DPYD |  |
|  | DLST |  |
|  | MAGI2 |  |
|  | ATXN1 |  |
|  | PGK1 |  |
|  | RTTN |  |
|  | IL23R |  |
|  | CDH5 |  |
|  | RPS28 |  |
|  | SYNPO2L |  |
|  | STRADA |  |
|  | LAMC2 |  |
|  | GAD1 |  |
|  | TNC |  |
|  | USP53 |  |
|  | IDH2 |  |
|  | MCIDAS |  |
|  | LYRM4 |  |
|  | CRB2 |  |
|  | AQP1 |  |
|  | AKAP6 |  |
|  | LIFR |  |
|  | GGN |  |
|  | PRKAA2 |  |
|  | CLPP |  |
|  | ACSF3 |  |
|  | USF1 |  |
|  | CD44 |  |
|  | HSF2 |  |
|  | AK1 |  |
|  | FAHD1 |  |
|  | DDAH2 |  |
|  | MAN2B1 |  |
|  | FUS |  |
|  | GPC6 |  |
|  | UNC80 |  |
|  | RBFOX2 |  |
|  | NDUFA2 |  |
|  | FREM1 |  |
|  | KLF4 |  |
|  | KLK3 |  |
|  | PARVA |  |
|  | BCL11A |  |
|  | CLDN19 |  |
|  | ACP5 |  |
|  | PCDH7 |  |
|  | SP1 |  |
|  | TRAPPC11 |  |
|  | COL6A2 |  |
|  | ELP4 |  |
|  | CCL4 |  |
|  | CATIP-AS2 |  |
|  | EPHX2 |  |
|  | PROCR |  |
|  | IL1RAPL2 |  |
|  | PROX1 |  |
|  | ABCD3 |  |
|  | CLMP |  |
|  | RPS7 |  |
|  | EXOSC8 |  |
|  | CDC45 |  |
|  | GATB |  |
|  | SOCS1 |  |
|  | KCNB1 |  |
|  | ERF |  |
|  | SERPINF2 |  |
|  | CAMKMT |  |
|  | PROP1 |  |
|  | DNAAF6 |  |
|  | GC |  |
|  | DAW1 |  |
|  | ZNFX1 |  |
|  | ATP1A3 |  |
|  | ESS2 |  |
|  | NSUN2 |  |
|  | HCN1 |  |
|  | CFLAR |  |
|  | MIR486-1 |  |
|  | SOX6 |  |
|  | CDIN1 |  |
|  | CKMT2 |  |
|  | NXF5 |  |
|  | IL12RB1 |  |
|  | MIR15B |  |
|  | DVL2 |  |
|  | PRDX1 |  |
|  | AGRN |  |
|  | WDR72 |  |
|  | PKD1-AS1 |  |
|  | SH2D1A |  |
|  | AXIN1 |  |
|  | TCF20 |  |
|  | ICOSLG |  |
|  | SSBP1 |  |
|  | XPNPEP3 |  |
|  | PIGP |  |
|  | CD59 |  |
|  | RTN4 |  |
|  | NTRK2 |  |
|  | FGF7 |  |
|  | PLP1 |  |
|  | FUT8 |  |
|  | ZMYND10 |  |
|  | HENMT1 |  |
|  | APOC1 |  |
|  | CACNA1H |  |
|  | PEPD |  |
|  | TARID |  |
|  | ST2 |  |
|  | SLC1A3 |  |
|  | OGDH |  |
|  | CD3D |  |
|  | AVPR1A |  |
|  | EPB41L4A |  |
|  | EEF1A2 |  |
|  | SMG8 |  |
|  | KRT5 |  |
|  | ITPR1 |  |
|  | MRE11 |  |
|  | KAT5 |  |
|  | CEP104 |  |
|  | A4GALT |  |
|  | ITGB1BP2 |  |
|  | FKBP14 |  |
|  | AK2 |  |
|  | TRIP11 |  |
|  | LARGE1 |  |
|  | MIR30B |  |
|  | RBM8A |  |
|  | KLHL41 |  |
|  | FGF13 |  |
|  | GANAB |  |
|  | ALG13 |  |
|  | H19-ICR |  |
|  | OPTN |  |
|  | BCL6 |  |
|  | OTUD5 |  |
|  | MS4A1 |  |
|  | POLR2A |  |
|  | ROBO2 |  |
|  | TPP1 |  |
|  | GFRA1 |  |
|  | SLC19A1 |  |
|  | MIR149 |  |
|  | CSF2RA |  |
|  | UCP1 |  |
|  | MMP8 |  |
|  | LTC4S |  |
|  | AFF3 |  |
|  | NAGS |  |
|  | PDLIM3 |  |
|  | DDX3X |  |
|  | HOXA13 |  |
|  | EXTL3 |  |
|  | CCL26 |  |
|  | TP73 |  |
|  | TUSC7 |  |
|  | CHMP2B |  |
|  | DAND5 |  |
|  | CD96 |  |
|  | NDN |  |
|  | SF3B2 |  |
|  | TBL1X |  |
|  | PLVAP |  |
|  | FOXG1 |  |
|  | BACE1-AS |  |
|  | MIR494 |  |
|  | STRC |  |
|  | GH-LCR |  |
|  | TBXT |  |
|  | ISCU |  |
|  | KCNQ2 |  |
|  | EPB42 |  |
|  | LOC106029312 |  |
|  | IDH1 |  |
|  | ADSL |  |
|  | JAZF1 |  |
|  | ACOX1 |  |
|  | IRF6 |  |
|  | CCL17 |  |
|  | ATP6V1B1 |  |
|  | SLC19A3 |  |
|  | CXCR2 |  |
|  | SUN2 |  |
|  | RBM25 |  |
|  | TGFBR3 |  |
|  | MT-LIPCAR |  |
|  | ADCY6 |  |
|  | RAB3GAP1 |  |
|  | STAT6 |  |
|  | MGAT2 |  |
|  | SFTPA2 |  |
|  | MIR483 |  |
|  | GLP1R |  |
|  | MRPL12 |  |
|  | ITGA4 |  |
|  | GMPPB |  |
|  | SLC35A2 |  |
|  | CFAP52 |  |
|  | THSD1 |  |
|  | PPP3CB |  |
|  | XIRP2 |  |
|  | IL2RG |  |
|  | PPP2R5D |  |
|  | DGCR6 |  |
|  | PC |  |
|  | SCN3A |  |
|  | NRIP1 |  |
|  | SYNGAP1 |  |
|  | ITCH |  |
|  | SLC10A2 |  |
|  | NUS1 |  |
|  | SLC39A13 |  |
|  | WNT7B |  |
|  | COA6 |  |
|  | C12orf29 |  |
|  | STAMBP |  |
|  | LOC105371046 |  |
|  | PTCH2 |  |
|  | PALLD |  |
|  | LOC109280163 |  |
|  | DDOST |  |
|  | FGFRL1 |  |
|  | SLC41A1 |  |
|  | AHSP |  |
|  | GREB1L |  |
|  | IL11 |  |
|  | ALG3 |  |
|  | NCAPH2 |  |
|  | PRKCQ |  |
|  | ASL |  |
|  | MPDZ |  |
|  | MIR222 |  |
|  | CNTROB |  |
|  | KAT2B |  |
|  | NRP1 |  |
|  | RGS4 |  |
|  | CPOX |  |
|  | FANCD2OS |  |
|  | VLDLR |  |
|  | PDE6D |  |
|  | S100A12 |  |
|  | NRON |  |
|  | CDKN1B |  |
|  | SLC24A3 |  |
|  | HBB-LCR |  |
|  | PGAP1 |  |
|  | MKRN3 |  |
|  | GNPTG |  |
|  | PDSS1 |  |
|  | MT-TA |  |
|  | NISCH |  |
|  | PIGN |  |
|  | LAMB3 |  |
|  | PDE11A |  |
|  | KLF13 |  |
|  | TUBA1A |  |
|  | VASP |  |
|  | RIPK4 |  |
|  | APTX |  |
|  | C12orf57 |  |
|  | COPB2 |  |
|  | GBA2 |  |
|  | RNPC3 |  |
|  | OCA2 |  |
|  | WNT10B |  |
|  | TRAF1 |  |
|  | FARSB |  |
|  | MYO1H |  |
|  | IL21 |  |
|  | NTS |  |
|  | SEMA3D |  |
|  | LRIG2 |  |
|  | DPM2 |  |
|  | KISS1R |  |
|  | CYP2B6 |  |
|  | MLH1 |  |
|  | MSH3 |  |
|  | CYP2E1 |  |
|  | EMC10 |  |
|  | RPS27 |  |
|  | DPP6 |  |
|  | SLC10A7 |  |
|  | MCMDC2 |  |
|  | ANTXR1 |  |
|  | KCNAB1 |  |
|  | NFATC2 |  |
|  | ZP2 |  |
|  | MIB2 |  |
|  | COQ8A |  |
|  | SIRT3 |  |
|  | UTS2R |  |
|  | NOL3 |  |
|  | KIAA0319L |  |
|  | GNS |  |
|  | NIPA1 |  |
|  | AQP4 |  |
|  | EIF2B4 |  |
|  | COQ7 |  |
|  | NBPF12 |  |
|  | SCARF2 |  |
|  | ICOS |  |
|  | ALG11 |  |
|  | RFT1 |  |
|  | BUB1 |  |
|  | PAX3 |  |
|  | GPX4 |  |
|  | PFAS |  |
|  | DCC |  |
|  | QRSL1 |  |
|  | SLC5A1 |  |
|  | DPAGT1 |  |
|  | CBY1 |  |
|  | KATNIP |  |
|  | SSR4 |  |
|  | MYO18B |  |
|  | GAL |  |
|  | CFHR4 |  |
|  | ALDOB |  |
|  | PLTP |  |
|  | SP7 |  |
|  | STAC3 |  |
|  | HSD3B1 |  |
|  | MNS1 |  |
|  | PMP22 |  |
|  | XRCC5 |  |
|  | PIGY |  |
|  | TNFRSF13C |  |
|  | SEC31A |  |
|  | TAF6 |  |
|  | SYT2 |  |
|  | WNT2B |  |
|  | TFAP2A |  |
|  | ARSL |  |
|  | COL11A1 |  |
|  | LBP |  |
|  | ANXA6 |  |
|  | ABHD5 |  |
|  | C11orf65 |  |
|  | TUBG1 |  |
|  | SLC25A11 |  |
|  | GARS1 |  |
|  | CSF2RB |  |
|  | SGO1-AS1 |  |
|  | AAAS |  |
|  | CPT1B |  |
|  | NCAPG2 |  |
|  | MIR196A1 |  |
|  | TBX6 |  |
|  | STT3B |  |
|  | DGAT1 |  |
|  | HUWE1 |  |
|  | DNASE1L3 |  |
|  | TLR8 |  |
|  | INSL3 |  |
|  | NOG |  |
|  | XRCC6 |  |
|  | STK4 |  |
|  | TFEB |  |
|  | C5 |  |
|  | FCGR3A |  |
|  | SLC22A4 |  |
|  | CALB2 |  |
|  | SRPRA |  |
|  | ANK3 |  |
|  | BPTF |  |
|  | AIP |  |
|  | CCDC28B |  |
|  | AP1B1 |  |
|  | CTSG |  |
|  | FOXO1 |  |
|  | PDLIM1 |  |
|  | YAP1 |  |
|  | KCNA2 |  |
|  | HCRT |  |
|  | IRF2BP2 |  |
|  | KRT18 |  |
|  | WNK1 |  |
|  | SLC11A1 |  |
|  | DEAF1 |  |
|  | CLTC |  |
|  | TRIO |  |
|  | LOC107303338 |  |
|  | CYP7B1 |  |
|  | NOTCH4 |  |
|  | OGA |  |
|  | CFAP298-TCP10L |  |
|  | ANTXR2 |  |
|  | SCAPER |  |
|  | CHIT1 |  |
|  | ATG5 |  |
|  | PIK3R2 |  |
|  | SHC1 |  |
|  | TMEM165 |  |
|  | COX14 |  |
|  | RLBP1 |  |
|  | ATP12A |  |
|  | CANT1 |  |
|  | ALG6 |  |
|  | CTH |  |
|  | MMAB |  |
|  | ITPR3 |  |
|  | IAPP |  |
|  | PYCR1 |  |
|  | FADS2 |  |
|  | ALAS2 |  |
|  | SLC52A3 |  |
|  | VEGFB |  |
|  | MED23 |  |
|  | RARS2 |  |
|  | PDK4 |  |
|  | PARD3 |  |
|  | FGF17 |  |
|  | COL6A3 |  |
|  | RIPK1 |  |
|  | KIF24 |  |
|  | DDAH1 |  |
|  | ARSA |  |
|  | CENPT |  |
|  | CXCR3 |  |
|  | HIC1 |  |
|  | MICA |  |
|  | RPS14 |  |
|  | MAPRE2 |  |
|  | CDKN2B |  |
|  | ATP6V1E1 |  |
|  | COL12A1 |  |
|  | FLRT2 |  |
|  | TRIM54 |  |
|  | FADD |  |
|  | ITGA3 |  |
|  | HERC2 |  |
|  | KCNN2 |  |
|  | DNAH7 |  |
|  | EXOC6B |  |
|  | NACC1 |  |
|  | SRSF2 |  |
|  | MAPKBP1 |  |
|  | CD274 |  |
|  | ESRRG |  |
|  | RIGI |  |
|  | ATN1 |  |
|  | POMGNT1 |  |
|  | KHDRBS1 |  |
|  | NEUROG3 |  |
|  | MECOM |  |
|  | TNFRSF25 |  |
|  | KCNK1 |  |
|  | SRP19 |  |
|  | COQ5 |  |
|  | MCU |  |
|  | SMAD1 |  |
|  | NES |  |
|  | NIPSNAP3B |  |
|  | RGS2 |  |
|  | NRTN |  |
|  | CCR3 |  |
|  | GRIN1 |  |
|  | PCSK1 |  |
|  | LOC107982234 |  |
|  | S100A9 |  |
|  | LOC107303340 |  |
|  | SEC24D |  |
|  | NUP93 |  |
|  | DPM3 |  |
|  | ATFB1 |  |
|  | NSDHL |  |
|  | KLF15 |  |
|  | KAT6A |  |
|  | KMT5B |  |
|  | UCP3 |  |
|  | TMEM138 |  |
|  | M6PR |  |
|  | HDAC2 |  |
|  | RPL18 |  |
|  | MIR101-1 |  |
|  | MILR1 |  |
|  | ATXN10 |  |
|  | CALD1 |  |
|  | MUS81 |  |
|  | QDPR |  |
|  | PFKM |  |
|  | DSPP |  |
|  | KCNE4 |  |
|  | PLEK |  |
|  | HTRA1 |  |
|  | AXDND1 |  |
|  | HMCN1 |  |
|  | KDM6B |  |
|  | RLN1 |  |
|  | DNAH6 |  |
|  | CEP41 |  |
|  | ATP5F1D |  |
|  | XBP1 |  |
|  | PROC |  |
|  | PGAP2 |  |
|  | UQCC2 |  |
|  | GDF3 |  |
|  | RPL31 |  |
|  | MIR191 |  |
|  | KLKB1 |  |
|  | CDK9 |  |
|  | COL17A1 |  |
|  | UBE2L3 |  |
|  | ATR |  |
|  | CFAP45 |  |
|  | SIN3A |  |
|  | FADS1 |  |
|  | TOPORS |  |
|  | SPRY4 |  |
|  | PNKP |  |
|  | RPS10-NUDT3 |  |
|  | RO60 |  |
|  | NIPA2 |  |
|  | ATAD3A |  |
|  | VAPB |  |
|  | PYCR2 |  |
|  | CRTAP |  |
|  | ACSL4 |  |
|  | TRPC1 |  |
|  | SGK1 |  |
|  | D2HGDH |  |
|  | HEATR3 |  |
|  | SLC7A9 |  |
|  | DEL16P13.3 |  |
|  | PPM1B |  |
|  | PTLS |  |
|  | SLC26A4 |  |
|  | WAC |  |
|  | DSTYK |  |
|  | EXT1 |  |
|  | KRIT1 |  |
|  | CYSLTR1 |  |
|  | TULP3 |  |
|  | CGA |  |
|  | CDK5RAP3 |  |
|  | TIMM50 |  |
|  | MBD5 |  |
|  | FPGT-TNNI3K |  |
|  | TBCE |  |
|  | ACO2 |  |
|  | RNF8 |  |
|  | JAK1 |  |
|  | FTL |  |
|  | LRRK2 |  |
|  | FGF16 |  |
|  | FBXO32 |  |
|  | CARS2 |  |
|  | PAPPA2 |  |
|  | NADSYN1 |  |
|  | GLRX5 |  |
|  | UBAC2 |  |
|  | KDM5C |  |
|  | DOCK8 |  |
|  | LOC112486223 |  |
|  | ANG |  |
|  | ASTN2 |  |
|  | LRP4 |  |
|  | SOCS3 |  |
|  | DDB1 |  |
|  | SIX5 |  |
|  | ADIPOR1 |  |
|  | ADAMTS19 |  |
|  | SMAD7 |  |
|  | RGS3 |  |
|  | RPL10 |  |
|  | EFNB2 |  |
|  | TASP1 |  |
|  | NAXE |  |
|  | MUC5B |  |
|  | EDARADD |  |
|  | SLN |  |
|  | FOXL1 |  |
|  | STX3 |  |
|  | MX1 |  |
|  | ADM2 |  |
|  | TYK2 |  |
|  | MIR19B1 |  |
|  | SYT1 |  |
|  | PAX4 |  |
|  | FRZB |  |
|  | CNOT1 |  |
|  | ARHGAP24 |  |
|  | TUFM |  |
|  | MIR23B |  |
|  | DDX59 |  |
|  | SIX1 |  |
|  | CHI3L1 |  |
|  | MIR125B1 |  |
|  | TNFSF13B |  |
|  | CSNK2A2 |  |
|  | BNIP3 |  |
|  | CD2AP |  |
|  | PNP |  |
|  | ATP6V1B2 |  |
|  | ACTL6A |  |
|  | HPSE2 |  |
|  | SLC25A15 |  |
|  | AXIN2 |  |
|  | CLDN5 |  |
|  | ADD3 |  |
|  | SLC46A1 |  |
|  | ZNF423 |  |
|  | EHHADH |  |
|  | JAGN1 |  |
|  | WBP11 |  |
|  | NDP |  |
|  | FAM111A |  |
|  | CLPB |  |
|  | SNIP1 |  |
|  | NRAP |  |
|  | PUS1 |  |
|  | COL13A1 |  |
|  | NSMF |  |
|  | LMNB2 |  |
|  | VRK1 |  |
|  | DHDDS |  |
|  | P3H1 |  |
|  | ORC4 |  |
|  | KISS1 |  |
|  | CCK |  |
|  | ADAMTS9 |  |
|  | ADAMTS3 |  |
|  | GOT2 |  |
|  | ABCC3 |  |
|  | USH2A |  |
|  | PDGFA |  |
|  | VDAC1 |  |
|  | EDA |  |
|  | ARAF |  |
|  | LHCGR |  |
|  | PAX5 |  |
|  | SMYD2 |  |
|  | PAEP |  |
|  | FGB |  |
|  | BLOC1S1 |  |
|  | LTF |  |
|  | GHRH |  |
|  | ERMARD |  |
|  | AEBP1 |  |
|  | IFT88 |  |
|  | APC2 |  |
|  | CYP2A6 |  |
|  | LOC107548112 |  |
|  | PTDSS1 |  |
|  | RPL27 |  |
|  | MIR106B |  |
|  | TRIP13 |  |
|  | ARNT2 |  |
|  | CD80 |  |
|  | SLC26A2 |  |
|  | PPP1R1A |  |
|  | NLGN4X |  |
|  | PPP1CA |  |
|  | GLUD1 |  |
|  | SLC6A8 |  |
|  | USP7 |  |
|  | LFNG |  |
|  | CDAN1 |  |
|  | PRKCSH |  |
|  | PSMB4 |  |
|  | CHN1 |  |
|  | TAC3 |  |
|  | TRAF6 |  |
|  | TSEN54 |  |
|  | UBE2A |  |
|  | ABCC4 |  |
|  | RNU7-1 |  |
|  | LDHA |  |
|  | PNMT |  |
|  | TJP1 |  |
|  | DTNBP1 |  |
|  | TMOD1 |  |
|  | SRA1 |  |
|  | KMT2B |  |
|  | CMYA5 |  |
|  | MIR424 |  |
|  | GPX1 |  |
|  | RASGRP1 |  |
|  | TBX22 |  |
|  | SLC13A5 |  |
|  | TNIP1 |  |
|  | SERPINH1 |  |
|  | SUMO1 |  |
|  | AIFM1 |  |
|  | FZD1 |  |
|  | ATP5MK |  |
|  | CPE |  |
|  | ANXA2 |  |
|  | RAB7A |  |
|  | FNDC5 |  |
|  | FRMD4B |  |
|  | NUP62 |  |
|  | CEBPA |  |
|  | CR1 |  |
|  | CHRNB1 |  |
|  | HLA-DPA1 |  |
|  | FOXN1 |  |
|  | SLC9A6 |  |
|  | SULT1A3 |  |
|  | MCCC2 |  |
|  | PPP1R1B |  |
|  | SMPX |  |
|  | SLC51A |  |
|  | TOP3A |  |
|  | DPF3 |  |
|  | DPM1 |  |
|  | ITGB4 |  |
|  | G6PC1 |  |
|  | KCNJ10 |  |
|  | PXK |  |
|  | PNPLA3 |  |
|  | ACOX2 |  |
|  | PCK2 |  |
|  | UCN2 |  |
|  | PEBP1 |  |
|  | DSTN |  |
|  | CIDEA |  |
|  | BAMBI |  |
|  | NOX1 |  |
|  | CXCL2 |  |
|  | APCS |  |
|  | ACLY |  |
|  | ATP2A1 |  |
|  | PRKAR2B |  |
|  | MAP2K7 |  |
|  | FIP1L1 |  |
|  | KAT8 |  |
|  | MAS1 |  |
|  | P2RX4 |  |
|  | FXYD1 |  |
|  | KCNK2 |  |
|  | ARRB1 |  |
|  | PPP1CC |  |
|  | DDR1 |  |
|  | DYNLL1 |  |
|  | CASP12 |  |
|  | CHRM4 |  |
|  | CHRNA4 |  |
|  | DIO3 |  |
|  | ACAN |  |
|  | ENDOG |  |
|  | NPTXR |  |
|  | NGFR |  |
|  | PLCD1 |  |
|  | PLD2 |  |
|  | MED1 |  |
|  | GRK1 |  |
|  | FOSL1 |  |
|  | OGT |  |
|  | BECN1 |  |
|  | TOMM70 |  |
|  | ZGLP1 |  |
|  | C8orf37-AS1 |  |
|  | CERT1 |  |
|  | LINC01782 |  |
|  | LIPC-AS1 |  |
|  | CXCR6 |  |
|  | CMTM7 |  |
|  | OTUD7A |  |
|  | CELSR2 |  |
|  | SIK3 |  |
|  | COPD |  |
|  | GCKR |  |
|  | GPR42 |  |
|  | GRHL1 |  |
|  | ITPK1 |  |
|  | POLK |  |
|  | PLCG1 |  |
|  | SEMA5B |  |
|  | CHDH |  |
|  | MAML3 |  |
|  | ANKS1B |  |
|  | ACKR3 |  |
|  | CPNE5 |  |
|  | SUGP1 |  |
|  | REG1A |  |
|  | RFC1 |  |
|  | SSTR4 |  |
|  | BRS3 |  |
|  | TM7SF2 |  |
|  | VPS51 |  |
|  | BEST1 |  |
|  | SLC30A3 |  |
|  | FSD1 |  |
|  | C1orf21 |  |
|  | FSD1L |  |
|  | ZPR1 |  |
|  | LPAR2 |  |
|  | CYTH3 |  |
|  | DECR1 |  |
|  | CAD |  |
|  | GOLGA6A |  |
|  | MIR25 |  |
|  | MFAP1 |  |
|  | MUC2 |  |
|  | RCBTB1 |  |
|  | MARCKSL1 |  |
|  | AHSA1 |  |
|  | MRGPRX3 |  |
|  | MRGPRX4 |  |
|  | GPR151 |  |
|  | CRK |  |
|  | OXER1 |  |
|  | GPRC6A |  |
|  | LPAR3 |  |
|  | RNF19A |  |
|  | MRGPRX1 |  |
|  | POLDIP2 |  |
|  | VN1R17P |  |
|  | GPR166P |  |
|  | LGR6 |  |
|  | AIMP2 |  |
|  | GRAP2 |  |
|  | PGR-AS1 |  |
|  | GABPA |  |
|  | UTRN |  |
|  | SLC33A1 |  |
|  | MTCO2P12 |  |
|  | PPARGC1B |  |
|  | CRMP1 |  |
|  | CSH1 |  |
|  | CSH2 |  |
|  | DAPK2 |  |
|  | HSPA9 |  |
|  | COX2 |  |
|  | NM |  |
|  | CCHCR1 |  |
|  | DENR |  |
|  | SELENBP1 |  |
|  | MIR665 |  |
|  | FSTL1 |  |
|  | PRRT2 |  |
|  | MMRN1 |  |
|  | INSRR |  |
|  | PRKAA1 |  |
|  | CENPJ |  |
|  | SDC4 |  |
|  | TAZ |  |
|  | ARHGEF5 |  |
|  | PPP1R2C |  |
|  | TIMELESS |  |
|  | TLX1NB |  |
|  | ABCB6 |  |
|  | FSTL3 |  |
|  | FST |  |
|  | MORF4 |  |
|  | IL34 |  |
|  | SMYD1 |  |
|  | GRK3 |  |
|  | DBP |  |
|  | AAVS1 |  |
|  | IL37 |  |
|  | CHAMP1 |  |
|  | NR4A1 |  |
|  | TLX2 |  |
|  | RND3 |  |
|  | MIR199A2 |  |
|  | MIR19A |  |
|  | OSM |  |
|  | P2RX1 |  |
|  | P2RY2 |  |
|  | TNFRSF12A |  |
|  | SCARA3 |  |
|  | PENK |  |
|  | PIN1 |  |
|  | KRT20 |  |
|  | RBFOX1 |  |
|  | PPIA |  |
|  | PTPA |  |
|  | PRKAB1 |  |
|  | MOK |  |
|  | ROS1 |  |
|  | GINGF2 |  |
|  | PINK1 |  |
|  | THBS2 |  |
|  | DNER |  |
|  | C20orf181 |  |
|  | HDAC5 |  |
|  | P2RX5-TAX1BP3 |  |
|  | GDF11 |  |
|  | NOD1 |  |
|  | RACK1 |  |
|  | SPON1 |  |
|  | ATG7 |  |
|  | SLC35A1 |  |
|  | SLCO1B1 |  |
|  | CST12P |  |
|  | RIPK3 |  |
|  | UCN3 |  |
|  | CNP |  |
|  | CREM |  |
|  | CRHR2 |  |
|  | CRX |  |
|  | CRYGC |  |
|  | CTNNA1 |  |
|  | TMTC3 |  |
|  | S1PR1 |  |
|  | ELAVL2 |  |
|  | CRYGEP |  |
|  | ENPEP |  |
|  | FBL |  |
|  | F2RL1 |  |
|  | FGFR4 |  |
|  | VEGFD |  |
|  | P2RX2 |  |
|  | MPRIP |  |
|  | MLC1 |  |
|  | ANGPTL2 |  |
|  | ABCA4 |  |
|  | GAS6 |  |
|  | GHSR |  |
|  | PYCARD |  |
|  | HTR4 |  |
|  | IGFBP4 |  |
|  | IGHG3 |  |
|  | ILK |  |
|  | IRF1 |  |
|  | LUM |  |
|  | MIR130B |  |
|  | MIR18A |  |
|  | MIR212 |  |
|  | MIR216A |  |
|  | MIR27B |  |
|  | MIR29B2 |  |
|  | ARRB2 |  |
|  | STS |  |
|  | MIR340 |  |
|  | MIR342 |  |
|  | COX1 |  |
|  | CNOT3 |  |
|  | CCN3 |  |
|  | ACR |  |
|  | NT5E |  |
|  | ATP2B4 |  |
|  | TRIM72 |  |
|  | MIR425 |  |
|  | P2RX5 |  |
|  | P2RY1 |  |
|  | PCSK6 |  |
|  | DUOX2 |  |
|  | SERPINA5 |  |
|  | GP6 |  |
|  | UBR5 |  |
|  | ISYNA1 |  |
|  | PDK1 |  |
|  | LUC7L3 |  |
|  | DUOX1 |  |
|  | RNF111 |  |
|  | MFN1 |  |
|  | ERBIN |  |
|  | BIRC6 |  |
|  | CREBZF |  |
|  | ROCK1 |  |
|  | BDH1 |  |
|  | CCL19 |  |
|  | CCL21 |  |
|  | TMBIM1 |  |
|  | BMI1 |  |
|  | BNIP3L |  |
|  | STIM1 |  |
|  | ZEB1 |  |
|  | HOPX |  |
|  | TNFRSF10B |  |
|  | CCN5 |  |
|  | P2RX6 |  |
|  | GAL3ST1 |  |
|  | RNA18SN5 |  |
|  | NR2E3 |  |
|  | MIR675 |  |
|  | MIR147B |  |
|  | MIR744 |  |
|  | TRAP |  |
|  | HLP |  |
|  | MIR1306 |  |
|  | NPPA-AS1 |  |
|  | TMX2-CTNND1 |  |
|  | PPR1 |  |
|  | KLRC4-KLRK1 |  |
|  | FAME3 |  |
|  | MIR4491 |  |
|  | COX17 |  |
|  | DPP3 |  |
|  | LINC-ROR |  |
|  | NPY4R2 |  |
|  | PPIF |  |
|  | OPN1MW3 |  |
|  | HIPK3 |  |
|  | FRY |  |
|  | CDH15 |  |
|  | RASA4 |  |
|  | ALYREF |  |
|  | AK6 |  |
|  | CALCOCO2 |  |
|  | STUB1 |  |
|  | MARCHF6 |  |
|  | CNPY2 |  |
|  | KLF2 |  |
|  | CDS1 |  |
|  | PRMT5 |  |
|  | LINC02210-CRHR1 |  |
|  | CIB1 |  |
|  | PERCC1 |  |
|  | CXCL13 |  |
|  | SLU7 |  |
|  | GNLY |  |
|  | SCGN |  |
|  | YME1L1 |  |
|  | TCFL5 |  |
|  | CGB3 |  |
|  | LILRB1 |  |
|  | SUGT1 |  |
|  | FASTK |  |
|  | PRDX3 |  |
|  | CKAP4 |  |
|  | PDXDC2P |  |
|  | FERMT2 |  |
|  | LILRB4 |  |
|  | LIAS |  |
|  | CHGB |  |
|  | WDHD1 |  |
|  | MAP4K5 |  |
|  | USP18 |  |
|  | LYST |  |
|  | ECD |  |
|  | ACOT7 |  |
|  | AZIN2 |  |
|  | TXNRD3 |  |
|  | FHAD1 |  |
|  | C1QTNF1 |  |
|  | OMA1 |  |
|  | CIRBP |  |
|  | CISH |  |
|  | ADCYAP1 |  |
|  | ADCYAP1R1 |  |
|  | JAML |  |
|  | JDP2 |  |
|  | CCR4 |  |
|  | CCR7 |  |
|  | CNN1 |  |
|  | CNR2 |  |
|  | MYOM3 |  |
|  | CMPK2 |  |
|  | RBM45 |  |
|  | KLF6 |  |
|  | EMB |  |
|  | SLCO6A1 |  |
|  | KLF14 |  |
|  | CRABP1 |  |
|  | FUNDC1 |  |
|  | ASB14 |  |
|  | CSE1L |  |
|  | LGALS16 |  |
|  | CTNND1 |  |
|  | CTRL |  |
|  | RMDN2 |  |
|  | PPM1K |  |
|  | AMZ1 |  |
|  | WBP2NL |  |
|  | GADD45A |  |
|  | DDT |  |
|  | TIMM8A |  |
|  | SLC30A8 |  |
|  | HFM |  |
|  | ADAMTS16 |  |
|  | PPP1R18 |  |
|  | NLRP6 |  |
|  | DUSP2 |  |
|  | E2F6 |  |
|  | EGR1 |  |
|  | EIF4EBP1 |  |
|  | ELAVL1 |  |
|  | DHRS7C |  |
|  | CTTN |  |
|  | PRSS55 |  |
|  | EPHA3 |  |
|  | ETV3 |  |
|  | ALCAM |  |
|  | FBLN1 |  |
|  | TPCN2 |  |
|  | FDPS |  |
|  | FDXR |  |
|  | FGF4 |  |
|  | FKBP1AP1 |  |
|  | FKBP1AP2 |  |
|  | FKBP1AP3 |  |
|  | FKBP1AP4 |  |
|  | KLRK1 |  |
|  | MON2 |  |
|  | PDS5B |  |
|  | FOXM1 |  |
|  | ARC |  |
|  | PDS5A |  |
|  | FMOD |  |
|  | UFL1 |  |
|  | TRS-AGA2-3 |  |
|  | POFUT1 |  |
|  | SLC39A14 |  |
|  | SMUG1 |  |
|  | PHLDA3 |  |
|  | OSBP2 |  |
|  | IL17RA |  |
|  | DIANPH |  |
|  | BRD1 |  |
|  | RICTOR |  |
|  | GAB1 |  |
|  | TXN2 |  |
|  | PART1 |  |
|  | GALNT1 |  |
|  | TOR1AIP1 |  |
|  | LRIT1 |  |
|  | OPTC |  |
|  | GCH1 |  |
|  | OPN1MW |  |
|  | OPLAH |  |
|  | CBLIF |  |
|  | VPS4A |  |
|  | OXGR1 |  |
|  | EIF3K |  |
|  | MAT2B |  |
|  | TOR2A |  |
|  | GNA12 |  |
|  | ABO |  |
|  | GOLGB1 |  |
|  | SLCO1B3 |  |
|  | NEAT1 |  |
|  | GPR17 |  |
|  | SYPL2 |  |
|  | BTBD8 |  |
|  | SLC9C1 |  |
|  | HIPK2 |  |
|  | ANPEP |  |
|  | LGALS13 |  |
|  | CXCL1 |  |
|  | GSK3A |  |
|  | GUCA2B |  |
|  | GNL2 |  |
|  | RMC1 |  |
|  | MDFIC |  |
|  | H2AX |  |
|  | HDC |  |
|  | KCNIP3 |  |
|  | EHD3 |  |
|  | EHD2 |  |
|  | HK2 |  |
|  | HMBS |  |
|  | HMGB2 |  |
|  | HNRNPD |  |
|  | APOF |  |
|  | HRH2 |  |
|  | PRMT1 |  |
|  | FFAR4 |  |
|  | C1QTNF9 |  |
|  | ID2 |  |
|  | NRBP2 |  |
|  | COL6A4P1 |  |
|  | ACTBL2 |  |
|  | IGFBP5 |  |
|  | IL4R |  |
|  | IL13RA1 |  |
|  | ILF3 |  |
|  | ITGB2 |  |
|  | ITPR2 |  |
|  | GSTK1 |  |
|  | KCP |  |
|  | ENHO |  |
|  | MALAT1 |  |
|  | NPSR1 |  |
|  | LAD1 |  |
|  | LGALS1 |  |
|  | LY75 |  |
|  | MIR100 |  |
|  | MIR127 |  |
|  | MIR134 |  |
|  | MIR137 |  |
|  | MIR148A |  |
|  | MIR183 |  |
|  | MIR185 |  |
|  | MIR197 |  |
|  | MIR199B |  |
|  | MIR200B |  |
|  | MIR296 |  |
|  | MIR30C1 |  |
|  | MIR30C2 |  |
|  | MIR30E |  |
|  | MIR33A |  |
|  | MIR93 |  |
|  | MIR99A |  |
|  | TNFSF12-TNFSF13 |  |
|  | MAP6 |  |
|  | ARSD |  |
|  | MATN1 |  |
|  | SMCP |  |
|  | ME1 |  |
|  | MGAT1 |  |
|  | MICE |  |
|  | MITF |  |
|  | MAP3K11 |  |
|  | AFDN |  |
|  | MRC1 |  |
|  | PLIN5 |  |
|  | POTEKP |  |
|  | MIR148B |  |
|  | MIR324 |  |
|  | TRNT |  |
|  | MYF6 |  |
|  | MYOC |  |
|  | PPP1R12A |  |
|  | ATF3 |  |
|  | NDUFAB1 |  |
|  | NFE2L1 |  |
|  | NFIL3 |  |
|  | NHS |  |
|  | NME3 |  |
|  | NNMT |  |
|  | ATP2A3 |  |
|  | NRF1 |  |
|  | MIR375 |  |
|  | OGN |  |
|  | ORM1 |  |
|  | ALDH7A1 |  |
|  | NOX3 |  |
|  | IL22 |  |
|  | PAM |  |
|  | AK3 |  |
|  | NTM |  |
|  | APIP |  |
|  | PCMT1 |  |
|  | RMDN1 |  |
|  | ANGPTL4 |  |
|  | UBAP1 |  |
|  | BFAR |  |
|  | PDC |  |
|  | PDE1C |  |
|  | TRPV2 |  |
|  | SUCO |  |
|  | UFM1 |  |
|  | GDE1 |  |
|  | NLK |  |
|  | CMPK1 |  |
|  | PFN1 |  |
|  | PIGF |  |
|  | PITX3 |  |
|  | TPCN1 |  |
|  | CNTN5 |  |
|  | TLR9 |  |
|  | CYTL1 |  |
|  | DDIT4 |  |
|  | EGLN1 |  |
|  | PPA1 |  |
|  | PPID |  |
|  | MARCHF1 |  |
|  | MOCOS |  |
|  | WIPI1 |  |
|  | SLC52A1 |  |
|  | PPP1R7 |  |
|  | RMDN3 |  |
|  | PPP2R1A |  |
|  | ADI1 |  |
|  | AVPR1B |  |
|  | PPP5C |  |
|  | IMPACT |  |
|  | PPT1 |  |
|  | NPY4R |  |
|  | SRGN |  |
|  | SLC30A10 |  |
|  | SYBU |  |
|  | ENAH |  |
|  | DCAF6 |  |
|  | USE1 |  |
|  | BEX1 |  |
|  | PKN2 |  |
|  | ZKSCAN7 |  |
|  | ZC4H2 |  |
|  | MAPK9 |  |
|  | MAP2K3 |  |
|  | YLPM1 |  |
|  | METTL3 |  |
|  | KCNK13 |  |
|  | MAP3K7CL |  |
|  | FAM20C |  |
|  | CHPT1 |  |
|  | PLSCR4 |  |
|  | MIR409 |  |
|  | NLN |  |
|  | KCNT1 |  |
|  | DPP10 |  |
|  | PTN |  |
|  | PURB |  |
|  | PVALB |  |
|  | CXCL16 |  |
|  | RHOU |  |
|  | RAB1A |  |
|  | RARRES2 |  |
|  | CACNG6 |  |
|  | RENBP |  |
|  | RHD |  |
|  | RPE65 |  |
|  | RPL32 |  |
|  | S100A8 |  |
|  | SAT1 |  |
|  | SCN7A |  |
|  | SDC1 |  |
|  | POTEM |  |
|  | SGTA |  |
|  | GIGYF1 |  |
|  | GORASP1 |  |
|  | UBE2Z |  |
|  | SLC18A3 |  |
|  | SMPD2 |  |
|  | SUMO3 |  |
|  | SUMO2 |  |
|  | MIR539 |  |
|  | SPARC |  |
|  | SPRR2B |  |
|  | STAT5A |  |
|  | STC1 |  |
|  | STK11 |  |
|  | STRN |  |
|  | BTD |  |
|  | TEAD1 |  |
|  | TERF2 |  |
|  | TMSB4X |  |
|  | TOP2B |  |
|  | TPT1 |  |
|  | CRISP2 |  |
|  | TRAF2 |  |
|  | TRAF3 |  |
|  | C3AR1 |  |
|  | MIR650 |  |
|  | MIR652 |  |
|  | C5AR1 |  |
|  | OPN1MW2 |  |
|  | UVRAG |  |
|  | VDI |  |
|  | VSNL1 |  |
|  | WNT1 |  |
|  | WNT2 |  |
|  | XIST |  |
|  | XRCC1 |  |
|  | PCGF2 |  |
|  | ZBTB17 |  |
|  | MAP3K12 |  |
|  | SCG2 |  |
|  | SLMAP |  |
|  | MANF |  |
|  | REEP5 |  |
|  | TMEM109 |  |
|  | MMP28 |  |
|  | LAP |  |
|  | MYH14 |  |
|  | SHCBP1 |  |
|  | CAAP1 |  |
|  | DGLUCY |  |
|  | ZC3H12A |  |
|  | TCL1A |  |
|  | TXNDC5 |  |
|  | UNC93B1 |  |
|  | AKAP1 |  |
|  | CAPG |  |
|  | CAST |  |
|  | BCL2L12 |  |
|  | EVA1A |  |
|  | TCHP |  |
|  | SPZ1 |  |
|  | ATP5MD |  |
|  | ORAI1 |  |
|  | ADO |  |
|  | PLPP3 |  |
|  | STC2 |  |
|  | PSMG1 |  |
|  | URI1 |  |
|  | CBR3 |  |
|  | TNFSF10 |  |
|  | CDS2 |  |
|  | PROM1 |  |
|  | SPHK1 |  |
|  | NAE1 |  |
|  | WASF1 |  |
|  | MLIP |  |
|  | CLDN10 |  |
|  | DPP9 |  |
|  | HGS |  |
|  | MAP3K13 |  |
|  | XPR1 |  |
|  | MTG1 |  |
|  | IL32 |  |
|  | MSC |  |
|  | ORAI3 |  |
|  | CGB5 |  |
|  | TSIX |  |
|  | CGB8 |  |
|  | OPN4 |  |
|  | TNFRSF8 |  |
|  | FHL5 |  |
|  | TBPL1 |  |
|  | BCAR1 |  |
|  | AKAP12 |  |
|  | ISG15 |  |
|  | TGS1 |  |
|  | FAM53B |  |
|  | CD69 |  |
|  | MLEC |  |
|  | PIEZO1 |  |
|  | BMS1 |  |
|  | CCS |  |
|  | CVDPX |  |
|  | LVNC1 |  |
|  | SCFI |  |
|  | JDSCD |  |
|  | HDCA |  |
|  | POF1 |  |
|  | PFHB1A |  |
|  | CHTD2 |  |
|  | ATRST1 |  |
|  | MFM9 |  |
|  | CHDSKM |  |
|  | MUL |  |
|  | TARPS |  |
|  | MOSPGF |  |
|  | HBMS |  |
|  | CHTD3 |  |
|  | MFM1 |  |
|  | BTHS |  |
|  | SHDRA |  |
|  | CMD1Y |  |
|  | CMD1S |  |
|  | BPES |  |
|  | PFE |  |
|  | DSMA1 |  |
|  | ODG2 |  |
|  | EPM4 |  |
|  | SRXY3 |  |
|  | SPGF10 |  |
|  | SPGFY2 |  |
|  | SPGF17 |  |
|  | RFH1 |  |
|  | CMD1P |  |
|  | CMD1U |  |
|  | CMD1D |  |
|  | GRNG |  |
|  | NEDBEH |  |
|  | CMH4 |  |
|  | CDSP |  |
|  | CCHS1 |  |
|  | GPHYSD1 |  |
|  | MCOPS9 |  |
|  | CMD1I |  |
|  | JLNS1 |  |
|  | CMD1G |  |
|  | CMD1J |  |
|  | GACI2 |  |
|  | CMD2B |  |
|  | HTX1 |  |
|  | CMD1A |  |
|  | HFE2A |  |
|  | CMD2A |  |
|  | MFS |  |
|  | HHT1 |  |
|  | MTTL1 |  |
|  | DCWHK |  |
|  | CMH15 |  |
|  | BMFS6 |  |
|  | CMD1AA |  |
|  | SCFAI |  |
|  | NXD |  |
|  | RSMD1 |  |
|  | SAV1 |  |
|  | CMH1 |  |
|  | GACI1 |  |
|  | MELAS |  |
|  | MMDD |  |
|  | PPH5 |  |
|  | CMD1R |  |
|  | CMD1M |  |
|  | CMD1DD |  |
|  | CMD2F |  |
|  | CMD1E |  |
|  | PVOD1 |  |
|  | CMD1Z |  |
|  | CMD1O |  |
|  | CMD1HH |  |
|  | AOS1 |  |
|  | PFHB1B |  |
|  | CHIME |  |
|  | MCOPS2 |  |
|  | HLHS1 |  |
|  | CHTD6 |  |
|  | HLHS2 |  |
|  | CHDS6 |  |
|  | CHDTHP |  |
|  | CTHM |  |
|  | CHDS7 |  |
|  | TACHD |  |
|  | CHTD4 |  |
|  | CHOPS |  |
|  | HOS |  |
|  | SDDHD |  |
|  | CHDFIDD |  |
|  | CHDED |  |
|  | CHTD5 |  |
|  | DCHE |  |
|  | CHTD7 |  |
|  | CHTD8 |  |
|  | CHDS8 |  |
|  | CHDS9 |  |
|  | LACHT |  |
|  | CRIP1 |  |
|  | CA-II |  |
|  | D2R |  |
|  | MR |  |
|  | V1AR |  |
|  | NET |  |
|  | V2R |  |
|  | MERS |  |
|  | ADRA1D |  |
|  | ENaC |  |
|  | SPT ATPase |  |
|  | TN-C |  |
|  | ASIC1 |  |
|  | SGLT2 |  |
|  | GUCY2D |  |
|  | GCS |  |
|  | SLCO4C1 |  |
|  | CYM |  |
|  | OPRL1 |  |
|  | oral |  |
|  | ADORA1 mRNA |  |
|  | GUCY1B1 |  |
|  | VIPR2 |  |
|  | SGLT1 |  |
|  | HSP70 |  |
|  | Pro-NRG1 |  |
|  | RXFP1 |  |
|  | mTORC1 |  |
|  | NKCC |  |
|  | CACNA1E |  |
|  | NaC |  |
|  | ADRA1 |  |
|  | PDE3 |  |
|  | CaC |  |
|  | VR |  |
|  | PDE |  |
|  | ECE |  |
|  | T-cells |  |
|  | MIR92 |  |
|  | ADCY |  |
|  | CLASP1 |  |
|  | KCNIP4 |  |
|  | CYP4A11 |  |

|  |  | Table S7. Herbs-ingredients–target genes-disease network statistics. | | | | | | | | | | | | | | | | | |
| --- | --- | --- | --- | --- | --- | --- | --- | --- | --- | --- | --- | --- | --- | --- | --- | --- | --- | --- | --- |
| AverageShortestPathLength | BetweennessCentrality | ClosenessCentrality | ClusteringCoefficient | Degree | degree.layout | Eccentricity | IsSingleNode | name | NeighborhoodConnectivity | NumberOfDirectedEdges | NumberOfUndirectedEdges | PartnerOfMultiEdgedNodePairs | Radiality | selected | SelfLoops | shared name | Stress | TopologicalCoefficient | Type |
| 1.213568 | 0.66282 | 0.824016563 | 0 | 320 |  | 4 | FALSE | Heart Failure | 4.821875 | 0 | 320 | 0 | 0.999332601 | FALSE | 0 | HF | 1310444 | 0.053081597 | HF |
| 2.113065 | 0.086087 | 0.473246136 | 0 | 284 | 284 | 4 | FALSE | GD1 | 6.118881119 | 0 | 284 | 141 | 0.996521671 | FALSE | 0 | GD1 | 330608 | 0.073126873 | GD |
| 2.379397 | 0.030139 | 0.420274551 | 0 | 172 | 172 | 4 | FALSE | GD2 | 7.397727273 | 0 | 172 | 84 | 0.995689384 | FALSE | 0 | GD2 | 167226 | 0.088857323 | GD |
| 2.580402 | 0.013124 | 0.387536514 | 0 | 93 | 93 | 4 | FALSE | GD3 | 11.79166667 | 0 | 93 | 45 | 0.995061244 | FALSE | 0 | GD3 | 81608 | 0.149884259 | GD |
| 2.444724 | 0.02306 | 0.409044193 | 0 | 79 | 79 | 4 | FALSE | GZ1 | 7.202531646 | 0 | 79 | 0 | 0.995485239 | FALSE | 0 | GZ1 | 121312 | 0.091213701 | GZ |
| 2.595477 | 0.020931 | 0.385285576 | 0 | 76 |  | 4 | FALSE | BP2 | 4.424657534 | 0 | 76 | 3 | 0.995014133 | FALSE | 0 | BP2 | 90830 | 0.077833126 | BP |
| 2.48995 | 0.01744 | 0.401614531 | 0 | 67 | 67 | 4 | FALSE | GZ2 | 7.955223881 | 0 | 67 | 0 | 0.995343907 | FALSE | 0 | GZ2 | 101328 | 0.0979609 | GZ |
| 2.5 | 0.024935 | 0.4 | 0 | 67 |  | 4 | FALSE | BP1 | 6.253731343 | 0 | 67 | 0 | 0.9953125 | FALSE | 0 | BP1 | 107938 | 0.076141034 | BP |
| 2.595477 | 0.015789 | 0.385285576 | 0 | 66 | 66 | 4 | FALSE | DX3 | 6.196969697 | 0 | 66 | 0 | 0.995014133 | FALSE | 0 | DX3 | 66038 | 0.101901367 | DX |
| 1.874372 | 0.076065 | 0.533512064 | 0 | 65 |  | 3 | FALSE | PTGS2 | 22.41935484 | 0 | 65 | 3 | 0.997267588 | FALSE | 0 | PTGS2 | 241536 | 0.06610912 | gene |
| 2.610553 | 0.012464 | 0.383060635 | 0 | 64 | 64 | 4 | FALSE | GZ3 | 5.453125 | 0 | 64 | 0 | 0.994967023 | FALSE | 0 | GZ3 | 46506 | 0.0890625 | GZ |
| 3.130653 | 0.018147 | 0.319422151 | 0 | 58 |  | 4 | FALSE | Danshen | 8.637931034 | 0 | 58 | 0 | 0.993341709 | FALSE | 0 | Danshen | 61662 | 0.097922193 | DRUG |
| 2.595477 | 0.010469 | 0.385285576 | 0 | 48 | 48 | 4 | FALSE | GZ4 | 7.875 | 0 | 48 | 0 | 0.995014133 | FALSE | 0 | GZ4 | 44038 | 0.099637681 | GZ |
| 2.610553 | 0.010021 | 0.383060635 | 0 | 42 | 42 | 4 | FALSE | DX4 | 12.5952381 | 0 | 42 | 0 | 0.994967023 | FALSE | 0 | DX4 | 60878 | 0.161044974 | DX |
| 2.625628 | 0.008549 | 0.380861244 | 0 | 39 | 39 | 4 | FALSE | DS8 | 9.794871795 | 0 | 39 | 0 | 0.994919912 | FALSE | 0 | DS8 | 59446 | 0.122150997 | DS |
| 2 | 0.018533 | 0.5 | 0 | 39 |  | 3 | FALSE | SCN5A | 30.2972973 | 0 | 39 | 2 | 0.996875 | FALSE | 0 | SCN5A | 112456 | 0.090423757 | gene |
| 2.01005 | 0.017966 | 0.4975 | 0 | 38 |  | 3 | FALSE | PTGS1 | 33.74285714 | 0 | 38 | 3 | 0.996843593 | FALSE | 0 | PTGS1 | 104906 | 0.101058201 | gene |
| 2.020101 | 0.013574 | 0.495024876 | 0 | 36 |  | 4 | FALSE | ADRB2 | 28.76470588 | 0 | 36 | 2 | 0.996812186 | FALSE | 0 | ADRB2 | 81314 | 0.085958842 | gene |
| 2.035176 | 0.020357 | 0.491358025 | 0 | 34 |  | 4 | FALSE | ACHE | 28.74193548 | 0 | 34 | 3 | 0.996765075 | FALSE | 0 | ACHE | 77974 | 0.085888345 | gene |
| 2.045226 | 0.014216 | 0.488943489 | 0 | 31 |  | 3 | FALSE | AR | 40.03571429 | 0 | 31 | 3 | 0.996733668 | FALSE | 0 | AR | 84562 | 0.1204806 | gene |
| 2.045226 | 0.008942 | 0.488943489 | 0 | 31 |  | 4 | FALSE | OPRM1 | 23.73333333 | 0 | 31 | 1 | 0.996733668 | FALSE | 0 | OPRM1 | 46720 | 0.070600414 | gene |
| 2.045226 | 0.012806 | 0.488943489 | 0 | 30 |  | 4 | FALSE | RXRA | 32.27586207 | 0 | 30 | 1 | 0.996733668 | FALSE | 0 | RXRA | 68592 | 0.096829294 | gene |
| 2.670854 | 0.006625 | 0.374412041 | 0 | 29 | 29 | 3 | FALSE | DS55 | 15.65517241 | 0 | 29 | 0 | 0.99477858 | FALSE | 0 | DS55 | 45014 | 0.200755786 | DS |
| 2.055276 | 0.011851 | 0.486552567 | 0 | 29 |  | 4 | FALSE | ESR1 | 33.7037037 | 0 | 29 | 2 | 0.996702261 | FALSE | 0 | ESR1 | 63658 | 0.101249857 | gene |
| 2.055276 | 0.007366 | 0.486552567 | 0 | 29 |  | 4 | FALSE | ADRA1A | 26.28571429 | 0 | 29 | 1 | 0.996702261 | FALSE | 0 | ADRA1A | 44058 | 0.078527063 | gene |
| 2.055276 | 0.009734 | 0.486552567 | 0 | 28 |  | 4 | FALSE | CHRM3 | 28.17857143 | 0 | 28 | 0 | 0.996702261 | FALSE | 0 | CHRM3 | 58436 | 0.084405501 | gene |
| 2.100503 | 0.005248 | 0.476076555 | 0 | 21 |  | 4 | FALSE | OPRD1 | 27.76190476 | 0 | 21 | 0 | 0.99656093 | FALSE | 0 | OPRD1 | 26940 | 0.083630952 | gene |
| 2.726131 | 0.002508 | 0.366820276 | 0 | 18 | 18 | 3 | FALSE | DS29 | 21.94444444 | 0 | 18 | 0 | 0.994605842 | FALSE | 0 | DS29 | 26152 | 0.286910198 | DS |
| 2.731156 | 0.001727 | 0.366145354 | 0 | 17 | 17 | 3 | FALSE | DS51 | 24.05882353 | 0 | 17 | 0 | 0.994590138 | FALSE | 0 | DS51 | 20876 | 0.315874295 | DS |
| 2.731156 | 0.00102 | 0.366145354 | 0 | 17 | 17 | 3 | FALSE | DS33 | 28.23529412 | 0 | 17 | 0 | 0.994590138 | FALSE | 0 | DS33 | 24292 | 0.373086221 | DS |
| 2.736181 | 0.000852 | 0.365472911 | 0 | 16 | 16 | 3 | FALSE | DS43 | 28.6875 | 0 | 16 | 0 | 0.994574435 | FALSE | 0 | DS43 | 21858 | 0.379280822 | DS |
| 2.736181 | 0.000996 | 0.365472911 | 0 | 16 | 16 | 3 | FALSE | DS13 | 28.0625 | 0 | 16 | 0 | 0.994574435 | FALSE | 0 | DS13 | 20894 | 0.370719178 | DS |
| 2.115578 | 0.003675 | 0.472684086 | 0 | 16 |  | 3 | FALSE | GSK3B | 65.14285714 | 0 | 16 | 2 | 0.996513819 | FALSE | 0 | GSK3B | 33170 | 0.197971781 | gene |
| 2.120603 | 0.003193 | 0.471563981 | 0 | 16 |  | 3 | FALSE | ESR2 | 72.46153846 | 0 | 16 | 3 | 0.996498116 | FALSE | 0 | ESR2 | 28398 | 0.220560304 | gene |
| 2.115578 | 0.010719 | 0.472684086 | 0 | 16 |  | 3 | FALSE | PGR | 51.71428571 | 0 | 16 | 2 | 0.996513819 | FALSE | 0 | PGR | 33646 | 0.156525573 | gene |
| 2.741206 | 0.000745 | 0.364802933 | 0 | 15 | 15 | 3 | FALSE | DS38 | 30.06666667 | 0 | 15 | 0 | 0.994558731 | FALSE | 0 | DS38 | 21186 | 0.398173516 | DS |
| 2.741206 | 0.000736 | 0.364802933 | 0 | 15 | 15 | 3 | FALSE | DS37 | 31 | 0 | 15 | 0 | 0.994558731 | FALSE | 0 | DS37 | 20204 | 0.410958904 | DS |
| 2.746231 | 0.000643 | 0.364135407 | 0 | 14 | 14 | 3 | FALSE | DS39 | 31.57142857 | 0 | 14 | 0 | 0.994543028 | FALSE | 0 | DS39 | 19338 | 0.418786693 | DS |
| 2.130653 | 0.001291 | 0.469339623 | 0 | 14 |  | 3 | FALSE | CASP3 | 92.36363636 | 0 | 14 | 3 | 0.996466709 | FALSE | 0 | CASP3 | 27228 | 0.281986532 | gene |
| 2.125628 | 0.002556 | 0.470449173 | 0 | 14 |  | 4 | FALSE | SLC6A4 | 46.53846154 | 0 | 14 | 1 | 0.996482412 | FALSE | 0 | SLC6A4 | 18706 | 0.140985949 | gene |
| 2.751256 | 0.000473 | 0.36347032 | 0 | 13 | 13 | 3 | FALSE | DS42 | 32.76923077 | 0 | 13 | 0 | 0.994527324 | FALSE | 0 | DS42 | 16532 | 0.435194942 | DS |
| 2.751256 | 0.000533 | 0.36347032 | 0 | 13 | 13 | 3 | FALSE | DS35 | 31.38461538 | 0 | 13 | 0 | 0.994527324 | FALSE | 0 | DS35 | 15666 | 0.416227608 | DS |
| 2.751256 | 0.000473 | 0.36347032 | 0 | 13 | 13 | 3 | FALSE | DS32 | 33.15384615 | 0 | 13 | 0 | 0.994527324 | FALSE | 0 | DS32 | 17410 | 0.440463646 | DS |
| 2.756281 | 0.000728 | 0.362807657 | 0 | 13 | 13 | 4 | FALSE | DS30 | 29.61538462 | 0 | 13 | 0 | 0.994511621 | FALSE | 0 | DS30 | 18724 | 0.397435897 | DS |
| 2.751256 | 0.000497 | 0.36347032 | 0 | 13 | 13 | 3 | FALSE | DS10 | 32.15384615 | 0 | 13 | 0 | 0.994527324 | FALSE | 0 | DS10 | 16626 | 0.426765016 | DS |
| 2.751256 | 0.000563 | 0.36347032 | 0 | 13 | 13 | 3 | FALSE | DS1 | 30 | 0 | 13 | 0 | 0.994527324 | FALSE | 0 | DS1 | 15044 | 0.397260274 | DS |
| 2.130653 | 0.001111 | 0.469339623 | 0 | 13 |  | 3 | FALSE | MMP2 | 95.81818182 | 0 | 13 | 2 | 0.996466709 | FALSE | 0 | MMP2 | 26160 | 0.292648709 | gene |
| 2.756281 | 0.000388 | 0.362807657 | 0 | 12 | 12 | 3 | FALSE | DS31 | 34.33333333 | 0 | 12 | 0 | 0.994511621 | FALSE | 0 | DS31 | 14820 | 0.456621005 | DS |
| 2.145729 | 0.000782 | 0.466042155 | 0 | 12 |  | 4 | FALSE | BCL2 | 100.4444444 | 0 | 12 | 3 | 0.996419598 | FALSE | 0 | BCL2 | 16636 | 0.307877537 | gene |
| 2.130653 | 0.005809 | 0.469339623 | 0 | 12 |  | 3 | FALSE | NR3C1 | 52.90909091 | 0 | 12 | 1 | 0.996466709 | FALSE | 0 | NR3C1 | 21374 | 0.160213244 | gene |
| 2.130653 | 0.002491 | 0.469339623 | 0 | 12 |  | 3 | FALSE | NOS2 | 57.90909091 | 0 | 12 | 1 | 0.996466709 | FALSE | 0 | NOS2 | 17672 | 0.175645342 | gene |
| 2.140704 | 0.00093 | 0.46713615 | 0 | 12 |  | 4 | FALSE | MMP9 | 94.3 | 0 | 12 | 2 | 0.996435302 | FALSE | 0 | MMP9 | 19592 | 0.288854489 | gene |
| 2.761307 | 0.000323 | 0.362147407 | 0 | 11 | 11 | 3 | FALSE | DS17 | 33.72727273 | 0 | 11 | 0 | 0.994495917 | FALSE | 0 | DS17 | 12172 | 0.448318804 | DS |
| 2.761307 | 0.000325 | 0.362147407 | 0 | 11 | 11 | 3 | FALSE | DS6 | 34.90909091 | 0 | 11 | 0 | 0.994495917 | FALSE | 0 | DS6 | 13294 | 0.464508095 | DS |
| 2.155779 | 0.002416 | 0.463869464 | 0 | 11 |  | 4 | FALSE | CYP19A1 | 98.75 | 0 | 11 | 3 | 0.996388191 | FALSE | 0 | CYP19A1 | 13988 | 0.303571429 | gene |
| 2.145729 | 0.000881 | 0.466042155 | 0 | 11 |  | 4 | FALSE | APP | 98.22222222 | 0 | 11 | 2 | 0.996419598 | FALSE | 0 | APP | 16904 | 0.300997592 | gene |
| 2.477387 | 0.003298 | 0.403651116 | 0 | 10 |  | 4 | FALSE | Guangzao | 55 | 0 | 10 | 0 | 0.995383166 | FALSE | 0 | Guangzao | 16070 | 0.2109375 | DRUG |
| 2.766332 | 0.000255 | 0.361489555 | 0 | 10 | 10 | 3 | FALSE | DS45 | 37.2 | 0 | 10 | 0 | 0.994480214 | FALSE | 0 | DS45 | 11674 | 0.495890411 | DS |
| 2.766332 | 0.000257 | 0.361489555 | 0 | 10 | 10 | 3 | FALSE | DS21 | 36.4 | 0 | 10 | 0 | 0.994480214 | FALSE | 0 | DS21 | 11254 | 0.484931507 | DS |
| 2.766332 | 0.000255 | 0.361489555 | 0 | 10 | 10 | 3 | FALSE | DS9 | 37.2 | 0 | 10 | 0 | 0.994480214 | FALSE | 0 | DS9 | 11674 | 0.495890411 | DS |
| 2.766332 | 0.000344 | 0.361489555 | 0 | 10 | 10 | 3 | FALSE | DS5 | 33.5 | 0 | 10 | 0 | 0.994480214 | FALSE | 0 | DS5 | 10460 | 0.445205479 | DS |
| 2.150754 | 0.00056 | 0.464953271 | 0 | 10 |  | 4 | FALSE | ADORA1 | 112.5 | 0 | 10 | 2 | 0.996403894 | FALSE | 0 | ADORA1 | 14886 | 0.345201238 | gene |
| 2.145729 | 0.000546 | 0.466042155 | 0 | 10 |  | 3 | FALSE | MET | 107.125 | 0 | 10 | 2 | 0.996419598 | FALSE | 0 | MET | 14084 | 0.327546296 | gene |
| 2.145729 | 0.000546 | 0.466042155 | 0 | 10 |  | 3 | FALSE | INSR | 107.125 | 0 | 10 | 2 | 0.996419598 | FALSE | 0 | INSR | 14084 | 0.327546296 | gene |
| 2.145729 | 0.001186 | 0.466042155 | 0 | 10 |  | 3 | FALSE | PPARG | 101.125 | 0 | 10 | 2 | 0.996419598 | FALSE | 0 | PPARG | 13530 | 0.309027778 | gene |
| 2.150754 | 0.000751 | 0.464953271 | 0 | 10 |  | 4 | FALSE | RELA | 95.5 | 0 | 10 | 2 | 0.996403894 | FALSE | 0 | RELA | 12666 | 0.292569659 | gene |
| 2.155779 | 0.000403 | 0.463869464 | 0 | 9 |  | 4 | FALSE | KDR | 116.8571429 | 0 | 9 | 2 | 0.996388191 | FALSE | 0 | KDR | 11024 | 0.358690845 | gene |
| 2.155779 | 0.000403 | 0.463869464 | 0 | 9 |  | 4 | FALSE | SRC | 116.8571429 | 0 | 9 | 2 | 0.996388191 | FALSE | 0 | SRC | 11024 | 0.358690845 | gene |
| 2.155779 | 0.000396 | 0.463869464 | 0 | 9 |  | 4 | FALSE | BACE1 | 116 | 0 | 9 | 2 | 0.996388191 | FALSE | 0 | BACE1 | 10274 | 0.356037152 | gene |
| 2.160804 | 0.000404 | 0.462790698 | 0 | 9 |  | 4 | FALSE | JUN | 111.1666667 | 0 | 9 | 3 | 0.996372487 | FALSE | 0 | JUN | 8058 | 0.341073271 | gene |
| 2.150754 | 0.000757 | 0.464953271 | 0 | 9 |  | 4 | FALSE | BCL2L1 | 99.5 | 0 | 9 | 1 | 0.996403894 | FALSE | 0 | BCL2L1 | 13730 | 0.30495356 | gene |
| 2.145729 | 0.000839 | 0.466042155 | 0 | 9 |  | 3 | FALSE | CCND1 | 96.375 | 0 | 9 | 1 | 0.996419598 | FALSE | 0 | CCND1 | 14216 | 0.294367284 | gene |
| 2.155779 | 0.001401 | 0.463869464 | 0 | 9 |  | 4 | FALSE | SLC6A3 | 51.88888889 | 0 | 9 | 0 | 0.996388191 | FALSE | 0 | SLC6A3 | 9676 | 0.158532364 | gene |
| 2.633166 | 0.002422 | 0.379770992 | 0 | 8 |  | 4 | FALSE | Dingxiang | 50.25 | 0 | 8 | 0 | 0.994896357 | FALSE | 0 | Dingxiang | 11376 | 0.216960352 | DRUG |
| 2.796482 | 0.000312 | 0.357592093 | 0 | 8 | 8 | 4 | FALSE | DX2 | 29.5 | 0 | 8 | 0 | 0.994385992 | FALSE | 0 | DX2 | 4616 | 0.413043478 | DX |
| 2.781407 | 0.000164 | 0.359530262 | 0 | 8 | 8 | 4 | FALSE | DS56 | 37.625 | 0 | 8 | 0 | 0.994433103 | FALSE | 0 | DS56 | 7926 | 0.508680556 | DS |
| 2.791457 | 0.000163 | 0.358235824 | 0 | 8 | 8 | 4 | FALSE | DS16 | 36.625 | 0 | 8 | 0 | 0.994401696 | FALSE | 0 | DS16 | 7508 | 0.508928571 | DS |
| 2.165829 | 0.000251 | 0.461716937 | 0 | 8 |  | 4 | FALSE | CA5A | 124.1666667 | 0 | 8 | 2 | 0.996356784 | FALSE | 0 | CA5A | 6858 | 0.382505176 | gene |
| 2.165829 | 0.000251 | 0.461716937 | 0 | 8 |  | 4 | FALSE | IGF1R | 124.1666667 | 0 | 8 | 2 | 0.996356784 | FALSE | 0 | IGF1R | 6858 | 0.382505176 | gene |
| 2.165829 | 0.000251 | 0.461716937 | 0 | 8 |  | 4 | FALSE | CA12 | 124.1666667 | 0 | 8 | 2 | 0.996356784 | FALSE | 0 | CA12 | 6858 | 0.382505176 | gene |
| 2.165829 | 0.000251 | 0.461716937 | 0 | 8 |  | 4 | FALSE | CA2 | 124.1666667 | 0 | 8 | 2 | 0.996356784 | FALSE | 0 | CA2 | 6858 | 0.382505176 | gene |
| 2.160804 | 0.000296 | 0.462790698 | 0 | 8 |  | 4 | FALSE | SYK | 128.3333333 | 0 | 8 | 2 | 0.996372487 | FALSE | 0 | SYK | 9028 | 0.394220846 | gene |
| 2.160804 | 0.000296 | 0.462790698 | 0 | 8 |  | 4 | FALSE | MMP13 | 128.3333333 | 0 | 8 | 2 | 0.996372487 | FALSE | 0 | MMP13 | 9028 | 0.394220846 | gene |
| 2.155779 | 0.00048 | 0.463869464 | 0 | 8 |  | 4 | FALSE | PIK3CA | 105.1428571 | 0 | 8 | 1 | 0.996388191 | FALSE | 0 | PIK3CA | 11114 | 0.322423706 | gene |
| 2.160804 | 0.000288 | 0.462790698 | 0 | 8 |  | 4 | FALSE | MMP3 | 127.3333333 | 0 | 8 | 2 | 0.996372487 | FALSE | 0 | MMP3 | 8360 | 0.391124871 | gene |
| 2.160804 | 0.000349 | 0.462790698 | 0 | 8 |  | 4 | FALSE | TERT | 124.6666667 | 0 | 8 | 2 | 0.996372487 | FALSE | 0 | TERT | 8796 | 0.382868937 | gene |
| 2.160804 | 0.000288 | 0.462790698 | 0 | 8 |  | 4 | FALSE | PLA2G1B | 127.3333333 | 0 | 8 | 2 | 0.996372487 | FALSE | 0 | PLA2G1B | 8360 | 0.391124871 | gene |
| 2.160804 | 0.00052 | 0.462790698 | 0 | 8 |  | 4 | FALSE | PTPN1 | 93.57142857 | 0 | 8 | 1 | 0.996372487 | FALSE | 0 | PTPN1 | 8784 | 0.287488909 | gene |
| 2.160804 | 0.000334 | 0.462790698 | 0 | 8 |  | 4 | FALSE | CYP3A4 | 121 | 0 | 8 | 2 | 0.996372487 | FALSE | 0 | CYP3A4 | 7832 | 0.371517028 | gene |
| 2.160804 | 0.001268 | 0.462790698 | 0 | 8 |  | 4 | FALSE | KCNH2 | 89.66666667 | 0 | 8 | 2 | 0.996372487 | FALSE | 0 | KCNH2 | 7032 | 0.274509804 | gene |
| 2.160804 | 0.000293 | 0.462790698 | 0 | 8 |  | 4 | FALSE | GSTP1 | 119.5 | 0 | 8 | 2 | 0.996372487 | FALSE | 0 | GSTP1 | 7470 | 0.366873065 | gene |
| 2.155779 | 0.000507 | 0.463869464 | 0 | 8 |  | 3 | FALSE | TNF | 112.5 | 0 | 8 | 2 | 0.996388191 | FALSE | 0 | TNF | 8918 | 0.344135802 | gene |
| 2.160804 | 0.000293 | 0.462790698 | 0 | 8 |  | 4 | FALSE | AKT1 | 119.5 | 0 | 8 | 2 | 0.996372487 | FALSE | 0 | AKT1 | 7470 | 0.366873065 | gene |
| 2.155779 | 0.000357 | 0.463869464 | 0 | 8 |  | 3 | FALSE | EGFR | 118.5 | 0 | 8 | 2 | 0.996388191 | FALSE | 0 | EGFR | 8976 | 0.362654321 | gene |
| 2.781407 | 0.000149 | 0.359530262 | 0 | 7 | 7 | 3 | FALSE | DS44 | 40.57142857 | 0 | 7 | 0 | 0.994433103 | FALSE | 0 | DS44 | 7706 | 0.542074364 | DS |
| 2.811558 | 0.000122 | 0.35567471 | 0 | 7 | 7 | 4 | FALSE | DS36 | 35.14285714 | 0 | 7 | 0 | 0.994338882 | FALSE | 0 | DS36 | 6168 | 0.509594883 | DS |
| 2.786432 | 0.000138 | 0.358881876 | 0 | 7 | 7 | 4 | FALSE | DS34 | 40.42857143 | 0 | 7 | 0 | 0.994417399 | FALSE | 0 | DS34 | 7760 | 0.547619048 | DS |
| 2.786432 | 0.000138 | 0.358881876 | 0 | 7 | 7 | 4 | FALSE | DS11 | 39 | 0 | 7 | 0 | 0.994417399 | FALSE | 0 | DS11 | 7014 | 0.527777778 | DS |
| 2.170854 | 0.000162 | 0.460648148 | 0 | 7 |  | 4 | FALSE | MMP12 | 139.4 | 0 | 7 | 2 | 0.99634108 | FALSE | 0 | MMP12 | 5252 | 0.429813665 | gene |
| 2.170854 | 0.000162 | 0.460648148 | 0 | 7 |  | 4 | FALSE | CA3 | 139.4 | 0 | 7 | 2 | 0.99634108 | FALSE | 0 | CA3 | 5252 | 0.429813665 | gene |
| 2.170854 | 0.000217 | 0.460648148 | 0 | 7 |  | 4 | FALSE | ADORA2A | 136.2 | 0 | 7 | 2 | 0.99634108 | FALSE | 0 | ADORA2A | 5746 | 0.419875776 | gene |
| 2.170854 | 0.000162 | 0.460648148 | 0 | 7 |  | 4 | FALSE | ALOX12 | 139.4 | 0 | 7 | 2 | 0.99634108 | FALSE | 0 | ALOX12 | 5252 | 0.429813665 | gene |
| 2.170854 | 0.000217 | 0.460648148 | 0 | 7 |  | 4 | FALSE | ALOX15 | 136.2 | 0 | 7 | 2 | 0.99634108 | FALSE | 0 | ALOX15 | 5746 | 0.419875776 | gene |
| 2.170854 | 0.000162 | 0.460648148 | 0 | 7 |  | 4 | FALSE | ABCG2 | 139.4 | 0 | 7 | 2 | 0.99634108 | FALSE | 0 | ABCG2 | 5252 | 0.429813665 | gene |
| 2.170854 | 0.000162 | 0.460648148 | 0 | 7 |  | 4 | FALSE | ESRRA | 139.4 | 0 | 7 | 2 | 0.99634108 | FALSE | 0 | ESRRA | 5252 | 0.429813665 | gene |
| 2.170854 | 0.000162 | 0.460648148 | 0 | 7 |  | 4 | FALSE | ABCC1 | 139.4 | 0 | 7 | 2 | 0.99634108 | FALSE | 0 | ABCC1 | 5252 | 0.429813665 | gene |
| 2.170854 | 0.000162 | 0.460648148 | 0 | 7 |  | 4 | FALSE | NOX4 | 139.4 | 0 | 7 | 2 | 0.99634108 | FALSE | 0 | NOX4 | 5252 | 0.429813665 | gene |
| 2.170854 | 0.000243 | 0.460648148 | 0 | 7 |  | 4 | FALSE | MAOA | 128.2 | 0 | 7 | 2 | 0.99634108 | FALSE | 0 | MAOA | 5098 | 0.395031056 | gene |
| 2.170854 | 0.000162 | 0.460648148 | 0 | 7 |  | 4 | FALSE | CYP1B1 | 139.4 | 0 | 7 | 2 | 0.99634108 | FALSE | 0 | CYP1B1 | 5252 | 0.429813665 | gene |
| 2.170854 | 0.000256 | 0.460648148 | 0 | 7 |  | 4 | FALSE | PRKCA | 128.2 | 0 | 7 | 2 | 0.99634108 | FALSE | 0 | PRKCA | 5460 | 0.395031056 | gene |
| 2.165829 | 0.000239 | 0.461716937 | 0 | 7 |  | 4 | FALSE | CSNK2A1 | 134.4 | 0 | 7 | 2 | 0.996356784 | FALSE | 0 | CSNK2A1 | 6512 | 0.413003096 | gene |
| 2.165829 | 0.000239 | 0.461716937 | 0 | 7 |  | 4 | FALSE | PTK2 | 134.4 | 0 | 7 | 2 | 0.996356784 | FALSE | 0 | PTK2 | 6512 | 0.413003096 | gene |
| 2.165829 | 0.000236 | 0.461716937 | 0 | 7 |  | 4 | FALSE | ALOX5 | 133.2 | 0 | 7 | 2 | 0.996356784 | FALSE | 0 | ALOX5 | 6014 | 0.409287926 | gene |
| 2.165829 | 0.000284 | 0.461716937 | 0 | 7 |  | 4 | FALSE | PPARD | 124 | 0 | 7 | 2 | 0.996356784 | FALSE | 0 | PPARD | 5698 | 0.380804954 | gene |
| 2.160804 | 0.000355 | 0.462790698 | 0 | 7 |  | 4 | FALSE | SHBG | 103.8333333 | 0 | 7 | 1 | 0.996372487 | FALSE | 0 | SHBG | 7220 | 0.318369453 | gene |
| 2.165829 | 0.000388 | 0.461716937 | 0 | 7 |  | 4 | FALSE | RORC | 101.1666667 | 0 | 7 | 1 | 0.996356784 | FALSE | 0 | RORC | 6802 | 0.311076605 | gene |
| 2.165829 | 0.00041 | 0.461716937 | 0 | 7 |  | 4 | FALSE | SLC6A2 | 103 | 0 | 7 | 2 | 0.996356784 | FALSE | 0 | SLC6A2 | 5090 | 0.315789474 | gene |
| 2.160804 | 0.000252 | 0.462790698 | 0 | 7 |  | 3 | FALSE | MMP1 | 131.4 | 0 | 7 | 2 | 0.996372487 | FALSE | 0 | MMP1 | 6608 | 0.402469136 | gene |
| 2.165829 | 0.000222 | 0.461716937 | 0 | 7 |  | 4 | FALSE | XDH | 127.6 | 0 | 7 | 2 | 0.996356784 | FALSE | 0 | XDH | 5488 | 0.391950464 | gene |
| 2.155779 | 0.000394 | 0.463869464 | 0 | 7 |  | 3 | FALSE | MAPK1 | 120.1666667 | 0 | 7 | 1 | 0.996388191 | FALSE | 0 | MAPK1 | 9838 | 0.367798354 | gene |
| 2.160804 | 0.000303 | 0.462790698 | 0 | 7 |  | 4 | FALSE | VEGFA | 116 | 0 | 7 | 1 | 0.996372487 | FALSE | 0 | VEGFA | 7240 | 0.356037152 | gene |
| 2.165829 | 0.000901 | 0.461716937 | 0 | 7 |  | 4 | FALSE | DRD2 | 71 | 0 | 7 | 1 | 0.996356784 | FALSE | 0 | DRD2 | 6038 | 0.217391304 | gene |
| 2.826633 | 0.000255 | 0.353777778 | 0 | 6 | 6 | 4 | FALSE | GZ5 | 24.33333333 | 0 | 6 | 0 | 0.994291771 | FALSE | 0 | GZ5 | 2116 | 0.358974359 | GZ |
| 2.791457 | 0.00013 | 0.358235824 | 0 | 6 | 6 | 4 | FALSE | DS57 | 41.16666667 | 0 | 6 | 0 | 0.994401696 | FALSE | 0 | DS57 | 7446 | 0.55787037 | DS |
| 2.806533 | 0.000203 | 0.356311549 | 0 | 6 | 6 | 4 | FALSE | DS50 | 34.16666667 | 0 | 6 | 0 | 0.994354585 | FALSE | 0 | DS50 | 6192 | 0.480676329 | DS |
| 2.796482 | 0.00016 | 0.357592093 | 0 | 6 | 6 | 4 | FALSE | DS46 | 36.83333333 | 0 | 6 | 0 | 0.994385992 | FALSE | 0 | DS46 | 6862 | 0.504694836 | DS |
| 2.796482 | 0.000165 | 0.357592093 | 0 | 6 | 6 | 4 | FALSE | DS28 | 37.16666667 | 0 | 6 | 0 | 0.994385992 | FALSE | 0 | DS28 | 7340 | 0.509389671 | DS |
| 2.791457 | 0.000212 | 0.358235824 | 0 | 6 | 6 | 4 | FALSE | DS27 | 37.83333333 | 0 | 6 | 0 | 0.994401696 | FALSE | 0 | DS27 | 6740 | 0.511574074 | DS |
| 2.801508 | 0.000104 | 0.356950673 | 0 | 6 | 6 | 4 | FALSE | DS25 | 39.33333333 | 0 | 6 | 0 | 0.994370289 | FALSE | 0 | DS25 | 5228 | 0.547619048 | DS |
| 2.796482 | 0.000173 | 0.357592093 | 0 | 6 | 6 | 4 | FALSE | DS14 | 25.16666667 | 0 | 6 | 0 | 0.994385992 | FALSE | 0 | DS14 | 6622 | 0.340375587 | DS |
| 2.884422 | 4.2E-05 | 0.346689895 | 0 | 6 |  | 4 | FALSE | HSD17B1 | 94.25 | 0 | 6 | 2 | 0.994111181 | FALSE | 0 | HSD17B1 | 1238 | 0.51519337 | gene |
| 2.175879 | 0.000126 | 0.459584296 | 0 | 6 |  | 4 | FALSE | GPR35 | 149.75 | 0 | 6 | 2 | 0.996325377 | FALSE | 0 | GPR35 | 3494 | 0.461956522 | gene |
| 2.170854 | 0.000247 | 0.460648148 | 0 | 6 |  | 4 | FALSE | BCHE | 111.2 | 0 | 6 | 1 | 0.99634108 | FALSE | 0 | BCHE | 4830 | 0.342236025 | gene |
| 2.170854 | 0.000229 | 0.460648148 | 0 | 6 |  | 4 | FALSE | MAOB | 130.2 | 0 | 6 | 1 | 0.99634108 | FALSE | 0 | MAOB | 5270 | 0.401242236 | gene |
| 2.175879 | 0.000126 | 0.459584296 | 0 | 6 |  | 4 | FALSE | GSTM1 | 149.75 | 0 | 6 | 2 | 0.996325377 | FALSE | 0 | GSTM1 | 3494 | 0.461956522 | gene |
| 2.170854 | 0.00015 | 0.460648148 | 0 | 6 |  | 4 | FALSE | ABCB1 | 156 | 0 | 6 | 2 | 0.99634108 | FALSE | 0 | ABCB1 | 4892 | 0.479876161 | gene |
| 2.170854 | 0.00015 | 0.460648148 | 0 | 6 |  | 4 | FALSE | PIK3R1 | 156 | 0 | 6 | 2 | 0.99634108 | FALSE | 0 | PIK3R1 | 4892 | 0.479876161 | gene |
| 2.170854 | 0.00015 | 0.460648148 | 0 | 6 |  | 4 | FALSE | PARP1 | 156 | 0 | 6 | 2 | 0.99634108 | FALSE | 0 | PARP1 | 4892 | 0.479876161 | gene |
| 2.170854 | 0.000147 | 0.460648148 | 0 | 6 |  | 4 | FALSE | PIM1 | 154.5 | 0 | 6 | 2 | 0.99634108 | FALSE | 0 | PIM1 | 4476 | 0.475232198 | gene |
| 2.170854 | 0.000181 | 0.460648148 | 0 | 6 |  | 4 | FALSE | CASP8 | 144.5 | 0 | 6 | 2 | 0.99634108 | FALSE | 0 | CASP8 | 4248 | 0.444272446 | gene |
| 2.165829 | 0.000271 | 0.461716937 | 0 | 6 |  | 4 | FALSE | HMGCR | 111.2 | 0 | 6 | 1 | 0.996356784 | FALSE | 0 | HMGCR | 5254 | 0.341176471 | gene |
| 2.170854 | 0.000191 | 0.460648148 | 0 | 6 |  | 4 | FALSE | NR1I2 | 145 | 0 | 6 | 2 | 0.99634108 | FALSE | 0 | NR1I2 | 4200 | 0.445820433 | gene |
| 2.170854 | 0.000191 | 0.460648148 | 0 | 6 |  | 4 | FALSE | CYP1A1 | 145 | 0 | 6 | 2 | 0.99634108 | FALSE | 0 | CYP1A1 | 4200 | 0.445820433 | gene |
| 2.170854 | 0.000143 | 0.460648148 | 0 | 6 |  | 4 | FALSE | ICAM1 | 147.5 | 0 | 6 | 2 | 0.99634108 | FALSE | 0 | ICAM1 | 4080 | 0.453560372 | gene |
| 2.170854 | 0.000143 | 0.460648148 | 0 | 6 |  | 4 | FALSE | HMOX1 | 147.5 | 0 | 6 | 2 | 0.99634108 | FALSE | 0 | HMOX1 | 4080 | 0.453560372 | gene |
| 2.160804 | 0.00028 | 0.462790698 | 0 | 6 |  | 3 | FALSE | ERBB2 | 124.6 | 0 | 6 | 1 | 0.996372487 | FALSE | 0 | ERBB2 | 6498 | 0.381481481 | gene |
| 2.165829 | 0.000309 | 0.461716937 | 0 | 6 |  | 4 | FALSE | NFKBIA | 115.8 | 0 | 6 | 1 | 0.996356784 | FALSE | 0 | NFKBIA | 5628 | 0.355417957 | gene |
| 2.165829 | 0.000309 | 0.461716937 | 0 | 6 |  | 4 | FALSE | CDKN1A | 115.8 | 0 | 6 | 1 | 0.996356784 | FALSE | 0 | CDKN1A | 5628 | 0.355417957 | gene |
| 2.160804 | 0.004128 | 0.462790698 | 0 | 6 |  | 4 | FALSE | NR3C2 | 73.33333333 | 0 | 6 | 0 | 0.996372487 | FALSE | 0 | NR3C2 | 9968 | 0.223942208 | gene |
| 2.175879 | 0.0009 | 0.459584296 | 0 | 6 |  | 4 | FALSE | PDE3A | 66 | 0 | 6 | 0 | 0.996325377 | FALSE | 0 | PDE3A | 5700 | 0.203125 | gene |
| 2.811558 | 0.000158 | 0.35567471 | 0 | 5 | 5 | 4 | FALSE | DS40 | 35.2 | 0 | 5 | 0 | 0.994338882 | FALSE | 0 | DS40 | 4856 | 0.495652174 | DS |
| 2.811558 | 0.000155 | 0.35567471 | 0 | 5 | 5 | 4 | FALSE | DS26 | 35 | 0 | 5 | 0 | 0.994338882 | FALSE | 0 | DS26 | 4486 | 0.492753623 | DS |
| 2.811558 | 0.000237 | 0.35567471 | 0 | 5 | 5 | 4 | FALSE | DS19 | 30.2 | 0 | 5 | 0 | 0.994338882 | FALSE | 0 | DS19 | 4568 | 0.423188406 | DS |
| 2.175879 | 0.000113 | 0.459584296 | 0 | 5 |  | 4 | FALSE | SERPINE1 | 152.25 | 0 | 5 | 1 | 0.996325377 | FALSE | 0 | SERPINE1 | 3560 | 0.469720497 | gene |
| 2.175879 | 0.000113 | 0.459584296 | 0 | 5 |  | 4 | FALSE | IGFBP3 | 152.25 | 0 | 5 | 1 | 0.996325377 | FALSE | 0 | IGFBP3 | 3560 | 0.469720497 | gene |
| 2.175879 | 0.000113 | 0.459584296 | 0 | 5 |  | 4 | FALSE | F3 | 152.25 | 0 | 5 | 1 | 0.996325377 | FALSE | 0 | F3 | 3560 | 0.469720497 | gene |
| 2.170854 | 0.000222 | 0.460648148 | 0 | 5 |  | 4 | FALSE | VCP | 119.2 | 0 | 5 | 0 | 0.99634108 | FALSE | 0 | VCP | 5212 | 0.367080745 | gene |
| 2.170854 | 0.000222 | 0.460648148 | 0 | 5 |  | 4 | FALSE | CTSB | 119.2 | 0 | 5 | 0 | 0.99634108 | FALSE | 0 | CTSB | 5212 | 0.367080745 | gene |
| 2.180905 | 5.59E-05 | 0.458525346 | 0 | 5 |  | 4 | FALSE | MAPT | 183.6666667 | 0 | 5 | 2 | 0.996309673 | FALSE | 0 | MAPT | 2264 | 0.567287785 | gene |
| 2.180905 | 5.59E-05 | 0.458525346 | 0 | 5 |  | 4 | FALSE | AXL | 183.6666667 | 0 | 5 | 2 | 0.996309673 | FALSE | 0 | AXL | 2264 | 0.567287785 | gene |
| 2.180905 | 5.59E-05 | 0.458525346 | 0 | 5 |  | 4 | FALSE | ALK | 183.6666667 | 0 | 5 | 2 | 0.996309673 | FALSE | 0 | ALK | 2264 | 0.567287785 | gene |
| 2.180905 | 5.59E-05 | 0.458525346 | 0 | 5 |  | 4 | FALSE | CXCR1 | 183.6666667 | 0 | 5 | 2 | 0.996309673 | FALSE | 0 | CXCR1 | 2264 | 0.567287785 | gene |
| 2.180905 | 5.59E-05 | 0.458525346 | 0 | 5 |  | 4 | FALSE | PKN1 | 183.6666667 | 0 | 5 | 2 | 0.996309673 | FALSE | 0 | PKN1 | 2264 | 0.567287785 | gene |
| 2.180905 | 5.59E-05 | 0.458525346 | 0 | 5 |  | 4 | FALSE | PYGL | 183.6666667 | 0 | 5 | 2 | 0.996309673 | FALSE | 0 | PYGL | 2264 | 0.567287785 | gene |
| 2.180905 | 5.59E-05 | 0.458525346 | 0 | 5 |  | 4 | FALSE | MPO | 183.6666667 | 0 | 5 | 2 | 0.996309673 | FALSE | 0 | MPO | 2264 | 0.567287785 | gene |
| 2.180905 | 5.59E-05 | 0.458525346 | 0 | 5 |  | 4 | FALSE | DRD4 | 183.6666667 | 0 | 5 | 2 | 0.996309673 | FALSE | 0 | DRD4 | 2264 | 0.567287785 | gene |
| 2.180905 | 5.59E-05 | 0.458525346 | 0 | 5 |  | 4 | FALSE | F2 | 183.6666667 | 0 | 5 | 2 | 0.996309673 | FALSE | 0 | F2 | 2264 | 0.567287785 | gene |
| 2.180905 | 5.59E-05 | 0.458525346 | 0 | 5 |  | 4 | FALSE | AVPR2 | 183.6666667 | 0 | 5 | 2 | 0.996309673 | FALSE | 0 | AVPR2 | 2264 | 0.567287785 | gene |
| 2.180905 | 5.59E-05 | 0.458525346 | 0 | 5 |  | 4 | FALSE | TTR | 183.6666667 | 0 | 5 | 2 | 0.996309673 | FALSE | 0 | TTR | 2264 | 0.567287785 | gene |
| 2.180905 | 5.59E-05 | 0.458525346 | 0 | 5 |  | 4 | FALSE | SLC22A12 | 183.6666667 | 0 | 5 | 2 | 0.996309673 | FALSE | 0 | SLC22A12 | 2264 | 0.567287785 | gene |
| 2.180905 | 5.59E-05 | 0.458525346 | 0 | 5 |  | 4 | FALSE | ARG1 | 183.6666667 | 0 | 5 | 2 | 0.996309673 | FALSE | 0 | ARG1 | 2264 | 0.567287785 | gene |
| 2.180905 | 5.59E-05 | 0.458525346 | 0 | 5 |  | 4 | FALSE | VCAM1 | 183.6666667 | 0 | 5 | 2 | 0.996309673 | FALSE | 0 | VCAM1 | 2264 | 0.567287785 | gene |
| 2.180905 | 5.59E-05 | 0.458525346 | 0 | 5 |  | 4 | FALSE | SELE | 183.6666667 | 0 | 5 | 2 | 0.996309673 | FALSE | 0 | SELE | 2264 | 0.567287785 | gene |
| 2.175879 | 0.000147 | 0.459584296 | 0 | 5 |  | 4 | FALSE | MAPK8 | 134.5 | 0 | 5 | 1 | 0.996325377 | FALSE | 0 | MAPK8 | 3566 | 0.414596273 | gene |
| 2.180905 | 9.52E-05 | 0.458525346 | 0 | 5 |  | 4 | FALSE | PON1 | 170.3333333 | 0 | 5 | 2 | 0.996309673 | FALSE | 0 | PON1 | 2266 | 0.525879917 | gene |
| 2.180905 | 9.52E-05 | 0.458525346 | 0 | 5 |  | 4 | FALSE | TGFB1 | 170.3333333 | 0 | 5 | 2 | 0.996309673 | FALSE | 0 | TGFB1 | 2266 | 0.525879917 | gene |
| 2.175879 | 0.00019 | 0.459584296 | 0 | 5 |  | 4 | FALSE | CYP17A1 | 119.25 | 0 | 5 | 1 | 0.996325377 | FALSE | 0 | CYP17A1 | 3214 | 0.367236025 | gene |
| 2.175879 | 0.00019 | 0.459584296 | 0 | 5 |  | 4 | FALSE | NPC1L1 | 119.25 | 0 | 5 | 1 | 0.996325377 | FALSE | 0 | NPC1L1 | 3214 | 0.367236025 | gene |
| 2.175879 | 0.00019 | 0.459584296 | 0 | 5 |  | 4 | FALSE | NR1H3 | 119.25 | 0 | 5 | 1 | 0.996325377 | FALSE | 0 | NR1H3 | 3214 | 0.367236025 | gene |
| 2.170854 | 0.000152 | 0.460648148 | 0 | 5 |  | 4 | FALSE | PPARA | 152.25 | 0 | 5 | 1 | 0.99634108 | FALSE | 0 | PPARA | 4384 | 0.468266254 | gene |
| 2.175879 | 0.00019 | 0.459584296 | 0 | 5 |  | 4 | FALSE | G6PD | 119.25 | 0 | 5 | 1 | 0.996325377 | FALSE | 0 | G6PD | 3214 | 0.367236025 | gene |
| 2.175879 | 0.00019 | 0.459584296 | 0 | 5 |  | 4 | FALSE | HSD11B2 | 119.25 | 0 | 5 | 1 | 0.996325377 | FALSE | 0 | HSD11B2 | 3214 | 0.367236025 | gene |
| 2.175879 | 0.00019 | 0.459584296 | 0 | 5 |  | 4 | FALSE | HSD11B1 | 119.25 | 0 | 5 | 1 | 0.996325377 | FALSE | 0 | HSD11B1 | 3214 | 0.367236025 | gene |
| 2.165829 | 0.000305 | 0.461716937 | 0 | 5 |  | 4 | FALSE | EDNRA | 112.4 | 0 | 5 | 0 | 0.996356784 | FALSE | 0 | EDNRA | 5840 | 0.344891641 | gene |
| 2.170854 | 0.003732 | 0.460648148 | 0 | 5 |  | 4 | FALSE | F7 | 138.25 | 0 | 5 | 1 | 0.99634108 | FALSE | 0 | F7 | 6192 | 0.424922601 | gene |
| 2.170854 | 0.00029 | 0.460648148 | 0 | 5 |  | 4 | FALSE | BIRC5 | 130 | 0 | 5 | 1 | 0.99634108 | FALSE | 0 | BIRC5 | 4206 | 0.399380805 | gene |
| 2.170854 | 0.000223 | 0.460648148 | 0 | 5 |  | 4 | FALSE | TP53 | 132.75 | 0 | 5 | 1 | 0.99634108 | FALSE | 0 | TP53 | 4208 | 0.407894737 | gene |
| 2.165829 | 0.000186 | 0.461716937 | 0 | 5 |  | 3 | FALSE | IL6 | 142.25 | 0 | 5 | 1 | 0.996356784 | FALSE | 0 | IL6 | 4496 | 0.43595679 | gene |
| 2.816583 | 0.000505 | 0.355040143 | 0 | 4 |  | 5 | FALSE | GXST | 19.75 | 0 | 4 | 0 | 0.994323178 | FALSE | 0 | GXST | 4370 | 0.260416667 | GXST |
| 2.806533 | 6.86E-05 | 0.356311549 | 0 | 4 | 4 | 4 | FALSE | DS47 | 43.75 | 0 | 4 | 0 | 0.994354585 | FALSE | 0 | DS47 | 4188 | 0.602112676 | DS |
| 2.806533 | 6.66E-05 | 0.356311549 | 0 | 4 | 4 | 4 | FALSE | DS15 | 44.25 | 0 | 4 | 0 | 0.994354585 | FALSE | 0 | DS15 | 4154 | 0.60915493 | DS |
| 2.180905 | 6.24E-05 | 0.458525346 | 0 | 4 |  | 4 | FALSE | SOD1 | 180.6666667 | 0 | 4 | 1 | 0.996309673 | FALSE | 0 | SOD1 | 2252 | 0.557971014 | gene |
| 2.180905 | 0.000136 | 0.458525346 | 0 | 4 |  | 4 | FALSE | SNCA | 128.5 | 0 | 4 | 0 | 0.996309673 | FALSE | 0 | SNCA | 3038 | 0.397196262 | gene |
| 2.180905 | 8.19E-05 | 0.458525346 | 0 | 4 |  | 4 | FALSE | PRKCB | 170.3333333 | 0 | 4 | 1 | 0.996309673 | FALSE | 0 | PRKCB | 2028 | 0.525879917 | gene |
| 2.180905 | 8.19E-05 | 0.458525346 | 0 | 4 |  | 4 | FALSE | CXCL8 | 170.3333333 | 0 | 4 | 1 | 0.996309673 | FALSE | 0 | CXCL8 | 2028 | 0.525879917 | gene |
| 2.18593 | 0.000117 | 0.457471264 | 0 | 4 |  | 4 | FALSE | DHCR7 | 136.6666667 | 0 | 4 | 1 | 0.99629397 | FALSE | 0 | DHCR7 | 1720 | 0.422637591 | gene |
| 2.18593 | 0.000117 | 0.457471264 | 0 | 4 |  | 4 | FALSE | NR1H2 | 136.6666667 | 0 | 4 | 1 | 0.99629397 | FALSE | 0 | NR1H2 | 1720 | 0.422637591 | gene |
| 2.18593 | 0.000117 | 0.457471264 | 0 | 4 |  | 4 | FALSE | VDR | 136.6666667 | 0 | 4 | 1 | 0.99629397 | FALSE | 0 | VDR | 1720 | 0.422637591 | gene |
| 2.18593 | 0.000117 | 0.457471264 | 0 | 4 |  | 4 | FALSE | CHRM2 | 136.6666667 | 0 | 4 | 1 | 0.99629397 | FALSE | 0 | CHRM2 | 1720 | 0.422637591 | gene |
| 2.18593 | 0.000117 | 0.457471264 | 0 | 4 |  | 4 | FALSE | CYP2C19 | 136.6666667 | 0 | 4 | 1 | 0.99629397 | FALSE | 0 | CYP2C19 | 1720 | 0.422637591 | gene |
| 2.180905 | 0.000107 | 0.458525346 | 0 | 4 |  | 4 | FALSE | PLAU | 168.3333333 | 0 | 4 | 1 | 0.996309673 | FALSE | 0 | PLAU | 2144 | 0.519668737 | gene |
| 2.175879 | 0.000155 | 0.459584296 | 0 | 4 |  | 4 | FALSE | FGFR1 | 134.75 | 0 | 4 | 0 | 0.996325377 | FALSE | 0 | FGFR1 | 4134 | 0.415372671 | gene |
| 2.175879 | 0.000155 | 0.459584296 | 0 | 4 |  | 4 | FALSE | PIK3CB | 134.75 | 0 | 4 | 0 | 0.996325377 | FALSE | 0 | PIK3CB | 4134 | 0.415372671 | gene |
| 2.175879 | 0.000155 | 0.459584296 | 0 | 4 |  | 4 | FALSE | LCK | 134.75 | 0 | 4 | 0 | 0.996325377 | FALSE | 0 | LCK | 4134 | 0.415372671 | gene |
| 2.175879 | 0.000158 | 0.459584296 | 0 | 4 |  | 4 | FALSE | TSPO | 130.75 | 0 | 4 | 0 | 0.996325377 | FALSE | 0 | TSPO | 3362 | 0.402950311 | gene |
| 2.175879 | 0.000158 | 0.459584296 | 0 | 4 |  | 4 | FALSE | PDE4A | 130.75 | 0 | 4 | 0 | 0.996325377 | FALSE | 0 | PDE4A | 3362 | 0.402950311 | gene |
| 2.175879 | 9.67E-05 | 0.459584296 | 0 | 4 |  | 4 | FALSE | CFTR | 160.3333333 | 0 | 4 | 1 | 0.996325377 | FALSE | 0 | CFTR | 2854 | 0.493292054 | gene |
| 2.175879 | 0.000158 | 0.459584296 | 0 | 4 |  | 4 | FALSE | ELANE | 130.75 | 0 | 4 | 0 | 0.996325377 | FALSE | 0 | ELANE | 3362 | 0.402950311 | gene |
| 2.175879 | 0.000155 | 0.459584296 | 0 | 4 |  | 4 | FALSE | KIT | 134.75 | 0 | 4 | 0 | 0.996325377 | FALSE | 0 | KIT | 4134 | 0.415372671 | gene |
| 2.175879 | 0.000158 | 0.459584296 | 0 | 4 |  | 4 | FALSE | CAPN1 | 130.75 | 0 | 4 | 0 | 0.996325377 | FALSE | 0 | CAPN1 | 3362 | 0.402950311 | gene |
| 2.175879 | 0.000158 | 0.459584296 | 0 | 4 |  | 4 | FALSE | FKBP1A | 130.75 | 0 | 4 | 0 | 0.996325377 | FALSE | 0 | FKBP1A | 3362 | 0.402950311 | gene |
| 2.175879 | 0.000158 | 0.459584296 | 0 | 4 |  | 4 | FALSE | TRPV1 | 130.75 | 0 | 4 | 0 | 0.996325377 | FALSE | 0 | TRPV1 | 3362 | 0.402950311 | gene |
| 2.175879 | 0.000158 | 0.459584296 | 0 | 4 |  | 4 | FALSE | CTSK | 130.75 | 0 | 4 | 0 | 0.996325377 | FALSE | 0 | CTSK | 3362 | 0.402950311 | gene |
| 2.175879 | 9.32E-05 | 0.459584296 | 0 | 4 |  | 4 | FALSE | PIK3CG | 178.6666667 | 0 | 4 | 1 | 0.996325377 | FALSE | 0 | PIK3CG | 2930 | 0.5500516 | gene |
| 2.175879 | 0.000158 | 0.459584296 | 0 | 4 |  | 4 | FALSE | MAPK14 | 130.75 | 0 | 4 | 0 | 0.996325377 | FALSE | 0 | MAPK14 | 3362 | 0.402950311 | gene |
| 2.175879 | 0.000158 | 0.459584296 | 0 | 4 |  | 4 | FALSE | PDE5A | 130.75 | 0 | 4 | 0 | 0.996325377 | FALSE | 0 | PDE5A | 3362 | 0.402950311 | gene |
| 2.175879 | 0.000158 | 0.459584296 | 0 | 4 |  | 4 | FALSE | CRHR1 | 130.75 | 0 | 4 | 0 | 0.996325377 | FALSE | 0 | CRHR1 | 3362 | 0.402950311 | gene |
| 2.175879 | 0.000158 | 0.459584296 | 0 | 4 |  | 4 | FALSE | HCRTR2 | 130.75 | 0 | 4 | 0 | 0.996325377 | FALSE | 0 | HCRTR2 | 3362 | 0.402950311 | gene |
| 2.175879 | 0.000158 | 0.459584296 | 0 | 4 |  | 4 | FALSE | PDE10A | 130.75 | 0 | 4 | 0 | 0.996325377 | FALSE | 0 | PDE10A | 3362 | 0.402950311 | gene |
| 2.175879 | 0.000158 | 0.459584296 | 0 | 4 |  | 4 | FALSE | ADORA2B | 130.75 | 0 | 4 | 0 | 0.996325377 | FALSE | 0 | ADORA2B | 3362 | 0.402950311 | gene |
| 2.175879 | 0.000167 | 0.459584296 | 0 | 4 |  | 4 | FALSE | CCR1 | 129.25 | 0 | 4 | 0 | 0.996325377 | FALSE | 0 | CCR1 | 3362 | 0.398291925 | gene |
| 2.180905 | 0.000112 | 0.458525346 | 0 | 4 |  | 4 | FALSE | FDFT1 | 145 | 0 | 4 | 1 | 0.996309673 | FALSE | 0 | FDFT1 | 2136 | 0.447204969 | gene |
| 2.175879 | 0.000167 | 0.459584296 | 0 | 4 |  | 4 | FALSE | GPBAR1 | 129.25 | 0 | 4 | 0 | 0.996325377 | FALSE | 0 | GPBAR1 | 3362 | 0.398291925 | gene |
| 2.175879 | 0.000167 | 0.459584296 | 0 | 4 |  | 4 | FALSE | AGTR1 | 129.25 | 0 | 4 | 0 | 0.996325377 | FALSE | 0 | AGTR1 | 3362 | 0.398291925 | gene |
| 2.175879 | 0.000137 | 0.459584296 | 0 | 4 |  | 4 | FALSE | MYC | 164 | 0 | 4 | 1 | 0.996325377 | FALSE | 0 | MYC | 2596 | 0.504643963 | gene |
| 2.175879 | 0.000613 | 0.459584296 | 0 | 4 |  | 4 | FALSE | FASN | 108.5 | 0 | 4 | 0 | 0.996325377 | FALSE | 0 | FASN | 3764 | 0.333850932 | gene |
| 2.175879 | 0.000137 | 0.459584296 | 0 | 4 |  | 4 | FALSE | FOS | 164 | 0 | 4 | 1 | 0.996325377 | FALSE | 0 | FOS | 2596 | 0.504643963 | gene |
| 2.180905 | 0.000415 | 0.458525346 | 0 | 4 |  | 4 | FALSE | ADRA2A | 98.75 | 0 | 4 | 0 | 0.996309673 | FALSE | 0 | ADRA2A | 3250 | 0.304517134 | gene |
| 2.175879 | 9.63E-05 | 0.459584296 | 0 | 4 |  | 4 | FALSE | CD40LG | 167.3333333 | 0 | 4 | 1 | 0.996325377 | FALSE | 0 | CD40LG | 2514 | 0.51496388 | gene |
| 2.175879 | 9.63E-05 | 0.459584296 | 0 | 4 |  | 4 | FALSE | IFNG | 167.3333333 | 0 | 4 | 1 | 0.996325377 | FALSE | 0 | IFNG | 2514 | 0.51496388 | gene |
| 2.175879 | 9.63E-05 | 0.459584296 | 0 | 4 |  | 4 | FALSE | IL2 | 167.3333333 | 0 | 4 | 1 | 0.996325377 | FALSE | 0 | IL2 | 2514 | 0.51496388 | gene |
| 2.175879 | 9.63E-05 | 0.459584296 | 0 | 4 |  | 4 | FALSE | RB1 | 167.3333333 | 0 | 4 | 1 | 0.996325377 | FALSE | 0 | RB1 | 2514 | 0.51496388 | gene |
| 2.175879 | 9.63E-05 | 0.459584296 | 0 | 4 |  | 4 | FALSE | IL10 | 167.3333333 | 0 | 4 | 1 | 0.996325377 | FALSE | 0 | IL10 | 2514 | 0.51496388 | gene |
| 3.120603 | 0.000938 | 0.320450886 | 0 | 3 |  | 4 | FALSE | Bingpian | 48 | 0 | 3 | 0 | 0.993373116 | FALSE | 0 | Bingpian | 2264 | 0.348148148 | DRUG |
| 2.851759 | 5.51E-05 | 0.350660793 | 0 | 3 | 3 | 4 | FALSE | GZ6 | 35.66666667 | 0 | 3 | 0 | 0.994213254 | FALSE | 0 | GZ6 | 818 | 0.55026455 | GZ |
| 3.002513 | 3.28E-05 | 0.333054393 | 0 | 3 | 3 | 4 | FALSE | DX1 | 14.66666667 | 0 | 3 | 0 | 0.993742148 | FALSE | 0 | DX1 | 740 | 0.414141414 | DX |
| 2.821608 | 3.25E-05 | 0.354407836 | 0 | 3 | 3 | 4 | FALSE | DS54 | 50.33333333 | 0 | 3 | 0 | 0.994307475 | FALSE | 0 | DS54 | 2426 | 0.714975845 | DS |
| 2.821608 | 3.25E-05 | 0.354407836 | 0 | 3 | 3 | 4 | FALSE | DS53 | 50.33333333 | 0 | 3 | 0 | 0.994307475 | FALSE | 0 | DS53 | 2426 | 0.714975845 | DS |
| 2.821608 | 3.25E-05 | 0.354407836 | 0 | 3 | 3 | 4 | FALSE | DS52 | 50.33333333 | 0 | 3 | 0 | 0.994307475 | FALSE | 0 | DS52 | 2426 | 0.714975845 | DS |
| 2.831658 | 3.61E-05 | 0.353149956 | 0 | 3 | 3 | 4 | FALSE | DS41 | 38.66666667 | 0 | 3 | 0 | 0.994276068 | FALSE | 0 | DS41 | 2158 | 0.562189055 | DS |
| 2.821608 | 3.25E-05 | 0.354407836 | 0 | 3 | 3 | 4 | FALSE | DS22 | 50.33333333 | 0 | 3 | 0 | 0.994307475 | FALSE | 0 | DS22 | 2426 | 0.714975845 | DS |
| 2.821608 | 3.39E-05 | 0.354407836 | 0 | 3 | 3 | 4 | FALSE | DS20 | 49.66666667 | 0 | 3 | 0 | 0.994307475 | FALSE | 0 | DS20 | 2464 | 0.70531401 | DS |
| 2.856784 | 9.7E-05 | 0.350043975 | 0 | 3 | 3 | 4 | FALSE | DS18 | 25 | 0 | 3 | 0 | 0.99419755 | FALSE | 0 | DS18 | 1690 | 0.387096774 | DS |
| 2.846734 | 9.6E-05 | 0.351279788 | 0 | 3 | 3 | 4 | FALSE | DS2 | 26 | 0 | 3 | 0 | 0.994228957 | FALSE | 0 | DS2 | 1872 | 0.390625 | DS |
| 2.18593 | 7.09E-05 | 0.457471264 | 0 | 3 |  | 4 | FALSE | YWHAG | 155.3333333 | 0 | 3 | 0 | 0.99629397 | FALSE | 0 | YWHAG | 1946 | 0.4807892 | gene |
| 2.18593 | 2.04E-05 | 0.457471264 | 0 | 3 |  | 4 | FALSE | THBD | 231.5 | 0 | 3 | 1 | 0.99629397 | FALSE | 0 | THBD | 1006 | 0.715838509 | gene |
| 2.18593 | 2.04E-05 | 0.457471264 | 0 | 3 |  | 4 | FALSE | SULT1E1 | 231.5 | 0 | 3 | 1 | 0.99629397 | FALSE | 0 | SULT1E1 | 1006 | 0.715838509 | gene |
| 2.18593 | 7.09E-05 | 0.457471264 | 0 | 3 |  | 4 | FALSE | SLC5A2 | 155.3333333 | 0 | 3 | 0 | 0.99629397 | FALSE | 0 | SLC5A2 | 1946 | 0.4807892 | gene |
| 2.18593 | 2.04E-05 | 0.457471264 | 0 | 3 |  | 4 | FALSE | RAF1 | 231.5 | 0 | 3 | 1 | 0.99629397 | FALSE | 0 | RAF1 | 1006 | 0.715838509 | gene |
| 2.18593 | 2.04E-05 | 0.457471264 | 0 | 3 |  | 4 | FALSE | POR | 231.5 | 0 | 3 | 1 | 0.99629397 | FALSE | 0 | POR | 1006 | 0.715838509 | gene |
| 2.18593 | 2.04E-05 | 0.457471264 | 0 | 3 |  | 4 | FALSE | PLAT | 231.5 | 0 | 3 | 1 | 0.99629397 | FALSE | 0 | PLAT | 1006 | 0.715838509 | gene |
| 2.18593 | 7.09E-05 | 0.457471264 | 0 | 3 |  | 4 | FALSE | PLA2G2A | 155.3333333 | 0 | 3 | 0 | 0.99629397 | FALSE | 0 | PLA2G2A | 1946 | 0.4807892 | gene |
| 2.18593 | 7.09E-05 | 0.457471264 | 0 | 3 |  | 4 | FALSE | PGF | 155.3333333 | 0 | 3 | 0 | 0.99629397 | FALSE | 0 | PGF | 1946 | 0.4807892 | gene |
| 2.18593 | 2.04E-05 | 0.457471264 | 0 | 3 |  | 4 | FALSE | NQO1 | 231.5 | 0 | 3 | 1 | 0.99629397 | FALSE | 0 | NQO1 | 1006 | 0.715838509 | gene |
| 2.18593 | 2.04E-05 | 0.457471264 | 0 | 3 |  | 4 | FALSE | NOS3 | 231.5 | 0 | 3 | 1 | 0.99629397 | FALSE | 0 | NOS3 | 1006 | 0.715838509 | gene |
| 2.18593 | 2.04E-05 | 0.457471264 | 0 | 3 |  | 4 | FALSE | NCF1 | 231.5 | 0 | 3 | 1 | 0.99629397 | FALSE | 0 | NCF1 | 1006 | 0.715838509 | gene |
| 2.18593 | 2.04E-05 | 0.457471264 | 0 | 3 |  | 4 | FALSE | MYLK | 231.5 | 0 | 3 | 1 | 0.99629397 | FALSE | 0 | MYLK | 1006 | 0.715838509 | gene |
| 2.18593 | 2.04E-05 | 0.457471264 | 0 | 3 |  | 4 | FALSE | MGAM | 231.5 | 0 | 3 | 1 | 0.99629397 | FALSE | 0 | MGAM | 1006 | 0.715838509 | gene |
| 2.18593 | 2.04E-05 | 0.457471264 | 0 | 3 |  | 4 | FALSE | IL1B | 231.5 | 0 | 3 | 1 | 0.99629397 | FALSE | 0 | IL1B | 1006 | 0.715838509 | gene |
| 2.18593 | 2.04E-05 | 0.457471264 | 0 | 3 |  | 4 | FALSE | IL1A | 231.5 | 0 | 3 | 1 | 0.99629397 | FALSE | 0 | IL1A | 1006 | 0.715838509 | gene |
| 2.18593 | 2.04E-05 | 0.457471264 | 0 | 3 |  | 4 | FALSE | HSPB1 | 231.5 | 0 | 3 | 1 | 0.99629397 | FALSE | 0 | HSPB1 | 1006 | 0.715838509 | gene |
| 2.18593 | 2.04E-05 | 0.457471264 | 0 | 3 |  | 4 | FALSE | HSPA5 | 231.5 | 0 | 3 | 1 | 0.99629397 | FALSE | 0 | HSPA5 | 1006 | 0.715838509 | gene |
| 2.18593 | 2.04E-05 | 0.457471264 | 0 | 3 |  | 4 | FALSE | HSF1 | 231.5 | 0 | 3 | 1 | 0.99629397 | FALSE | 0 | HSF1 | 1006 | 0.715838509 | gene |
| 2.18593 | 7.09E-05 | 0.457471264 | 0 | 3 |  | 4 | FALSE | HNF4A | 155.3333333 | 0 | 3 | 0 | 0.99629397 | FALSE | 0 | HNF4A | 1946 | 0.4807892 | gene |
| 2.18593 | 2.04E-05 | 0.457471264 | 0 | 3 |  | 4 | FALSE | HIF1A | 231.5 | 0 | 3 | 1 | 0.99629397 | FALSE | 0 | HIF1A | 1006 | 0.715838509 | gene |
| 2.18593 | 8.84E-05 | 0.457471264 | 0 | 3 |  | 4 | FALSE | GSR | 149 | 0 | 3 | 0 | 0.99629397 | FALSE | 0 | GSR | 1852 | 0.46105919 | gene |
| 2.18593 | 2.04E-05 | 0.457471264 | 0 | 3 |  | 4 | FALSE | GJA1 | 231.5 | 0 | 3 | 1 | 0.99629397 | FALSE | 0 | GJA1 | 1006 | 0.715838509 | gene |
| 2.18593 | 7.09E-05 | 0.457471264 | 0 | 3 |  | 4 | FALSE | ERN1 | 155.3333333 | 0 | 3 | 0 | 0.99629397 | FALSE | 0 | ERN1 | 1946 | 0.4807892 | gene |
| 2.18593 | 2.04E-05 | 0.457471264 | 0 | 3 |  | 4 | FALSE | EGF | 231.5 | 0 | 3 | 1 | 0.99629397 | FALSE | 0 | EGF | 1006 | 0.715838509 | gene |
| 2.18593 | 7.09E-05 | 0.457471264 | 0 | 3 |  | 4 | FALSE | DYRK1A | 155.3333333 | 0 | 3 | 0 | 0.99629397 | FALSE | 0 | DYRK1A | 1946 | 0.4807892 | gene |
| 2.18593 | 2.04E-05 | 0.457471264 | 0 | 3 |  | 4 | FALSE | CXCL10 | 231.5 | 0 | 3 | 1 | 0.99629397 | FALSE | 0 | CXCL10 | 1006 | 0.715838509 | gene |
| 2.18593 | 2.04E-05 | 0.457471264 | 0 | 3 |  | 4 | FALSE | CTSD | 231.5 | 0 | 3 | 1 | 0.99629397 | FALSE | 0 | CTSD | 1006 | 0.715838509 | gene |
| 2.18593 | 2.04E-05 | 0.457471264 | 0 | 3 |  | 4 | FALSE | CRP | 231.5 | 0 | 3 | 1 | 0.99629397 | FALSE | 0 | CRP | 1006 | 0.715838509 | gene |
| 2.18593 | 2.04E-05 | 0.457471264 | 0 | 3 |  | 4 | FALSE | COL3A1 | 231.5 | 0 | 3 | 1 | 0.99629397 | FALSE | 0 | COL3A1 | 1006 | 0.715838509 | gene |
| 2.18593 | 2.04E-05 | 0.457471264 | 0 | 3 |  | 4 | FALSE | COL1A1 | 231.5 | 0 | 3 | 1 | 0.99629397 | FALSE | 0 | COL1A1 | 1006 | 0.715838509 | gene |
| 2.18593 | 2.04E-05 | 0.457471264 | 0 | 3 |  | 4 | FALSE | CHUK | 231.5 | 0 | 3 | 1 | 0.99629397 | FALSE | 0 | CHUK | 1006 | 0.715838509 | gene |
| 2.18593 | 7.09E-05 | 0.457471264 | 0 | 3 |  | 4 | FALSE | CHRNA7 | 155.3333333 | 0 | 3 | 0 | 0.99629397 | FALSE | 0 | CHRNA7 | 1946 | 0.4807892 | gene |
| 2.18593 | 2.04E-05 | 0.457471264 | 0 | 3 |  | 4 | FALSE | CHEK2 | 231.5 | 0 | 3 | 1 | 0.99629397 | FALSE | 0 | CHEK2 | 1006 | 0.715838509 | gene |
| 2.18593 | 7.09E-05 | 0.457471264 | 0 | 3 |  | 4 | FALSE | CES1 | 155.3333333 | 0 | 3 | 0 | 0.99629397 | FALSE | 0 | CES1 | 1946 | 0.4807892 | gene |
| 2.18593 | 2.04E-05 | 0.457471264 | 0 | 3 |  | 4 | FALSE | CCL2 | 231.5 | 0 | 3 | 1 | 0.99629397 | FALSE | 0 | CCL2 | 1006 | 0.715838509 | gene |
| 2.18593 | 7.09E-05 | 0.457471264 | 0 | 3 |  | 4 | FALSE | ADORA3 | 155.3333333 | 0 | 3 | 0 | 0.99629397 | FALSE | 0 | ADORA3 | 1946 | 0.4807892 | gene |
| 2.18593 | 2.04E-05 | 0.457471264 | 0 | 3 |  | 4 | FALSE | ACACA | 231.5 | 0 | 3 | 1 | 0.99629397 | FALSE | 0 | ACACA | 1006 | 0.715838509 | gene |
| 2.18593 | 8.07E-05 | 0.457471264 | 0 | 3 |  | 4 | FALSE | TACR3 | 150 | 0 | 3 | 0 | 0.99629397 | FALSE | 0 | TACR3 | 1606 | 0.464174455 | gene |
| 2.18593 | 8.07E-05 | 0.457471264 | 0 | 3 |  | 4 | FALSE | SCARB1 | 150 | 0 | 3 | 0 | 0.99629397 | FALSE | 0 | SCARB1 | 1606 | 0.464174455 | gene |
| 2.18593 | 8.07E-05 | 0.457471264 | 0 | 3 |  | 4 | FALSE | BCL2A1 | 150 | 0 | 3 | 0 | 0.99629397 | FALSE | 0 | BCL2A1 | 1606 | 0.464174455 | gene |
| 2.18593 | 8.07E-05 | 0.457471264 | 0 | 3 |  | 4 | FALSE | BAD | 150 | 0 | 3 | 0 | 0.99629397 | FALSE | 0 | BAD | 1606 | 0.464174455 | gene |
| 2.18593 | 8.07E-05 | 0.457471264 | 0 | 3 |  | 4 | FALSE | PDE4B | 150 | 0 | 3 | 0 | 0.99629397 | FALSE | 0 | PDE4B | 1606 | 0.464174455 | gene |
| 2.18593 | 8.07E-05 | 0.457471264 | 0 | 3 |  | 4 | FALSE | MTNR1B | 150 | 0 | 3 | 0 | 0.99629397 | FALSE | 0 | MTNR1B | 1606 | 0.464174455 | gene |
| 2.18593 | 8.07E-05 | 0.457471264 | 0 | 3 |  | 4 | FALSE | KCNN4 | 150 | 0 | 3 | 0 | 0.99629397 | FALSE | 0 | KCNN4 | 1606 | 0.464174455 | gene |
| 2.18593 | 8.07E-05 | 0.457471264 | 0 | 3 |  | 4 | FALSE | SCN2A | 150 | 0 | 3 | 0 | 0.99629397 | FALSE | 0 | SCN2A | 1606 | 0.464174455 | gene |
| 2.18593 | 8.07E-05 | 0.457471264 | 0 | 3 |  | 4 | FALSE | ABL1 | 150 | 0 | 3 | 0 | 0.99629397 | FALSE | 0 | ABL1 | 1606 | 0.464174455 | gene |
| 2.18593 | 8.07E-05 | 0.457471264 | 0 | 3 |  | 4 | FALSE | MMP7 | 150 | 0 | 3 | 0 | 0.99629397 | FALSE | 0 | MMP7 | 1606 | 0.464174455 | gene |
| 2.18593 | 8.07E-05 | 0.457471264 | 0 | 3 |  | 4 | FALSE | SLC2A2 | 150 | 0 | 3 | 0 | 0.99629397 | FALSE | 0 | SLC2A2 | 1606 | 0.464174455 | gene |
| 2.18593 | 8.07E-05 | 0.457471264 | 0 | 3 |  | 4 | FALSE | CMA1 | 150 | 0 | 3 | 0 | 0.99629397 | FALSE | 0 | CMA1 | 1606 | 0.464174455 | gene |
| 2.18593 | 8.07E-05 | 0.457471264 | 0 | 3 |  | 4 | FALSE | ADAM17 | 150 | 0 | 3 | 0 | 0.99629397 | FALSE | 0 | ADAM17 | 1606 | 0.464174455 | gene |
| 2.18593 | 8.07E-05 | 0.457471264 | 0 | 3 |  | 4 | FALSE | SLC2A1 | 150 | 0 | 3 | 0 | 0.99629397 | FALSE | 0 | SLC2A1 | 1606 | 0.464174455 | gene |
| 2.18593 | 8.07E-05 | 0.457471264 | 0 | 3 |  | 4 | FALSE | TRPV4 | 150 | 0 | 3 | 0 | 0.99629397 | FALSE | 0 | TRPV4 | 1606 | 0.464174455 | gene |
| 2.18593 | 8.07E-05 | 0.457471264 | 0 | 3 |  | 4 | FALSE | TRPC3 | 150 | 0 | 3 | 0 | 0.99629397 | FALSE | 0 | TRPC3 | 1606 | 0.464174455 | gene |
| 2.18593 | 8.07E-05 | 0.457471264 | 0 | 3 |  | 4 | FALSE | TRPC6 | 150 | 0 | 3 | 0 | 0.99629397 | FALSE | 0 | TRPC6 | 1606 | 0.464174455 | gene |
| 2.18593 | 8.07E-05 | 0.457471264 | 0 | 3 |  | 4 | FALSE | MEN1 | 150 | 0 | 3 | 0 | 0.99629397 | FALSE | 0 | MEN1 | 1606 | 0.464174455 | gene |
| 2.18593 | 8.07E-05 | 0.457471264 | 0 | 3 |  | 4 | FALSE | CPB1 | 150 | 0 | 3 | 0 | 0.99629397 | FALSE | 0 | CPB1 | 1606 | 0.464174455 | gene |
| 2.18593 | 8.07E-05 | 0.457471264 | 0 | 3 |  | 4 | FALSE | SCN10A | 150 | 0 | 3 | 0 | 0.99629397 | FALSE | 0 | SCN10A | 1606 | 0.464174455 | gene |
| 2.18593 | 8.07E-05 | 0.457471264 | 0 | 3 |  | 4 | FALSE | TNFRSF1A | 150 | 0 | 3 | 0 | 0.99629397 | FALSE | 0 | TNFRSF1A | 1606 | 0.464174455 | gene |
| 2.18593 | 8.07E-05 | 0.457471264 | 0 | 3 |  | 4 | FALSE | KCNA5 | 150 | 0 | 3 | 0 | 0.99629397 | FALSE | 0 | KCNA5 | 1606 | 0.464174455 | gene |
| 2.18593 | 8.07E-05 | 0.457471264 | 0 | 3 |  | 4 | FALSE | ELOVL6 | 150 | 0 | 3 | 0 | 0.99629397 | FALSE | 0 | ELOVL6 | 1606 | 0.464174455 | gene |
| 2.18593 | 8.07E-05 | 0.457471264 | 0 | 3 |  | 4 | FALSE | P2RX7 | 150 | 0 | 3 | 0 | 0.99629397 | FALSE | 0 | P2RX7 | 1606 | 0.464174455 | gene |
| 2.18593 | 8.07E-05 | 0.457471264 | 0 | 3 |  | 4 | FALSE | ACE | 150 | 0 | 3 | 0 | 0.99629397 | FALSE | 0 | ACE | 1606 | 0.464174455 | gene |
| 2.18593 | 8.07E-05 | 0.457471264 | 0 | 3 |  | 4 | FALSE | CTSS | 150 | 0 | 3 | 0 | 0.99629397 | FALSE | 0 | CTSS | 1606 | 0.464174455 | gene |
| 2.18593 | 8.07E-05 | 0.457471264 | 0 | 3 |  | 4 | FALSE | PRKCD | 150 | 0 | 3 | 0 | 0.99629397 | FALSE | 0 | PRKCD | 1606 | 0.464174455 | gene |
| 2.190955 | 5.35E-05 | 0.456422018 | 0 | 3 |  | 4 | FALSE | UGT2B7 | 184 | 0 | 3 | 1 | 0.996278266 | FALSE | 0 | UGT2B7 | 902 | 0.570093458 | gene |
| 2.190955 | 5.35E-05 | 0.456422018 | 0 | 3 |  | 4 | FALSE | SHH | 184 | 0 | 3 | 1 | 0.996278266 | FALSE | 0 | SHH | 902 | 0.570093458 | gene |
| 2.18593 | 3.08E-05 | 0.457471264 | 0 | 3 |  | 4 | FALSE | PPP3CA | 204 | 0 | 3 | 1 | 0.99629397 | FALSE | 0 | PPP3CA | 1110 | 0.630434783 | gene |
| 2.18593 | 3.08E-05 | 0.457471264 | 0 | 3 |  | 4 | FALSE | IKBKB | 204 | 0 | 3 | 1 | 0.99629397 | FALSE | 0 | IKBKB | 1110 | 0.630434783 | gene |
[truncated: 425,325 more chars]
