# Supplementary material for: Integrating network pharmacology and experimental verification to explore the pharmacological mechanisms of Guanxin Shutong capsule in treating heart failure
Source: Medicine (Baltimore). 2024 Oct 18;103(42):e40118. doi: 10.1097/MD.0000000000040118 (PMC11495747; doi:10.1097/MD.0000000000040118)

Figure S1. Calculated RMSF values for Macromolecule Cα atoms.

(A)VEGFR2-Kaempferol complex. The red line represents VEGFR2. (B) eNOS-Quercetin complex. The blue line represents eNOS. (C) VEGFR2-(2R)-5,7-dihydroxy-2-(4-hydroxyphenyl) chroman-4-one complex. The green line represents VEGFR2. (D) AKT-Ellagic acid complex. The yellow line represents AKT.


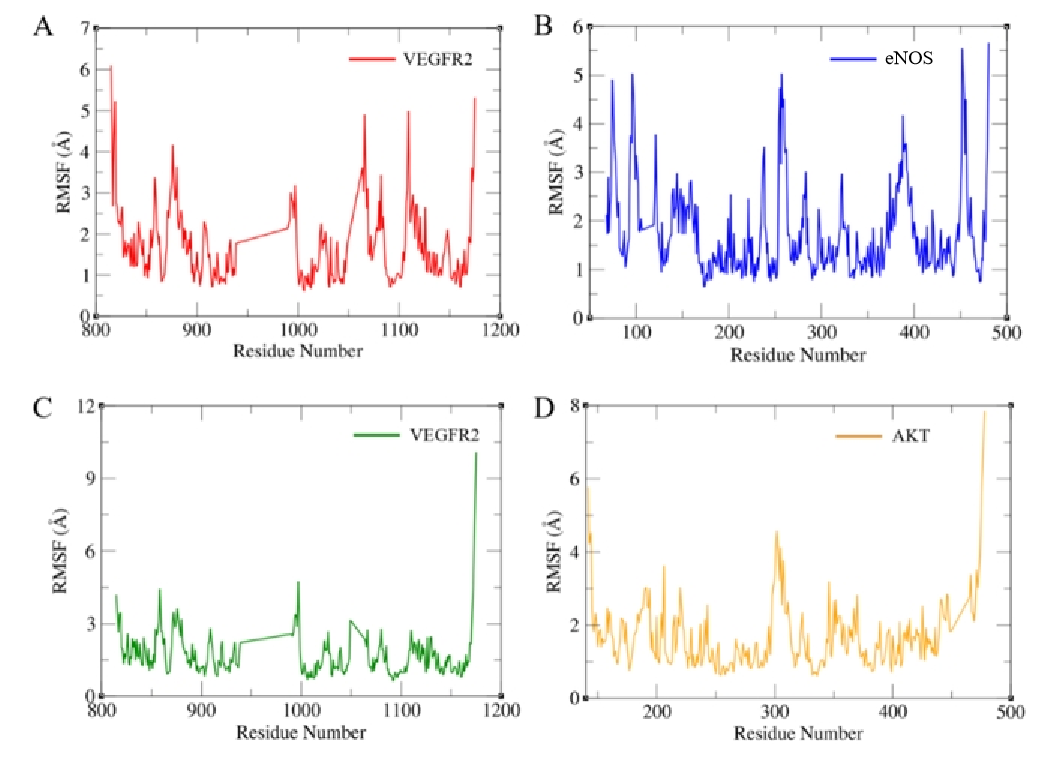


Figure S2. The changes of hydrogen bonds number between ligand and protein during 50 ns MD simulations trajectory.

1. VEGFR2-Kaempferol complex. (B) eNOS-Quercetin complex. (C) VEGFR2-(2R)-5,7-dihydroxy-2-(4-hydroxyphenyl) chroman-4-one complex. (D) AKT-Ellagic acid complex.


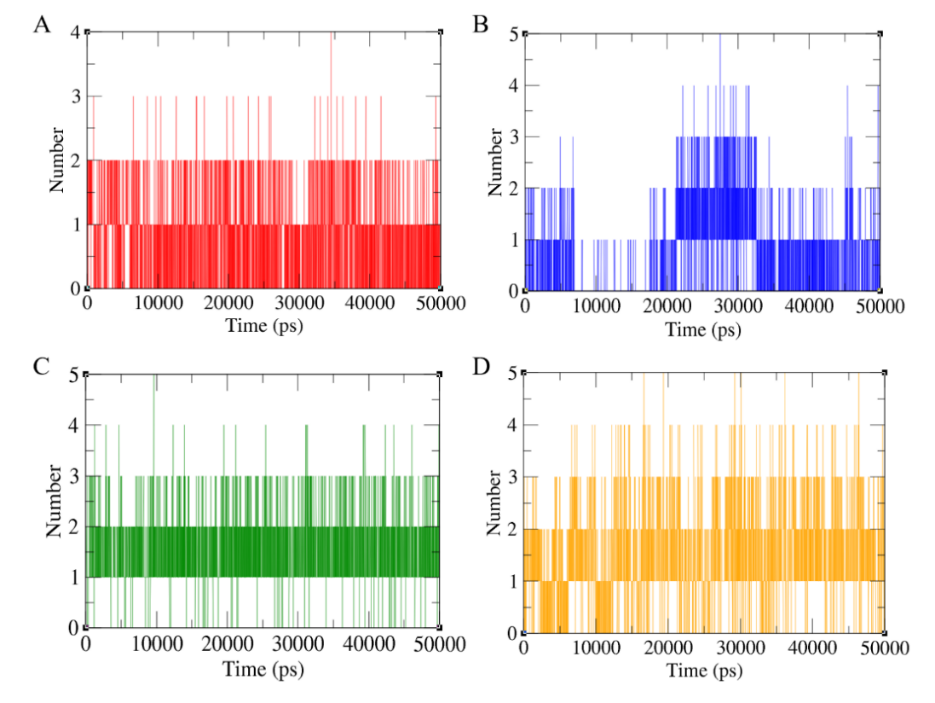


Figure S3. Protective effects of GXST on H2O2-induced apoptosis in the H9C2 cells.

1. Cell viability of H9C2 cells stimulated with different concentrations of H2O2 (100, 200, 400 and 800 μM) for 24h, n = 5. (B) Cell viability of H9C2 cells induced with various concentrations of GXST (100, 200, 300 and 400 μg/μL) for 24 h, n = 5. (C) Cell viability of H2O2-induced injury following treatment with GXST extract at different concentrations (100, 200, 300, 400μg/μL), n = 5. (D-E) Representative western blotting pictures and quantitative analysis of BAX, cleaved caspase 3 and caspase 3, n =3.


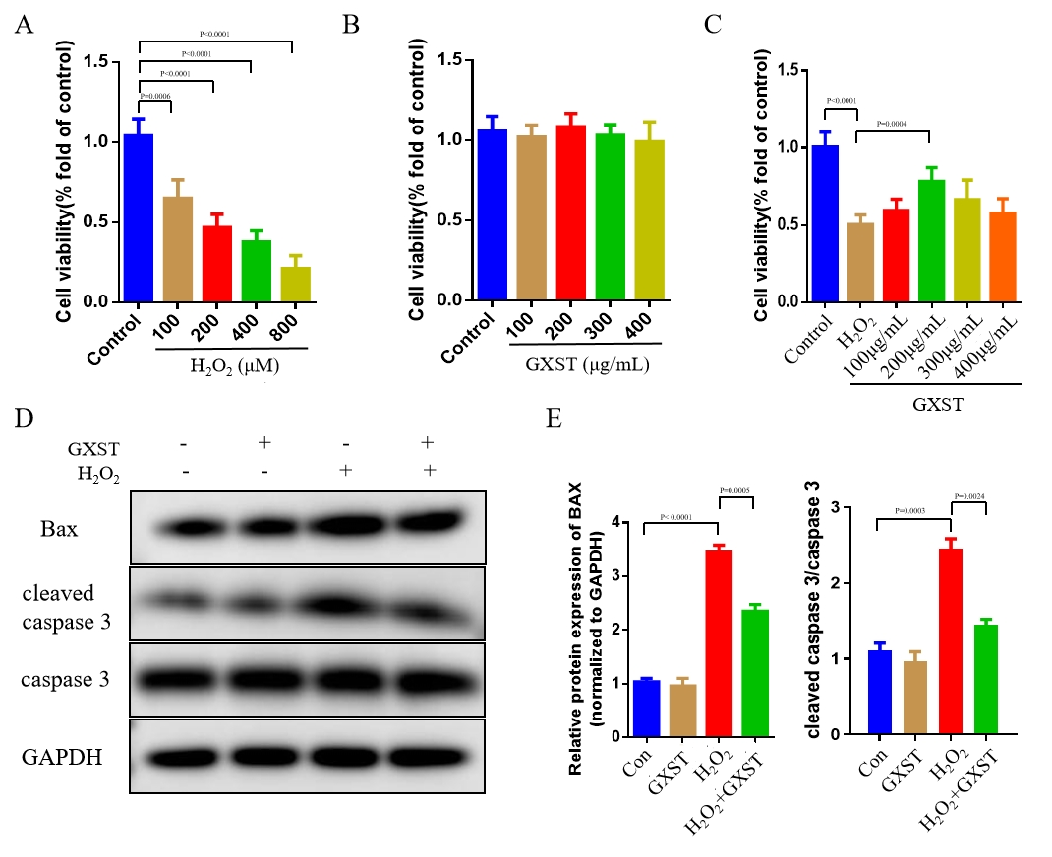

Supplement: Supplementary file 2 [file medi-103-e40118-s002.docx]
